# Copper-Catalyzed Intramolecular Olefinic C(sp^2^)–H Amidation for the Synthesis of *γ*-Alkylidene-*γ*-lactams

**DOI:** 10.3390/molecules28186682

**Published:** 2023-09-18

**Authors:** Kanako Nozawa-Kumada, Masahito Hayashi, Eunsang Kwon, Masanori Shigeno, Akira Yada, Yoshinori Kondo

**Affiliations:** 1 Graduate School of Pharmaceutical Sciences, Tohoku University, 6-3 Aoba, Aramaki, Aoba-ku, Sendai 980-8578, Miyagi, Japanmasanori.shigeno.e5@tohoku.ac.jp (M.S.); yoshinori.kondo.a7@tohoku.ac.jp (Y.K.); 2 Interdisciplinary Research Center for Catalytic Chemistry, National Institute of Advanced Industrial Science and Technology (AIST), Central 5, 1-1-1 Higashi, Tsukuba 305-8565, Ibaraki, Japan; a-yada@aist.go.jp; 3 Endowed Research Laboratory of Dimensional Integrated Nanomaterials, Graduate School of Science, Tohoku University, Aoba-ku, Sendai 980-8578, Miyagi, Japan; ekwon@tohoku.ac.jp; 4 Research and Analytical Center for Giant Molecules, Graduate School of Science, Tohoku University, Aoba-ku, Sendai 980-8578, Miyagi, Japan; 5 Japan Science and Technology Agency (JST), Precursory Research for Embryonic Science and Technology (PRESTO), Kawaguchi 332-0012, Saitama, Japan

**Keywords:** C(sp^2^)–H amidation, *γ*-alkylidene-*γ*-lactam, nitrogen-centered radical, cross-dehydrogenative coupling, copper-catalyst

## Abstract

Herein, we report the copper-catalyzed dehydrogenative C(sp^2^)–N bond formation of 4-pentenamides via nitrogen-centered radicals. This reaction provides a straightforward and efficient preparation method for *γ*-alkylidene-*γ*-lactams. Notably, we could controllably synthesize *α*,*β*-unsaturated- or *α*,*β*-saturated-*γ*-alkylidene-*γ*-lactams depending on the reaction conditions.

## 1. Introduction

Cross-dehydrogenative coupling (CDC) of C(sp^2^)–H/N–H bonds is one of the most straightforward methods for forming C(sp^2^)–N bonds [1,2,3,4], which are found in many pharmaceuticals, natural products, and materials [5,6,7,8]. Consequently, various approaches to accomplish CDC reactions have been reported, including *aza*-Wacker [9,10,11,12,13,14,15] and transition-metal-catalyzed, directing-group-assisted reactions [16,17,18,19]. The nitrogen-centered, radical-mediated reaction is considered a powerful strategy for dehydrogenative C(sp^2^)–N bond formation, which proceeds via the addition of N-radical species to the π-system of arenes or alkenes, following recovery of the π-system by oxidation or elimination, because it can preclude the use of precious transition-metals or the introduction and removal of directing groups. Over the past few decades, various such processes have been developed [20,21,22,23,24]; however, most of them have been applied to aryl C–H bonds, whereas olefinic C–H aminations are less explored [25].

**Scheme 1:**
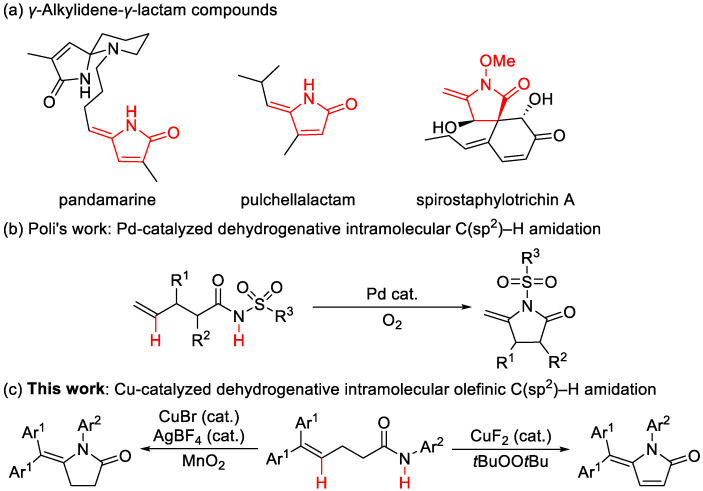
Syntheses of *γ*-alkylidene-*γ*-lactams [39].

*γ*-Alkylidene-*γ*-lactams are core structures of various natural and bioactive compounds (Scheme 1a) [26,27,28,29,30]. Several preparation methods have been developed, such as the cyclization of 4-ketoamides followed by dehydration [31,32,33], hetero Pauson–Khand reaction of ketenimines [34], cobalt-catalyzed reductive coupling of nitriles with acrylamides [35], Zn/TiCl_4_-mediated reductive coupling of imides with ketones [36], and photooxidative coupling of furans with amines [37,38]. Dehydrogenative C–N bond formation of 4-pentenamides is considered an efficient preparation approach because it can achieve atom- and step-economic syntheses. Recently, Poli et al. reported a palladium-catalyzed *γ*-methylidene-*γ*-lactam synthesis through the CDC between olefinic C–H and N–H bonds (Scheme 1b) [39]. However, to the best of our knowledge, this is the only example of its dehydrogenative synthesis; therefore, further development of such synthetic methods is highly desirable. Herein, we report a copper-catalyzed intramolecular dehydrogenative coupling reaction of 4-pentenamides for the synthesis of *γ*-alkylidene-*γ*-lactams via nitrogen-centered radicals (Scheme 1c). Notably, the reaction affords *α*,*β*-unsaturated- or *α*,*β*-saturated-*γ*-alkylidene-*γ*-lactams, which could be controlled by the reaction conditions. Furthermore, our method can be applied to various 4-pentenamides with methoxy, alkyl, halogen (fluoride, chloride, and bromide), trifluoromethyl, ester, and cyano substituents.

## 2. Results

We began our investigation of intramolecular C(sp^2^)–H amidation using *N*,5,5-triphenylpent-4-enamide **1a** as the model substrate (Table 1). When the reaction was performed in the presence of CuF_2_ (10 mol%), 4-*tert*-butylpyridine (1.0 equiv), and *t*BuOO*t*Bu (4.0 equiv) in 1,2-DCE at 120 °C for 18 h (condition A), 5-alkylidene-3-pyrrolin-2-one **2a** was obtained in high yield (entry 1). Reactions with other copper sources, such as CuCl, CuCl_2_, and Cu(OAc)_2_, also proceeded (entry 2). Subsequent screening of pyridine derivatives revealed that 4-*tert*-butylpyridine afforded the best yield of **2a** (entry 3). Performing the reaction in the absence of 4-*tert*-butylpyridine drastically decreased the yield of **2a** (entry 4). Other oxidants such as *tert*-butyl peroxide and *tert*-butyl peroxyacetate lowered the yield (entry 5). On the other hand, when MnO_2_ was used as the oxidant, 5-alkylidene-pyrrolidin-2-one derivative **3a**, which has a saturated lactam ring, was obtained with high selectivity (entry 6). Evaluation of various solvents revealed 1,2-DCE to be the most effective (entry 7). Lowering the reaction temperature to 100 °C resulted in a slightly decreased yield of the desired product **2a** (entry 8). Finally, the reaction could be scaled up to 1.0 mmol to afford **2a** in a good yield (entry 9).

**Scheme 2:**
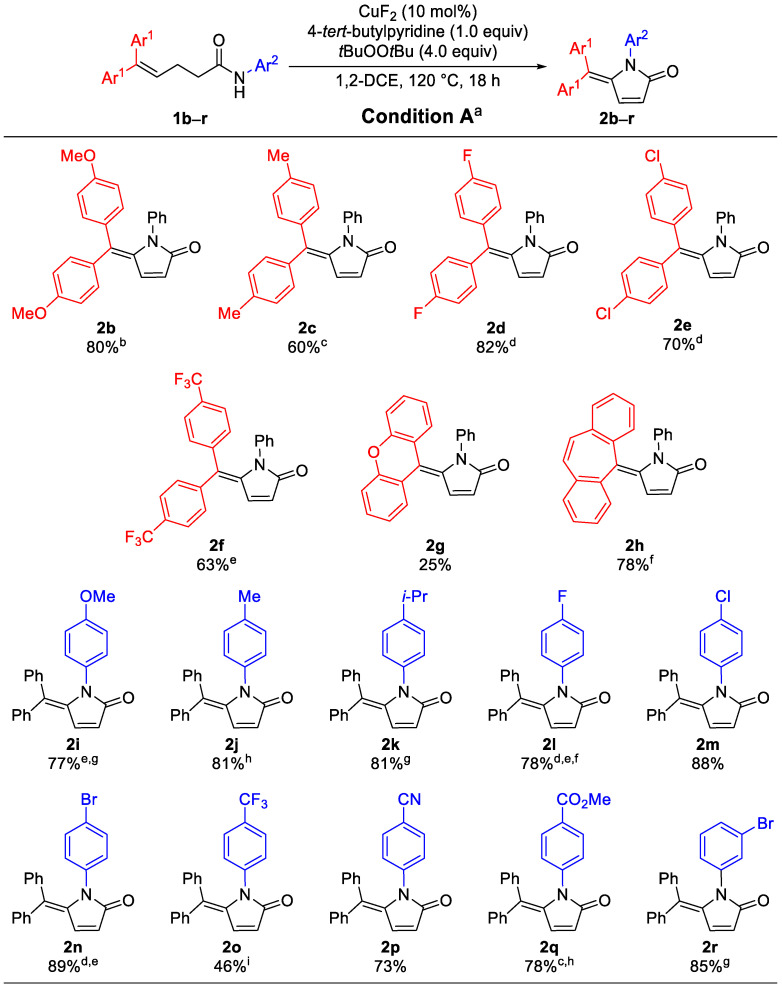
Substrate scope of 5-alkylidene-3-pyrrolin-2-one synthesis. ^a^ Isolated yields. Reaction conditions: **1** (0.20 mmol), CuF_2_ (0.020 mmol), 4-*tert*-butylpyridine (0.20 mmol), and *t*BuOO*t*Bu (0.80 mmol) in 1,2-DCE (2.5 mL) at 120 °C for 18 h. ^b^ Concentration of **1** was 0.10 M. ^c^ 5.0 equiv of *t*BuOO*t*Bu was used. ^d^ 75 mol% of 4-*tert*-butylpyridine was used. ^e^ 3.0 equiv of *t*BuOO*t*Bu was used. ^f^ Concentration of **1** was 0.13 M. ^g^ Reaction was conducted at 140 °C. ^h^ Concentration of **1** was 0.067 M. ^i^ Reaction conditions: **1** (0.20 mmol), CuF_2_ (0.020 mmol), 4-*tert*-butylpyridine (0.30 mmol), and *t*BuOO*t*Bu (1.0 mmol) in 1,2-DCE (1.0 mL) at 140 °C for 26 h.

With the optimized reaction conditions in hand, we next investigated the substrate scope for the 5-alkylidene-3-pyrrolin-2-one synthesis using *t*BuOO*t*Bu as the oxidant (Scheme 2). First, we explored the scope of diarylethylene acceptors. Substrates possessing functional groups such as methyl and methoxy groups, and halogen atoms on both benzene rings afforded the desired products in moderate to high yields (**2b**–**f**). The cyclization of the substrates with tricyclic scaffolds proceeded to furnish the corresponding products (**2g** and **2h**). Subsequently, we investigated substitution in the aniline ring and observed that the process afforded good-to-high amounts of cyclized products, regardless of the electronic properties of the aniline ring (**2i**–**r**).

**Scheme 3:**
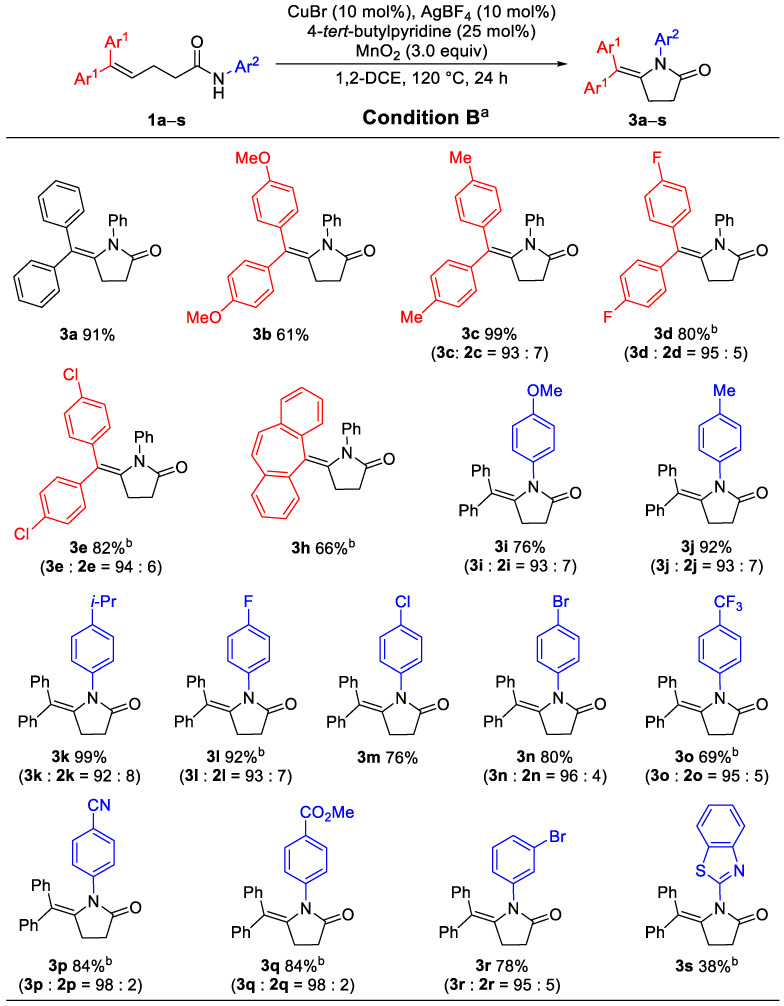
Substrate scope of 5-alkylidene-pyrrolidin-2-one synthesis. Isolated yields. Ratio was determined by ^1^H-NMR analysis. ^a^ Reaction conditions: **1** (0.20 mmol), CuBr (0.020 mmol), AgBF_4_ (0.020 mmol), 4-*tert*-butylpyridine (0.050 mmol), and MnO_2_ (0.60 mmol) in 1,2-DCE (1.5 mL) at 120 °C for 24 h. ^b^ Reaction was conducted at 140 °C.

Next, we investigated the synthesis of 5-alkylidene-pyrrolidin-2-ones using MnO_2_ as the oxidant (Scheme 3). When the reaction of **1a** was performed with CuBr (10 mol%), AgBF_4_ (10 mol%), 4-*tert*-butylpyridine (25 mol%), and MnO_2_ (3.0 equiv) in 1,2-DCE, at 120 °C, for 24 h (condition B) [40,41,42], 5-alkylidene-pyrrolidin-2-one **3a** was obtained in a high yield (for details, see Supplementary Materials). Using these optimized conditions, we then explored the scope and generality of the 5-alkylidene-pyrrolidin-2-one synthesis. Substrates with various functional groups on the diarylethylene moieties were first examined and afforded the corresponding cyclized products with high selectivities (**3b**–**e**). Subsequently, the reaction of amide bearing tricyclic dibenzo[*a*,*d*]cycloheptene scaffold proceeded smoothly (**3h**). Finally, the effect of aryl groups on the nitrogen atoms was investigated. Substrates possessing benzene derivatives on their amide nitrogen atoms smoothly underwent cyclization (**3i**–**r**). This process could also be applied to *N*-benzothiazole-substituted amide, which, however, afforded a low yield (**3s**).

**Scheme 4:**
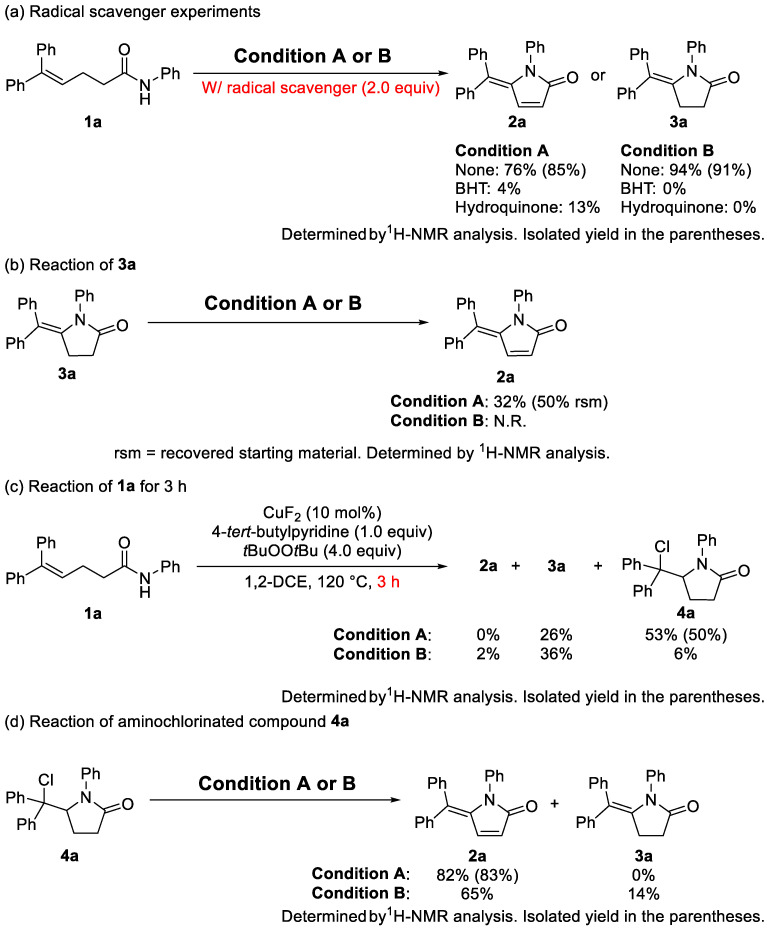
Control experiments.

Having studied the scope of the reaction, we next conducted experiments to obtain an insight into the reaction mechanism (Scheme 4). First, under both optimized conditions, the reactions were performed in the presence of the radical scavengers 2,6-di-*tert*-butyl-4-methylphenol (BHT) or hydroquinone, which led to a significant decrease in the yield of **2a** (condition A) or **3a** (condition B) (Scheme 4a). These results suggest that the reactions proceeded via radical processes. Next, to investigate the possibility of saturated-*r*-lactam **3a** acting as an intermediate for **2a**, the reaction using **3a** as a substrate was conducted under condition A (Scheme 4b). Consequently, **2a** was obtained in 32% yield, indicating that **3a** is one of the intermediates in the synthesis of **2a**. In contrast, we recovered the starting material **3a** under condition B. Furthermore, regarding *α*,*β*-unsaturated-*γ*-alkylidene-*γ*-lactam **2** synthesis, we shortened the reaction time to unveil the reaction intermediate, and aminochlorinated product **4a** was obtained in a good yield (Scheme 4c). The structure of **4a** was confirmed by X-ray crystallographic analysis (for details, see Supplementary Materials). Contrary, the reaction under condition B for 3 h produced only 6% of **4a**. Subsequently, the reaction starting from **4a** under condition A proceeded smoothly, suggesting that **4a** is a possible intermediate for the synthesis of *α*,*β*-unsaturated-*γ*-alkylidene-*γ*-lactam **2a** (Scheme 4d). Additionally, transformation of **4a** under condition B also proceeded to afford **2a** in good yield. From these results, we assume that the preference for either **2** or **3** is determined by whether aminochlorinated compound **4** is formed in situ.

**Scheme 5:**
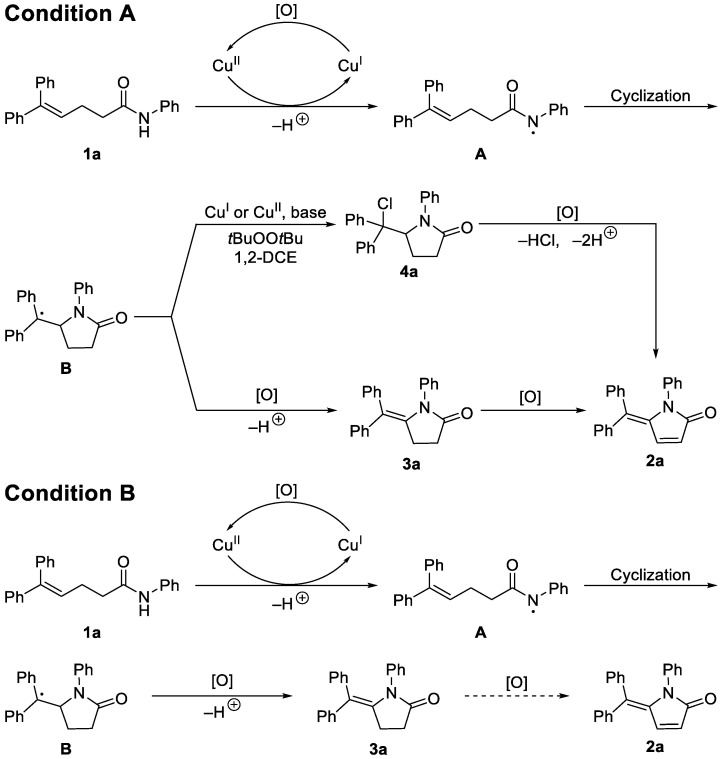
Plausible mechanism for copper-catalyzed intramolecular dehydrogenative coupling of **1a**.

Based on these experimental results, a plausible mechanism is proposed for copper-catalyzed intramolecular olefinic C(sp^2^)–H amidation (Scheme 5). For the formation of 5-alkylidene-3-pyrrolin-2-ones **2** under condition A, the nitrogen-centered radical **A** is initially generated by the Cu^II^ species [43,44,45]. It subsequently undergoes addition to an alkene moiety present in the substrate to afford the dibenzylic radical species **B**. For the next step, there are two possibilities: In the first, **B** is chlorinated under condition A to form the aminochlorinated product **4a** [46,47,48,49], and. subsequent HCl elimination and further oxidation results in the formation of **2a**. In the second, **3a** is generated by oxidation and deprotonation of **B**, and further oxidation of **3a** occurs to provide **2a** [50,51,52]. On the other hand, under condition B, **3a** is formed via oxidation and deprotonation of **B** as in condition A, however, no further transformation of **3a** occurs, resulting in the formation of **3a** as the major product. The detailed mechanism is unclear at present and needs to be clarified through further investigation.

## 3. Materials and Methods

### 3.1. Materials

Materials were purchased from Tokyo Kasei Co. (Tokyo, Japan), Sigma-Aldrich Inc. (St. Louis, MO, USA) and other commercial suppliers, and were used as received. Flash column chromatography was performed with Kanto silica gel 60 N (spherical, neutral, 70–230 mesh). Melting points were measured with a Yazawa micro melting point apparatus and uncorrected. IR spectra were recorded on a SHIMADZU IRAffinity. ^1^H NMR spectra were recorded on a JEOL JNMAL400 (400 MHz) spectrometer or a JEOL ECA600 (600 MHz) spectrometer. Chemical shifts are expressed in δ (parts per million, ppm) values and coupling constants are expressed in herts (Hz). ^1^H NMR spectra were referenced to tetramethylsilane as an internal standard or to a solvent signal (CDCl_3_: 7.26 ppm, DMSO-*d*_6_: 2.49 ppm). ^13^C NMR spectra were referenced to a solvent signal (CDCl_3_: 77.0 ppm, DMSO-*d*_6_: 39.5 ppm). ^19^F NMR spectra were referenced to 4-fluorotoluene as an internal standard (−118.0 ppm). The following abbreviations are used: s = singlet, d = doublet, t = triplet, q = quartet, dd, = double doublet, m = multiplet, and br.s. = broad singlet. Low- and high-resolution mass spectra (LRMS and HRMS) were obtained from Mass Spectrometry Resource, Graduate School of Pharmaceutical Sciences, Tohoku University, on a JEOL JMS-DX 303 and JMS700/JMS-T 100 GC spectrometer. The Bruker D8 VENTURE X-ray diffractometer was used to determine the structure of the grown crystals.

### 3.2. General Procedure for the Synthesis of 5-Alkylidene-pyrrolin-2-ones

In a glove box, amide **1** (0.20 mmol), CuF_2_ (2.0 mg, 0.020 mmol), 4-*tert*-butylpyridine (29.3 μL, 0.020 mmol), *t*BuOO*t*Bu (147.0 μL, 0.80 mmol), and 1,2-dichloroethane (2.5 mL) were added to a sealed tube. The mixture was stirred at 120 °C for 18 h. The reaction was diluted with water (10 mL) and extracted with chloroform (10 mL × 3). The organic layers were washed with brine (10 mL) and dried over MgSO_4_. The solvent was removed under a reduced pressure and the residue was purified by SiO_2_ column chromatography.

### 3.3. General Procedure for the Synthesis of 5-Alkylidene-pyrrolidin-2-ones

In a glove box, amide **1** (0.20 mmol), CuBr (2.8 mg, 0.020 mmol), AgBF_4_ (3.8 mg, 0.020 mmol), *tert*-butylpyridine (7.4 μL, 0.050 mmol), MnO_2_ (52.2 mg, 0.60 mmol), and 1,2-dichloroethane (1.5 mL) were added to a sealed tube. The mixture was stirred at 120 °C for 24 h. After the reaction, the mixture was filtered through Celite and a SiO_2_ pad with AcOEt, and then the solvent was removed under a reduced pressure. The residue was purified by SiO_2_ column chromatography.

### 3.4. Spectroscopic Data of Products

**5-(Diphenylmethylene)-1-phenyl-1,5-dihydro-2*H*-pyrrol-2-one** (**2a**). Obtained as yellow needles in 85% (55.0 mg, 0.20 mmol scale), 87% (286.6 mg, 1.0 mmol scale), recrystallized from DCM/hexane, mp. 160–163 °C. ^1^H NMR (400 MHz, CDCl_3_/TMS) δ (ppm): 7.38–7.36 (3H, m), 7.26–7.23 (2H, m), 7.21 (1H, d, *J* = 5.8 Hz), 7.00–6.83 (10H, m), 6.28 (1H, d, *J* = 5.4 Hz); ^13^C{^1^H} NMR (100 MHz, CDCl_3_/TMS) δ (ppm): 171.8, 140.5, 140.3, 138.1, 137.9, 135.8, 131.6, 130.94, 130.89, 128.4, 128.0, 127.9, 127.7, 127.2, 127.1, 126.0, 121.7; LRMS (EI) *m*/*z*: 323 (M^+^); HRMS (EI-TOF) Calcd. for C_23_H_17_NO: 323.1310, found: 323.1286; IR (neat): 3052, 1691, 1683, 1498, 1443, 1370, 1213, 1203, 1163, 1073, 968, 801, 774, 765, 756 cm^−1^.

**5-(Bis(4-methoxyphenyl)methylene)-1-phenyl-1,5-dihydro-2*H*-pyrrol-2-one** (**2b**). Obtained as red crystals in 80% (61.5 mg), recrystallized from DCM/hexane, mp. 177–178 °C. ^1^H NMR (600 MHz, CDCl_3_/TMS) δ (ppm): 7.19–7.17 (3H, m), 7.01 (2H, t, *J* = 7.6 Hz), 6.97–6.94 (3H, m), 6.90 (2H, d, *J* = 8.9 Hz), 6.77 (2H, d, *J* = 8.9 Hz), 6.42 (2H, d, *J* = 8.9 Hz), 6.23 (1H, d, *J* = 5.5 Hz), 3.85 (3H, s), 3.66 (3H, s); ^13^C{^1^H} NMR (150 MHz, CDCl_3_/TMS) δ (ppm): 171.9, 160.1, 159.3, 140.3, 136.8, 136.1, 133.1, 132.5, 131.0, 130.6, 127.8, 127.0, 125.73, 125.72, 120.6, 113.5, 112.7, 55.3, 55.2; LRMS (EI) *m*/*z*: 383 (M^+^); HRMS (EI-TOF) Calcd. for C_25_H_21_NO_3_: 383.1521, found: 383.1539; IR (neat): 1675, 1604, 1508, 1498, 1252, 1179, 1028, 969, 831 cm^−1^.

**5-(Di-*p*-tolylmethylene)-1-phenyl-1,5-dihydro-2*H*-pyrrol-2-one** (**2c**). Obtained as colorless needles in 60% (42.4 mg), recrystallized from DCM/hexane, mp. 161–162 °C. ^1^H NMR (600 MHz, DMSO-*d*_6_) δ (ppm): 7.21 (2H, d, *J* = 8.2 Hz), 7.18 (1H, d, *J* = 5.9 Hz), 7.11 (2H, d, *J* = 8.3 Hz), 6.99 (2H, t, *J* = 7.2 Hz), 6.94–6.91 (3H, m), 6.70 (2H, d, *J* = 8.2 Hz), 6.67 (2H, d, *J* = 8.2 Hz), 6.30 (1H, d, *J* = 5.9 Hz), 2.34 (3H, s), 2.07 (3H, s); ^13^C{^1^H} NMR (150 MHz, DMSO-*d*_6_) δ (ppm): 171.0, 140.6, 138.1, 137.5, 137.3, 137.1, 136.0, 135.1, 131.4, 130.6, 130.3, 128.8, 127.7, 127.6, 127.2, 125.5, 121.0, 20.8, 20.7; LRMS (EI) *m*/*z*: 351 (M^+^); HRMS (EI-TOF) Calcd. for C_25_H_21_NO: 351.1623, found: 351.1606; IR (neat): 1688, 1497, 1371, 1300, 1209, 1182, 969, 823, 802 cm^−1^.

**5-(Bis(4-fluorophenyl)methylene)-1-phenyl-1,5-dihydro-2*H*-pyrrol-2-one** (**2d**). Obtained as yellow crystals in 82% (58.9 mg), recrystallized from DCM/hexane, mp. 153–155 °C. ^1^H NMR (600 MHz, CDCl_3_/TMS) δ (ppm): 7.21 (2H, dd, *J* = 8.6, 5.2 Hz), 7.17 (1H, d, *J* = 5.5 Hz), 7.08 (2H, t, *J* = 8.6 Hz), 7.03 (2H, t, *J* = 7.6 Hz), 6.98 (1H, t, *J* = 7.6 Hz), 6.95–6.93 (2H, m), 6.80 (2H, dd, *J* = 8.9, 5.5 Hz), 6.59 (2H, t, *J* = 8.9 Hz), 6.29 (1H, d, *J* = 5.5 Hz); ^13^C{^1^H} NMR (150 MHz, CDCl_3_/TMS) δ (ppm): 171.6, 163.0 (d, *J*_C–F_ = 249.3 Hz), 162.1 (d, *J*_C–F_ = 249.3 Hz), 139.8, 138.2, 136.4 (d, *J*_C–F_ = 2.9 Hz), 135.7, 133.9 (d, *J*_C–F_ = 2.9 Hz), 133.2 (d, *J*_C–F_ = 8.6 Hz), 132.6 (d, *J*_C–F_ = 8.6 Hz), 128.2, 128.1, 127.1, 126.3, 122.1, 115.3 (d, *J*_C–F_ = 21.5 Hz), 114.4 (d, *J*_C–F_ = 22.9 Hz); ^19^F NMR (565 MHz, CDCl_3_) δ (ppm): −111.7, −111.9; LRMS (EI) *m*/*z*: 359 (M^+^); HRMS (EI-TOF) Calcd. for C_23_H_15_F_2_NO: 359.1122, found: 359.1110; IR (neat): 3039, 1688, 1594, 1505, 1497, 1366, 1305, 1231, 1156, 1101, 969, 844 cm^−1^.

**5-(Bis(4-chlorophenyl)methylene)-1-phenyl-1,5-dihydro-2*H*-pyrrol-2-one** (**2e**). Obtained as yellow crystals in 70% (54.6 mg), recrystallized from DCM/hexane, mp. 206–208 °C. ^1^H NMR (600 MHz, CDCl_3_/TMS) δ (ppm): 7.36 (2H, d, *J* = 8.2 Hz), 7.18–7.16 (3H, m), 7.05–6.99 (3H, m), 6.93–6.92 (2H, m), 6.86 (2H, d, *J* = 8.3 Hz), 6.74 (2H, d, *J* = 8.9 Hz), 6.31 (1H, d, *J* = 5.5 Hz); ^13^C{^1^H} NMR (150 MHz, CDCl_3_/TMS) δ (ppm): 171.5, 139.6, 138.7, 138.5, 136.0, 135.5, 134.9, 133.9, 132.7, 132.0, 128.5, 128.2, 127.6, 127.5, 127.1, 126.3, 122.4; LRMS (EI) *m*/*z*: 391 (M^+^); HRMS (EI-TOF) Calcd. for C_23_H_15_^35^Cl_2_NO: 391.0531, found: 391.0503; IR (neat): 3063, 1689, 1595, 1498, 1489, 1216, 1087, 1011, 806 cm^−1^.

**5-(Bis(4-(trifluoromethyl)phenyl)methylene)-1-phenyl-1,5-dihydro-2*H*-pyrrol-2-one** (**2f**). Obtained as yellow crystals in 63% (58.3 mg), recrystallized from DCM/hexane, mp. 168–170 °C. ^1^H NMR (600 MHz, CDCl_3_/TMS) δ (ppm): 7.66 (2H, d, *J* = 8.2 Hz), 7.37 (2H, d, *J* = 8.2 Hz), 7.21 (1H, d, *J* = 6.2 Hz), 7.14 (2H, d, *J* = 8.2 Hz), 7.02–6.95 (3H, m), 6.93–6.90 (4H, m), 6.37 (1H, d, *J* = 5.5 Hz); ^13^C{^1^H, ^19^F} NMR (150 MHz, CDCl_3_/TMS) δ (ppm): 171.3, 143.4, 140.9, 140.0, 139.4, 135.3, 131.8, 130.9, 130.7, 129.8, 128.3, 127.3, 126.7, 126.4, 125.4, 124.3, 123.9, 123.6, 123.4; ^19^F NMR (565 MHz, CDCl_3_) δ (ppm): −62.1, −62.5; LRMS (EI) *m*/*z*: 459 (M^+^); HRMS (EI-TOF) Calcd. for C_25_H_15_F_6_NO: 459.1058, found: 459.1048; IR (neat): 1696, 1612, 1322, 1155, 1121, 1065, 810 cm^−1^.

**1-Phenyl-5-(9*H*-xanthen-9-ylidene)-1,5-dihydro-2*H*-pyrrol-2-one** (**2g**). Obtained as yellow crystals in 25% (16.4 mg), recrystallized from DCM/hexane, mp. 180–183 °C. ^1^H NMR (600 MHz, CDCl_3_/TMS) δ (ppm): 8.02 (1H, d, *J* = 6.0 Hz), 7.62 (1H, dd, *J* = 7.5, 1.5 Hz), 7.40–7.37 (1H, m), 7.30–7.25 (2H, m), 7.07–7.02 (4H, m), 6.99 (1H, td, *J* = 7.7, 1.5 Hz), 6.92 (2H, dd, *J* = 8.0, 1.5 Hz), 6.70 (1H, dd, *J* = 7.5, 1.5 Hz), 6.40 (1H, d, *J* = 6.0 Hz), 6.38–6.35 (1H, m); ^13^C{^1^H} NMR (150 MHz, CDCl_3_/TMS) δ (ppm): 171.7, 154.2, 153.2, 138.8, 136.5, 134.4, 128.9, 128.7, 128.6, 128.1, 127.5, 126.6, 126.3, 124.2, 123.8, 122.5, 122.0, 121.6, 116.9, 116.3, 115.2; LRMS (EI) *m*/*z*: 337 (M^+^); HRMS (EI-TOF) Calcd. for C_23_H_15_NO_2_: 337.1103, found: 337.1096; IR (neat): 1684, 1593, 1495, 1447, 1199, 966, 872 cm^−1^.

**5-(5*H*-Dibenzo[*a*,*d*]**[7]**annulen-5-ylidene)-1-phenyl-1,5-dihydro-2*H*-pyrrol-2-one** (**2h**). Obtained as yellow crystals in 78% (54.0 mg), recrystallized from DCM/hexane, mp. 235–236 °C. ^1^H NMR (600 MHz, CDCl_3_/TMS) δ (ppm): 7.40–7.38 (1H, m), 7.35–7.32 (3H, m), 7.28–7.18 (3H, m), 7.08 (1H, d, *J* = 8.3 Hz), 6.99–6.89 (4H, m), 6.70–6.65 (3H, m), 6.29–6.24 (2H, m); ^13^C{^1^H} NMR (150 MHz, CDCl_3_/TMS) δ (ppm): 171.3, 138.4, 136.8, 136.3, 135.7, 135.3, 134.8, 133.5, 131.2, 130.9, 129.3, 128.5, 128.1, 128.0, 127.8, 127.54, 127.49, 127.4, 127.3, 126.9, 126.6, 126.3, 122.6; LRMS (EI) *m*/*z*: 347 (M^+^); HRMS (EI-TOF) Calcd. for C_25_H_17_NO: 347.1310, found: 347.1286; IR (neat): 1690, 1496, 1371, 1210, 1167, 786 cm^−1^.

**5-(Diphenylmethylene)-1-(4-methoxyphenyl)-1,5-dihydro-2*H*-pyrrol-2-one** (**2i**). Obtained as yellow crystals in 77% (54.1 mg), recrystallized from DCM/hexane, mp. 133–134 °C. ^1^H NMR (600 MHz, CDCl_3_/TMS) δ (ppm): 7.37–7.35 (3H, m), 7.23 (2H, dd, *J* = 7.9, 1.7 Hz), 7.18 (1H, d, *J* = 6.2 Hz), 6.99–6.96 (1H, m), 6.91 (2H, t, *J* = 7.6 Hz), 6.86–6.82 (4H, m), 6.53–6.51 (2H, m), 6.27 (1H, d, *J* = 6.2 Hz), 3.66 (3H, s); ^13^C{^1^H} NMR (150 MHz, CDCl_3_/TMS) δ (ppm): 172.0, 157.6, 140.6, 139.9, 138.3, 137.9, 131.6, 130.9, 130.6, 128.8, 128.33, 128.28, 128.0, 127.6, 127.2, 121.7, 113.4, 55.3; LRMS (EI) *m*/*z*: 353 (M^+^); HRMS (EI-TOF) Calcd. for C_24_H_19_NO_2_: 353.1416, found: 353.1410; IR (neat): 3052, 1690, 1613, 1512, 1443, 1248, 1159, 1026, 830, 806 cm^−1^.

**5-(Diphenylmethylene)-1-(*p*-tolyl)-1,5-dihydro-2*H*-pyrrol-2-one** (**2j**). Obtained as yellow crystals in 81% (54.4 mg), recrystallized from DCM/hexane, mp. 103–105 °C. ^1^H NMR (600 MHz, CDCl_3_/TMS) δ (ppm): 7.37–7.35 (3H, m), 7.24 (2H, dd, *J* = 7.9, 1.7 Hz), 7.18 (1H, d, *J* = 6.2 Hz), 6.95 (1H, t, *J* = 7.5 Hz), 6.88 (2H, t, *J* = 7.5 Hz), 6.83–6.81 (4H, m), 6.77 (2H, d, *J* = 8.2 Hz), 6.26 (1H, d, *J* = 6.2 Hz), 2.14 (3H, s); ^13^C{^1^H} NMR (150 MHz, CDCl_3_/TMS) δ (ppm): 171.9, 140.7, 140.0, 138.3, 138.0, 135.7, 133.2, 131.6, 130.9, 130.7, 128.5, 128.4, 128.0, 127.4, 127.1, 127.0, 121.8, 20.8; LRMS (EI) *m*/*z*: 337 (M^+^); HRMS (EI-TOF) Calcd. for C_24_H_19_NO: 337.1467, found: 337.1441; IR (neat): 3030, 1690, 1593, 1515, 1489, 1446, 1374, 1218, 1162, 971, 824 cm^−1^.

**5-(Diphenylmethylene)-1-(4-isopropylphenyl)-1,5-dihydro-2*H*-pyrrol-2-one** (**2k**). Obtained as orange crystals in 81% (58.9 mg), recrystallized from DCM/hexane, mp. 121–124 °C. ^1^H NMR (600 MHz, CDCl_3_/TMS) δ (ppm): 7.37–7.34 (3H, m), 7.25–7.23 (2H, m), 7.19 (1H, d, *J* = 5.5 Hz), 6.91 (1H, t, *J* = 7.2 Hz), 6.86–6.78 (8H, m), 6.27 (1H, d, *J* = 6.2 Hz), 2.69 (1H, sep, *J* = 6.9 Hz), 1.09 (6H, d, *J* = 6.9 Hz); ^13^C{^1^H} NMR (150 MHz, CDCl_3_/TMS) δ (ppm): 171.8, 146.7, 140.7, 139.9, 138.2, 137.9, 133.3, 131.6, 130.8, 130.7, 128.3, 128.0, 127.5, 127.1, 127.0, 125.9, 121.7, 33.6, 23.8; LRMS (EI) *m*/*z*: 365 (M^+^); HRMS (EI-TOF) Calcd. for C_26_H_23_NO: 365.1780, found: 365.1752; IR (neat): 2958, 2863, 1692, 1371, 1155, 805 cm^−1^.

**5-(Diphenylmethylene)-1-(4-fluorophenyl)-1,5-dihydro-2*H*-pyrrol-2-one** (**2l**). Obtained as yellow crystals in 78% (53.3 mg), recrystallized from DCM/hexane, mp. 153–155 °C. ^1^H NMR (600 MHz, CDCl_3_/TMS) δ (ppm): 7.39–7.36 (3H, m), 7.25–7.21 (3H, m), 7.01 (1H, t, *J* = 7.2 Hz), 6.94–6.90 (4H, m), 6.83 (2H, d, *J* = 6.9 Hz), 6.68 (2H, t, *J* = 8.6 Hz), 6.27 (1H, d, *J* = 6.2 Hz); ^13^C{^1^H, ^19^F} NMR (150 MHz, CDCl_3_/TMS) δ (ppm): 171.9, 160.6, 140.4, 140.3, 138.1, 137.9, 131.9, 131.7, 131.03, 130.95, 128.8, 128.6, 128.2, 128.0, 127.4, 121.7, 114.8; ^19^F NMR (565 MHz, CDCl_3_) δ (ppm): −115.4; LRMS (EI) *m*/*z*: 341 (M^+^); HRMS (EI-TOF) Calcd. for C_23_H_16_FNO: 341.1216, found: 341.1239; IR (neat): 1693, 1600, 1506, 1490, 1382, 1216, 974, 838, 808, 738 cm^−1^.

**1-(4-Chlorophenyl)-5-(diphenylmethylene)-1,5-dihydro-2*H*-pyrrol-2-one** (**2m**). Obtained as yellow crystals in 88% (63.2 mg), recrystallized from DCM/hexane, mp. 128–130 °C. ^1^H NMR (600 MHz, CDCl_3_/TMS) δ (ppm): 7.39–7.36 (3H, m), 7.25–7.22 (3H, m), 7.03 (1H, t, *J* = 7.2 Hz), 6.95–6.92 (4H, m), 6.89–6.87 (2H, m), 6.83 (2H, d, *J* = 6.9 Hz), 6.27 (1H, d, *J* = 6.2 Hz); ^13^C{^1^H} NMR (150 MHz, CDCl_3_/TMS) δ (ppm): 171.5, 140.4, 140.2, 137.8, 137.7, 134.5, 131.62, 131.57, 131.2, 130.9, 128.6, 128.2, 128.1, 127.99, 127.97, 127.4, 121.6; LRMS (EI) *m*/*z*: 357 (M^+^); HRMS (EI-TOF) Calcd. for C_23_H_16_^35^ClNO: 357.0920, found: 357.0910; IR (neat): 1688, 1593, 1554, 1493, 1446, 1374, 1203, 1089, 971, 833, 798 cm^−1^.

**1-(4-Bromophenyl)-5-(diphenylmethylene)-1,5-dihydro-2*H*-pyrrol-2-one** (**2n**). Obtained as yellow crystals in 89% (71.4 mg), recrystallized from DCM/hexane, mp. 149–151 °C. ^1^H NMR (600 MHz, CDCl_3_/TMS) δ (ppm): 7.39–7.36 (3H, m), 7.25–7.22 (3H, m), 7.10 (2H, d, *J* = 8.2 Hz), 7.04 (1H, t, *J* = 7.6 Hz), 6.94 (2H, t, *J* = 7.9 Hz), 6.83–6.81 (4H, m), 6.27 (1H, d, *J* = 5.5 Hz); ^13^C{^1^H} NMR (150 MHz, CDCl_3_/TMS) δ (ppm): 171.5, 140.5, 140.2, 137.8, 137.6, 135.0, 131.6, 131.2, 130.93, 130.89, 128.61, 128.58, 128.1, 128.0, 127.4, 121.7, 119.5; LRMS (EI) *m*/*z*: 401 (M^+^); HRMS (EI-TOF) Calcd. for C_23_H_16_^79^BrNO: 401.0415, found: 401.0427; IR (neat): 3068, 1684, 1489, 1162, 1068, 1015, 831, 798 cm^−1^.

**5-(Diphenylmethylene)-1-(4-(trifluoromethyl)phenyl)-1,5-dihydro-2*H*-pyrrol-2-one** (**2o**). Obtained as colorless needles in 46% (35.8 mg), recrystallized from DCM/hexane, mp. 136–137 °C. ^1^H NMR (600 MHz, CDCl_3_/TMS) δ (ppm): 7.41–7.37 (3H, m), 7.27–7.26 (3H, m), 7.23 (2H, d, *J* = 8.9 Hz), 7.06 (2H, d, *J* = 8.2 Hz), 6.97 (1H, t, *J* = 7.2 Hz), 6.90 (2H, t, *J* = 7.6 Hz), 6.82 (2H, d, *J* = 7.6 Hz), 6.30 (1H, d, *J* = 5.5 Hz); ^13^C{^1^H, ^19^F} NMR (150 MHz, CDCl_3_/TMS) δ (ppm): 171.3, 140.8, 140.1, 139.1, 137.8, 137.5, 131.7, 131.5, 130.8, 128.8, 128.24, 128.19, 127.9, 127.5, 127.2, 124.9, 123.8, 121.7; ^19^F NMR (565 MHz, CDCl_3_) δ (ppm): −62.1; LRMS (EI) *m*/*z*: 391 (M^+^); HRMS (EI-TOF) Calcd. for C_24_H_16_F_3_NO (M^+^): 391.1184, found: 391.1174; IR (neat): 1691, 1379, 1322, 1112, 1063, 975, 853, 800 cm^−1^.

**4-(2-(Diphenylmethylene)-5-oxo-2,5-dihydro-1*H*-pyrrol-1-yl)benzonitrile** (**2p**). Obtained as yellow crystals in 73% (51.0 mg), recrystallized from DCM/hexane, mp. 178–179 °C. ^1^H NMR (600 MHz, CDCl_3_/TMS) δ (ppm): 7.43–7.38 (3H, m), 7.29–7.25 (5H, m), 7.09–7.08 (2H, m), 7.03 (1H, t, *J* = 7.2 Hz), 6.94 (2H, t, *J* = 7.9 Hz), 6.86–6.84 (2H, m), 6.29 (1H, d, *J* = 6.2 Hz); ^13^C{^1^H} NMR (150 MHz, CDCl_3_/TMS) δ (ppm): 171.0, 141.2, 140.0, 139.8, 137.8, 137.0, 131.8, 131.7, 131.6, 130.9, 128.9, 128.5, 128.3, 127.6, 127.2, 121.5, 118.5, 109.0; LRMS (EI) *m*/*z*: 348 (M^+^); HRMS (EI-TOF) Calcd. for C_24_H_16_N_2_O: 348.1263, found: 348.1263; IR (neat): 2226, 1690, 1600, 1506, 1367, 1207, 1159, 970, 845, 799 cm^−1^.

**Methyl 4-(2-(diphenylmethylene)-5-oxo-2,5-dihydro-1*H*-pyrrol-1-yl)benzoate** (**2q**). Obtained as colorless needles in 78% (58.9 mg), recrystallized from DCM/hexane, mp. 193–195 °C. ^1^H NMR (600 MHz, CDCl_3_/TMS) δ (ppm): 7.67 (2H, d, *J* = 8.2 Hz), 7.41–7.38 (3H, m), 7.27–7.25 (3H, m), 7.04 (2H, d, *J* = 8.2 Hz), 6.94 (1H, t, *J* = 7.2 Hz), 6.90 (2H, t, *J* = 7.6 Hz), 6.85 (2H, d, *J* = 6.8 Hz), 6.28 (1H, d, *J* = 6.2 Hz), 3.84 (3H, s); ^13^C{^1^H} NMR (150 MHz, CDCl_3_/TMS) δ (ppm): 171.4, 166.4, 140.8, 140.2, 140.1, 137.9, 137.5, 131.65, 131.58, 131.57, 130.9, 129.3, 128.7, 128.2, 127.5, 127.1, 126.5, 121.6, 52.0; LRMS (EI) *m*/*z*: 381 (M^+^); HRMS (EI-TOF) Calcd. for C_25_H_19_NO_3_: 381.1365, found: 381.1341; IR (neat): 3062, 1722, 1696, 1601, 1507, 1437, 1361, 1275, 1167, 1102, 1072, 969, 863, 802 cm^−1^.

**1-(3-Bromophenyl)-5-(diphenylmethylene)-1,5-dihydro-2*H*-pyrrol-2-one** (**2r**). Obtained as yellow needles in 85% (68.2 mg), recrystallized from DCM/hexane, mp. 143–145 °C. ^1^H NMR (600 MHz, CDCl_3_/TMS) δ (ppm): 7.39–7.36 (3H, m), 7.25–7.23 (3H, m), 7.04 (1H, d, *J* = 8.2 Hz), 7.01–6.96 (5H, m), 6.89 (1H, t, *J* = 7.9 Hz), 6.86 (2H, d, *J* = 6.8 Hz), 6.27 (1H, d, *J* = 5.5 Hz); ^13^C{^1^H} NMR (150 MHz, CDCl_3_/TMS) δ (ppm): 171.4, 140.5, 140.2, 137.8, 137.5, 136.9, 131.6, 131.3, 130.6, 130.2, 129.1, 128.9, 128.6, 128.14, 128.10, 127.4, 125.8, 121.6, 121.3; LRMS (EI) *m*/*z*: 401 (M^+^); HRMS (EI-TOF) Calcd. for C_23_H_16_^79^BrNO (M^+^): 401.0415, found: 401.0438; IR (neat): 1690, 1587, 1476, 1364, 1201, 1152, 980, 802 cm^−1^.

**5-(Diphenylmethylene)-1-phenylpyrrolidin-2-one** (**3a**). Obtained as colorless plates in 91% (59.0 mg), recrystallized from DCM/hexane, mp. 146–148 °C. ^1^H NMR (600 MHz, CDCl_3_/TMS) δ (ppm): 7.31–7.29 (2H, m), 7.25–7.22 (1H, m), 7.19–7.18 (2H, m), 7.00–6.96 (4H, m), 6.90–6.88 (1H, m), 6.83–6.81 (3H, m), 6.72–6.71 (2H, m), 2.99–2.97 (2H, m), 2.71–2.68 (2H, m); ^13^C{^1^H} NMR (150 MHz, CDCl_3_/TMS) δ (ppm): 176.3, 142.1, 139.2, 137.9, 136.1, 130.1, 130.0, 128.2, 127.9, 127.1, 126.7, 126.2, 126.0, 125.9, 120.2, 30.9, 28.3; LRMS (EI) *m*/*z*: 325 (M^+^); HRMS (EI-TOF) Calcd. for C_23_H_19_NO: 325.1467, found: 325.1456; IR (neat): 3046, 1723, 1620, 1595, 1495, 1359, 1160, 1027, 773, 751 cm^−1^.

**5-(Bis(4-methoxyphenyl)methylene)-1-phenylpyrrolidin-2-one** (**3b**). Obtained as yellow crystals in 61% (46.8 mg), recrystallized from DCM/hexane, mp. 164–166 °C. ^1^H NMR (600 MHz, CDCl_3_/TMS) δ (ppm): 7.09 (2H, d, *J* = 8.9 Hz), 6.99–6.98 (4H, m), 6.91–6.90 (1H, m), 6.84 (2H, d, *J* = 8.9 Hz), 6.61 (2H, d, *J* = 8.9 Hz), 6.36 (2H, d, *J* = 8.9 Hz), 3.80 (3H, s), 3.62 (3H, s), 2.96 (2H, t, *J* = 7.7 Hz), 2.68 (2H, t, *J* = 7.7 Hz); ^13^C{^1^H} NMR (150 MHz, CDCl_3_/TMS) δ (ppm): 176.3, 158.3, 157.7, 136.7, 136.1, 134.6, 132.0, 131.1, 131.0, 127.9, 126.1, 125.9, 119.5, 113.5, 112.6, 55.2, 55,1, 31.1, 28.4; LRMS (EI) *m*/*z*: 385 (M^+^); HRMS (EI-TOF) Calcd. for C_25_H_23_NO_3_: 385.1678, found: 385.1706; IR (neat): 2969, 2835, 1713, 1606, 1508, 1358, 1179, 1027, 829, 761 cm^−1^.

**5-(Di-*p*-tolylmethylene)-1-phenylpyrrolidin-2-one** (**3c**). Obtained as colorless needles in 99% (69.4 mg, **3c**:**2c** = 93:7), recrystallized from DCM/hexane, mp. 208–211 °C. ^1^H NMR (600 MHz, CDCl_3_/TMS) δ (ppm): 7.10 (2H, d, *J* = 8.2 Hz), 7.06 (2H, d, *J* = 8.2 Hz), 6.98–6.95 (4H, m), 6.90–6.87 (1H, m), 6.61 (2H, d, *J* = 8.2 Hz), 6.58 (2H, d, *J* = 8.2 Hz), 2.97 (2H, t, *J* = 7.6 Hz), 2.68 (2H, t, *J* = 7.6 Hz), 2.34 (3H, s), 2.09 (3H, s); ^13^C{^1^H} NMR (150 MHz, CDCl_3_/TMS) δ (ppm): 176.3, 139.3, 137.3, 136.4, 136.3, 136.2, 135.5, 129.9, 129.8, 128.8, 127.85, 127.76, 126.2, 125.7, 120.2, 31.1, 28.4, 21.1, 20.9; LRMS (EI) *m*/*z*: 353 (M^+^); HRMS (EI-TOF) Calcd. for C_25_H_23_NO: 353.1780, found: 353.1775; IR (neat): 3019, 2927, 1732, 1636, 1496, 1369, 1228, 1154, 815 cm^−1^.

**5-(Bis(4-fluorophenyl)methylene)-1-phenylpyrrolidin-2-one** (**3d**). Obtained as colorless crystals in 80% (58.2 mg, **3d**:**2d** = 95:5), recrystallized from DCM/hexane, mp. 179–181 °C. ^1^H NMR (600 MHz, CDCl_3_/TMS) δ (ppm): 7.14–7.12 (2H, m), 7.03–6.94 (7H, m), 6.67–6.64 (2H, m), 6.52 (2H, t, *J* = 8.6 Hz), 2.95 (2H, t, *J* = 7.9 Hz), 2.70 (2H, t, *J* = 7.9 Hz); ^13^C{^1^H} NMR (150 MHz, CDCl_3_/TMS) δ (ppm): 176.2, 161.6 (^1^*J*_C–F_ = 246.4 Hz), 161.0 (^1^*J*_C–F_ = 246.4 Hz), 138.4, 137.8 (^4^*J*_C–F_ = 4.3 Hz), 135.9, 135.1 (^4^*J*_C–F_ = 2.9 Hz), 131.55 (^3^ *J*_C–F_ = 7.2 Hz), 131.48 (^3^*J*_C–F_ = 8.6 Hz), 128.1, 126.4, 126.3, 117.8, 115.2 (^2^*J*_C–F_ = 21.5 Hz), 114.1 (^2^*J*_C–F_ = 21.5 Hz), 30.8, 28.2; ^19^F NMR (565 MHz, CDCl_3_) δ (ppm): −114.5, −115.2; LRMS (EI) *m*/*z*: 361 (M^+^); HRMS (EI-TOF) Calcd. for C_23_H_17_F_2_NO: 361.1278, found: 361.1262; IR (neat): 3040, 1736, 1632, 1598, 1505, 1370, 1293, 1153, 831, 758 cm^−1^.

**5-(Bis(4-chlorophenyl)methylene)-1-phenylpyrrolidin-2-one** (**3e**). Obtained as colorless needles in 82% (65.5 mg, **3e**:**2e** = 94:6), recrystallized from DCM/hexane, mp. 204–206 °C. ^1^H NMR (600 MHz, CDCl_3_/TMS) δ (ppm): 7.28 (2H, d, *J* = 8.2 Hz), 7.09 (2H, d, *J* = 8.2 Hz), 7.04–7.01 (2H, m), 6.98 (1H, d, *J* = 6.9 Hz), 6.95 (2H, d, *J* = 7.6 Hz), 6.79 (2H, d, *J* = 8.2 Hz), 6.61 (2H, d, *J* = 8.2 Hz), 2.96 (2H, t, *J* = 7.7 Hz), 2.71 (2H, t, *J* = 7.7 Hz); ^13^C{^1^H} NMR (150 MHz, CDCl_3_/TMS) δ (ppm): 176.1, 140.0, 139.1, 137.3, 135.8, 132.7, 132.0, 131.30, 131.26, 128.5, 128.2, 127.4, 126.5, 126.3, 117.4, 30.7, 28.2; LRMS (EI) *m*/*z*: 393 (M^+^); HRMS (EI-TOF) Calcd. for C_23_H_17_^35^Cl_2_NO: 393.0687, found: 393.0671; IR (neat): 3064, 2944, 1732, 1612, 1488, 1355, 1156, 1012, 828, 756 cm^−1^.

**5-(5*H*-Dibenzo[*a*,*d*][7]annulen-5-ylidene)-1-phenylpyrrolidin-2-one** (**3h**). Obtained as colorless crystals in 66% (46.3 mg), recrystallized from DCM/hexane, mp. 199–200 °C. ^1^H NMR (600 Hz, DMSO-*d*_6_) δ (ppm): 7.42–7.36 (3H, m), 7.27–7.24 (1H, m), 7.02–6.89 (6H, m), 6.83–6.56 (5H, m), 3.12–3.08 (1H, m), 2.70–2.64 (1H, m), 2.45–2.41 (1H, m), 2.26–2.21 (1H, m); ^13^C{^1^H} NMR (150 Hz, DMSO-*d*_6_), δ (ppm): 176.3, 138.3, 137.1, 136.8, 136.4, 134.9, 133.8, 131.3, 130.9, 128.9, 128.7, 128.3, 128.1, 127.8, 127.3, 127.2, 127.0, 126.4, 126.3, 125.6, 114.1, 28.7, 24.2; LRMS (EI) *m*/*z*: 349 (M^+^); HRMS (EI-TOF) Calcd. for C_25_H_19_NO: 349.1467, found: 349.1465; IR (neat): 3017, 1717, 1636, 1497, 1370, 1239, 1171, 803, 738 cm^−1^.

**5-(Diphenylmethylene)-1-(4-methoxyphenyl)pyrrolidin-2-one** (**3i**). Obtained as colorless needles in 76% (54.7 mg, **3i**:**2i** = 93:7), recrystallized from DCM/hexane, mp. 158–161 °C. ^1^H NMR (600 MHz, CDCl_3_/TMS) δ (ppm): 7.29 (2H, t, *J* = 7.6 Hz), 7.22 (1H, t, *J* = 7.2 Hz), 7.18 (2H, d, *J* = 6.9 Hz), 6.88–6.84 (5H, m), 6.71–6.70 (2H, m), 6.50 (2H, d, *J* = 8.9 Hz), 3.65 (3H, s), 2.96 (2H, t, *J* = 7.9 Hz), 2.67 (2H, t, *J* = 7.9 Hz); ^13^C{^1^H} NMR (150 MHz, CDCl_3_/TMS) δ (ppm): 176.6, 157.6, 142.2, 139.1, 138.2, 130.2, 129.9, 129.1, 128.2, 127.5, 127.1, 126.6, 125.9, 119.7, 113.4, 55.4, 30.7, 28.1; LRMS (EI) *m*/*z*: 355 (M^+^); HRMS (EI-TOF) Calcd. for C_24_H_21_NO_2_: 355.1572, found: 355.1549; IR (neat): 3257, 1700, 1636, 1511, 1444, 1242, 1031, 741 cm^−1^.

**5-(Diphenylmethylene)-1-(*p*-tolyl)pyrrolidin-2-one** (**3j**). Obtained as yellow oil in 92% (62.7 mg, **3j**:**2j** = 93:7). ^1^H NMR (600 MHz, CDCl_3_/TMS) δ (ppm): 7.30 (2H, t, *J* = 7.5 Hz), 7.22 (1H, tt, *J* = 7.4, 1.5 Hz), 7.19–7.17 (2H, m), 6.85–6.80 (5H, m), 6.76 (2H, d, *J* = 8.1 Hz), 6.71–6.69 (2H, m), 2.98–2.95 (2H, m), 2.69–2.67 (2H, m), 2.13 (3H, s); ^13^C{^1^H} NMR (150 MHz, CDCl_3_/TMS) δ (ppm): 176.5, 142.2, 139.2, 138.1, 135.8, 133.5, 130.2, 130.0, 128.5, 128.2, 127.1, 126.6, 126.2, 125.7, 119.9, 30.8, 28.2, 20.8; LRMS (EI) *m*/*z*: 339 (M^+^); HRMS (EI-TOF) Calcd. for C_24_H_21_NO: 339.1623, found: 339.1617; IR (neat): 3057, 1718, 1631, 1512, 1364, 1229, 1167, 1030, 816, 751 cm^−1^.

**5-(Diphenylmethylene)-1-(4-isopropylphenyl)pyrrolidin-2-one** (**3k**). Obtained as colorless oil in 99% (72.5 mg, **3k**:**2k** = 92:8). ^1^H NMR (600 MHz, CDCl_3_/TMS) δ (ppm): 7.29 (2H, t, *J* = 7.6 Hz), 7.22 (1H, t, *J* = 7.6 Hz), 7.18 (2H, d, *J* = 7.6 Hz), 6.85 (2H, d, *J* = 8.9 Hz), 6.80–6.79 (5H, m), 6.67 (2H, dd, *J* = 6.5, 3.1 Hz), 2.96 (2H, t, *J* = 7.9 Hz), 2.70–2.65 (3H, m), 1.09 (6H, d, *J* = 6.8 Hz); ^13^C{^1^H} NMR (150 MHz, CDCl_3_/TMS) δ (ppm): 176.5, 146.8, 142.2, 139.1, 138.1, 133.6, 130.1, 129.9, 128.2, 127.0, 126.5, 126.3, 126.0, 125.8, 119.7, 33.7, 30.7, 28.1, 23.9; LRMS (EI) *m*/*z*: 367 (M^+^); HRMS (EI-TOF) Calcd. for C_26_H_25_NO: 367.1936, found: 367.1925; IR (neat): 3018, 2959, 1723, 1630, 1512, 1364, 1300, 1229, 1167, 832 cm^−1^.

**5-(Diphenylmethylene)-1-(4-fluorophenyl)pyrrolidin-2-one** (**3l**). Obtained as colorless needle in 92% (63.4 mg, **3l**:**2l** = 93:7), recrystallized from DCM/hexane, mp. 128–130 °C. ^1^H NMR (600 MHz, CDCl_3_/TMS) δ (ppm): 7.32–7.29 (2H, m), 7.25–7.22 (1H, m), 7.18–7.16 (2H, m), 6.96–6.93 (2H, m), 6.91–6.85 (3H, m), 6.72–6.70 (2H, m), 6.68–6.65 (2H, m), 3.00–2.97 (2H, m), 2.70–2.68 (2H, m); ^13^C{^1^H} NMR (150 MHz, CDCl_3_/TMS) δ (ppm): 176.4, 160.5 (^1^*J*_C–F_ = 245.0 Hz), 141.9, 139.1, 137.9, 132.1 (^4^*J*_C–F_ = 2.9 Hz) 130.2, 129.9, 128.2, 128.0 (^3^*J*_C–F_ = 8.6 Hz), 127.3, 126.8, 126.2, 120.2, 114.8 (^2^*J*_C–F_ = 22.9 Hz), 30.8, 28.2; ^19^F NMR (565 MHz, CDCl_3_) δ (ppm): −115.1; LRMS (EI) *m*/*z*: 343 (M^+^); HRMS (EI-TOF) Calcd. For C_23_H_18_FN: 343.1372, found: 343.1352; IR (neat): 3058, 1724, 1633, 1604, 1507, 1368, 1299, 1228, 1153, 910, 752 cm^−1^.

**1-(4-Chlorophenyl)-5-(diphenylmethylene)pyrrolidin-2-one** (**3m**). Obtained as colorless needles in 76% (55.4 mg), recrystallized from DCM/hexane, mp. 165–167 °C. ^1^H NMR (600 MHz, CDCl_3_/TMS) δ (ppm): 7.31 (2H, t, *J* = 7.6 Hz), 7.25–7.22 (1H, m), 7.18–7.17 (2H, m), 6.94–6.86 (7H, m), 6.72–6.70 (2H, m), 2.98 (2H, t, *J* = 7.9 Hz), 2.69 (2H, t, *J* = 7.9 Hz); ^13^C{^1^H} NMR (150 MHz, CDCl_3_/TMS) δ (ppm): 176.1, 141.7, 139.0, 137.6, 134.6, 131.4, 130.1, 129.9, 128.2, 128.0, 127.42, 127.38, 126.8, 126.2, 120.5, 30.9, 28.2; LRMS (EI) *m*/*z*: 359 (M^+^); HRMS (EI-TOF) Calcd. for C_23_H_18_^35^ClNO: 359.1077, found: 359.1092; IR (neat): 3076, 2929, 1719, 1636, 1492, 1364, 1300, 1233, 1165, 1088, 833 cm^−1^.

**1-(4-Bromophenyl)-5-(diphenylmethylene)pyrrolidin-2-one** (**3n**). Obtained as colorless needles in 80% (64.7 mg, **3n**:**2n** = 96:4), recrystallized from DCM/hexane, mp. 188–189 °C. ^1^H NMR (600 MHz, CDCl_3_/TMS) δ (ppm): 7.30 (2H, t, *J* = 7.6 Hz), 7.25–7.24 (1H, m), 7.17 (2H, d, *J* = 7.5 Hz), 7.08 (2H, d, *J* = 8.9 Hz), 6.91 (1H, t, *J* = 9.6 Hz), 6.87–6.85 (4H, m), 6.70 (2H, d, *J* = 7.6 Hz), 2.98 (2H, t, *J* = 7.9 Hz), 2.68 (2H, t, *J* = 7.9 Hz); ^13^C{^1^H} NMR (150 MHz, CDCl_3_/TMS) δ (ppm): 176.1, 141.7, 139.0, 137.5, 135.1, 131.0, 130.1, 129.9, 128.2, 127.7, 127.4, 126.8, 126.2, 120.5, 119.4, 30.9, 28.2; LRMS (EI) *m*/*z*: 403 (M^+^); HRMS (EI-TOF) Calcd. For C_23_H_18_^79^BrNO: 403.0572, found: 403.0550; IR (neat): 3074, 1718, 1636, 1488, 1365, 1301, 1235, 1166, 1067, 1012 cm^−1^.

**5-(Diphenylmethylene)-1-(4-(trifluoromethyl)phenyl)pyrrolidin-2-one** (**3o**). Obtained as yellow crystals in 69% (54.4 mg, **3o**:**2o** = 95:5), recrystallized from DCM/hexane, mp. 169–170 °C. ^1^H NMR (600 MHz, CDCl_3_/TMS) δ (ppm): 7.32 (2H, t, *J* = 7.6 Hz), 7.26–7.18 (5H, m), 7.10 (2H, d, *J* = 8.3 Hz), 6.84–6.83 (3H, m), 6.69 (2H, dd, *J* = 7.6, 1.4 Hz), 3.01 (2H, t, *J* = 7.7 Hz), 2.72 (2H, t, *J* = 7.7 Hz); ^13^C{^1^H, ^19^F} NMR (150 MHz, CDCl_3_/TMS) δ (ppm): 176.0, 141.5, 139.1, 139.0, 137.2, 130.0, 129.9, 128.2, 127.8, 127.4, 126.9, 126.4, 126.3, 125.0, 123.7, 121.0, 31.0, 28.2; ^19^F NMR (565 MHz, CDCl_3_) δ (ppm): −62.1; LRMS (EI) *m*/*z*: 393 (M^+^); HRMS (EI-TOF) Calcd. For C_24_H_18_F_3_NO: 393.1340, found: 393.1328; IR (neat): 3060, 1721, 1636, 1592, 1490, 1366, 1324, 1232, 1161, 851, 753 cm^−1^.

**4-(2-(Diphenylmethylene)-5-oxopyrrolidin-1-yl)benzonitrile** (**3p**). Obtained as colorless crystals in 84% (59.1 mg, **3p**:**2p** = 98:2), recrystallized from DCM/hexane, mp. 200–201 °C. ^1^H NMR (600 MHz, CDCl_3_/TMS) δ (ppm): 7.32 (2H, t, *J* = 7.5 Hz), 7.28–7.25 (3H, m), 7.18 (2H, d, *J* = 6.9 Hz), 7.14 (2H, d, *J* = 8.3 Hz), 6.90–6.86 (3H, m), 6.72–6.71 (2H, m), 3.01 (2H, t, *J* = 7.7 Hz), 2.73 (2H, t, *J* = 7.7 Hz); ^13^C{^1^H} NMR (150 MHz, CDCl_3_/TMS) δ (ppm): 175.8, 141.2, 140.0, 139.0, 136.73, 136.72, 131.8, 129.9, 128.3, 127.6, 127.1, 126.7, 126.2, 121.8, 118.5, 108.9, 31.2, 28.3; LRMS (EI) *m*/*z*: 350 (M^+^); HRMS (EI-TOF) Calcd. For C_24_H_18_N_2_O: 350.1419, found: 350.1396; IR (neat): 3044, 2224, 1721, 1635, 1601, 1506, 1355, 1227, 1162, 844 cm^−1^.

**Methyl 4-(2-(diphenylmethylene)-5-oxopyrrolidin-1-yl)benzoate** (**3q**). Obtained as colorless crystals in 84% (64.4 mg, **3q**:**2q** = 98:2), recrystallized from DCM/hexane, mp. 191–192 °C. ^1^H NMR (600 MHz, CDCl_3_/TMS) δ (ppm): 7.65 (2H, d, *J* = 8.9 Hz), 7.31 (2H, t, *J* = 7.6 Hz), 7.26–7.24 (1H, m), 7.19 (2H, d, *J* = 6.8 Hz), 7.09 (2H, d, *J* = 8.2 Hz), 6.85–6.81 (3H, m), 6.73 (2H, d, *J* = 7.6 Hz), 3.84 (3H, s), 3.00 (2H, t, *J* = 7.9 Hz), 2.71 (2H, t, *J* = 7.9 Hz); ^13^C{^1^H} NMR (150 MHz, CDCl_3_/TMS) δ (ppm): 176.0, 166.4, 141.6, 140.2, 139.0, 137.3, 130.0, 129.3, 128.2, 127.4, 127.1, 126.9, 126.40, 126.39, 125.6, 121.4, 52.0, 31.2, 28.4; LRMS (EI) *m*/*z*: 383 (M^+^); HRMS (EI-TOF) Calcd. For C_25_H_21_NO_3_: 383.1521, found: 383.1511; IR (neat): 3056, 2952, 1723, 1710, 1630, 1439, 1361, 1278, 1227, 855, 765 cm^−1^.

**1-(3-Bromophenyl)-5-(diphenylmethylene)pyrrolidin-2-one** (**3r**). Obtained as yellow crystals in 78% (63.2 mg, **3r**:**2r** = 95:5), recrystallized from DCM/hexane, mp. 138–140 °C. ^1^H NMR (600 MHz, CDCl_3_/TMS) δ (ppm): 7.31 (2H, t, *J* = 7.5 Hz), 7.25–7.23 (1H, m), 7.18 (2H, d, *J* = 7.5 Hz), 7.06 (1H, s), 7.01 (2H, d, *J* = 8.0 Hz), 6.92–6.85 (4H, m), 6.74 (2H, d, *J* = 6.5 Hz), 2.99 (2H, t, *J* = 7.8 Hz), 2.69 (2H, t, *J* = 7.8 Hz); ^13^C{^1^H} NMR (150 MHz, CDCl_3_/TMS) δ (ppm): 176.0, 141.6, 139.0, 137.4, 137.1, 129.93, 129.91, 129.4, 129.1, 129.0, 128.2, 127.4, 126.8, 126.3, 124.9, 121.4, 120.8, 30.9, 28.2; LRMS (EI) *m*/*z*: 403 (M^+^); HRMS (EI-TOF) Calcd. For C_23_H_18_^79^BrNO: 403.0572, found: 403.0580; IR (neat): 3057, 1724, 1631, 1590, 1571, 1476, 1352, 1220, 1155, 764 cm^−1^.

**1-(Benzo[*d*]thiazol-2-yl)-5-(diphenylmethylene)pyrrolidin-2-one** (**3s**). Obtained as colorless needles in 38% (29.9 mg), recrystallized from DCM/hexane, mp. 192–193 °C. ^1^H NMR (600 MHz, CDCl_3_/TMS) δ (ppm): 7.64 (1H, d, *J* = 8.2 Hz), 7.45 (1H, d, *J* = 8.3 Hz), 7.34–7.23 (6H, m), 7.19 (1H, t, *J* = 7.2 Hz), 6.93 (2H, d, *J* = 7.6 Hz), 6.82 (2H, t, *J* = 7.9 Hz), 6.64 (1H, t, *J* = 7.5 Hz), 3.08 (2H, t, *J* = 7.5 Hz), 2.79 (2H, t, *J* = 7.5 Hz); ^13^C{^1^H} NMR (150 MHz, CDCl_3_/TMS) δ (ppm): 175.1, 154.2, 148.3, 140.9, 140.3, 134.8, 133.1, 130.2, 129.0, 128.1, 127.3, 127.2, 126.4, 125.7, 125.5, 124.3, 122.1, 120.8, 31.5, 28.5; LRMS (EI) *m*/*z*: 382 (M^+^); HRMS (EI-TOF) Calcd. for C_24_H_18_N_2_OS (M^+^): 382.1140, found: 382.1167; IR (neat): 3048, 2914, 1723, 1635, 1512, 1279, 1234, 1168, 749 cm^−1^.

**5-(Chlorodiphenylmethyl)-1-phenylpyrrolidin-2-one** (**4a**). Obtained as colorless needles in 50% (36.2 mg), recrystallized from DCM/hexane, mp. 156–157 °C. ^1^H NMR (600 MHz, CDCl_3_/TMS) δ (ppm): 7.43 (2H, d, *J* = 6.9 Hz), 7.31–7.23 (5H, m), 7.09 (4H, d, *J* = 4.8 Hz), 7.05–7.01 (3H, m), 7.00–6.96 (1H, m), 5.62 (1H, d, *J* = 8.3 Hz), 2.62–2.54 (1H, m), 2.34–2.28 (2H, m), 2.24–2.15 (1H, m); ^13^C{^1^H} NMR (150 MHz, CDCl_3_/TMS) δ (ppm): 175.9, 142.6, 141.2, 138.7, 128.3, 128.2, 128.1, 127.9, 127.8, 127.5, 125.8, 125.77, 125.76, 82.5, 67.5, 30.5, 24.2; LRMS (FAB) *m*/*z*: 362 (M+H)^+^; HRMS (FAB-EB) Calcd. For C_23_H_21_^35^ClNO (M+H)^+^: 362.1312, found: 362.1324; IR (neat): 3067, 1689, 1599, 1499, 1404, 1291, 1039, 749 cm^−1^.

## 4. Conclusions

In conclusion, we developed a novel copper-catalyzed intramolecular olefinic C(sp^2^)–H amidation of 4-pentenamides. This reaction is an efficient approach to synthesize *α*,*β*-unsaturated-*γ*-alkylidene-*γ*-lactams or the *α*,*β*-saturated derivatives, which were controllable by varying the reaction conditions. The reaction exhibits good tolerance to various functional groups including alkyl, methoxy, halogen (fluoride, chloride, and bromide), trifluoromethyl, ester, and cyano moieties. Further studies aimed at expanding the substrate scope and elucidating the reaction mechanism are currently underway.

## Figures and Tables

**Table 1:** Effect of reaction parameters ^a^.

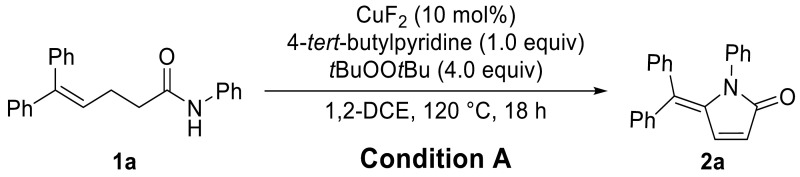		
Entry	Variation from Standard Conditions	Yield (%) ^b^
1	None	76 (85)
2	CuCl, CuCl_2_, or Cu(OAc)_2_ instead of CuF_2_	39–75
3	Pyridine, DMAP, or 1,10-phen instead of 4-*tert*-butylpyridine	21–66
4	W/O 4-*tert*-butylpyridine	29
5	*t*BuOOH, *t*BuOOAc, or PIDA instead of *t*BuOO*t*Bu	0–35
6	MnO_2_ instead of *t*BuOO*t*Bu	5 ^c^
7	DCM, toluene, or PhCF_3_ instead of 1,2-DCE	0–44
8	At 100 °C	73
9	1.0 mmol Scale	83 (87)

^a^ Reaction conditions: **1a** (0.20 mmol), 4-*tert*-butylpyridine (0.20 mmol), *t*BuOO*t*Bu (0.80 mmol), 1,2-DCE (2.5 mL), at 120 °C for 18 h under Ar. ^b^ Determined by ^1^H-NMR using 1,1,2-trichloroethane as the internal standard. Isolated yield in parentheses. ^c^ 5-(Diphenylmethylene)-1-phenylpyrrolidin-2-one **3a** was obtained in 89% yield instead of **2a**.

## Data Availability

The Supplementary Materials are available free of charge on the website.

## Supplementary Materials

The following supporting information can be downloaded at: https://www.mdpi.com/article/10.3390/molecules28186682/s1, Table S1: Effect of reaction parameter for the synthesis of **2a**; Table S2: Copper optimization for the synthesis of **3a**; Table S3: Optimization of copper and silver salts for the synthesis of **3a**; Table S4: Detailed reaction conditions for the synthesis of **3a**; Table S5: Crystal data and structure refinements for **4a**; Scheme S1: Unsuccessful substrates under condition B; Figure S1: ORTEP diagram of **4a** with thermal ellipsoids drawn at 50% probability level (CCDC No. 2284469). References [53,54,55] are cited in the supplementary materials.

## References

[1] Chen X., Li L., Wan C., Liu J.-B. (2020). FeCl_3_-catalyzed sequential cyclization for the construction of 12-aryl 5,7-dihydropyrido[2,3-*b*:6,5-*b*’]diindoles. Tetrahedron Lett..

[2] Liu J.-B., Ren M., Lai X., Qiu G. (2021). Iron-catalyzed stereoselective haloamidation of amide-tethered alkynes. Chem. Commun..

[3] Ren S., Huang K., Liu J.-B., Zhang L., Hou M., Qiu G. (2022). Palladium-catalyzed cyclization of 1-alkynyl-8-iodonaphthalene and double isocyanides for the synthesis of acenaphtho[1,2-*b*]pyrroles. Chin. Chem. Lett..

[4] Ren M., Yan X., Lai X., Liu J.-B., Zhou H., Qiu G. (2022). Nitrenium ion-based *ipso*-addition and *ortho*-cyclization of arenes under photo and iron dual-catalysis. Mol. Catal..

[5] Zhang C., Tang C., Jiao N. (2012). Recent advances in copper–catalyzed dehydrogenative functionalization *via* a single electron transfer (SET) process. Chem. Soc. Rev..

[6] Louillat M.-L., Patureau F.W. (2014). Oxidative C–H amination reactions. Chem. Soc. Rev..

[7] Kim H., Chang S. (2016). Transition-Metal-Mediated Direct C–H Amination of Hydrocarbons with Amine Reactants: The Most Desirable but Challenging C–N Bond-Formation Approach. ACS Catal..

[8] Park Y., Kim Y., Chang S. (2017). Transition Metal-Catalyzed C–H Amination: Scope, Mechanism, and Applications. Chem. Rev..

[9] Bozell J.J., Hegedus L.S. (1981). Palladium-Assisted Functionalization of Olefins: A New Amination of Electron-Deficient Olefins. J. Org. Chem..

[10] Hosokawa T., Takano M., Kuroki Y., Murahashi S. (1992). Palladium(II)-Catalyzed Amidation of Alkenes. Tetrahedron Lett..

[11] Beller M., Eichberger M., Trauthwein H. (1997). Anti-Markovnikov Functionalization of Olefins: Rhodium-Catalyzed Oxidative Aminations of Styrenes. Angew. Chem. Int. Ed..

[12] Timokhin V.I., Anastasi N.R., Stahl S.S. (2003). Dioxygen-Coupled Oxidative Amination of Styrene. J. Am. Chem. Soc..

[13] Brice J.L., Harang J.E., Timokhin V.I., Anastasi N.R., Stahl S.S. (2005). Aerobic Oxidative Amination of Unactivated Alkenes Catalyzed by Palladium. J. Am. Chem. Soc..

[14] Jiménez M.V., Pérez-Torrente J.J., Bartolomé M.I., Lahoz F.J., Oro L.A. (2010). Rational design of efficient rhodium catalysts for the anti-markovnikov oxidative amination of styrene. Chem. Commun..

[15] Wang X., Wang Q., Xue Y., Sun K., Wu L., Zhang B. (2020). An organoselenium-catalyzed N^1^- and N^2^-selective aza-Wacker reaction of alkenes with benzotriazoles. Chem. Commun..

[16] Liu L., Wang N., Dai C., Han Y., Yang S., Huang Z., Zhao Y. (2019). Rh(III)-Catalyzed Direct Amination of Aromatic Ketoximes Enabled by Potassium Acetate. Eur. J. Org. Chem..

[17] Yu L., Yang C., Yu Y., Liu D., Hu L., Xiao Y., Song Z.-N., Tan Z. (2019). Ammonia as Ultimate Amino Source in Synthesis of Primary Amines via Nickel-Promoted C–H Bond Amination. Org. Lett..

[18] Grandhi G.S., Dana S., Mandal A., Baidya M. (2020). Copper-Catalyzed 8-Aminoquinoline-Directed Oxidative C–H/N–H Coupling for *N*-Arylation of Sulfoximines. Org. Lett..

[19] Vu H.-M., Yong J.-Y., Chen F.-W., Li X.-Q., Shi G.-Q. (2020). Rhodium-Catalyzed C(sp^2^)–H Amidation of Azine with Sulfonamides. J. Org. Chem..

[20] Kärkäs M.D. (2017). Photochemical Generation of Nitrogen-Centered Amidyl, Hydrazonyl, and Imidyl Radicals: Methodology Developments and Catalytic Applications. ACS Catal..

[21] Chen N., Xu H.-C. (2021). Electrochemical generation of nitrogen-centered radicals for organic synthesis. Green Synth. Catal..

[22] Kwon K., Simons R.T., Nandakumar M., Roizen J.L. (2022). Strategies to Generate Nitrogen-centered Radicals That May Rely on Photoredox Catalysis: Development in Reaction Methodology and Applications in Organic Synthesis. Chem. Rev..

[23] Pratley C., Fenner S., Murphy J.A. (2022). Nitrogen-Centered Radicals in Functionalization of sp^2^ Systems: Generation, Reactivity, and Applications in Synthesis. Chem. Rev..

[24] Gao Y., Zhao Q., Li L., Ma Y.-N. (2022). Synthesis of Six-Membered *N*-Heterocycle Frameworks Based on Intramolecular Metal-Free *N*-Centered Radical Chemistry. Chem. Rec..

[25] Xiong T., Zhang Q. (2016). New amination strategies based on nitrogen-centered radical chemistry. Chem. Soc. Rev..

[26] Alvi K.A., Casey A., Nair B.G. (1998). Pulchellalactam: A CD45 Protein Tyrosine Phosphatase Inhibitor from the Marine Fungus *Corollospora pulchella*. J. Antibiot..

[27] Li W.-R., Lin S.T., Hsu N.-M., Chern M.-S. (2002). Efficient Total Synthesis of Pulchellalactam, a CD45 Protein Tyrosine Phosphatase Inhibitor. J. Org. Chem..

[28] Nay B., Riache N., Evanno L. (2009). Chemistry and biology of non-tetramic γ-hydroxy-γ-lactams and γ-alkylidene-γ-lactams from natural sources. Nat. Prod. Rep..

[29] Kalaitzakis D., Noutsias D., Vassilikogiannakis G. (2015). First Total Synthesis of Pandamarine. Org. Lett..

[30] Wang J., Chen F., Liu Y., Liu Y., Li K., Yang X., Liu S., Zhou X., Yang J. (2018). Spirostaphylotrichin X from a Marine-Derived Fungus as an Anti-influenza Agent Targeting RNA Polymerase PB2. J. Nat. Prod..

[31] Abell A.D., Oldham M.D., Taylor J.M. (1995). Synthesis of Cyclic Acylated Enamino Esters from Enol Lactones, 4-Keto Amides, and 5-Hydroxy Lactams. J. Org. Chem..

[32] Park B.R., Lim C.H., Lim J.W., Kim J.N. (2012). Facile Synthesis of 5-Alkylidene-1,5-dihydropyrrol-2-ones from Morita-Baylis-Hillman Adducts. Bull. Korean Chem. Soc..

[33] Mardjan M.I.D., Mayooufi A., Parrain J.-L., Thibonnet J., Commeiras L. (2020). Straightforward Access to a Great Diversity of Complex Biorelevant *γ*-Lactams Thanks to a Tunable Cascade Multicomponent Process. Org. Process Res. Dev..

[34] Saito T., Sugizaki K., Osada H., Kutsumura N., Otani T. (2010). A Hetero Pauson-Khand Reaction of Ketenimines: A New Synthetic Method for γ-Exomethylene-α,β-unsaturated γ-Lactams. Heterocycles.

[35] Wong Y.-C., Parthasarathy K., Cheng C.-H. (2009). Cobalt-Catalyzed Regioselective Synthesis of Pyrrolidinone Derivatives by Reductive Coupling of Nitriles and Acrylamides. J. Am. Chem. Soc..

[36] Kise N., Kinameri S., Sakurai T. (2019). Reductive coupling of aliphatic cyclic imides and ω-amidoesters with benzophenones by low-valent titanium: Synthesis of 5-diarylmethylene-1,5-dihydropyrrol-2-ones, 6-diarylmethyl-2-pyridones, and ω-(diarylmethylene)lactams. Tetrahedron.

[37] Kalaitzakis D., Kampouropoulos I., Sofiadis M., Montagnon T., Vassilikogiannakis G. (2022). Access to high value sp^3^-rich frameworks using photocatalyzed [2 + 2]-cycloadditions of γ-alkylidene-γ-lactams. Chem. Commun..

[38] Kalaitzakis D., Bosveli A., Montagnon T., Vassilikogiannakis G. (2022). Sequential Visible Light-Induced Reactions Using Different Photocatalysts: Transformation of Furans into 2-Pyridones via γ-Lactams Using a New Ring Expansion Reaction. Chem. Eur. J..

[39] Lorion M.M., Duarte F.J.S., Calhorda M.J., Oble J., Poli G. (2016). Opening the Way to Catalytic Aminopalladation/Proxicyclic Dehydropalladation: Access to Methylidene *γ*-Lactams. Org. Lett..

[40] Ishida Y., Nakamura I., Terada M. (2018). Copper-Catalyzed Domino [1,3]/[1,2] Rearrangement for the Efficient Synthesis of Multisubstituted *ortho*-Anisidines. J. Am. Chem. Soc..

[41] Fang Y.-Q., Dang L. (2020). Mechanistic Study of Domino Rearrangement-Promoted Meta C–H Activation in 2-Methyl-*N*-methoxyaniline via Cu(NHC)^+^: Motivation and Selectivity. Org. Lett..

[42] Nakamura I., Masukawa K., Ishida Y., Terada M. (2021). Cu-Catalyzed [1,3]-Alkoxy Rearrangement/Diels–Alder Cascade Reactions via in Situ Generation of Functionalized *ortho*-Quinol Imines. Org. Lett..

[43] Chemler S.R. (2011). Evolution of copper(II) as a new alkene amination promoter and catalyst. J. Organomet. Chem..

[44] Liwosz T.W., Chemler S.R. (2013). Copper-Catalyzed Oxidative Amination and Allylic Amination of Alkenes. Chem. Eur. J..

[45] Nozawa-Kumada K., Ono K., Kurosu S., Shigeno M., Kondo Y. (2022). Copper-catalyzed aerobic benzylic C(sp^3^)–H lactonization of 2-alkylbenzamides *via* N-centered radicals. Org. Biomol. Chem..

[46] Takeuchi K., Ishida S., Nishikata T. (2017). Dichloromethane as a Chlorination Reagent for α-Bromocarbonyl Compounds in the Presence of a Copper Catalyst. Chem. Lett..

[47] Bhakat M., Khatua B., Guin J. (2022). Photocatalytic Aerobic Coupling of Azaarenes and Alkanes via Nontraditional Cl^•^ Generation. Org. Lett..

[48] Wang H.-W., Wu J.-X., Huang X.-Q., Li D.-C., Wang S.-N., Lu Y., Dou J.-M. (2022). Rh^III^-Catalyzed C–H *N*-Heteroarylation and Esterification Cascade of Carboxylic Acid with Organoboron Reagents and 1,2-Dichloroethane in One-Pot Synthesis. Org. Lett..

[49] Ji J., Jiang L., Wang Z., Bin Z., You J., Yang Y. (2022). Copper-Catalyzed Oxidative C–H Annulation of Quinolines with Dichloroethane toward Benzoquinoliziniums Using an In Situ Activation Strategy. Org. Lett..

[50] Chen H., Liu L., Huang T., Chen J., Chen T. (2020). Direct Dehydrogenation for the Synthesis of α,β-Unsaturated Carbonyl Compounds. Adv. Synth. Catal..

[51] Gnaim S., Vantourout J.C., Serpier F., Echeverria P.-G., Baran P.S. (2021). Carbonyl Desaturation: Where Does Catalysis Stand?. ACS Catal..

[52] Gnaim S., Takahira Y., Wilke H.R., Yao Z., Li J., Delbrayelle D., Echeverria P.-G., Vantourout J.C., Baran P.S. (2021). Electrochemically driven desaturation of carbonyl compounds. Nat. Chem..

[53] Dolomanov O.V., Bourhis L.J., Gildea R.J., Howard JA K., Puschmann H. (2009). *OLEX2*: A complete structure solution, refinement and analysis program. J. Appl. Crystallogr..

[54] Bourhis L.J., Dolomanov O.V., Gildea R.J., Howard JA K., Puschmann H. (2015). The anatomy of a comprehensive constrained, restrained refinement program for the modern computing environment—Olex2 dissected. Acta Crystallogr. A Found. Adv..

[55] Sheldrick G.M. (2015). Crystal structure refinement with SHELXL. Acta Crystallogr..

