# Supplementary material for: Copper-Catalyzed Intramolecular Olefinic C(sp2)–H Amidation for the Synthesis of γ-Alkylidene-γ-lactams
Source: Molecules. 2023 Sep 18;28(18):6682. doi: 10.3390/molecules28186682 (PMC10537769; doi:10.3390/molecules28186682)

Supplementary Materials

Copper-Catalyzed Intramolecular Olefinic C(sp^2)–H Amidation for the Synthesis of *γ*-Alkylidene-*γ*-lactams

Kanako Nozawa-Kumada[1,2,*], Masahito Hayashi[1], Eunsang Kwon[3,4], Masanori Shigeno[1,5], Akira Yada[2] and Yoshinori Kondo[1]

[1] Graduate School of Pharmaceutical Sciences, Tohoku University, 6-3 Aoba, Aramaki, Aoba-ku, Sendai, Miyagi 980-8578, Japan

[2] Interdisciplinary Research Center for Catalytic Chemistry, National Institute of Advanced Industrial Science and Technology (AIST), Central 5, 1-1-1 Higashi, Tsukuba, Ibaraki 305-8565, Japan

[3] Endowed Research Laboratory of Dimensional Integrated Nanomaterials, Graduate School of Science, Tohoku University, Aoba-ku, Sendai, Miyagi 980-8578, Japan

[4] Research and Analytical Center for Giant Molecules, Graduate School of Science, Tohoku University, Aoba-ku, Sendai, Miyagi 980-8578, Japan

[5] Japan Science and Technology Agency (JST), Precursory Research for Embryonic Science and Technology (PRESTO), Kawaguchi, Saitama 332-0012, Japan

[*] Correspondence: k.kumada@aist.go.jp; Tel.: +81-29-861-5908

Table of Contents

1. General comments **S3**

2. Materials **S3**

3. Preparation of starting materials **S3**

4. General procedure for the synthesis of 5-alkylidene-pyrrolin-2-ones **S13**

5. General procedure for the synthesis of 5-alkylidene-pyrrolidin-2-ones **S21**

6. Mechanistic experiment **S30**

7. X-ray crystallographic data for 4a **S31**

8. ^{1}H-, ^{13}C-, and ^{19}F-NMR spectra **S34**

1. General comments

Melting points were measured with a Yazawa micro melting point apparatus and uncorrected. IR spectra were recorded on a SHIMADZU IRAffinity. ^{1}H NMR spectra were recorded on a JEOL JNMAL400 (400 MHz) spectrometer or a JEOL ECA600 (600 MHz) spectrometer. Chemical shifts are expressed in δ (parts per million, ppm) values and coupling constants are expressed in herts (Hz). ^{1}H NMR spectra were referenced to tetramethylsilane as an internal standard or to a solvent signal ($CDCl_3$: 7.26 ppm, DMSO-d_6: 2.49 ppm). ^{13}C NMR spectra were referenced to a solvent signal ($CDCl_3$: 77.0 ppm, DMSO-d_6: 39.5 ppm). ^{19}F NMR spectra were referenced to 4-fluorotoluene as an internal standard (−118.0 ppm). All NMR spectra were measured at 25 °C using a 5 mm diameter glass NMR tube. The following abbreviations are used: s = singlet, d = doublet, t = triplet, q = quartet, dd, = double doublet, m = multiplet, and br.s. = broad singlet. Low and high resolution mass spectra (LRMS and HRMS) were obtained from Mass Spectrometry Resource, Graduate School of Pharmaceutical Sciences, Tohoku University, on a JEOL JMS-DX 303 and JMS700/JMS-T 100 GC spectrometer. HRMS were performed using polyethylene glycol (PEG) or perfluorokerosene (PEK) as an internal standard and 3-nitrobenzyl alcohol (3-NBA) or glycerol (GLY) as matrix. The Bruker D8 VENTURE x-ray diffractometer was used to determine the structure of the grown crystals.

2. Materials

Materials were purchased from Tokyo Kasei Co., Aldrich Inc. and other commercial suppliers and were used as received. Flash column chromatography was performed with Kanto silica gel 60 N (spherical, neutral, 70−230 mesh).

3. Preparation of starting materials

Method A

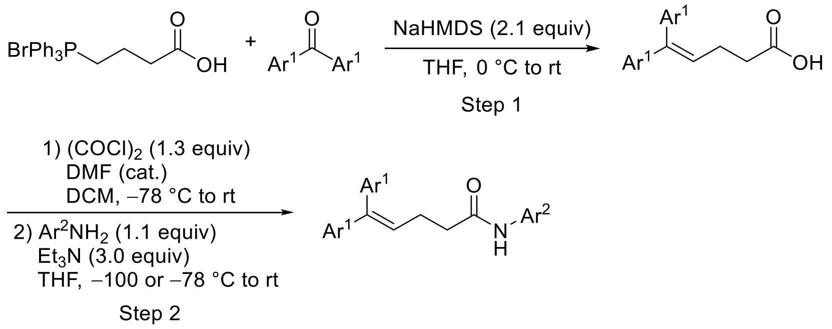

Step 1: (3-Carboxypropyl)triphenylphosphonium bromide (2.1 g, 5.0 mmol) was suspended in THF (20 mL). The colorless suspension was cooled to 0 °C and NaHMDS (1.1 M in hexane, 12.5 mL, 13.8 mmol) was added dropwise. Then, the resulting bright orange solution was stirred at 0 °C for 30 min. To the reaction mixture was added a solution of ketone (6.0 mmol) in THF (10

mL) dropwise at 0 °C and the reaction mixture was stirred overnight at rt. The reaction mixture was diluted with 1M HCl to pH 1, then extracted with AcOEt (20 mL x 3). The organic layers were washed with brine (10 mL) and dried over $MgSO_4$. The solvent was removed under reduced pressure and the residue was purified by SiO_2 column chromatography (eluent: hexane/AcOEt = 3/1).

Step 2: To a solution of the carboxylic acid (3.0 mmol) in dichloromethane (10 mL) were added dimethylformamide (2 drops) and oxalyl chloride (0.53 mL, 3.8 mmol) at −78 °C under N_2 atmosphere. The resulting mixture was stirred overnight at rt. The solvent was then removed under reduced pressure to afford the corresponding crude acyl chloride. To a stirred solution of the acyl chloride in THF (10 mL), aniline derivative (3.6 mmol) and triethylamine (1.3 mL, 9.0 mmol) were added dropwise using a dropping funnel at −100 or −78 °C. After complete addition, the reaction mixture was stirred for 6 h at rt. The solvent was then removed under reduced pressure and 3 M HCl aq. (10 mL) was added to the mixture. The resulting solution was extracted with AcOEt (10 mL x 3). The organic layers were washed with sat. Na_2CO_3 aq. (10 mL) and brine (10 mL). The solvent was dried over $MgSO_4$ and removed under reduced pressure. The residue was recrystallization to give the corresponding amide compound.

Method B

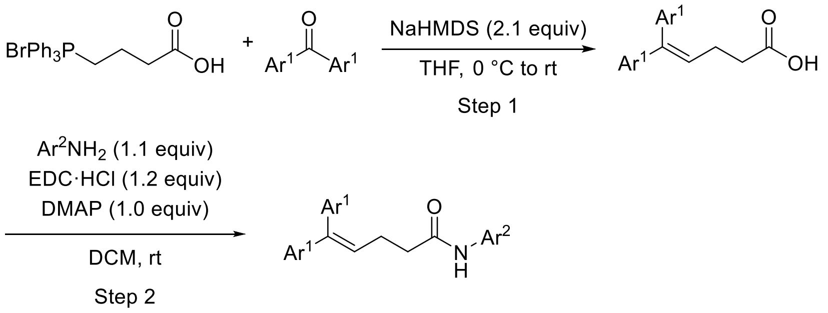

Step 1: (3-Carboxypropyl)triphenylphosphonium bromide (2.1 g, 5.0 mmol) was suspended in THF (20 mL). The colorless suspension was cooled to 0 °C and NaHMDS (1.1 M in hexane, 12.5 mL, 13.8 mmol) was added dropwise. Then, the resulting bright orange solution was stirred at 0 °C for 30 min. To the reaction mixture was added a solution of ketone (6.0 mmol) in THF (10 mL) dropwise at 0 °C and the reaction mixture was stirred overnight at rt. The reaction mixture was diluted with 1M HCl to pH 1, then extracted with AcOEt (10 mL x 3). The organic layers were washed with brine (15 mL) and dried over $MgSO_4$. The solvent was removed under reduced pressure and the residue was purified by SiO_2 column chromatography (eluent: hexane/AcOEt = 3/1).

Step 2: To a solution of the carboxylic acid (3.0 mmol) in dichloromethane (10 mL) were added EDC·HCl (690.1 mg, 3.6 mmol), *N,N*-dimethylaminopyridine (366.5 mg, 3.0 mmol), and amine

(3.3 mmol) at rt under N_2 atmosphere. The resulting mixture was stirred overnight at rt. After completion of the reaction, water (10 mL) was added to the reaction mixture. The resulting solution was extracted with AcOEt (20 mL x 3). The organic layers were washed with brine (10 mL). The solvent was dried over $MgSO_4$ and removed under reduced pressure. The residue was recrystallization to give the corresponding amide compound.

Method C

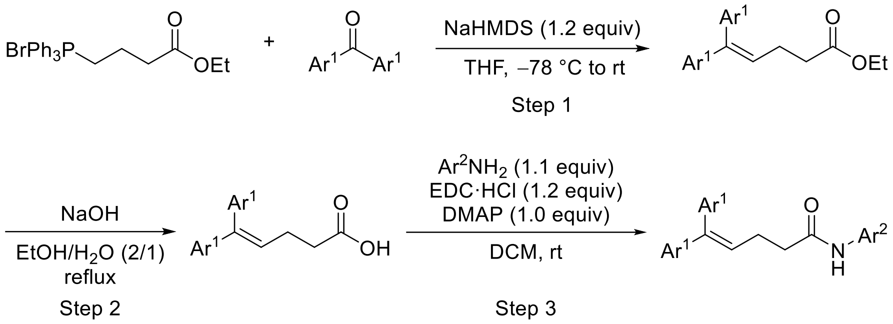

Step 1: (3-Ethoxycarbonylpropyl)triphenylphosphonium bromide (457.0 mg, 1.0 mmol) was suspended in THF (5 mL). The colorless suspension was cooled to −78℃ and NaHMDS (1.1 M in hexane, 1.2 mL, 1.2 mmol) was added dropwise. Then, the resulting bright orange solution was stirred at 0 °C for 30 min. To the reaction mixture was added a solution of ketone (1.1 mmol) in THF (5 mL) dropwise at 0 °C and the reaction mixture was stirred overnight at rt. The reaction mixture was diluted with 1M HCl (10 mL) and extracted with AcOEt (10 mL x 3). The organic layers were washed with brine (15 mL) and dried over $MgSO_4$. The solvent was removed under reduced pressure and the residue was purified by SiO_2 column chromatography (eluent: hexane/AcOEt = 3/1).

Step 2: After the purification, to a solution of the ester (1.0 mmol) in EtOH (10 mL) were added NaOH (2.0 g) and H_2O (5 mL) at rt under air atmosphere. The resulting mixture was stirred at reflux overnight. After completion of the reaction, the mixture was cooled to rt and diluted with water (10 mL) and conc. HCl (10 mL). The resulting solution was extracted with AcOEt (10 mL x 3). The organic layers were washed with brine (10 mL). The solvent was dried over $MgSO_4$ and removed under reduced pressure. The residue was purified by SiO_2 column chromatography (eluent: hexane/AcOEt = 2/1).

Step 3: The next amidation was conducted according to Step 2 of Method B.

***N*,5,5-Triphenylpent-4-enamide (1a)**

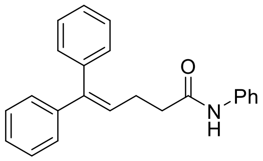

Prepared according to Method A, 2.0 g (60% over 2 steps, 10.0 mmol scale), recrystallized from DCM/hexane, colorless needles, mp. 139–140 ˚C.

^{1}H NMR (400 MHz, $CDCl_3$/TMS) δ (ppm): 7.47 (2H, d, *J* = 7.8 Hz), 7.38–7.18 (12H, m), 7.11–7.04 (2H, m), 6.13 (1H, t, *J* = 7.6 Hz), 2.56 (2H, t, *J* = 7.2 Hz), 2.48 (2H, t, *J* = 7.2 Hz); ^{13}C{^{1}H} NMR (100 MHz, $CDCl_3$/TMS) δ (ppm): 170.4, 143.2, 142.2, 139.6, 137.8, 129.7, 128.9, 128.3, 128.1, 127.23, 127.19, 127.1, 124.2, 119.8, 111.3, 37.6, 25.8; LRMS (EI) *m/z*: 327 (M^+); HRMS (EI-TOF) Calcd. for $C_{23}H_{21}NO$: 327.1623, found: 327.1606; IR (neat): 3317, 3296, 1656, 1601, 1542, 1533, 1490, 1442, 763, 755, 743 cm^{-1}.

5,5-Bis(4-methoxyphenyl)-*N*-phenylpent-4-enamide (1b)

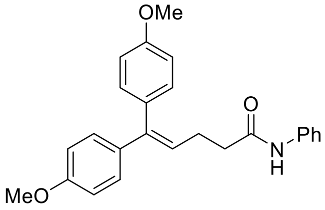

Prepared according to Method A, 1.2 g (61% over 2 steps, 5.0 mmol scale), recrystallized from DCM/hexane, colorless powder, mp. 158–160 ˚C.

^{1}H NMR (600 MHz, $CDCl_3$/TMS) δ (ppm): 7.46 (2H, d, *J* = 7.6 Hz), 7.29 (2H, t, *J* = 7.9 Hz), 7.18–7.13 (3H, m), 7.10–7.06 (3H, m), 6.89 (2H, d, *J* = 8.9 Hz), 6.79 (2H, d, *J* = 8.2 Hz), 5.96 (1H, t, *J* = 7.2 Hz), 3.82 (3H, s), 3.78 (3H, s), 2.55 (2H, q, *J* = 7.4 Hz), 2.45 (2H, t, *J* = 7.4 Hz); ^{13}C{^{1}H} NMR (150 MHz, $CDCl_3$/TMS) δ (ppm): 170.4, 158.9, 158.7, 142.4, 137.8, 135.3, 132.1, 130.9, 129.0, 128.4, 125.3, 124.2, 119.8, 113.7, 113.5, 55.3, 55.2, 37.9, 25.8;LRMS (EI) *m/z*: 387 (M^+); HRMS (EI-TOF) Calcd. for $C_{25}H_{25}NO_3$: 387.1834, found: 387.1820; IR (neat): 3296, 2834, 1649, 1607, 1509, 1439, 1247, 1176, 1031, 837 cm^{-1}.

***N*-Phenyl-5,5-di-*p*-tolylpent-4-enamide (1c)**

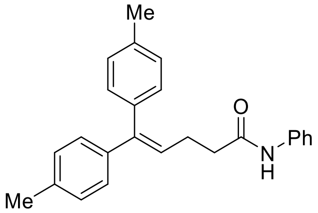

Prepared according to Method B, 230.2 mg (22% over 2 steps, 3.0 mmol scale), recrystallized from DCM/hexane, colorless plates, mp. 119–120 ˚C.
^{1}H NMR (600 MHz, $CDCl_3$/TMS) δ (ppm): 7.46 (2H, d, J = 8.0 Hz), 7.29 (2H, t, J = 7.8 Hz), 7.17 (2H, d, J = 7.5 Hz), 7.11–7.05 (8H, m), 6.04 (1H, t, J = 7.5 Hz), 2.55 (2H, q, J = 7.3 Hz), 2.45 (2H, t, J = 7.3 Hz), 2.37 (3H, s), 2.31 (3H, s); ^{13}C{^{1}H} NMR (150 MHz, $CDCl_3$/TMS) δ (ppm): 170.4, 143.1, 139.7, 137.8, 136.9, 136.81, 136.79, 129.7, 129.00, 128.96, 128.8, 127.2, 126.1, 124.2, 119.8, 37.8, 25.8, 21.2, 21.0; LRMS (EI) *m/z*: 355 (M^+); HRMS (EI-EB) Calcd. for $C_{25}H_{25}NO$: 355.1936, found: 355.1941; IR (neat): 3290, 3017, 2861, 1649, 1600, 1526, 1443, 817 cm^{-1}.

5,5-Bis(4-fluorophenyl)-*N*-phenylpent-4-enamide (1d)

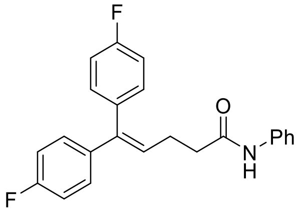

Prepared according to Method A, 1.0 g (57% over 2 steps, 5.0 mmol scale), recrystallized from DCM /hexane, colorless needles, mp. 133–135 ˚C.
^{1}H NMR (400 MHz, $CDCl_3$/TMS) δ (ppm): 7.47 (2H, d, J = 8.3 Hz), 7.30 (2H, t, J = 8.1 Hz), 7.18–7.03 (8H, m), 6.94 (2H, t, J = 8.8 Hz), 6.04 (1H, t, J = 7.1 Hz), 2.55–2.44 (4H, m); ^{13}C{^{1}H} NMR (100 MHz, $CDCl_3$/TMS) δ (ppm): 170.3, 162.1 ($^1J_{C\text{-}F}$ = 245.3 Hz), 162.0 ($^1J_{C\text{-}F}$ = 245.3 Hz), 141.1, 138.3 ($^4J_{C\text{-}F}$ = 3.3 Hz), 137.7, 135.3 ($^4J_{C\text{-}F}$ = 3.3 Hz), 131.3 ($^3J_{C\text{-}F}$ = 8.2 Hz), 128.9, 128.8 ($^3J_{C\text{-}F}$ = 8.2 Hz), 127.5, 124.3, 119.8, 115.3 ($^2J_{C\text{-}F}$ = 20.6 Hz), 115.0 ($^2J_{C\text{-}F}$ = 21.4 Hz), 37.4, 25.7; ^{19}F NMR (565 MHz, $CDCl_3$) δ (ppm): −114.0, −114.7; LRMS(EI) *m/z*: 363 (M^+); HRMS (EI-TOF) Calcd. for $C_{23}H_{19}F_2NO$ (M^+): 363.1435, found: 363.1423; IR (neat): 3286, 1654, 1601, 1506, 1501, 1222, 843, 749, 742 cm^{-1}.

5,5-Bis(4-chlorophenyl)-*N*-phenylpent-4-enamide (1e)

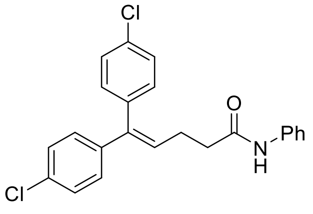

Prepared according to Method A, 1.3 g (65% over 2 steps, 5.0 mmol scale), recrystallized from DCM/hexane, colorless needles, mp. 148–149 ˚C.

^{1}H NMR (600 MHz, DMSO-d_6) δ (ppm): 9.89 (1H, br.s), 7.60–7.52 (2H, m), 7.45 (2H, d, *J* = 6.8 Hz), 7.34–7.33 (2H, m), 7.27 (2H, t, *J* = 7.9 Hz), 7.16–7.14 (4H, m), 7.00 (1H, t, *J* = 7.6 Hz), 6.19 (1H, t, *J* = 7.6 Hz), 2.45–2.43 (2H, m), 2.35–2.34 (2H, m); ^{13}C{^{1}H} NMR (150 MHz, DMSO-d_6) δ (ppm): 170.3, 140.4, 139.2, 139.1, 137.7, 132.1, 131.8, 131.3, 129.8, 128.6, 128.52, 128.48, 128.3, 123.0, 119.0, 36.1, 25.7; LRMS (EI) *m/z*: 395 (M^+); HRMS(EI-TOF) Calcd. for $C_{23}H_{19}{}^{35}Cl_2NO$: 395.0844, found: 395.0816; IR (neat): 3299, 1654, 1600, 1521, 1488, 1442, 1093, 1016, 828, 759, 753 cm^{-1}.

***N*-Phenyl-5,5-bis(4-(trifluoromethyl)phenyl)pent-4-enamide (1f)**

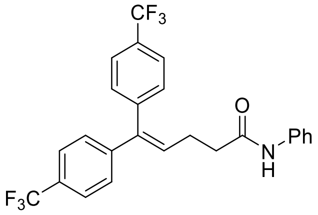

Prepared according to Method A, 665.5 mg (29% over 2 steps, 5.0 mmol scale), recrystallized from DCM/hexane, colorless needles, mp. 153−155 ˚C.

^{1}H NMR (600 MHz, CDCl$_3$/TMS) δ (ppm): 7.64 (2H, d, *J* = 8.2 Hz), 7.51 (2H, d, *J* = 8.9 Hz), 7.47 (2H, d, *J* = 8.2 Hz), 7.32–7.26 (6H, m), 7.11 (2H, t, *J* = 7.2 Hz), 6.26 (1H, t, *J* = 7.2 Hz), 2.56 (2H, q, *J* = 7.1 Hz), 2.50 (2H, t, *J* = 7.1 Hz); ^{13}C{^{1}H, ^{19}F} NMR (150 MHz, CDCl$_3$/TMS) δ (ppm): 169.8, 144.9, 142.7, 140.9, 137.6, 130.6, 130.1, 129.8, 129.4, 129.1, 127.4, 125.6, 125.3, 124.5, 124.11, 124.07, 119.8, 37.1, 25.7; ^{19}F NMR (565 MHz, CDCl$_3$/TMS) δ (ppm): −61.87, −61.89; LRMS (EI) *m/z*: 463 (M^+); HRMS (EI-TOF) Calcd. for $C_{25}H_{19}F_6NO$: 463.1371, found: 463.1399; IR (neat): 3312, 1658, 1600, 1532, 1501, 1324, 1104, 1068, 834, 707 cm^{-1}.

***N*-Phenyl-4-(9*H*-xanthen-9-ylidene)butanamide (1g)**

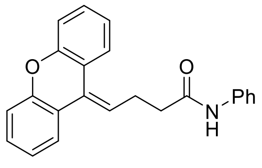

Prepared according to Method C, 142.4 mg (14% over 3 steps, 3.0 mmol scale), recrystallized from DCM/hexane, colorless needles, mp.141−143 ˚C.

^{1}H NMR (600 MHz, CDCl$_3$/TMS) δ (ppm): 7.58 (1H, d, *J* = 7.5 Hz), 7.52 (1H, d, *J* = 7.5 Hz), 7.49 (2H, d, *J* = 8.0 Hz), 7.33–7.29 (3H, m), 7.27–7.24 (1H, m), 7.18–7.09 (6H, m), 5.87 (1H, t, *J* = 7.0 Hz), 3.01 (2H, q, *J* = 7.4 Hz), 2.58 (2H, t, *J* = 7.4 Hz); ^{13}C{^{1}H} NMR (150 MHz, CDCl$_3$/TMS) δ (ppm): 170.2, 152.9, 151.3, 137.7, 129.0, 128.8, 128.3, 127.9, 127.6, 125.4, 124.3, 123.9, 123.8, 123.5, 122.9, 122.2, 119.8, 116.7, 116.5, 38.0, 25.6; LRMS (EI) *m/z*: 341 (M^+);

HRMS (EI-TOF) Calcd. for $C_{23}H_{19}NO_2$: 341.1416, found: 341.1413; IR (neat): 3293, 1648, 1601, 1477, 1451, 1256, 868 cm^{-1}.

4-(5*H*-Dibenzo[*a*,*d*][7]annulen-5-ylidene)-*N*-phenylbutanamide (1h)

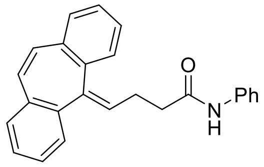

Prepared according to Method A, 607.9 mg (35% over 2 steps, 5.0 mmol scale), recrystallized from DCM/hexane, colorless crystals, mp. 164–167 ˚C.

^{1}H NMR (600 MHz, $CDCl_3$/TMS) δ (ppm): 7.43 (2H, d, J = 7.6 Hz), 7.36–7.24 (10H, m), 7.08 (1H, t, J = 7.6 Hz), 7.01 (1H, br.s), 6.84–6.80 (2H, m), 5.60 (1H, dd, J = 9.0, 5.5 Hz), 2.65–2.61 (1H, m), 2.50–2.46 (1H, m), 2.40–2.35 (2H, m); $^{13}C\{^{1}H\}$ NMR (150 MHz, $CDCl_3$/TMS) δ (ppm): 170.2, 143.0, 142.2, 137.7, 136.9, 134.7, 133.9, 131.7, 131.2, 131.0, 128.9, 128.80, 128.78, 128.7, 128.25, 128.18, 127.6, 127.1, 127.0, 124.2, 119.9, 37.6, 24.7; LRMS (EI) *m/z*: 351 (M^+); HRMS (EI-TOF) Calcd. for $C_{25}H_{21}NO$: 351.1623, found: 351.1605; IR (neat): 3019, 1704, 1487, 1432, 1208, 924, 796 cm^{-1}.

***N*-(4-Methoxyphenyl)-5,5-diphenylpent-4-enamide (1i)**

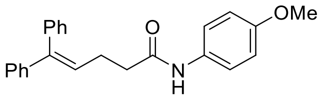

Prepared according to Method A, 1.0 g (58% over 2 steps, 5.0 mmol scale), recrystallized from DCM/hexane, colorless powder, mp. 144–147 ˚C.

^{1}H NMR (600 MHz, $CDCl_3$/TMS) δ (ppm): 7.36–7.34 (4H, m), 7.31–7.28 (2H, m), 7.25–7.19 (5H, m), 7.16 (2H, d, J = 7.5 Hz), 6.81 (2H, d, J = 8.9 Hz), 6.10 (1H, t, J = 7.2 Hz), 3.75 (3H, s), 2.53 (2H, q, J = 7.6 Hz), 2.42 (2H, t, J = 7.6 Hz); $^{13}C\{^{1}H\}$ NMR (150 MHz, $CDCl_3$/TMS) δ (ppm): 170.2, 156.3, 143.1, 142.3, 139.7, 130.9, 129.7, 128.3, 128.1, 127.4, 127.24, 127.18, 127.1, 121.7, 114.1, 55.4, 37.4, 25.8; LRMS (EI) *m/z*: 357 (M^+); HRMS (EI-TOF) Calcd. for $C_{24}H_{23}NO_2$ (M^+): 357.1729, found: 357.1721; IR (neat): 3288, 2835, 1652, 1609, 1510, 1246, 1031, 831 cm^{-1};

5,5-Diphenyl-*N*-(*p*-tolyl)pent-4-enamide (1j)

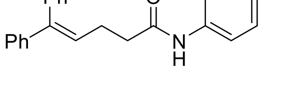

Prepared according to Method A, 1.2 g (69% over 2 steps, 5.0 mmol scale), recrystallized from DCM/hexane, colorless crystals, mp. 136–138 ˚C.

^{1}H NMR (600 MHz, $CDCl_3$/TMS) δ (ppm): 7.37–7.30 (5H, m), 7.26–7.24 (2H, m), 7.22–7.20 (3H, m), 7.17 (2H, d, *J* = 7.0 Hz), 7.12 (1H, br.s), 7.09 (2H, d, *J* = 8.0 Hz), 6.11 (1H, t, *J* = 7.3 Hz), 2.54 (2H, q, *J* = 7.4 Hz), 2.44 (2H, t, *J* = 7.4 Hz), 2.29 (3H, s); ^{13}C{^{1}H} NMR (150 MHz, $CDCl_3$/TMS) δ (ppm): 170.2, 143.2, 142.3, 139.7, 135.2, 133.8, 129.8, 129.4, 128.3, 128.1, 127.3, 127.25, 127.20, 127.1, 119.9, 37.6, 25.8, 20.8; LRMS (EI) *m/z*: 341 (M^+); HRMS (EI-TOF) Calcd. for $C_{24}H_{23}NO$: 341.1780, found: 341.1762; IR (neat): 3293, 1655, 1601, 1525, 1496, 1442, 817, 764 cm^{-1}.

***N*-(4-Isopropylphenyl)-5,5-diphenylpent-4-enamide (1k)**

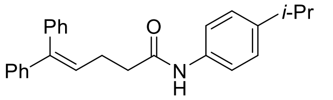

Prepared according to Method A, 873.9 mg (47% over 2 steps, 5.0 mmol scale), recrystallized from DCM/hexane, colorless powder, mp. 105–106 ˚C.

^{1}H NMR (600 MHz, $CDCl_3$/TMS) δ (ppm): 7.38–7.35 (4H, m), 7.31 (1H, t, *J* = 7.6 Hz), 7.26–7.14 (9H, m), 7.09 (1H, br.s), 6.11 (1H, t, *J* = 7.4 Hz), 2.86 (1H, sep, *J* = 6.8 Hz), 2.55 (2H, q, *J* = 7.4 Hz), 2.45 (2H, t, *J* = 7.4 Hz), 1.22 (6H, d, *J* = 6.8 Hz); ^{13}C{^{1}H} NMR (150 MHz, $CDCl_3$/TMS) δ (ppm): 170.2, 145.0, 143.2, 142.3, 139.7, 135.4, 129.8, 128.3, 128.1, 127.31, 127.26, 127.2, 127.1, 126.8, 120.0, 37.6, 33.6, 25.8, 24.0; LRMS (EI) *m/z*: 369 (M^+); HRMS (EI-TOF) Calcd. for $C_{26}H_{27}NO$: 369.2093, found: 369.2082; IR (neat): 3296, 2962, 1652, 1598, 1516, 1256, 831, 773 cm^{-1}.

***N*-(4-Fluorophenyl)-5,5-diphenylpent-4-enamide (1l)**

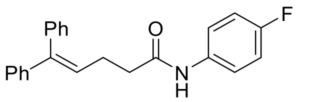

Prepared according to Method A, 1.2 g (70% over 2 steps, 5.0 mmol scale), recrystallized from DCM/hexane, colorless needles, mp. 120–122 ˚C.

^{1}H NMR (600 MHz, $CDCl_3$/TMS) δ (ppm): 7.40 (2H, dd, *J* = 8.9, 4.8 Hz), 7.36 (2H, t, *J* = 7.2 Hz), 7.32–7.29 (1H, m), 7.26–7.19 (6H, m), 7.16 (2H, d, *J* = 6.9 Hz), 6.97 (2H, t, *J* = 8.9 Hz), 6.09 (1H, t, *J* = 7.3 Hz), 2.54 (2H, q, *J* = 7.3 Hz), 2.44 (2H, t, *J* = 7.3 Hz); ^{13}C{^{1}H} NMR (150 MHz, $CDCl_3$/TMS) δ (ppm): 170.5, 159.3 ($^{1}J_{C\text{-}F}$ = 242.0 Hz), 143.3, 142.2, 139.6, 133.81, 133.79, 129.7, 128.3, 128.1, 127.21, 127.16, 127.13, 121.7 ($^{3}J_{C\text{-}F}$ = 7.7 Hz), 115.5 ($^{2}J_{C\text{-}F}$ = 22.3 Hz), 37.4, 25.8; ^{19}F NMR (565 MHz, $CDCl_3$) δ (ppm): −117.4; LRMS (EI) *m/z*: 345 (M^+); HRMS (EI-TOF)

Calcd. for $C_{23}H_{20}FNO$: 345.1529, found: 345.1514; IR (neat): 3303, 1660, 1506, 1407, 1210, 831 cm^{-1}.

***N*-(4-Chlorophenyl)-5,5-diphenylpent-4-enamide** (**1m**)

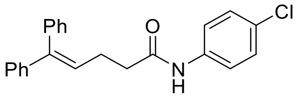

Prepared according to Method A, 631.1 mg (35% over 2 steps, 5.0 mmol scale), recrystallized from DCM/hexane, colorless needles, mp. 135−138 ˚C.

^{1}H NMR (600 MHz, $CDCl_3$/TMS) δ (ppm): 7.41 (2H, d, *J* = 8.3 Hz), 7.38–7.35 (2H, m), 7.33–7.30 (1H, m), 7.27–7.16 (9H, m), 7.12 (1H, br.s), 6.10 (1H, t, *J* = 7.4 Hz), 2.55 (2H, q, *J* = 7.4 Hz), 2.46 (2H, t, *J* = 7.4 Hz); ^{13}C{^{1}H} NMR (150 MHz, $CDCl_3$/TMS) δ (ppm): 170.3, 143.4, 142.2, 139.6, 136.3, 129.7, 129.2, 129.0, 128.4, 128.2, 127.3, 127.24, 127.22, 127.0, 121.0, 37.6, 25.7; LRMS (EI) *m/z*: 361 (M^+); HRMS (EI-TOF) Calcd. for $C_{23}H_{20}{}^{35}ClNO$ (M^+): 361.1233, found: 361.1204; IR (neat): 3310, 1660, 1594, 1515, 1491, 1397, 1091, 821 cm^{-1}.

***N*-(4-Bromophenyl)-5,5-diphenylpent-4-enamide** (**1n**)

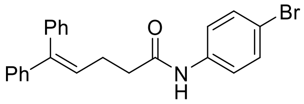

Prepared according to Method A, 1.0 g (49% over 2 steps, 5.0 mmol scale), recrystallized from DCM/hexane, yellow plates, mp. 156−158 ˚C.

^{1}H NMR (600 MHz, $CDCl_3$/TMS) δ (ppm): 7.40–7.35 (6H, m), 7.32–7.30 (1H, m), 7.26–7.19 (6H, m), 7.16 (2H, d, *J* = 7.6 Hz), 6.09 (1H, t, *J* = 7.3 Hz), 2.54 (2H, q, *J* = 7.3 Hz), 2.45 (2H, t, *J* = 7.3 Hz); ^{13}C{^{1}H} NMR (150 MHz, $CDCl_3$/TMS) δ (ppm): 170.4, 143.43, 143.42, 142.1, 139.6, 136.8, 131.9, 129.7, 128.4, 128.1, 127.3, 127.2, 127.0, 121.3, 116.8, 37.6, 25.7; LRMS (EI) *m/z*: 405 (M^+); HRMS (EI-TOF) Calcd. for $C_{23}H_{20}{}^{79}BrNO$: 405.0728, found: 405.0701; IR (neat): 3291, 1659, 1598, 1521, 1490, 1396, 1303, 1070 cm^{-1}.

5,5-Diphenyl-*N*-(4-(trifluoromethyl)phenyl)pent-4-enamide (**1o**)

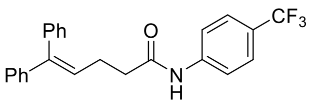

Prepared according to Method B, 1.1 g (58% over 2 steps, 5.0 mmol scale), recrystallized from DCM/hexane, colorless needles, mp. 124−126 ˚C.

^{1}H NMR (600 MHz, $CDCl_3$/TMS) δ (ppm): 7.59 (2H, d, *J* = 8.9 Hz), 7.55 (2H, d, *J* = 8.3 Hz), 7.37 (2H, t, *J* = 7.6 Hz), 7.33–7.31 (1H, m), 7.27–7.20 (6H, m), 7.18–7.16 (2H, m), 6.11 (1H, t, *J* = 7.1 Hz), 2.56 (2H, q, *J* = 7.1 Hz), 2.49 (2H, t, *J* = 7.1 Hz); ^{13}C{^{1}H, ^{19}F} NMR (150 MHz, $CDCl_3$/TMS) δ (ppm): 170.7, 143.6, 142.1, 140.8, 139.6, 129.7, 128.4, 128.2, 127.3, 127.25, 127.22, 126.8, 126.2, 126.0, 124.0, 119.3, 37.7, 25.6; ^{19}F NMR (565 MHz, $CDCl_3$/TMS) δ (ppm): –61.5; LRMS (EI) *m/z*: 395 (M^+); HRMS (EI-TOF) Calcd. for $C_{24}H_{20}F_3NO$ (M^+): 395.1497, found: 395.1490; IR (neat): 3333, 1669, 1601, 1522, 1326, 1112, 1067, 1017, 833 cm^{-1}.

_N_-(4-Cyanophenyl)-5,5-diphenylpent-4-enamide (1p)

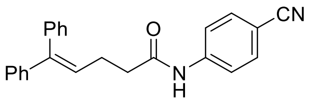

Prepared according to Method B, 755.5 mg (42% over 2 steps, 5.0 mmol scale), recrystallized from DCM/hexane, colorless powder, mp. 142–143 ˚C.

^{1}H NMR (600 MHz, $CDCl_3$/TMS) δ (ppm): 7.61–7.58 (4H, m), 7.37 (2H, t, *J* = 7.2 Hz), 7.34–7.31 (1H, m), 7.27–7.23 (4H, m), 7.20 (2H, d, *J* = 8.3 Hz), 7.17–7.16 (2H, m), 6.10 (1H, t, *J* = 7.2 Hz), 2.56 (2H, q, *J* = 7.2 Hz), 2.51 (2H, t, *J* = 7.2 Hz); ^{13}C{^{1}H} NMR (150 MHz, $CDCl_3$/TMS) δ (ppm): 170.6, 143.7, 142.0, 141.8, 139.5, 133.3, 129.7, 128.4, 128.2, 127.34, 127.32, 127.2, 126.6, 119.4, 118.8, 107.1, 37.8, 25.5; LRMS (EI) *m/z*: 352 (M^+); HRMS (EI-TOF) Calcd. for $C_{24}H_{20}N_2O$: 352.1576, found: 352.1583; IR (neat): 3106, 2220, 1665, 1594, 1498, 1408, 1173 cm^{-1}.

Methyl 4-(5,5-diphenylpent-4-enamido)benzoate (1q)

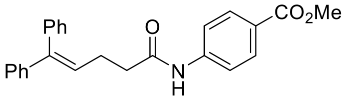

Prepared according to Method A, 927.1 mg (48% over 2 steps, 5.0 mmol scale), recrystallized from DCM/hexane, colorless needles, mp. 120–123 ˚C.

^{1}H NMR (600 MHz, DMSO-*d*$_6$) δ (ppm): 10.3 (1H, s), 7.89 (2H, d, *J* = 9.0 Hz), 7.70 (2H, d, *J* = 9.0 Hz), 7.39 (2H, t, *J* = 7.5 Hz), 7.33 (1H, t, *J* = 7.5 Hz), 7.27 (2H, t, *J* = 7.3 Hz), 7.21 (1H, t, *J* = 7.3 Hz), 7.15–7.12 (4H, m), 6.12 (1H, t, *J* = 7.4 Hz), 3.80 (3H, s), 2.50–2.48 (2H, m), 2.36 (2H, q, *J* = 7.4 Hz); ^{13}C{^{1}H} NMR (150 MHz, DMSO-*d*$_6$) δ (ppm): 171.1, 165.8, 143.5, 141.9, 141.6, 139.4, 130.2, 129.4, 128.3, 128.2, 128.0, 127.1, 127.0, 126.8, 123.7, 118.3, 51.8, 36.5, 25.5; LRMS (EI) *m/z*: 385 (M^+); HRMS (EI-TOF) Calcd. for $C_{25}H_{23}NO$: 385.1678, found: 385.1706; IR (neat): 3324, 2952, 1715, 1676, 1590, 1517, 1406, 1310, 1281, 1109, 856, 767 cm^{-1}.

***N*-(3-Bromophenyl)-5,5-diphenylpent-4-enamide (1r)**

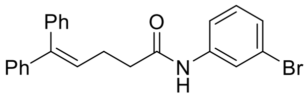

Prepared according to Method B, 1.1 g (53% over 2 steps, 5.0 mmol scale), recrystallized from AcOEt/hexane, colorless plates, mp. 82–83 ˚C.

^{1}H NMR (600 MHz, $CDCl_3$/TMS) δ (ppm): 7.72 (1H, s), 7.39–7.37 (3H, m), 7.32 (1H, tt, *J* = 7.3, 1.7 Hz), 7.27–7.14 (9H, m), 7.08 (1H, br.s), 6.10 (1H, t, *J* = 7.3 Hz), 2.55 (2H, q, *J* = 7.3 Hz), 2.46 (2H, t, *J* = 7.3 Hz); ^{13}C{^{1}H} NMR (150 MHz, $CDCl_3$/TMS) δ (ppm): 170.5, 143.4, 142.1, 139.6, 139.0, 130.2, 129.7, 128.3, 128.1, 128.0, 127.3, 127.23, 127.19, 126.9, 122.7, 122.5, 118.2, 37.6, 25.6; LRMS (EI) *m/z*: 405 (M^+); HRMS (EI-EB) Calcd. for $C_{23}H_{20}{}^{79}BrNO$: 405.0728, found: 405.0754; IR (neat): 3309, 1652, 1583, 1516, 1417, 874, 783 cm^{-1}.

***N*-(Benzo[*d*]thiazol-2-yl)-5,5-diphenylpent-4-enamide (1s)**

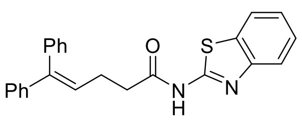

Prepared according to Method B, 784.9 mg (41% over 2 steps, 5.0 mmol scale), recrystallized from DCM/hexane, colorless plates, mp. 173–175 ˚C.

^{1}H NMR (600 MHz, $CDCl_3$/TMS) δ (ppm): 11.2 (1H, br.s), 7.85 (1H, d, *J* = 8.0 Hz), 7.75 (1H, d, *J* = 8.0 Hz), 7.44 (1H, t, *J* = 7.5 Hz), 7.35–7.19 (7H, m), 7.13 (2H, d, *J* = 8.0 Hz), 7.06 (2H, d, *J* = 7.5 Hz), 5.98 (1H, t, *J* = 7.0 Hz), 2.59–2.51 (4H, m); ^{13}C{^{1}H} NMR (150 MHz, $CDCl_3$/TMS) δ (ppm): 171.0, 159.8, 147.7, 143.5, 142.1, 139.4, 131.9, 129.6, 128.3, 128.1, 127.21, 127.18, 127.15, 126.4, 126.3, 124.0, 121.7, 120.3, 36.5, 25.2; LRMS (EI) *m/z*: 384 (M^+); HRMS (EI-TOF) Calcd. for $C_{24}H_{20}N_2OS$: 384.1296, found: 384.1291; IR (neat): 3189, 3060, 1700, 1598, 1551, 1497, 1443, 1270, 1167, 768, 752 cm^{-1}.

4. General procedure for the synthesis of 5-alkylidene-pyrrolin-2-ones

In a glove box, an amide **1** (0.20 mmol), CuF_2 (2.0 mg, 0.020 mmol), 4-*tert*-butylpyridine (29.3 μL, 0.020 mmol), *t*BuOO*t*Bu (147.0 μL, 0.80 mmol), and 1,2-dichloroethane (2.5 mL) were added to a sealed tube. The mixture was stirred at 120 °C for 18 h. The reaction was diluted with water (10 mL) and extracted with chloroform (10 mL × 3). The organic layers were washed with brine (10 mL) and dried over $MgSO_4$. The solvent was removed under a reduced pressure and the residue was purified by SiO_2 column chromatography.

Table S1. Effect of reaction parameter for the synthesis of **2a**.

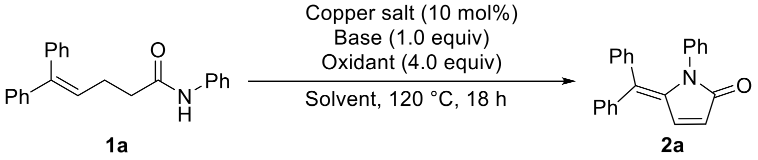

Entry	Copper salt	Base	Oxidant	Solvent	2a (%) [a]
1	CuF_2	4-*tert*-Butylpyridine	*t*BuOO*t*Bu	1,2-DCE	76 (85)
2	CuCl	4-*tert*-Butylpyridine	*t*BuOO*t*Bu	1,2-DCE	75
3	$CuCl_2$	4-*tert*-Butylpyridine	*t*BuOO*t*Bu	1,2-DCE	46
4	$Cu(OAc)_2$	4-*tert*-Butylpyridine	*t*BuOO*t*Bu	1,2-DCE	39
5	CuF_2	Pyridine	*t*BuOO*t*Bu	1,2-DCE	66
6	CuF_2	DMAP	*t*BuOO*t*Bu	1,2-DCE	44
7	CuF_2	1,10-Phen	*t*BuOO*t*Bu	1,2-DCE	21
8	CuF_2	None	*t*BuOO*t*Bu	1,2-DCE	29
9	CuF_2	4-*tert*-Butylpyridine	*t*BuOOH	1,2-DCE	2
10	CuF_2	4-*tert*-Butylpyridine	*t*BuOOAc	1,2-DCE	0
11	CuF_2	4-*tert*-Butylpyridine	PIDA	1,2-DCE	35
12	CuF_2	4-*tert*-Butylpyridine	MnO_2	1,2-DCE	5
13	CuF_2	4-*tert*-Butylpyridine	*t*BuOO*t*Bu	DCM	0
14	CuF_2	4-*tert*-Butylpyridine	*t*BuOO*t*Bu	Toluene	44
15	CuF_2	4-*tert*-Butylpyridine	*t*BuOO*t*Bu	$PhCF_3$	0
16 [b]	CuF_2	4-*tert*-Butylpyridine	*t*BuOO*t*Bu	1,2-DCE	73

[a] Determined by ^{1}H-NMR using 1,1,2-trichloroethane as an internal standard. Isolated yield in parentheses. [b] Reaction was conducted at 100 °C.

5-(Diphenylmethylene)-1-phenyl-1,5-dihydro-2*H*-pyrrol-2-one (2a)

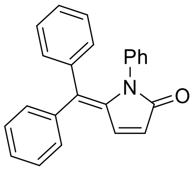

Obtained as yellow needles in 85% (55.0 mg, 0.20 mmol scale), 87% (286.6 mg, 1.0 mmol scale), recrystallized from DCM/hexane, mp. 160–163 ˚C.

^{1}H NMR (400 MHz, $CDCl_3$/TMS) δ (ppm): 7.38–7.36 (3H, m), 7.26–7.23 (2H, m), 7.21 (1H, d, *J* = 5.8 Hz), 7.00–6.83 (10H, m), 6.28 (1H, d, *J* = 5.4 Hz); $^{13}C\{^1H\}$ NMR (100 MHz, $CDCl_3$/TMS) δ (ppm): 171.8, 140.5, 140.3, 138.1, 137.9, 135.8, 131.6, 130.94, 130.89, 128.4, 128.0, 127.9, 127.7, 127.2, 127.1, 126.0, 121.7; LRMS (EI) *m/z*: 323 (M^+); HRMS (EI-TOF) Calcd. for $C_{23}H_{17}NO$: 323.1310, found: 323.1286; IR (neat): 3052, 1691, 1683, 1498, 1443, 1370, 1213, 1203, 1163, 1073, 968, 801, 774, 765, 756 cm^{-1}.

5-(Bis(4-methoxyphenyl)methylene)-1-phenyl-1,5-dihydro-2*H*-pyrrol-2-one (**2b**)

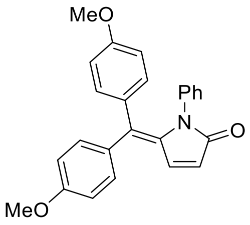

Obtained as red crystals in 80% (61.5 mg), recrystallized from DCM/hexane, mp. 177−178 ˚C.
^{1}H NMR (600 MHz, $CDCl_3$/TMS) δ (ppm): 7.19–7.17 (3H, m), 7.01 (2H, t, *J* = 7.6 Hz), 6.97–6.94 (3H, m), 6.90 (2H, d, *J* = 8.9 Hz), 6.77 (2H, d, *J* = 8.9 Hz), 6.42 (2H, d, *J* = 8.9 Hz), 6.23 (1H, d, *J* = 5.5 Hz), 3.85 (3H, s), 3.66 (3H, s); ^{13}C{^{1}H} NMR (150 MHz, $CDCl_3$/TMS) δ (ppm): 171.9, 160.1, 159.3, 140.3, 136.8, 136.1, 133.1, 132.5, 131.0, 130.6, 127.8, 127.0, 125.73, 125.72, 120.6, 113.5, 112.7, 55.3, 55.2; LRMS (EI) *m/z*: 383 (M^+); HRMS (EI-TOF) Calcd. for $C_{25}H_{21}NO_3$: 383.1521, found: 383.1539; IR (neat): 1675, 1604, 1508, 1498, 1252, 1179, 1028, 969, 831 cm^{-1}.

5-(Di-*p*-tolylmethylene)-1-phenyl-1,5-dihydro-2*H*-pyrrol-2-one (**2c**)

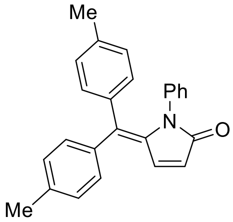

Obtained as colorless needles in 60% (42.4 mg), recrystallized from DCM/hexane, mp. 161−162 ˚C.
^{1}H NMR (600 MHz, DMSO-d_6) δ (ppm): 7.21 (2H, d, *J* = 8.2 Hz), 7.18 (1H, d, *J* = 5.9 Hz), 7.11 (2H, d, *J* = 8.3 Hz), 6.99 (2H, t, *J* = 7.2 Hz), 6.94–6.91 (3H, m), 6.70 (2H, d, *J* = 8.2 Hz), 6.67 (2H, d, *J* = 8.2 Hz), 6.30 (1H, d, *J* = 5.9 Hz), 2.34 (3H, s), 2.07 (3H, s); ^{13}C{^{1}H} NMR (150 MHz, DMSO-d_6) δ (ppm): 171.0, 140.6, 138.1, 137.5, 137.3, 137.1, 136.0, 135.1, 131.4, 130.6, 130.3, 128.8, 127.7, 127.6, 127.2, 125.5, 121.0, 20.8, 20.7; LRMS (EI) *m/z*: 351 (M^+); HRMS (EI-TOF) Calcd. for $C_{25}H_{21}NO$: 351.1623, found: 351.1606; IR (neat): 1688, 1497, 1371, 1300, 1209, 1182, 969, 823, 802 cm^{-1}.

5-(Bis(4-fluorophenyl)methylene)-1-phenyl-1,5-dihydro-2*H*-pyrrol-2-one (**2d**)

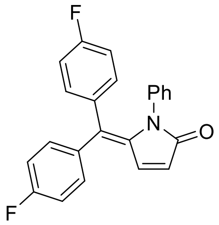

Obtained as yellow crystals in 82% (58.9 mg), recrystallized from DCM/hexane, mp. 153−155 ˚C. ^{1}H NMR (600 MHz, $CDCl_3$/TMS) δ (ppm): 7.21 (2H, dd, J = 8.6, 5.2 Hz), 7.17 (1H, d, J = 5.5 Hz), 7.08 (2H, t, J = 8.6 Hz), 7.03 (2H, t, J = 7.6 Hz), 6.98 (1H, t, J = 7.6 Hz), 6.95–6.93 (2H, m), 6.80 (2H, dd, J = 8.9, 5.5 Hz), 6.59 (2H, t, J = 8.9 Hz), 6.29 (1H, d, J = 5.5 Hz); ^{13}C{^{1}H} NMR (150 MHz, $CDCl_3$/TMS) δ (ppm): 171.6, 163.0 (d, $J_{C\text{-}F}$ = 249.3 Hz), 162.1 (d, $J_{C\text{-}F}$ = 249.3 Hz), 139.8, 138.2, 136.4 (d, $J_{C\text{-}F}$ = 2.9 Hz), 135.7, 133.9 (d, $J_{C\text{-}F}$ = 2.9 Hz), 133.2 (d, $J_{C\text{-}F}$ = 8.6 Hz), 132.6 (d, $J_{C\text{-}F}$ = 8.6 Hz), 128.2, 128.1, 127.1, 126.3, 122.1, 115.3 (d, $J_{C\text{-}F}$ = 21.5 Hz), 114.4 (d, $J_{C\text{-}F}$ = 22.9 Hz); ^{19}F NMR (565 MHz, $CDCl_3$) δ (ppm): −111.7, −111.9; LRMS (EI) *m/z*: 359 (M^+); HRMS (EI-TOF) Calcd. for $C_{23}H_{15}F_2NO$: 359.1122, found: 359.1110; IR (neat): 3039, 1688, 1594, 1505, 1497, 1366, 1305, 1231, 1156, 1101, 969, 844 cm^{-1}.

5-(Bis(4-chlorophenyl)methylene)-1-phenyl-1,5-dihydro-2*H*-pyrrol-2-one (2e)

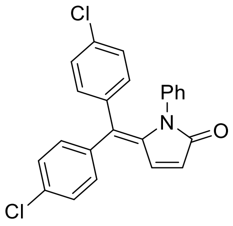

Obtained as yellow crystals in 70% (54.6 mg), recrystallized from DCM/hexane, mp. 206−208 ˚C. ^{1}H NMR (600 MHz, $CDCl_3$/TMS) δ (ppm): 7.36 (2H, d, J = 8.2 Hz), 7.18–7.16 (3H, m), 7.05–6.99 (3H, m), 6.93–6.92 (2H, m), 6.86 (2H, d, J = 8.3 Hz), 6.74 (2H, d, J = 8.9 Hz), 6.31 (1H, d, J = 5.5 Hz); ^{13}C{^{1}H} NMR (150 MHz, $CDCl_3$/TMS) δ (ppm): 171.5, 139.6, 138.7, 138.5, 136.0, 135.5, 134.9, 133.9, 132.7, 132.0, 128.5, 128.2, 127.6, 127.5, 127.1, 126.3, 122.4; LRMS (EI) *m/z*: 391 (M^+); HRMS (EI-TOF) Calcd. for $C_{23}H_{15}{}^{35}Cl_2NO$: 391.0531, found: 391.0503; IR (neat): 3063, 1689, 1595, 1498, 1489, 1216, 1087, 1011, 806 cm^{-1}.

5-(Bis(4-(trifluoromethyl)phenyl)methylene)-1-phenyl-1,5-dihydro-2*H*-pyrrol-2-one (2f)

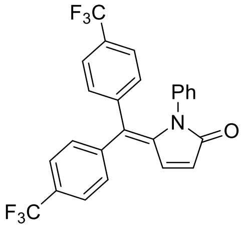

Obtained as yellow crystals in 63% (58.3 mg), recrystallized from DCM/hexane, mp. 168−170 ˚C. ^{1}H NMR (600 MHz, $CDCl_3$/TMS) δ (ppm): 7.66 (2H, d, J = 8.2 Hz), 7.37 (2H, d, J = 8.2 Hz), 7.21 (1H, d, J = 6.2 Hz), 7.14 (2H, d, J = 8.2 Hz), 7.02–6.95 (3H, m), 6.93–6.90 (4H, m), 6.37 (1H, d, J = 5.5 Hz); ^{13}C{^{1}H, ^{19}F} NMR (150 MHz, $CDCl_3$/TMS) δ (ppm): 171.3, 143.4, 140.9, 140.0, 139.4, 135.3, 131.8, 130.9, 130.7, 129.8, 128.3, 127.3, 126.7, 126.4, 125.4, 124.3, 123.9,

123.6, 123.4; ^{19}F NMR (565 MHz, $CDCl_3$) δ (ppm): –62.1, –62.5; LRMS (EI) *m/z*: 459 (M^+); HRMS (EI-TOF) Calcd. for $C_{25}H_{15}F_6NO$: 459.1058, found: 459.1048; IR (neat): 1696, 1612, 1322, 1155, 1121, 1065, 810 cm^{-1}.

1-Phenyl-5-(9*H*-xanthen-9-ylidene)-1,5-dihydro-2*H*-pyrrol-2-one (2g)

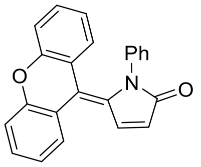

Obtained as yellow crystals in 25% (16.4 mg), recrystallized from DCM/hexane, mp. 180–183 ˚C. ^{1}H NMR (600 MHz, $CDCl_3$/TMS) δ (ppm): 8.02 (1H, d, *J* = 6.0 Hz), 7.62 (1H, dd, *J* = 7.5, 1.5 Hz), 7.40–7.37 (1H, m), 7.30–7.25 (2H, m), 7.07–7.02 (4H, m), 6.99 (1H, td, *J* = 7.7, 1.5 Hz), 6.92 (2H, dd, *J* = 8.0, 1.5 Hz), 6.70 (1H, dd, *J* = 7.5, 1.5 Hz), 6.40 (1H, d, *J* = 6.0 Hz), 6.38–6.35 (1H, m); $^{13}C\{^1H\}$ NMR (150 MHz, $CDCl_3$/TMS) δ (ppm): 171.7, 154.2, 153.2, 138.8, 136.5, 134.4, 128.9, 128.7, 128.6, 128.1, 127.5, 126.6, 126.3, 124.2, 123.8, 122.5, 122.0, 121.6, 116.9, 116.3, 115.2; LRMS (EI) *m/z*: 337 (M^+); HRMS (EI-TOF) Calcd. for $C_{23}H_{15}NO_2$: 337.1103, found: 337.1096; IR (neat): 1684, 1593, 1495, 1447, 1199, 966, 872 cm^{-1}.

5-(5*H*-Dibenzo[*a*,*d*][7]annulen-5-ylidene)-1-phenyl-1,5-dihydro-2*H*-pyrrol-2-one (2h)

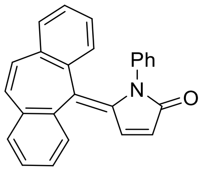

Obtained as yellow crystals in 78% (54.0 mg), recrystallized from DCM/hexane, mp. 235–236 ˚C. ^{1}H NMR (600 MHz, $CDCl_3$/TMS) δ (ppm): 7.40–7.38 (1H, m), 7.35–7.32 (3H, m), 7.28–7.18 (3H, m), 7.08 (1H, d, *J* = 8.3 Hz), 6.99–6.89 (4H, m), 6.70–6.65 (3H, m), 6.29–6.24 (2H, m); $^{13}C\{^1H\}$ NMR (150 MHz, $CDCl_3$/TMS) δ (ppm): 171.3, 138.4, 136.8, 136.3, 135.7, 135.3, 134.8, 133.5, 131.2, 130.9, 129.3, 128.5, 128.1, 128.0, 127.8, 127.54, 127.49, 127.4, 127.3, 126.9, 126.6, 126.3, 122.6; LRMS (EI) *m/z*: 347 (M^+); HRMS (EI-TOF) Calcd. for $C_{25}H_{17}NO$: 347.1310, found: 347.1286; IR (neat): 1690, 1496, 1371, 1210, 1167, 786 cm^{-1}.

5-(Diphenylmethylene)-1-(4-methoxyphenyl)-1,5-dihydro-2*H*-pyrrol-2-one (2i)

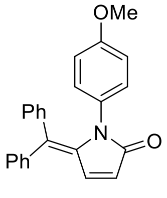

Obtained as yellow crystals in 77% (54.1 mg), recrystallized from DCM/hexane, mp. 133−134 ˚C. ^{1}H NMR (600 MHz, $CDCl_3$/TMS) δ (ppm): 7.37–7.35 (3H, m), 7.23 (2H, dd, *J* = 7.9, 1.7 Hz), 7.18 (1H, d, *J* = 6.2 Hz), 6.99–6.96 (1H, m), 6.91 (2H, t, *J* = 7.6 Hz), 6.86–6.82 (4H, m), 6.53–6.51 (2H, m), 6.27 (1H, d, *J* = 6.2 Hz), 3.66 (3H, s); $^{13}C\{^1H\}$ NMR (150 MHz, $CDCl_3$/TMS) δ (ppm): 172.0, 157.6, 140.6, 139.9, 138.3, 137.9, 131.6, 130.9, 130.6, 128.8, 128.33, 128.28, 128.0, 127.6, 127.2, 121.7, 113.4, 55.3; LRMS (EI) *m/z*: 353 (M^+); HRMS (EI-TOF) Calcd. for $C_{24}H_{19}NO_2$: 353.1416, found: 353.1410; IR (neat): 3052, 1690, 1613, 1512, 1443, 1248, 1159, 1026, 830, 806 cm^{-1}.

5-(Diphenylmethylene)-1-(*p*-tolyl)-1,5-dihydro-2*H*-pyrrol-2-one (2j)

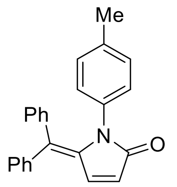

Obtained as yellow crystals in 81% (54.4 mg), recrystallized from DCM/hexane, mp. 103−105 ˚C. ^{1}H NMR (600 MHz, $CDCl_3$/TMS) δ (ppm): 7.37–7.35 (3H, m), 7.24 (2H, dd, *J* = 7.9, 1.7 Hz), 7.18 (1H, d, *J* = 6.2 Hz), 6.95 (1H, t, *J* = 7.5 Hz), 6.88 (2H, t, *J* = 7.5 Hz), 6.83–6.81 (4H, m), 6.77 (2H, d, *J* = 8.2 Hz), 6.26 (1H, d, *J* = 6.2 Hz), 2.14 (3H, s); $^{13}C\{^1H\}$ NMR (150 MHz, $CDCl_3$/TMS) δ (ppm): 171.9, 140.7, 140.0, 138.3, 138.0, 135.7, 133.2, 131.6, 130.9, 130.7, 128.5, 128.4, 128.0, 127.4, 127.1, 127.0, 121.8, 20.8; LRMS (EI) *m/z*: 337 (M^+); HRMS (EI-TOF) Calcd. for $C_{24}H_{19}NO$: 337.1467, found: 337.1441; IR (neat): 3030, 1690, 1593, 1515, 1489, 1446, 1374, 1218, 1162, 971, 824 cm^{-1}.

5-(Diphenylmethylene)-1-(4-isopropylphenyl)-1,5-dihydro-2*H*-pyrrol-2-one (2k)

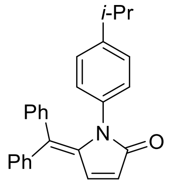

Obtained as orange crystals in 81% (58.9 mg), recrystallized from DCM/hexane, mp. 121−124 ˚C. ^{1}H NMR (600 MHz, $CDCl_3$/TMS) δ (ppm): 7.37–7.34 (3H, m), 7.25–7.23 (2H, m), 7.19 (1H, d, *J* = 5.5 Hz), 6.91 (1H, t, *J* = 7.2 Hz), 6.86–6.78 (8H, m), 6.27 (1H, d, *J* = 6.2 Hz), 2.69 (1H, sep, *J* = 6.9 Hz), 1.09 (6H, d, *J* = 6.9 Hz); $^{13}C\{^1H\}$ NMR (150 MHz, $CDCl_3$/TMS) δ (ppm): 171.8, 146.7, 140.7, 139.9, 138.2, 137.9, 133.3, 131.6, 130.8, 130.7, 128.3, 128.0, 127.5, 127.1, 127.0, 125.9, 121.7, 33.6, 23.8; LRMS (EI) *m/z*: 365 (M^+); HRMS (EI-TOF) Calcd. for $C_{26}H_{23}NO$: 365.1780, found: 365.1752; IR (neat): 2958, 2863, 1692, 1371, 1155, 805 cm^{-1}.

5-(Diphenylmethylene)-1-(4-fluorophenyl)-1,5-dihydro-2*H*-pyrrol-2-one (2l)

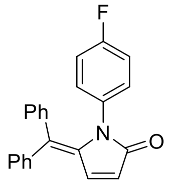

Obtained as yellow crystals in 78% (53.3 mg), recrystallized from DCM/hexane, mp. 153−155 °C. ^{1}H NMR (600 MHz, $CDCl_3$/TMS) δ (ppm): 7.39–7.36 (3H, m), 7.25–7.21 (3H, m), 7.01 (1H, t, *J* = 7.2 Hz), 6.94–6.90 (4H, m), 6.83 (2H, d, *J* = 6.9 Hz), 6.68 (2H, t, *J* = 8.6 Hz), 6.27 (1H, d, *J* = 6.2 Hz); ^{13}C{^{1}H, ^{19}F} NMR (150 MHz, $CDCl_3$/TMS) δ (ppm): 171.9, 160.6, 140.4, 140.3, 138.1, 137.9, 131.9, 131.7, 131.03, 130.95, 128.8, 128.6, 128.2, 128.0, 127.4, 121.7, 114.8; ^{19}F NMR (565 MHz, $CDCl_3$) δ (ppm): −115.4; LRMS (EI) *m/z*: 341 (M^+); HRMS (EI-TOF) Calcd. for $C_{23}H_{16}FNO$: 341.1216, found: 341.1239; IR (neat): 1693, 1600, 1506, 1490, 1382, 1216, 974, 838, 808, 738 cm^{-1}.

1-(4-Chlorophenyl)-5-(diphenylmethylene)-1,5-dihydro-2*H*-pyrrol-2-one (2m)

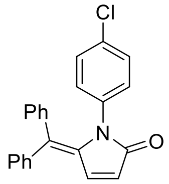

Obtained as yellow crystals in 88% (63.2 mg), recrystallized from DCM/hexane, mp. 128−130 °C. ^{1}H NMR (600 MHz, $CDCl_3$/TMS) δ (ppm): 7.39–7.36 (3H, m), 7.25–7.22 (3H, m), 7.03 (1H, t, *J* = 7.2 Hz), 6.95–6.92 (4H, m), 6.89–6.87 (2H, m), 6.83 (2H, d, *J* = 6.9 Hz), 6.27 (1H, d, *J* = 6.2 Hz); ^{13}C{^{1}H} NMR (150 MHz, $CDCl_3$/TMS) δ (ppm): 171.5, 140.4, 140.2, 137.8, 137.7, 134.5, 131.62, 131.57, 131.2, 130.9, 128.6, 128.2, 128.1, 127.99, 127.97, 127.4, 121.6; LRMS (EI) *m/z*: 357 (M^+); HRMS (EI-TOF) Calcd. for $C_{23}H_{16}{}^{35}ClNO$: 357.0920, found: 357.0910; IR (neat): 1688, 1593, 1554, 1493, 1446, 1374, 1203, 1089, 971, 833, 798 cm^{-1}.

1-(4-Bromophenyl)-5-(diphenylmethylene)-1,5-dihydro-2*H*-pyrrol-2-one (2n)

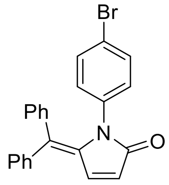

Obtained as yellow crystals in 89% (71.4 mg), recrystallized from DCM/hexane, mp. 149−151 °C. ^{1}H NMR (600 MHz, $CDCl_3$/TMS) δ (ppm): 7.39–7.36 (3H, m), 7.25–7.22 (3H, m), 7.10 (2H, d, *J* = 8.2 Hz), 7.04 (1H, t, *J* = 7.6 Hz), 6.94 (2H, t, *J* = 7.9 Hz), 6.83–6.81 (4H, m), 6.27 (1H, d, *J* = 5.5 Hz); ^{13}C{^{1}H} NMR (150 MHz, $CDCl_3$/TMS) δ (ppm): 171.5, 140.5, 140.2, 137.8, 137.6,

135.0, 131.6, 131.2, 130.93, 130.89, 128.61, 128.58, 128.1, 128.0, 127.4, 121.7, 119.5; LRMS (EI) *m/z*: 401 (M^+); HRMS (EI-TOF) Calcd. for $C_{23}H_{16}{}^{79}BrNO$: 401.0415, found: 401.0427; IR (neat): 3068, 1684, 1489, 1162, 1068, 1015, 831, 798 cm^{-1}.

5-(Diphenylmethylene)-1-(4-(trifluoromethyl)phenyl)-1,5-dihydro-2*H*-pyrrol-2-one (2o)

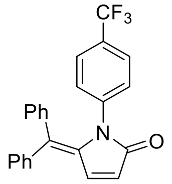

Obtained as colorless needles in 46% (35.8 mg), recrystallized from DCM/hexane, mp. 136−137 ˚C.

^{1}H NMR (600 MHz, $CDCl_3$/TMS) δ (ppm): 7.41−7.37 (3H, m), 7.27−7.26 (3H, m), 7.23 (2H, d, *J* = 8.9 Hz), 7.06 (2H, d, *J* = 8.2 Hz), 6.97 (1H, t, *J* = 7.2 Hz), 6.90 (2H, t, *J* = 7.6 Hz), 6.82 (2H, d, *J* = 7.6 Hz), 6.30 (1H, d, *J* = 5.5 Hz); ^{13}C{^{1}H, ^{19}F} NMR (150 MHz, $CDCl_3$/TMS) δ (ppm): 171.3, 140.8, 140.1, 139.1, 137.8, 137.5, 131.7, 131.5, 130.8, 128.8, 128.24, 128.19, 127.9, 127.5, 127.2, 124.9, 123.8, 121.7; ^{19}F NMR (565 MHz, $CDCl_3$) δ (ppm): −62.1; LRMS (EI) *m/z*: 391 (M^+); HRMS (EI-TOF) Calcd. for $C_{24}H_{16}F_3NO$ (M^+): 391.1184, found: 391.1174; IR (neat): 1691, 1379, 1322, 1112, 1063, 975, 853, 800 cm^{-1}.

4-(2-(Diphenylmethylene)-5-oxo-2,5-dihydro-1*H*-pyrrol-1-yl)benzonitrile (2p)

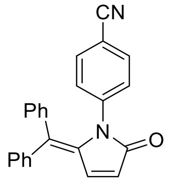

Obtained as yellow crystals in 73% (51.0 mg), recrystallized from DCM/hexane, mp. 178−179 ˚C.
^{1}H NMR (600 MHz, $CDCl_3$/TMS) δ (ppm): 7.43−7.38 (3H, m), 7.29−7.25 (5H, m), 7.09−7.08 (2H, m), 7.03 (1H, t, *J* = 7.2 Hz), 6.94 (2H, t, *J* = 7.9 Hz), 6.86–6.84 (2H, m), 6.29 (1H, d, *J* = 6.2 Hz); ^{13}C{^{1}H} NMR (150 MHz, $CDCl_3$/TMS) δ (ppm): 171.0, 141.2, 140.0, 139.8, 137.8, 137.0, 131.8, 131.7, 131.6, 130.9, 128.9, 128.5, 128.3, 127.6, 127.2, 121.5, 118.5, 109.0; LRMS (EI) *m/z*: 348 (M^+); HRMS (EI-TOF) Calcd. for $C_{24}H_{16}N_2O$: 348.1263, found: 348.1263; IR (neat): 2226, 1690, 1600, 1506, 1367, 1207, 1159, 970, 845, 799 cm^{-1}.

Methyl 4-(2-(diphenylmethylene)-5-oxo-2,5-dihydro-1*H*-pyrrol-1-yl)benzoate (2q)

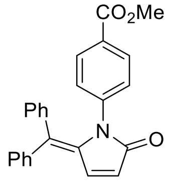

Obtained as colorless needles in 78% (58.9 mg), recrystallized from DCM/hexane, mp. 193−195 ˚C.

^{1}H NMR (600 MHz, $CDCl_3$/TMS) δ (ppm): 7.67 (2H, d, *J* = 8.2 Hz), 7.41–7.38 (3H, m), 7.27–7.25 (3H, m), 7.04 (2H, d, *J* = 8.2 Hz), 6.94 (1H, t, *J* = 7.2 Hz), 6.90 (2H, t, *J* = 7.6 Hz), 6.85 (2H, d, *J* = 6.8 Hz), 6.28 (1H, d, *J* = 6.2 Hz), 3.84 (3H, s); ^{13}C{^{1}H} NMR (150 MHz, $CDCl_3$/TMS) δ (ppm): 171.4, 166.4, 140.8, 140.2, 140.1, 137.9, 137.5, 131.65, 131.58, 131.57, 130.9, 129.3, 128.7, 128.2, 127.5, 127.1, 126.5, 121.6, 52.0; LRMS (EI) *m/z*: 381 (M^+); HRMS (EI-TOF) Calcd. for $C_{25}H_{19}NO_3$: 381.1365, found: 381.1341; IR (neat): 3062, 1722, 1696, 1601, 1507, 1437, 1361, 1275, 1167, 1102, 1072, 969, 863, 802 cm^{-1}.

1-(3-Bromophenyl)-5-(diphenylmethylene)-1,5-dihydro-2*H*-pyrrol-2-one (2r)

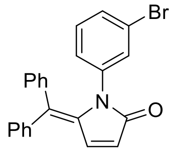

Obtained as yellow needles in 85% (68.2 mg), recrystallized from DCM/hexane, mp. 143−145 ˚C.
^{1}H NMR (600 MHz, $CDCl_3$/TMS) δ (ppm): 7.39–7.36 (3H, m), 7.25–7.23 (3H, m), 7.04 (1H, d, *J* = 8.2 Hz), 7.01–6.96 (5H, m), 6.89 (1H, t, *J* = 7.9 Hz), 6.86 (2H, d, *J* = 6.8 Hz), 6.27 (1H, d, *J* = 5.5 Hz); ^{13}C{^{1}H} NMR (150 MHz, $CDCl_3$/TMS) δ (ppm): 171.4, 140.5, 140.2, 137.8, 137.5, 136.9, 131.6, 131.3, 130.6, 130.2, 129.1, 128.9, 128.6, 128.14, 128.10, 127.4, 125.8, 121.6, 121.3; LRMS (EI) *m/z*: 401 (M^+); HRMS (EI-TOF) Calcd. for $C_{23}H_{16}{}^{79}BrNO$ (M^+): 401.0415, found: 401.0438; IR (neat): 1690, 1587, 1476, 1364, 1201, 1152, 980, 802 cm^{-1}.

5. General procedure for the synthesis of 5-alkylidene-pyrrolidin-2-ones

In a glove box, an amide **1** (0.20 mmol), CuBr (2.8 mg, 0.020 mmol), $AgBF_4$ (3.8 mg, 0.020 mmol), *tert*-butylpyridine (7.4 μL, 0.050 mmol), MnO_2 (52.2 mg, 0.60 mmol), and 1,2-dichloroethane (1.5 mL) were added to a sealed tube. The mixture was stirred at 120 °C for 24 h. After the reaction, the mixture was filtered through Celite and a SiO_2 pad with AcOEt and then the solvent was removed under a reduced pressure. The residue was purified by SiO_2 column chromatography.

Table S2. Copper optimization for the synthesis of **3a**.

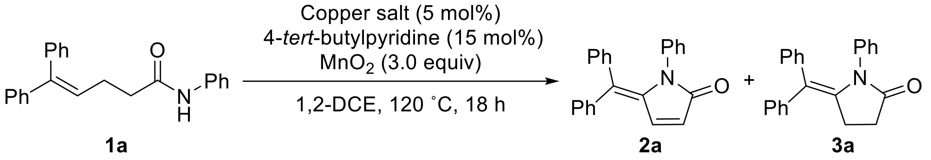

Entry	Copper salt	2a (%) [a]	3a (%) [a]
1 [b,c]	CuF_2	5	89
2 [b]	CuF_2	2	84
3	CuF_2	0	30
4	$Cu(acac)_2$	0	28
5	$Cu(BF_4)_2{\cdot}6H_2O$	0	49
6	$Cu(OAc)_2$	0	35
7	CuOAc	0	31
8	CuCl	0	23
9	$[(MeCN)_4Cu]BF_4$	0	28
10	$[(MeCN)_4Cu]PF_6$	0	36

[a] Determined by ^{1}H-NMR using 1,1,2-trichloroethane as an internal standard. [b] 10 mol% of CuF_2 and 1.0 equiv of 4-*tert*-butylpyridine were used. [c] 4.0 equiv of MnO_2 was used.

Table S3. Optimization of copper and silver salts for the synthesis of **3a**.

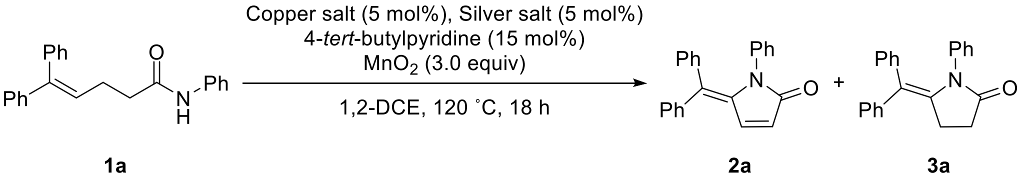

Entry	Copper salt	Silver salt	2a (%) [a]	3a (%) [a]
1	$Cu(BF_4)_2{\cdot}6H_2O$	None	0	49
2	CuBr	$AgBF_4$	2	69
3	CuBr	$AgSbF_6$	1	56
4	CuBr	$AgNTf_2$	3	30
5	None	$AgBF_4$	0	1
6 [b]	CuBr	$AgBF_4$	3	55

[a] Determined by ^{1}H-NMR using 1,1,2-trichloroethane as an internal standard.
[b] 10 mol% of copper and silver salts were used.

Table S4. Detailed reaction conditions for the synthesis of **3a**.

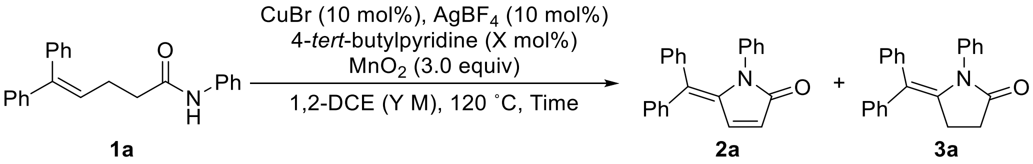

Entry	Time (h)	X (mol%)	Y (M)	2a (%) [a]	3a (%) [a]
1	18	15	0.080	3	55
2	24	15	0.080	3	67
3	24	25	0.080	3	74
4	24	35	0.080	1	50
6	24	25	0.067	1	42
7	24	25	0.10	3	69
**8**	**24**	**25**	**0.13**	**5**	**94 (91)**

[a] Determined by ^{1}H-NMR using 1,1,2-trichloroethane as an internal standard. Isolated yield in parentheses.

Scheme S1. Unsuccessful substrates under condition B.

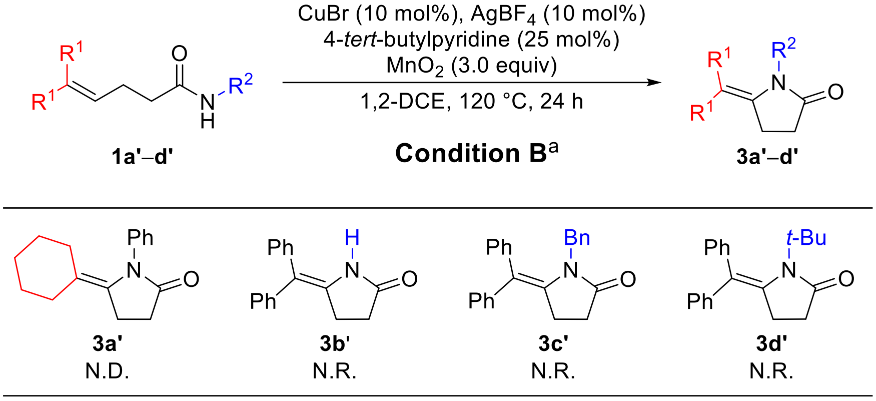

Yields were determined by ^{1}H-NMR using 1,1,2-trichloroethane as an internal standard.
[a] Reaction conditions: **1** (0.20 mmol), CuBr (0.020 mmol), $AgBF_4$ (0.020 mmol), 4-*tert*-butylpyridine (0.050 mmol), and MnO_2 (0.60 mmol) in 1,2-DCE (1.5 mL) at 120 °C for 24 h.

5-(Diphenylmethylene)-1-phenylpyrrolidin-2-one (**3a**)

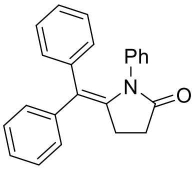

Obtained as colorless plates in 91% (59.0 mg), recrystallized from DCM/hexane, mp. 146–148 °C. ^{1}H NMR (600 MHz, $CDCl_3$/TMS) δ (ppm): 7.31–7.29 (2H, m), 7.25–7.22 (1H, m), 7.19–7.18 (2H, m), 7.00–6.96 (4H, m), 6.90–6.88 (1H, m), 6.83–6.81 (3H, m), 6.72–6.71 (2H, m), 2.99–2.97 (2H, m), 2.71–2.68 (2H, m); ^{13}C{^{1}H} NMR (150 MHz, $CDCl_3$/TMS) δ (ppm): 176.3, 142.1, 139.2, 137.9, 136.1, 130.1, 130.0, 128.2, 127.9, 127.1, 126.7, 126.2, 126.0, 125.9, 120.2, 30.9, 28.3; LRMS (EI) *m/z*: 325 (M^+); HRMS (EI-TOF) Calcd. for $C_{23}H_{19}NO$: 325.1467, found: 325.1456; IR (neat): 3046, 1723, 1620, 1595, 1495, 1359, 1160, 1027, 773, 751 cm^{-1}.

5-(Bis(4-methoxyphenyl)methylene)-1-phenylpyrrolidin-2-one (**3b**)

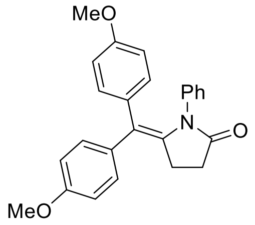

Obtained as yellow crystals in 61% (46.8 mg), recrystallized from DCM/hexane, mp. 164–166 ˚C. ^{1}H NMR (600 MHz, $CDCl_3$/TMS) δ (ppm): 7.09 (2H, d, *J* = 8.9 Hz), 6.99–6.98 (4H, m), 6.91–6.90 (1H, m), 6.84 (2H, d, *J* = 8.9 Hz), 6.61 (2H, d, *J* = 8.9 Hz), 6.36 (2H, d, *J* = 8.9 Hz), 3.80 (3H, s), 3.62 (3H, s), 2.96 (2H, t, *J* = 7.7 Hz), 2.68 (2H, t, *J* = 7.7 Hz); ^{13}C{^{1}H} NMR (150 MHz, $CDCl_3$/TMS) δ (ppm): 176.3, 158.3, 157.7, 136.7, 136.1, 134.6, 132.0, 131.1, 131.0, 127.9, 126.1, 125.9, 119.5, 113.5, 112.6, 55.2, 55,1, 31.1, 28.4; LRMS (EI) *m/z*: 385 (M^+); HRMS (EI-TOF) Calcd. for $C_{25}H_{23}NO_3$: 385.1678, found: 385.1706; IR (neat): 2969, 2835, 1713, 1606, 1508, 1358, 1179, 1027, 829, 761 cm^{-1}.

5-(Di-*p*-tolylmethylene)-1-phenylpyrrolidin-2-one (**3c**)

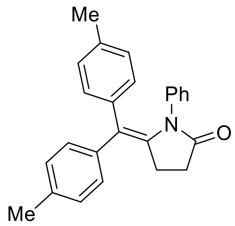

Obtained as colorless needles in 99% (69.4 mg, **3c** : **2c** = 93 : 7), recrystallized from DCM/hexane, mp. 208–211 ˚C.

^{1}H NMR (600 MHz, $CDCl_3$/TMS) δ (ppm): 7.10 (2H, d, *J* = 8.2 Hz), 7.06 (2H, d, *J* = 8.2 Hz), 6.98–6.95 (4H, m), 6.90–6.87 (1H, m), 6.61 (2H, d, *J* = 8.2 Hz), 6.58 (2H, d, *J* = 8.2 Hz), 2.97 (2H, t, *J* = 7.6 Hz), 2.68 (2H, t, *J* = 7.6 Hz), 2.34 (3H, s), 2.09 (3H, s); ^{13}C{^{1}H} NMR (150 MHz, $CDCl_3$/TMS) δ (ppm): 176.3, 139.3, 137.3, 136.4, 136.3, 136.2, 135.5, 129.9, 129.8, 128.8, 127.85, 127.76, 126.2, 125.7, 120.2, 31.1, 28.4, 21.1, 20.9; LRMS (EI) *m/z*: 353 (M^+); HRMS (EI-TOF) Calcd. for $C_{25}H_{23}NO$: 353.1780, found: 353.1775; IR (neat): 3019, 2927, 1732, 1636, 1496, 1369, 1228, 1154, 815 cm^{-1}.

5-(Bis(4-fluorophenyl)methylene)-1-phenylpyrrolidin-2-one (**3d**)

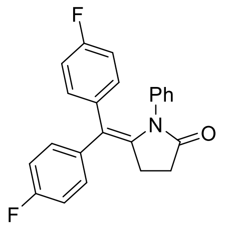

Obtained as colorless crystals in 80% (58.2 mg, **3d** : **2d** = 95 : 5), recrystallized from DCM/hexane, mp. 179–181 ˚C.

^{1}H NMR (600 MHz, $CDCl_3$/TMS) δ (ppm): 7.14–7.12 (2H, m), 7.03–6.94 (7H, m), 6.67–6.64 (2H, m), 6.52 (2H, t, J = 8.6 Hz), 2.95 (2H, t, J = 7.9 Hz), 2.70 (2H, t, J = 7.9 Hz); $^{13}C\{^1H\}$ NMR (150 MHz, $CDCl_3$/TMS) δ (ppm): 176.2, 161.6 ($^1J_{C\text{-}F}$ = 246.4 Hz), 161.0 ($^1J_{C\text{-}F}$ = 246.4 Hz), 138.4, 137.8 ($^4J_{C\text{-}F}$ = 4.3 Hz), 135.9, 135.1 ($^4J_{C\text{-}F}$ = 2.9 Hz), 131.55 ($^3J_{C\text{-}F}$ = 7.2 Hz), 131.48 ($^3J_{C\text{-}F}$ = 8.6 Hz), 128.1, 126.4, 126.3, 117.8, 115.2 ($^2J_{C\text{-}F}$ = 21.5 Hz), 114.1 ($^2J_{C\text{-}F}$ = 21.5 Hz), 30.8, 28.2; ^{19}F NMR (565 MHz, $CDCl_3$) δ (ppm): −114.5, −115.2; LRMS (EI) *m/z*: 361 (M^+); HRMS (EI-TOF) Calcd. for $C_{23}H_{17}F_2NO$: 361.1278, found: 361.1262; IR (neat): 3040, 1736, 1632, 1598, 1505, 1370, 1293, 1153, 831, 758 cm^{-1}.

5-(Bis(4-chlorophenyl)methylene)-1-phenylpyrrolidin-2-one (**3e**)

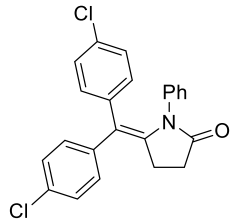

Obtained as colorless needles in 82% (65.5 mg, **3e** : **2e** = 94 : 6), recrystallized from DCM/hexane, mp. 204–206 ˚C.

^{1}H NMR (600 MHz, $CDCl_3$/TMS) δ (ppm): 7.28 (2H, d, J = 8.2 Hz), 7.09 (2H, d, J = 8.2 Hz), 7.04–7.01 (2H, m), 6.98 (1H, d, J = 6.9 Hz), 6.95 (2H, d, J = 7.6 Hz), 6.79 (2H, d, J = 8.2 Hz), 6.61 (2H, d, J = 8.2 Hz), 2.96 (2H, t, J = 7.7 Hz), 2.71 (2H, t, J = 7.7 Hz); $^{13}C\{^1H\}$ NMR (150 MHz, $CDCl_3$/TMS) δ (ppm): 176.1, 140.0, 139.1, 137.3, 135.8, 132.7, 132.0, 131.30, 131.26, 128.5, 128.2, 127.4, 126.5, 126.3, 117.4, 30.7, 28.2; LRMS (EI) *m/z*: 393 (M^+); HRMS (EI-TOF) Calcd. for $C_{23}H_{17}{}^{35}Cl_2NO$: 393.0687, found: 393.0671; IR (neat): 3064, 2944, 1732, 1612, 1488, 1355, 1156, 1012, 828, 756 cm^{-1}.

5-(5*H*-Dibenzo[*a*,*d*][7]annulen-5-ylidene)-1-phenylpyrrolidin-2-one (**3h**)

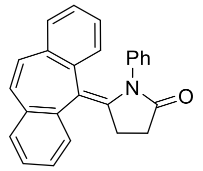

Obtained as colorless crystals in 66% (46.3 mg), recrystallized from DCM/hexane, mp. 199–200 ˚C.

^{1}H-NMR (600 Hz, DMSO-d_6) δ (ppm): 7.42–7.36 (3H, m), 7.27–7.24 (1H, m), 7.02–6.89 (6H, m), 6.83–6.56 (5H, m), 3.12–3.08 (1H, m), 2.70–2.64 (1H, m), 2.45–2.41 (1H, m), 2.26–2.21

(1H, m); $^{13}C\{^1H\}$ NMR (150 Hz, DMSO-d_6), δ (ppm): 176.3, 138.3, 137.1, 136.8, 136.4, 134.9, 133.8, 131.3, 130.9, 128.9, 128.7, 128.3, 128.1, 127.8, 127.3, 127.2, 127.0, 126.4, 126.3, 125.6, 114.1, 28.7, 24.2; LRMS (EI) *m/z*: 349 (M^+); HRMS (EI-TOF) Calcd. for $C_{25}H_{19}NO$: 349.1467, found: 349.1465; IR (neat): 3017, 1717, 1636, 1497, 1370, 1239, 1171, 803, 738 cm^{-1}.

5-(Diphenylmethylene)-1-(4-methoxyphenyl)pyrrolidin-2-one (3i)

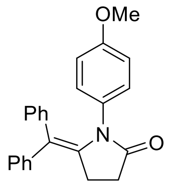

Obtained as colorless needles in 76% (54.7 mg, **3i** : **2i** = 93 : 7), recrystallized from DCM/hexane, mp. 158−161 °C.

^1H NMR (600 MHz, $CDCl_3$/TMS) δ (ppm): 7.29 (2H, t, *J* = 7.6 Hz), 7.22 (1H, t, *J* = 7.2 Hz), 7.18 (2H, d, *J* = 6.9 Hz), 6.88–6.84 (5H, m), 6.71–6.70 (2H, m), 6.50 (2H, d, *J* = 8.9 Hz), 3.65 (3H, s), 2.96 (2H, t, *J* = 7.9 Hz), 2.67 (2H, t, *J* = 7.9 Hz); $^{13}C\{^1H\}$ NMR (150 MHz, $CDCl_3$/TMS) δ (ppm): 176.6, 157.6, 142.2, 139.1, 138.2, 130.2, 129.9, 129.1, 128.2, 127.5, 127.1, 126.6, 125.9, 119.7, 113.4, 55.4, 30.7, 28.1; LRMS (EI) *m/z*: 355 (M^+); HRMS (EI-TOF) Calcd. for $C_{24}H_{21}NO_2$: 355.1572, found: 355.1549; IR (neat): 3257, 1700, 1636, 1511, 1444, 1242, 1031, 741 cm^{-1}.

5-(Diphenylmethylene)-1-(*p*-tolyl)pyrrolidin-2-one (3j)

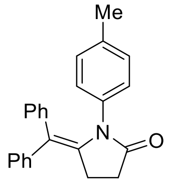

Obtained as yellow oil in 92% (62.7 mg, **3j** : **2j** = 93 : 7).

^1H NMR (600 MHz, $CDCl_3$/TMS) δ (ppm): 7.30 (2H, t, *J* = 7.5 Hz), 7.22 (1H, tt, *J* = 7.4, 1.5 Hz), 7.19–7.17 (2H, m), 6.85–6.80 (5H, m), 6.76 (2H, d, *J* = 8.1 Hz), 6.71–6.69 (2H, m), 2.98–2.95 (2H, m), 2.69–2.67 (2H, m), 2.13 (3H, s); $^{13}C\{^1H\}$ NMR (150 MHz, $CDCl_3$/TMS) δ (ppm): 176.5, 142.2, 139.2, 138.1, 135.8, 133.5, 130.2, 130.0, 128.5, 128.2, 127.1, 126.6, 126.2, 125.7, 119.9, 30.8, 28.2, 20.8; LRMS (EI) *m/z*: 339 (M^+); HRMS (EI-TOF) Calcd. for $C_{24}H_{21}NO$: 339.1623, found: 339.1617; IR (neat): 3057, 1718, 1631, 1512, 1364, 1229, 1167, 1030, 816, 751 cm^{-1}.

5-(Diphenylmethylene)-1-(4-isopropylphenyl)pyrrolidin-2-one (**3k**)

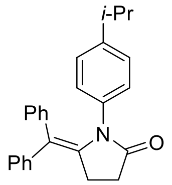

Obtained as colorless oil in 99% (72.5 mg, **3k** : **2k** = 92 : 8).

^{1}H NMR (600 MHz, $CDCl_3$/TMS) δ (ppm): 7.29 (2H, t, J = 7.6 Hz), 7.22 (1H, t, J = 7.6 Hz), 7.18 (2H, d, J = 7.6 Hz), 6.85 (2H, d, J = 8.9 Hz), 6.80–6.79 (5H, m), 6.67 (2H, dd, J = 6.5, 3.1 Hz), 2.96 (2H, t, J = 7.9 Hz), 2.70–2.65 (3H, m), 1.09 (6H, d, J = 6.8 Hz); ^{13}C{^{1}H} NMR (150 MHz, $CDCl_3$/TMS) δ (ppm): 176.5, 146.8, 142.2, 139.1, 138.1, 133.6, 130.1, 129.9, 128.2, 127.0, 126.5, 126.3, 126.0, 125.8, 119.7, 33.7, 30.7, 28.1, 23.9; LRMS (EI) *m/z*: 367 (M^+); HRMS (EI-TOF) Calcd. for $C_{26}H_{25}NO$: 367.1936, found: 367.1925; IR (neat): 3018, 2959, 1723, 1630, 1512, 1364, 1300, 1229, 1167, 832 cm^{-1}.

5-(Diphenylmethylene)-1-(4-fluorophenyl)pyrrolidin-2-one (**3l**)

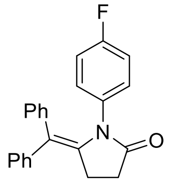

Obtained as colorless needle in 92% (63.4 mg, **3l** : **2l** = 93 : 7), recrystallized from DCM/hexane, mp. 128–130 °C.

^{1}H NMR (600 MHz, $CDCl_3$/TMS) δ (ppm): 7.32–7.29 (2H, m), 7.25–7.22 (1H, m), 7.18–7.16 (2H, m), 6.96–6.93 (2H, m), 6.91–6.85 (3H, m), 6.72–6.70 (2H, m), 6.68–6.65 (2H, m), 3.00–2.97 (2H, m), 2.70–2.68 (2H, m); ^{13}C{^{1}H} NMR (150 MHz, $CDCl_3$/TMS) δ (ppm): 176.4, 160.5 ($^1J_{C\text{-}F}$ = 245.0 Hz), 141.9, 139.1, 137.9, 132.1 ($^4J_{C\text{-}F}$ = 2.9 Hz) 130.2, 129.9, 128.2, 128.0 ($^3J_{C\text{-}F}$ = 8.6 Hz), 127.3, 126.8, 126.2, 120.2, 114.8 ($^2J_{C\text{-}F}$ = 22.9 Hz), 30.8, 28.2; ^{19}F NMR (565 MHz, $CDCl_3$) δ (ppm): –115.1; LRMS (EI) *m/z*: 343 (M^+); HRMS (EI-TOF) Calcd. For $C_{23}H_{18}FN$: 343.1372, found: 343.1352; IR (neat): 3058, 1724, 1633, 1604, 1507, 1368, 1299, 1228, 1153, 910, 752 cm^{-1}.

1-(4-Chlorophenyl)-5-(diphenylmethylene)pyrrolidin-2-one (**3m**)

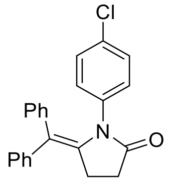

Obtained as colorless needles in 76% (55.4 mg), recrystallized from DCM/hexane, mp. 165−167 °C.

^{1}H NMR (600 MHz, $CDCl_3$/TMS) δ (ppm): 7.31 (2H, t, J = 7.6 Hz), 7.25–7.22 (1H, m), 7.18–7.17 (2H, m), 6.94–6.86 (7H, m), 6.72–6.70 (2H, m), 2.98 (2H, t, J = 7.9 Hz), 2.69 (2H, t, J = 7.9 Hz); ^{13}C{^{1}H} NMR (150 MHz, $CDCl_3$/TMS) δ (ppm): 176.1, 141.7, 139.0, 137.6, 134.6, 131.4, 130.1, 129.9, 128.2, 128.0, 127.42, 127.38, 126.8, 126.2, 120.5, 30.9, 28.2; LRMS (EI) *m/z*: 359 (M^+); HRMS (EI-TOF) Calcd. for $C_{23}H_{18}{}^{35}ClNO$: 359.1077, found: 359.1092; IR (neat): 3076, 2929, 1719, 1636, 1492, 1364, 1300, 1233, 1165, 1088, 833 cm^{-1}.

1-(4-Bromophenyl)-5-(diphenylmethylene)pyrrolidin-2-one (**3n**)

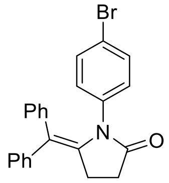

Obtained as colorless needles in 80% (64.7 mg, **3n** : **2n** = 96 : 4), recrystallized from DCM/hexane, mp. 188−189 °C.

^{1}H NMR (600 MHz, $CDCl_3$/TMS) δ (ppm): 7.30 (2H, t, J = 7.6 Hz), 7.25–7.24 (1H, m), 7.17 (2H, d, J = 7.5 Hz), 7.08 (2H, d, J = 8.9 Hz), 6.91 (1H, t, J = 9.6 Hz), 6.87–6.85 (4H, m), 6.70 (2H, d, J = 7.6 Hz), 2.98 (2H, t, J = 7.9 Hz), 2.68 (2H, t, J = 7.9 Hz); ^{13}C{^{1}H} NMR (150 MHz, $CDCl_3$/TMS) δ (ppm): 176.1, 141.7, 139.0, 137.5, 135.1, 131.0, 130.1, 129.9, 128.2, 127.7, 127.4, 126.8, 126.2, 120.5, 119.4, 30.9, 28.2; LRMS (EI) *m/z*: 403 (M^+); HRMS (EI-TOF) Calcd. For $C_{23}H_{18}{}^{79}BrNO$: 403.0572, found: 403.0550; IR (neat): 3074, 1718, 1636, 1488, 1365, 1301, 1235, 1166, 1067, 1012 cm^{-1}.

5-(Diphenylmethylene)-1-(4-(trifluoromethyl)phenyl)pyrrolidin-2-one (**3o**)

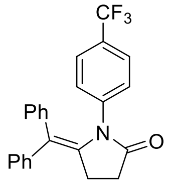

Obtained as yellow crystals in 69% (54.4 mg, **3o** : **2o** = 95 : 5), recrystallized from DCM/hexane, mp. 169−170 °C.

^{1}H NMR (600 MHz, $CDCl_3$/TMS) δ (ppm): 7.32 (2H, t, J = 7.6 Hz), 7.26–7.18 (5H, m), 7.10 (2H, d, J = 8.3 Hz), 6.84–6.83 (3H, m), 6.69 (2H, dd, J = 7.6, 1.4 Hz), 3.01 (2H, t, J = 7.7 Hz), 2.72 (2H, t, J = 7.7 Hz); ^{13}C{^{1}H, ^{19}F} NMR (150 MHz, $CDCl_3$/TMS) δ (ppm): 176.0, 141.5, 139.1, 139.0, 137.2, 130.0, 129.9, 128.2, 127.8, 127.4, 126.9, 126.4, 126.3, 125.0, 123.7, 121.0, 31.0, 28.2; ^{19}F NMR (565 MHz, $CDCl_3$) δ (ppm): –62.1; LRMS (EI) *m/z*: 393 (M^+); HRMS (EI-TOF)

Calcd. For $C_{24}H_{18}F_3NO$: 393.1340, found: 393.1328; IR (neat): 3060, 1721, 1636, 1592, 1490, 1366, 1324, 1232, 1161, 851, 753 cm^{-1}.

4-(2-(Diphenylmethylene)-5-oxopyrrolidin-1-yl)benzonitrile (**3p**)

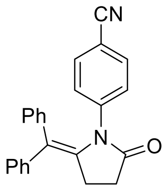

Obtained as colorless crystals in 84% (59.1 mg, **3p** : **2p** = 98 : 2), recrystallized from DCM/hexane, mp. 200–201 ˚C.

^{1}H NMR (600 MHz, $CDCl_3$/TMS) δ (ppm): 7.32 (2H, t, *J* = 7.5 Hz), 7.28–7.25 (3H, m), 7.18 (2H, d, *J* = 6.9 Hz), 7.14 (2H, d, *J* = 8.3 Hz), 6.90–6.86 (3H, m), 6.72–6.71 (2H, m), 3.01 (2H, t, *J* = 7.7 Hz), 2.73 (2H, t, *J* = 7.7 Hz); ^{13}C{^{1}H} NMR (150 MHz, $CDCl_3$/TMS) δ (ppm): 175.8, 141.2, 140.0, 139.0, 136.73, 136.72, 131.8, 129.9, 128.3, 127.6, 127.1, 126.7, 126.2, 121.8, 118.5, 108.9, 31.2, 28.3; LRMS (EI) *m/z*: 350 (M^+); HRMS (EI-TOF) Calcd. For $C_{24}H_{18}N_2O$: 350.1419, found: 350.1396; IR (neat): 3044, 2224, 1721, 1635, 1601, 1506, 1355, 1227, 1162, 844 cm^{-1}.

Methyl 4-(2-(diphenylmethylene)-5-oxopyrrolidin-1-yl)benzoate (**3q**)

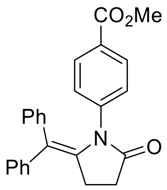

Obtained as colorless crystals in 84% (64.4 mg, **3q** : **2q** = 98 : 2), recrystallized from DCM/hexane, mp. 191–192 ˚C.

^{1}H NMR (600 MHz, $CDCl_3$/TMS) δ (ppm): 7.65 (2H, d, *J* = 8.9 Hz), 7.31 (2H, t, *J* = 7.6 Hz), 7.26–7.24 (1H, m), 7.19 (2H, d, *J* = 6.8 Hz), 7.09 (2H, d, *J* = 8.2 Hz), 6.85–6.81 (3H, m), 6.73 (2H, d, *J* = 7.6 Hz), 3.84 (3H, s), 3.00 (2H, t, *J* = 7.9 Hz), 2.71 (2H, t, *J* = 7.9 Hz); ^{13}C{^{1}H} NMR (150 MHz, $CDCl_3$/TMS) δ (ppm): 176.0, 166.4, 141.6, 140.2, 139.0, 137.3, 130.0, 129.3, 128.2, 127.4, 127.1, 126.9, 126.40, 126.39, 125.6, 121.4, 52.0, 31.2, 28.4; LRMS (EI) *m/z*: 383 (M^+); HRMS (EI-TOF) Calcd. For $C_{25}H_{21}NO_3$: 383.1521, found: 383.1511; IR (neat): 3056, 2952, 1723, 1710, 1630, 1439, 1361, 1278, 1227, 855, 765 cm^{-1}.

1-(3-Bromophenyl)-5-(diphenylmethylene)pyrrolidin-2-one (3r)

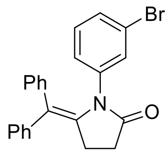

Obtained as yellow crystals in 78% (63.2 mg, **3r** : **2r** = 95 : 5), recrystallized from DCM/hexane, mp. 138−140 ˚C.

^{1}H NMR (600 MHz, $CDCl_3$/TMS) δ (ppm): 7.31 (2H, t, *J* = 7.5 Hz), 7.25–7.23 (1H, m), 7.18 (2H, d, *J* = 7.5 Hz), 7.06 (1H, s), 7.01 (2H, d, *J* = 8.0 Hz), 6.92–6.85 (4H, m), 6.74 (2H, d, *J* = 6.5 Hz), 2.99 (2H, t, *J* = 7.8 Hz), 2.69 (2H, t, *J* = 7.8 Hz); $^{13}C\{^1H\}$ NMR (150 MHz, $CDCl_3$/TMS) δ (ppm): 176.0, 141.6, 139.0, 137.4, 137.1, 129.93, 129.91, 129.4, 129.1, 129.0, 128.2, 127.4, 126.8, 126.3, 124.9, 121.4, 120.8, 30.9, 28.2; LRMS (EI) *m/z*: 403 (M^+); HRMS (EI-TOF) Calcd. For $C_{23}H_{18}{}^{79}BrNO$: 403.0572, found: 403.0580; IR (neat): 3057, 1724, 1631, 1590, 1571, 1476, 1352, 1220, 1155, 764 cm^{-1}.

1-(Benzo[*d*]thiazol-2-yl)-5-(diphenylmethylene)pyrrolidin-2-one (3s)

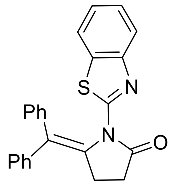

Obtained as colorless needles in 38% (29.9 mg), recrystallized from DCM/hexane, mp. 192−193 ˚C.

^{1}H NMR (600 MHz, $CDCl_3$/TMS) δ (ppm): 7.64 (1H, d, *J* = 8.2 Hz), 7.45 (1H, d, *J* = 8.3 Hz), 7.34–7.23 (6H, m), 7.19 (1H, t, *J* = 7.2 Hz), 6.93 (2H, d, *J* = 7.6 Hz), 6.82 (2H, t, *J* = 7.9 Hz), 6.64 (1H, t, *J* = 7.5 Hz), 3.08 (2H, t, *J* = 7.5 Hz), 2.79 (2H, t, *J* = 7.5 Hz); $^{13}C\{^1H\}$ NMR (150 MHz, $CDCl_3$/TMS) δ (ppm): 175.1, 154.2, 148.3, 140.9, 140.3, 134.8, 133.1, 130.2, 129.0, 128.1, 127.3, 127.2, 126.4, 125.7, 125.5, 124.3, 122.1, 120.8, 31.5, 28.5; LRMS (EI) *m/z*: 382 (M^+); HRMS (EI-TOF) Calcd. for $C_{24}H_{18}N_2OS$ (M^+): 382.1140, found: 382.1167; IR (neat): 3048, 2914, 1723, 1635, 1512, 1279, 1234, 1168, 749 cm^{-1}.

6. Mechanistic experiment

5-(Chlorodiphenylmethyl)-1-phenylpyrrolidin-2-one (4a)

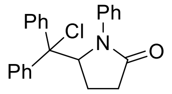

In a glove box, **1a** (65.6 mg, 0.20 mmol), CuF_2 (2.0 mg, 0.020 mmol), 4-*tert*-butylpyridine (29.3 µL, 0.020 mmol), *t*BuOO*t*Bu (147.0 µL, 0.80 mmol), and 1,2-dichloroethane (2.5 mL) were added

to a sealed tube. The mixture was stirred at 120 °C for 3 h. The reaction was diluted with water (10 mL) and extracted with chloroform (10 mL × 3). The organic layers were washed with brine (10 mL) and dried over $MgSO_4$ and the solvent was removed under a reduced pressure. The residue was purified by SiO_2 column chromatography.

Obtained as colorless needles in 50% (36.2 mg), recrystallized from DCM/hexane, mp. 156–157 °C.

^{1}H NMR (600 MHz, $CDCl_3$/TMS) δ (ppm): 7.43 (2H, d, *J* = 6.9 Hz), 7.31–7.23 (5H, m), 7.09 (4H, d, *J* = 4.8 Hz), 7.05–7.01 (3H, m), 7.00–6.96 (1H, m), 5.62 (1H, d, *J* = 8.3 Hz), 2.62–2.54 (1H, m), 2.34–2.28 (2H, m), 2.24–2.15 (1H, m); ^{13}C{^{1}H} NMR (150 MHz, $CDCl_3$/TMS) δ (ppm): 175.9, 142.6, 141.2, 138.7, 128.3, 128.2, 128.1, 127.9, 127.8, 127.5, 125.8, 125.77, 125.76, 82.5, 67.5, 30.5, 24.2; LRMS (FAB) *m/z*: 362 $(M+H)^+$; HRMS (FAB-EB) Calcd. For $C_{23}H_{21}{}^{35}ClNO$ $(M+H)^+$: 362.1312, found: 362.1324; IR (neat): 3067, 1689, 1599, 1499, 1404, 1291, 1039, 749 cm^{-1}.

7. X-Ray crystallographic data for 4a

Single crystals suitable for X-ray crystallography were obtained by vapor diffusion of hexane into a AcOEt solution of **4a**. A suitable crystal was selected and mounted on a Bruker D8 goniometer diffractometer. The crystal was kept at 93(1) K during data collection. Using Olex2[1)], the structure was solved with the olex2.solve[2)] structure solution program using Charge Flipping and refined with the SHELXL[3)] refinement package using Least Squares minimization.

Crystallographic data of **4a** has been deposited with Cambridge Crystallographic Data Center, Deposition no. CCDC 2284469. The data can be obtained free of charge from the Cambridge Crystallographic Data Centre via www.ccdc.cam.ac.uk/data_request/cif.

1) Dolomanov, O. V., Bourhis, L. J., Gildea, R. J, Howard, J. A. K. & Puschmann, H. (2009), J. Appl. Cryst. 42, 339–341.

2) Bourhis, L. J., Dolomanov, O. V., Gildea, R. J., Howard, J. A. K., Puschmann, H. (2015). Acta Cryst. A71, 59–75.

3) Sheldrick, G. M. (2015). Acta Cryst. C71, 3–8.

Table S5. Crystal data and structure refinements for **4a**.

Empirical formula	$C_{23}H_{20}ClNO$
Formula weight	361.85
Temperature/K	90(1)
Crystal system	triclinic
Space group	*P*-1
a/Å	12.583(3)
b/Å	13.818(3)
c/Å	22.012(5)
α /°	106.734(9)
β /°	90.764(8)
γ /°	90.173(8)
Volume/Å^3	3664.8(15)
Z	8
ρ calcg/cm3	1.312
μ /mm-1	0.220
F(000)	1520.0
Crystal size/mm3	0.58 × 0.21 × 0.15
Radiation	MoKα (λ = 0.71073)
2Θ range for data collection/°	3.864 to 55.906
Index ranges	$-16 \leq h \leq 16$, $-18 \leq k \leq 17$, $0 \leq l \leq 29$
Reflections collected	17725
Independent reflections	17725 [R_{sigma} = 0.0753]
Data/restraints/parameters	17725/0/938
Goodness-of-fit on F^2	1.029
Final *R* indexes [*I*>=2σ (*I*)]	R_1 = 0.0599, wR_2 = 0.1445
Final *R* indexes [all data]	R_1 = 0.0883, wR_2 = 0.1607
Largest diff. peak/hole / e Å^{-3}	0.61/-0.66

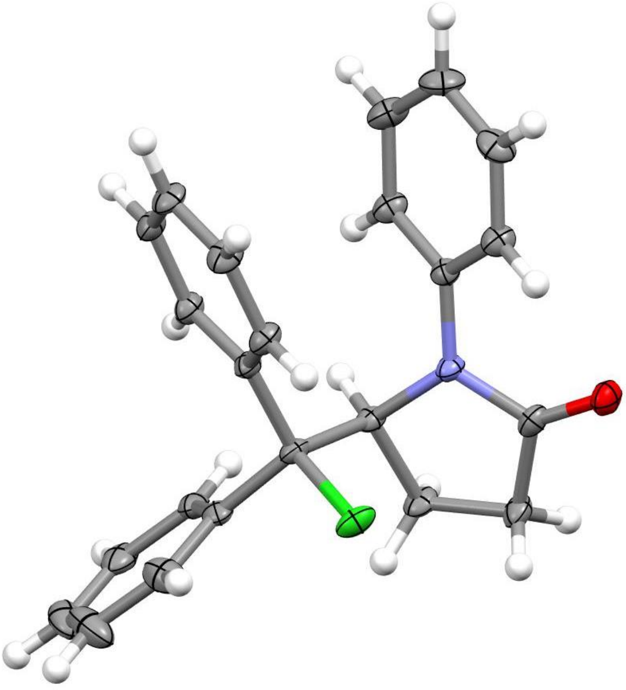

Figure S1. ORTEP diagram of **4a** with thermal ellipsoids drawn at 50% probability level (CCDC No. 2284469). Color code of atoms: hydrogen, white; carbon, gray; chlorine, green; nitrogen, blue; oxygen, red.

8. ^{1}H-, ^{13}C-, and ^{19}F-NMR spectra

^{1}H-NMR spectrum of **1a** ($CDCl_3$, 400 MHz)

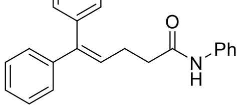

1a

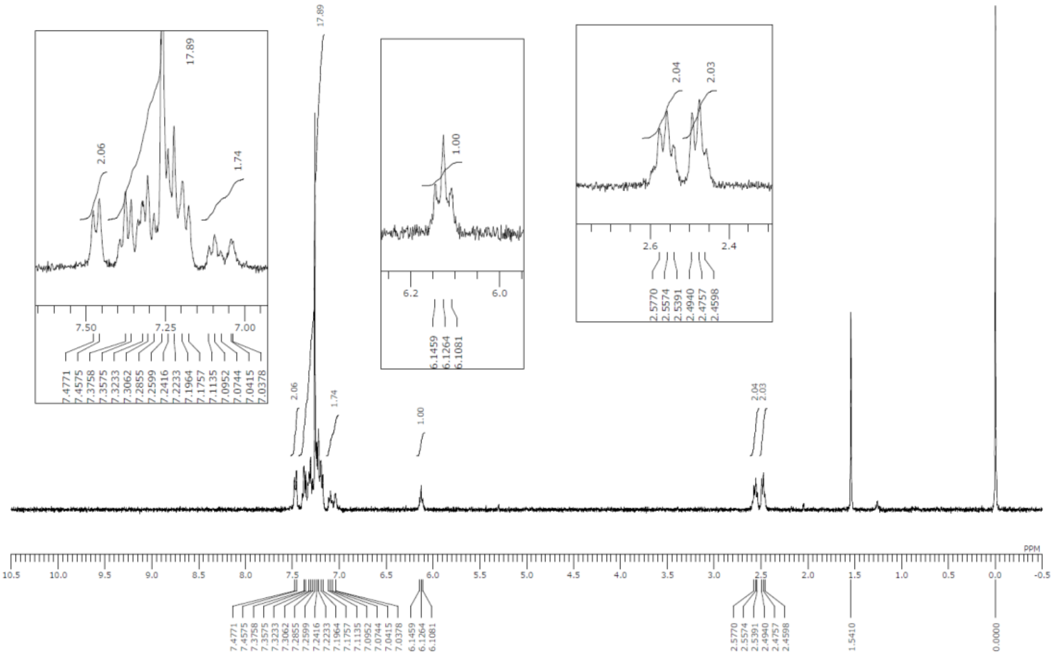

^{13}C-NMR spectrum of **1a** ($CDCl_3$, 100 MHz)

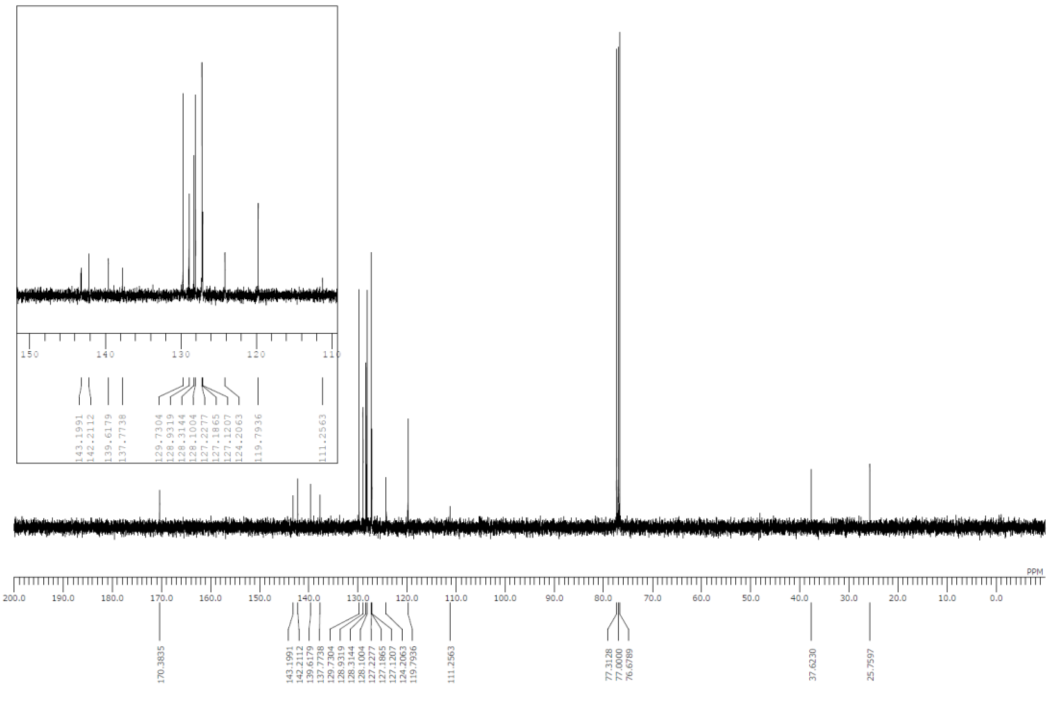

IR spectrum of **1a**

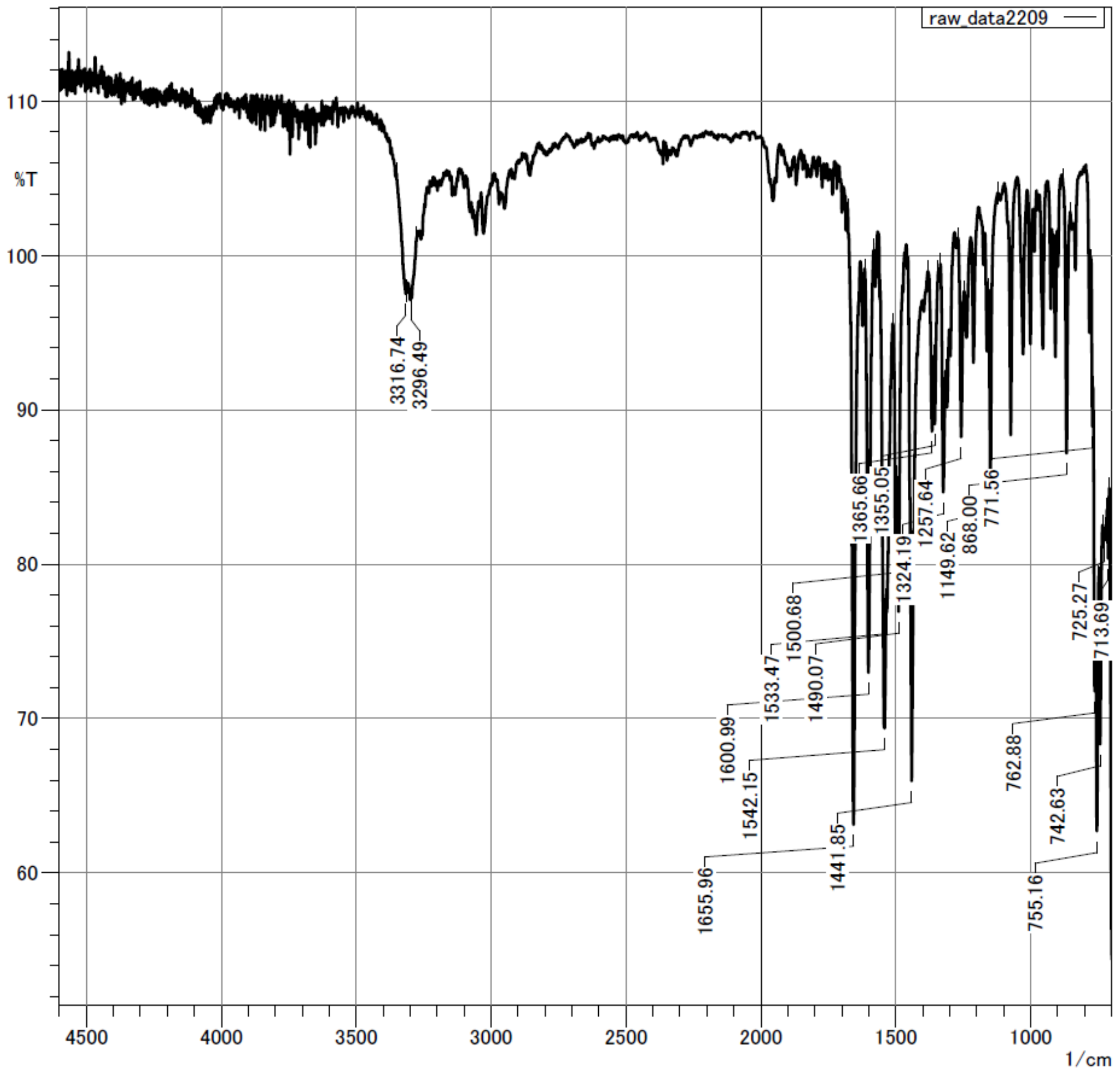

HRMS spectrum of **1a**

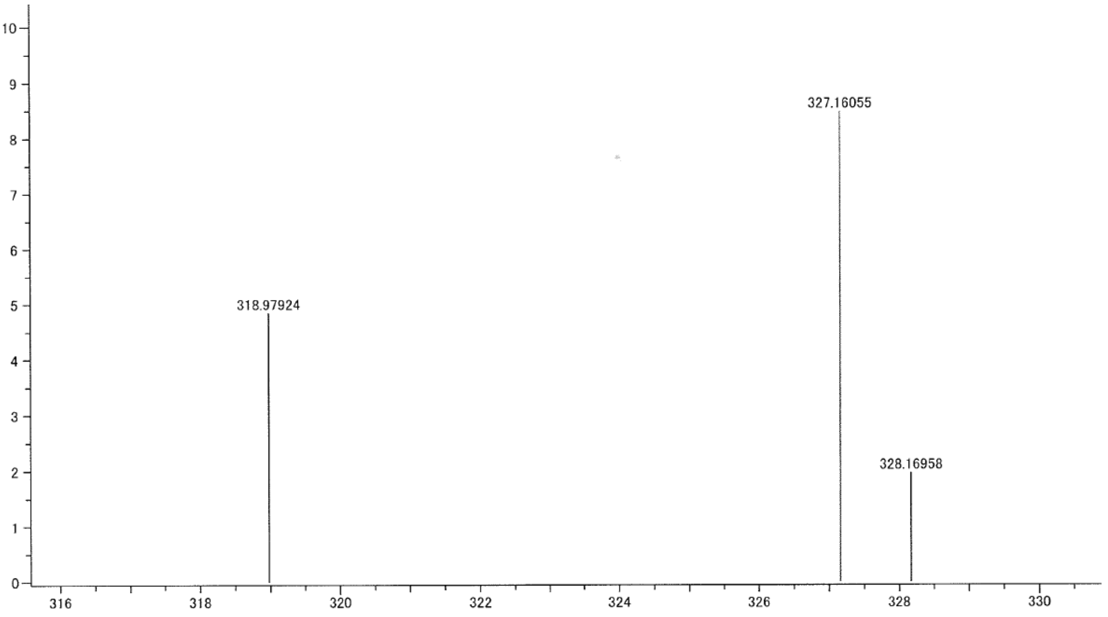

^{1}H-NMR spectrum of **1b** ($CDCl_3$, 600 MHz)

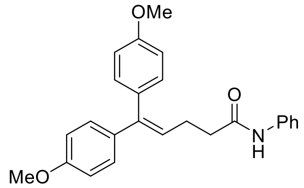

1b

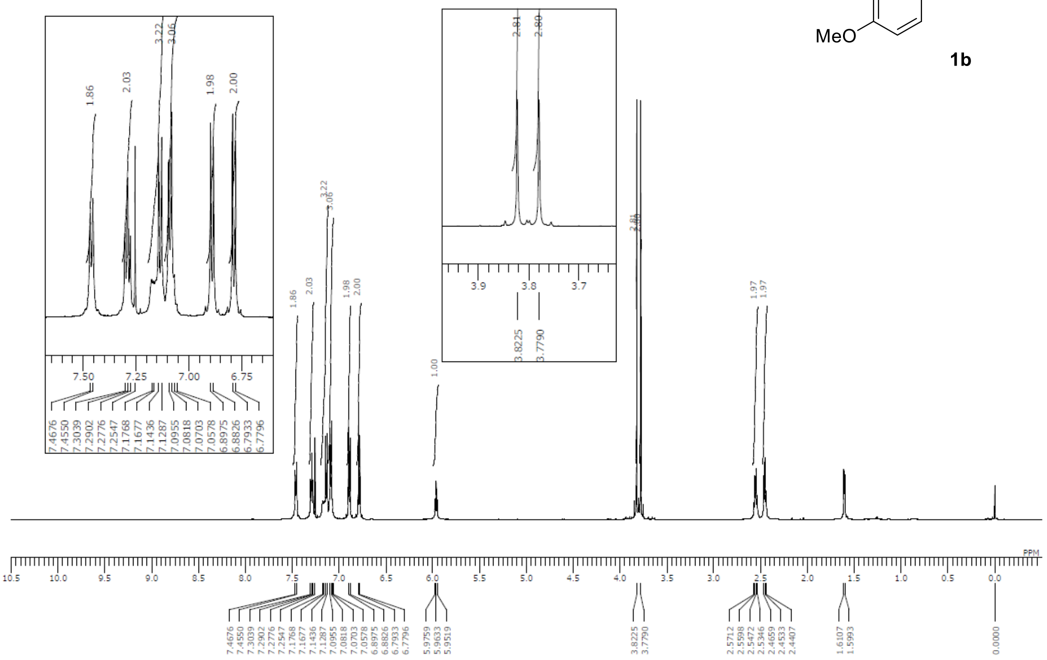

^{13}C-NMR spectrum of **1b** ($CDCl_3$, 150 MHz)

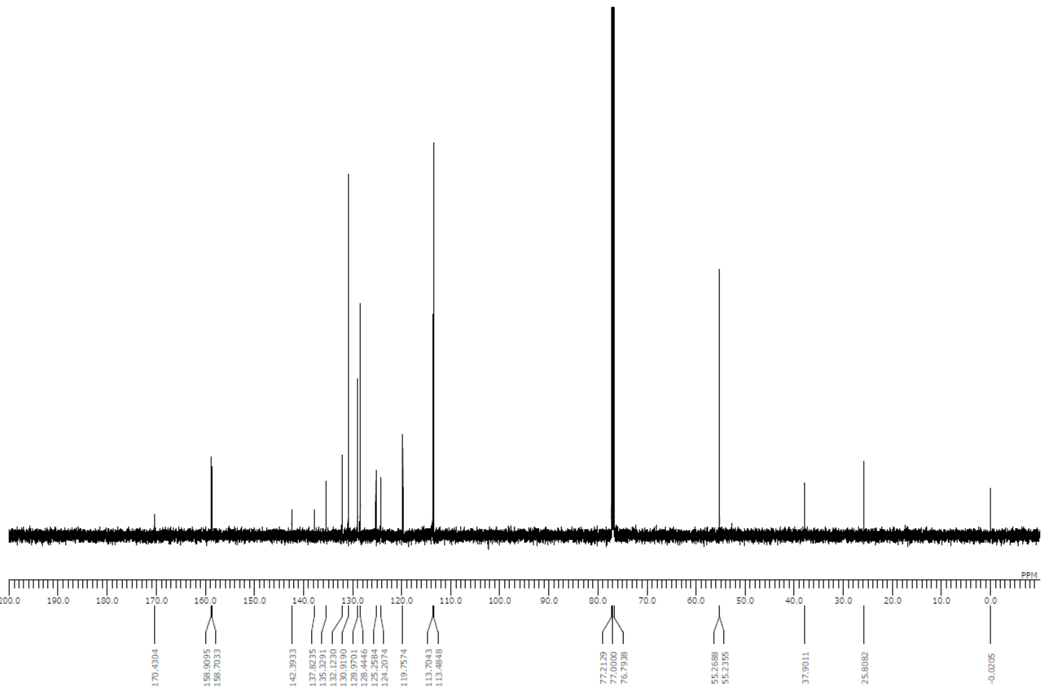

IR spectrum of **1b**

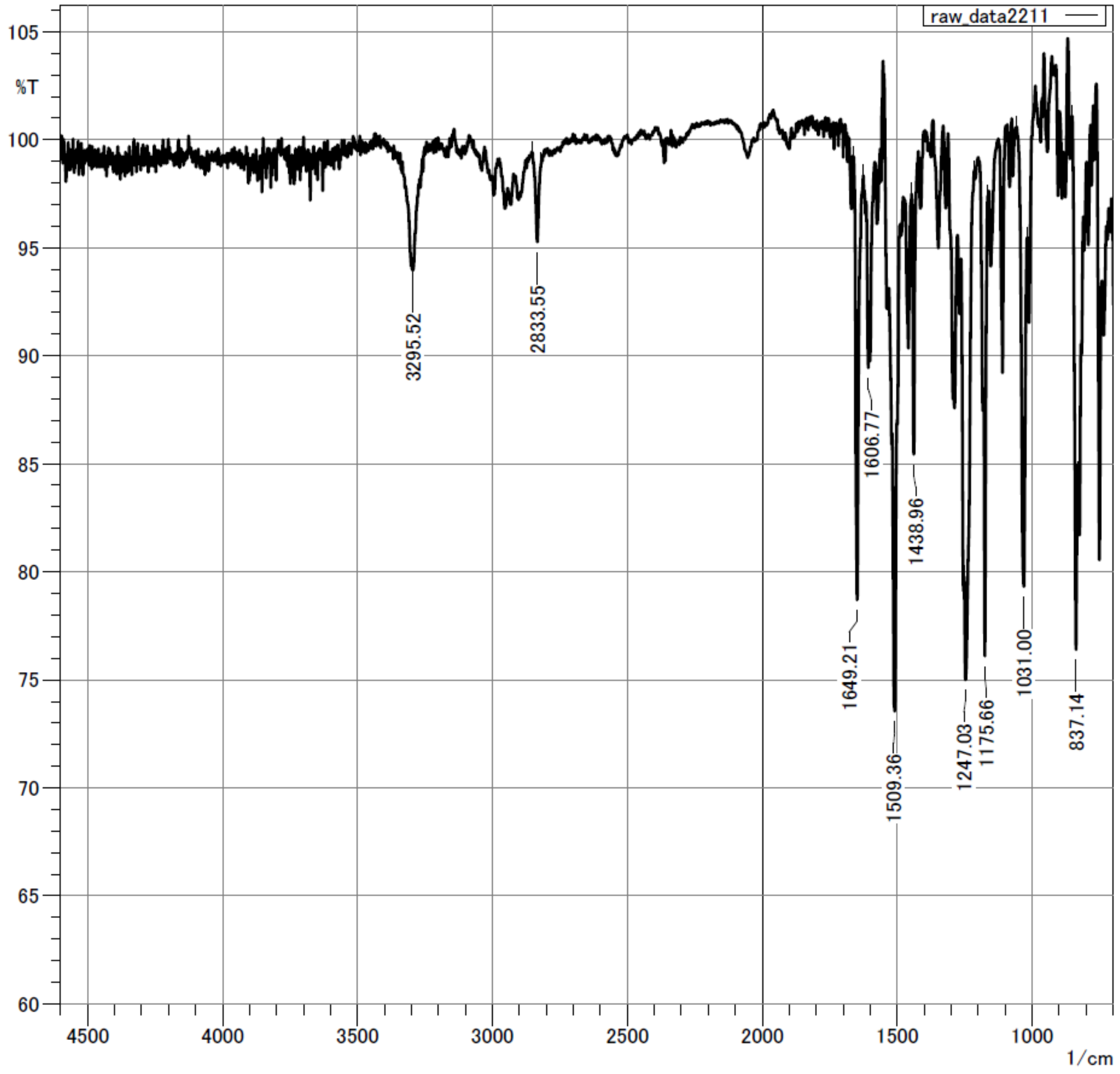

HRMS spectrum of **1b**

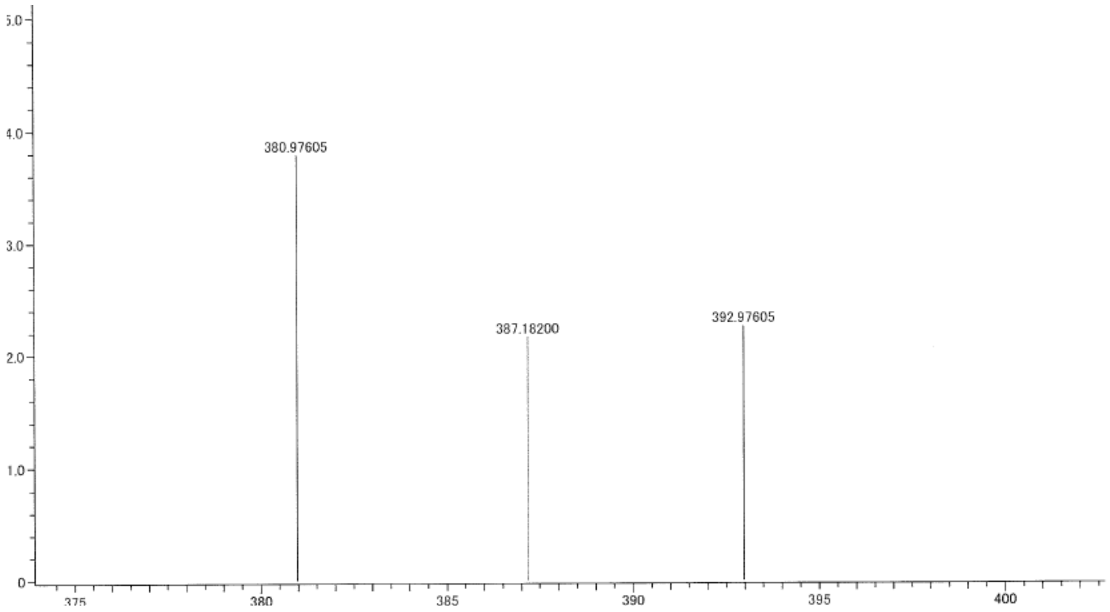

^{1}H-NMR spectrum of **1c** ($CDCl_3$, 600 MHz)

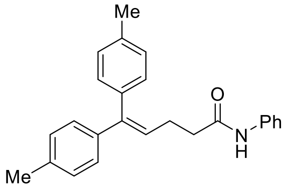

1c

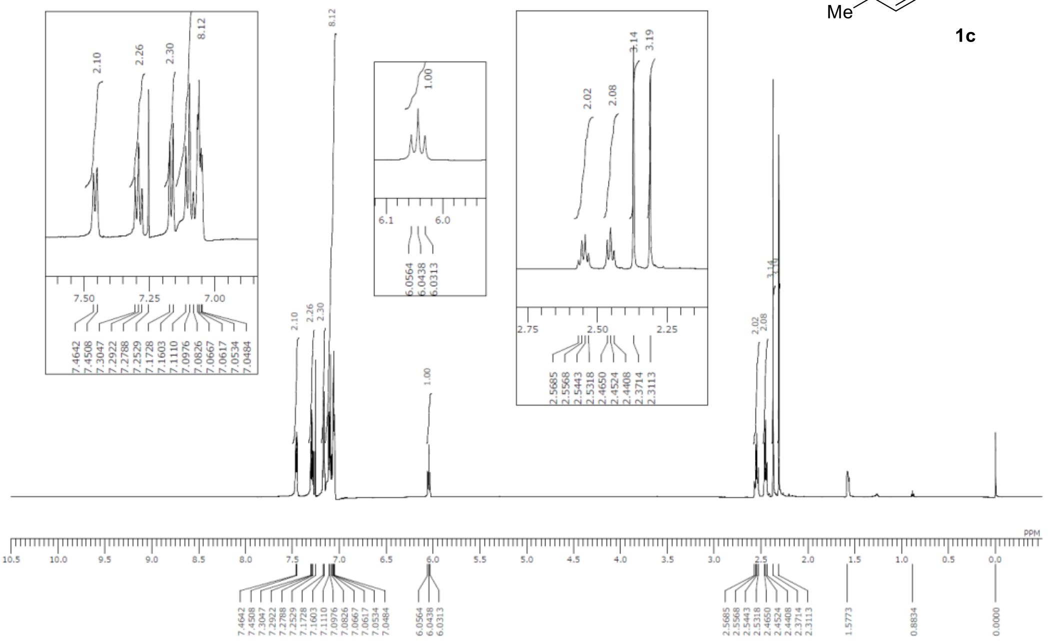

^{13}C-NMR spectrum of **1c** ($CDCl_3$, 150 MHz)

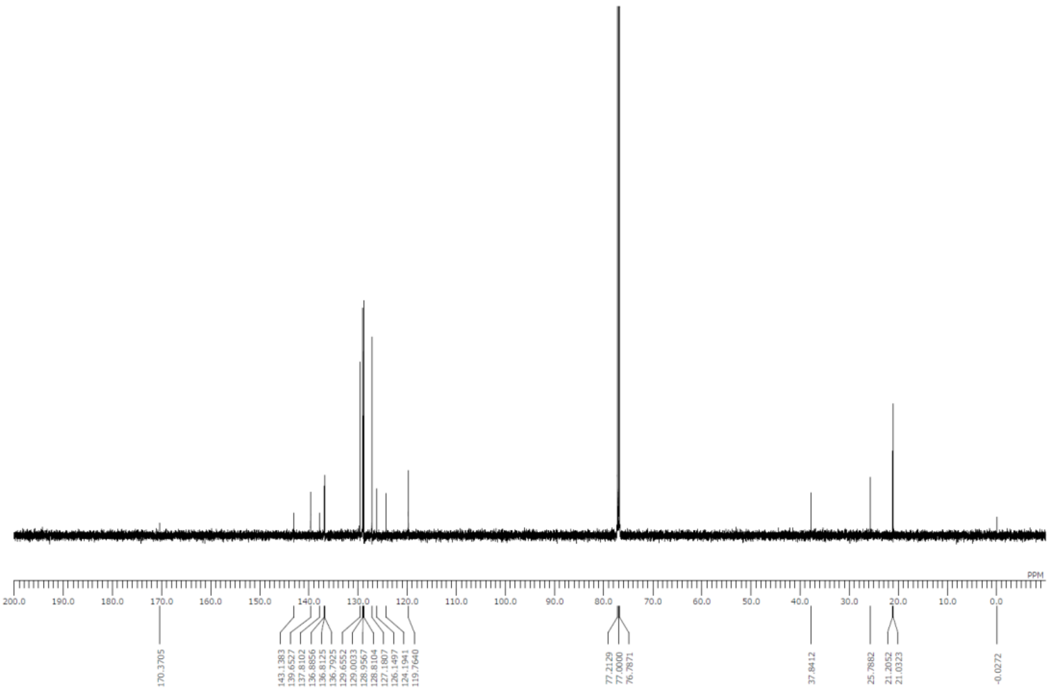

IR spectrum of **1c**

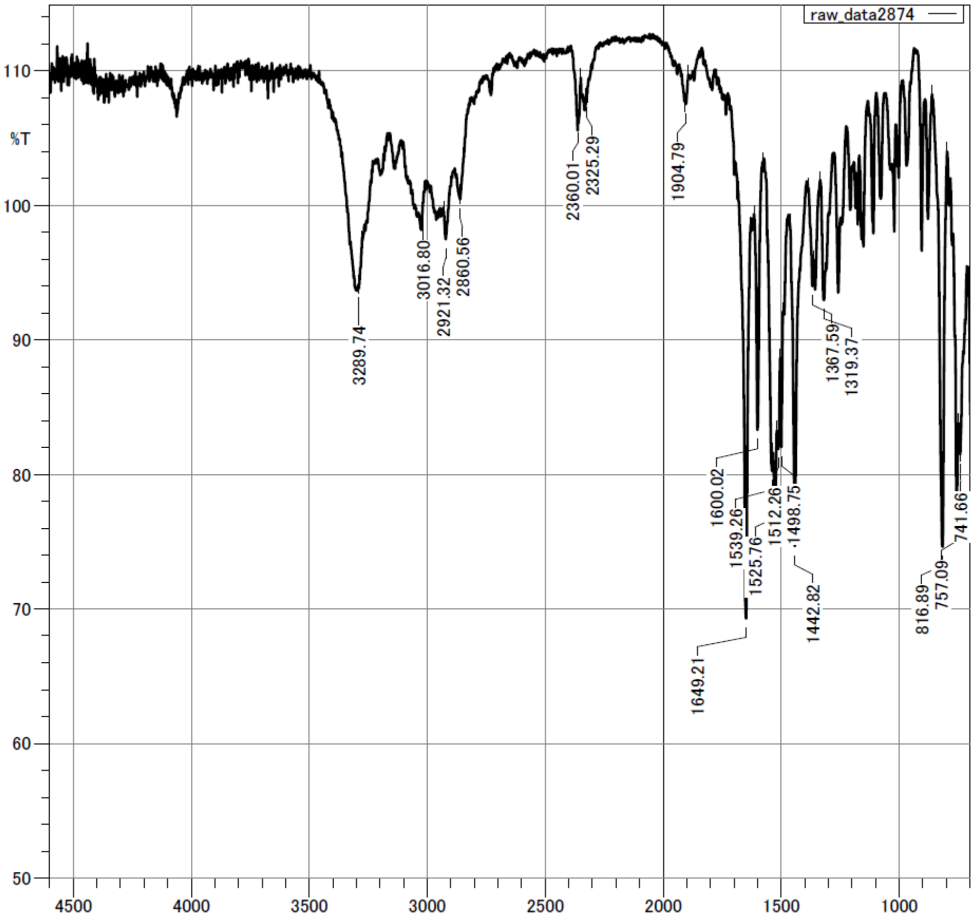

HRMS spectrum of **1c**

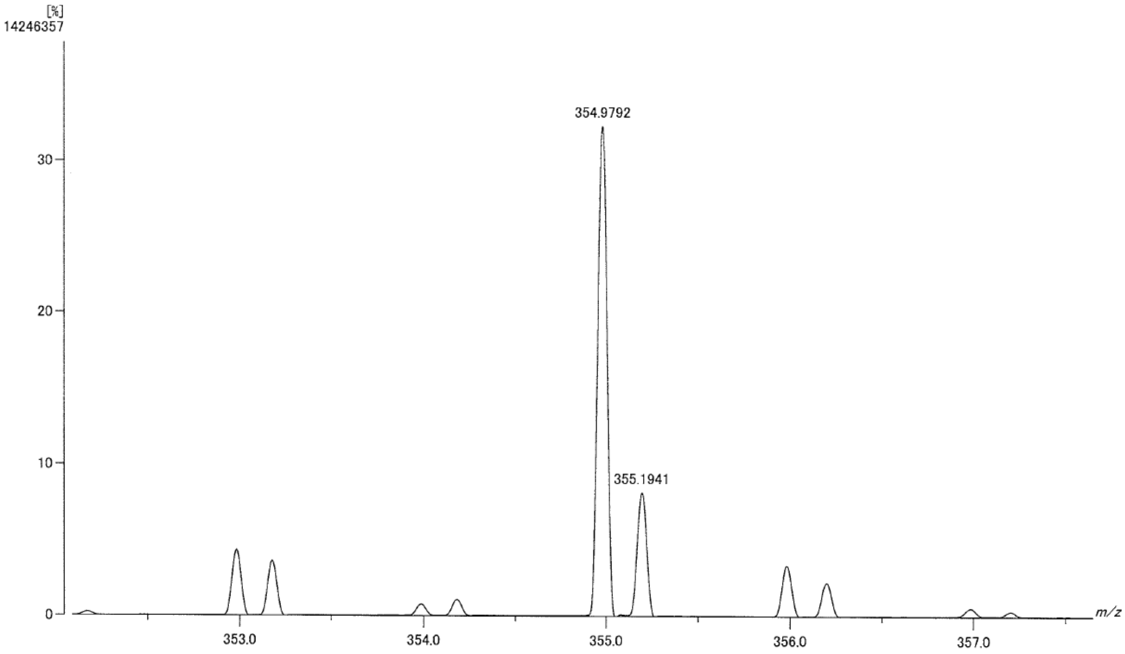

^{1}H-NMR spectrum of **1d** ($CDCl_3$, 400 MHz)

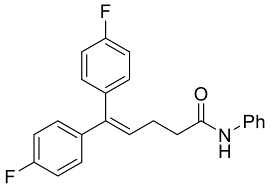

1d

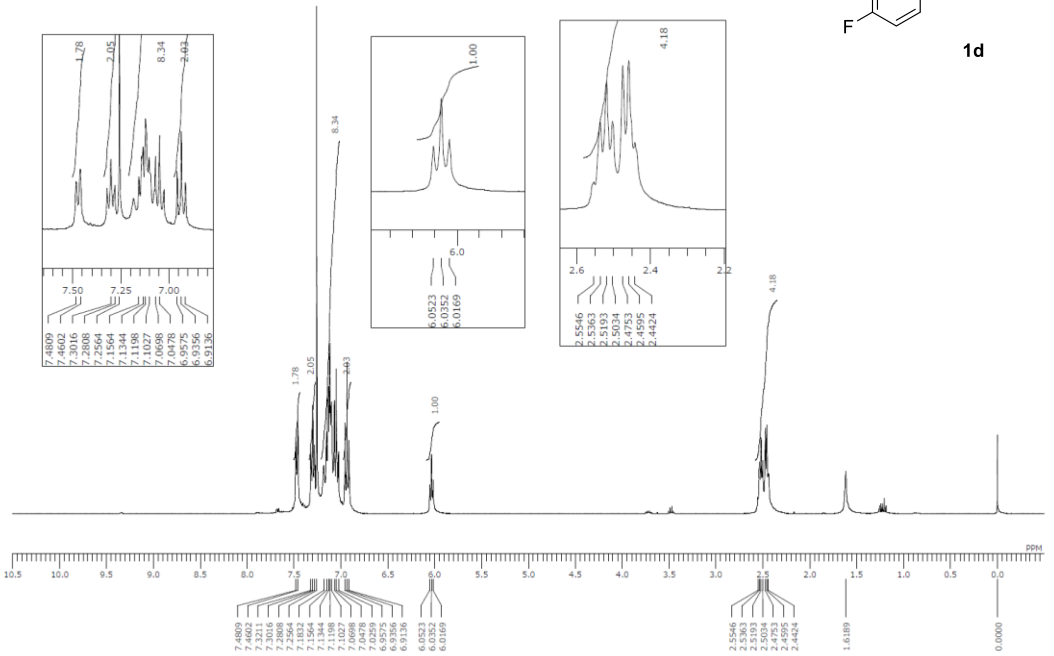

^{13}C-NMR spectrum of **1d** ($CDCl_3$, 100 MHz)

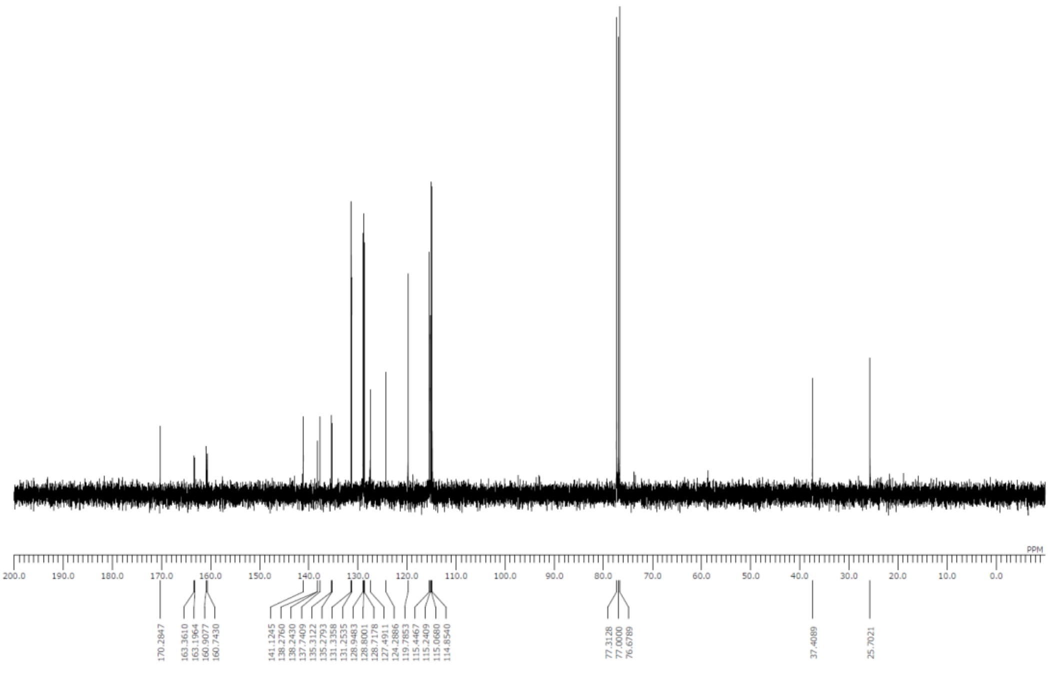

^{19}F-NMR spectrum of **1d** ($CDCl_3$, 565 MHz)

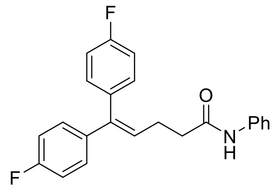

1d

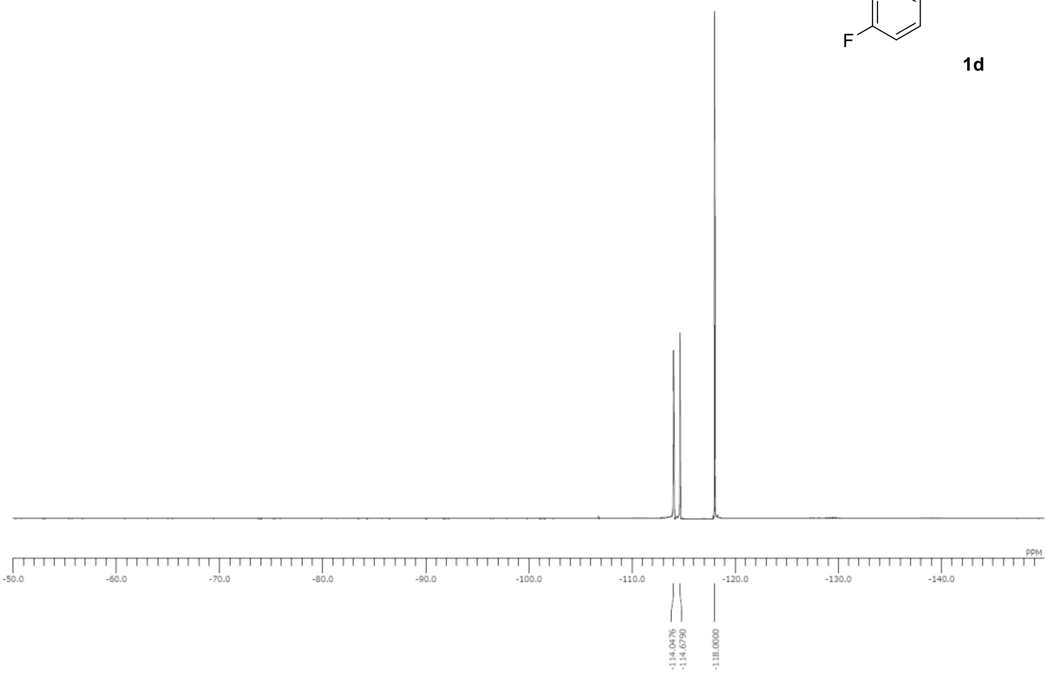

IR spectrum of **1d**

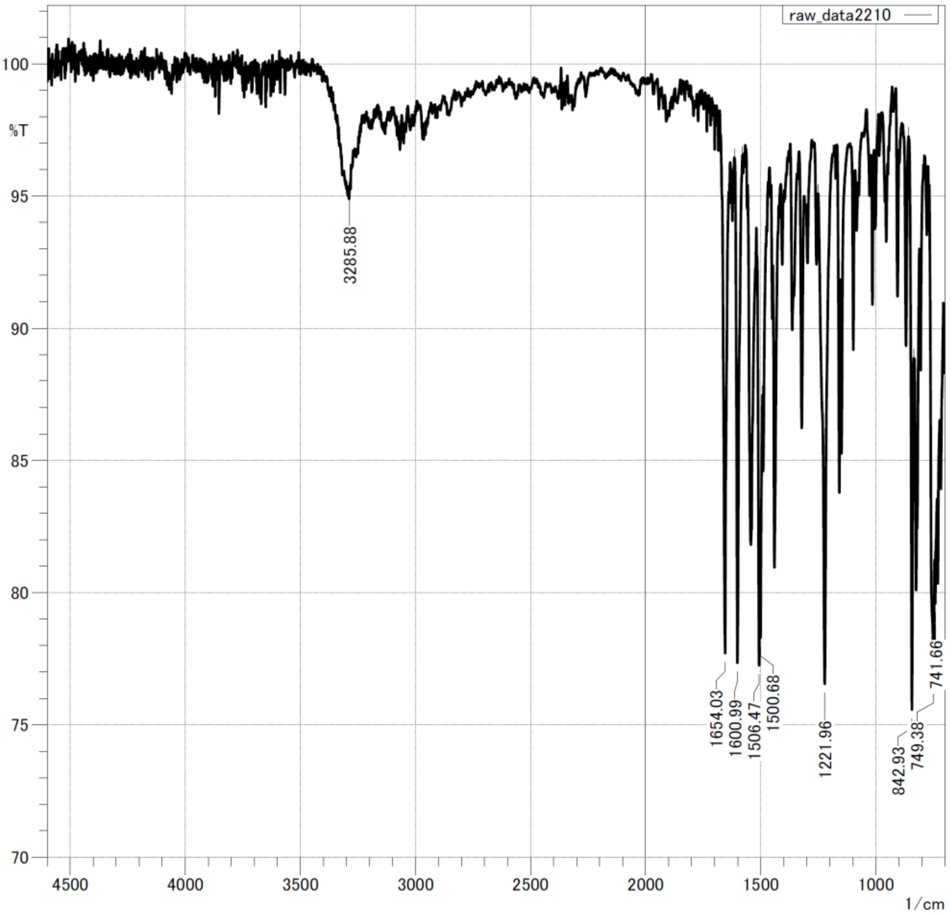

HRMS spectrum of **1d**

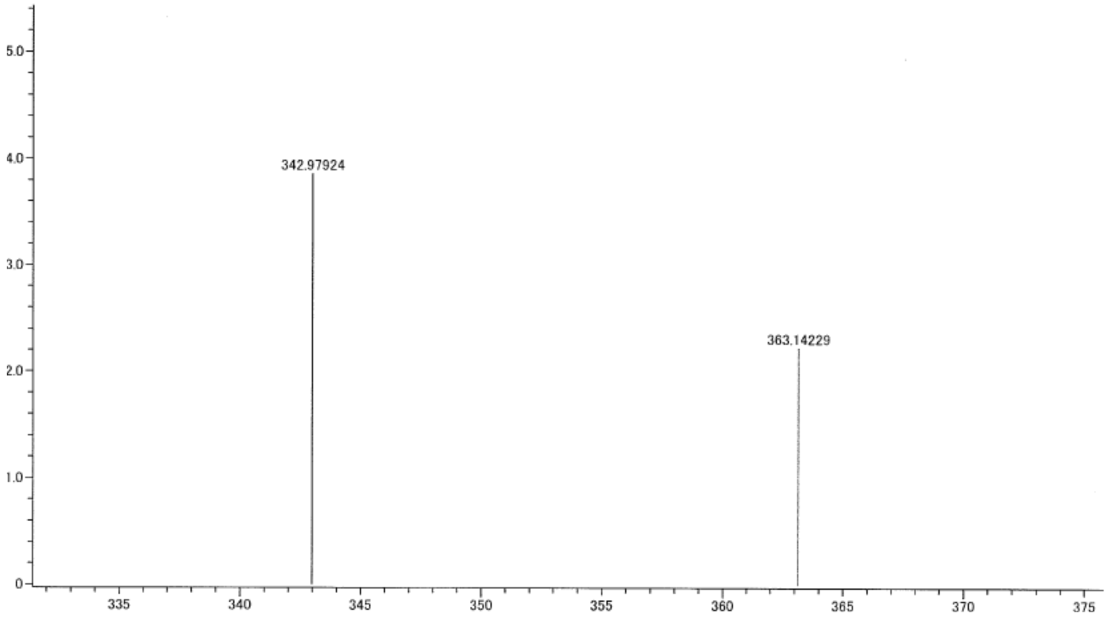

^{1}H-NMR spectrum of **1e** (DMSO-d_6, 600 MHz)

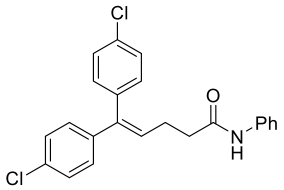

1e

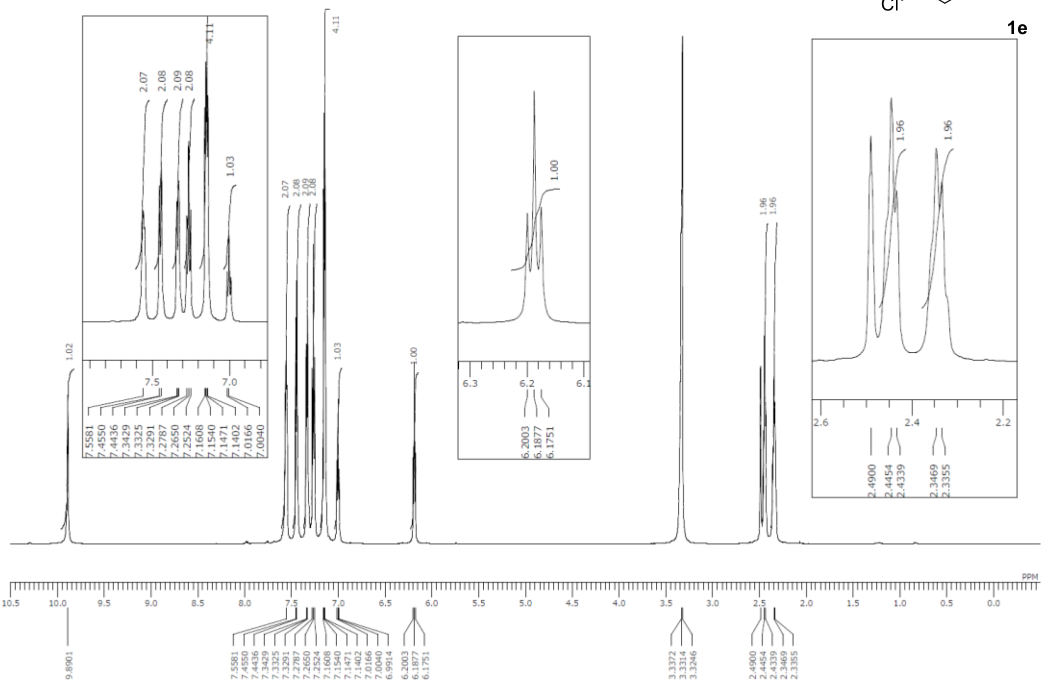

^{13}C-NMR spectrum of **1e** (DMSO-d_6, 150 MHz)

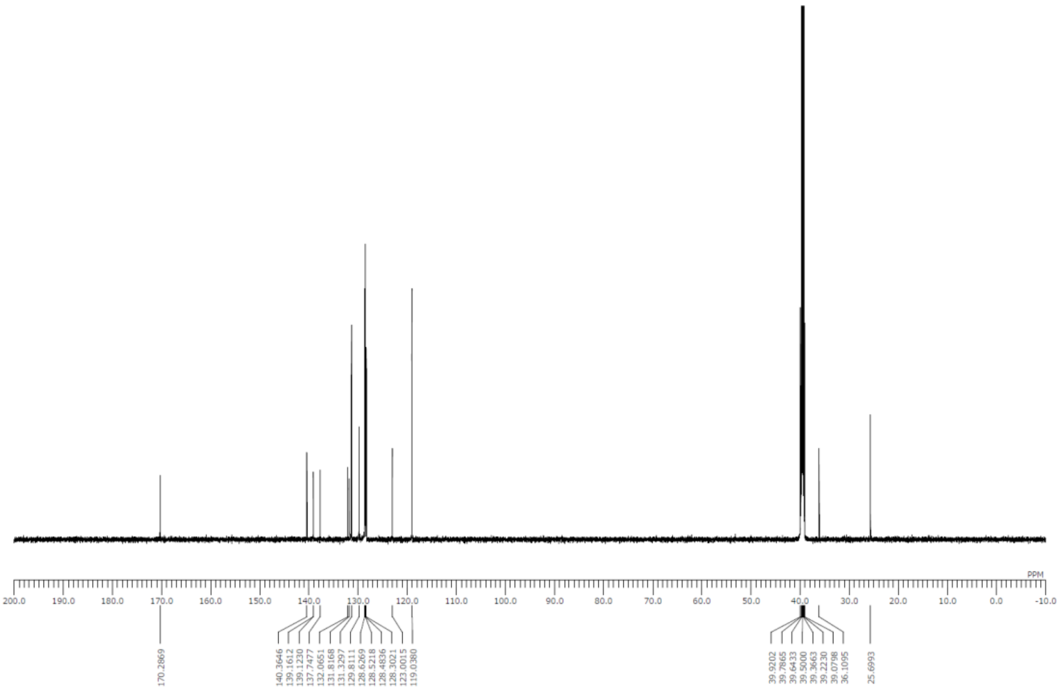

IR spectrum of **1e**

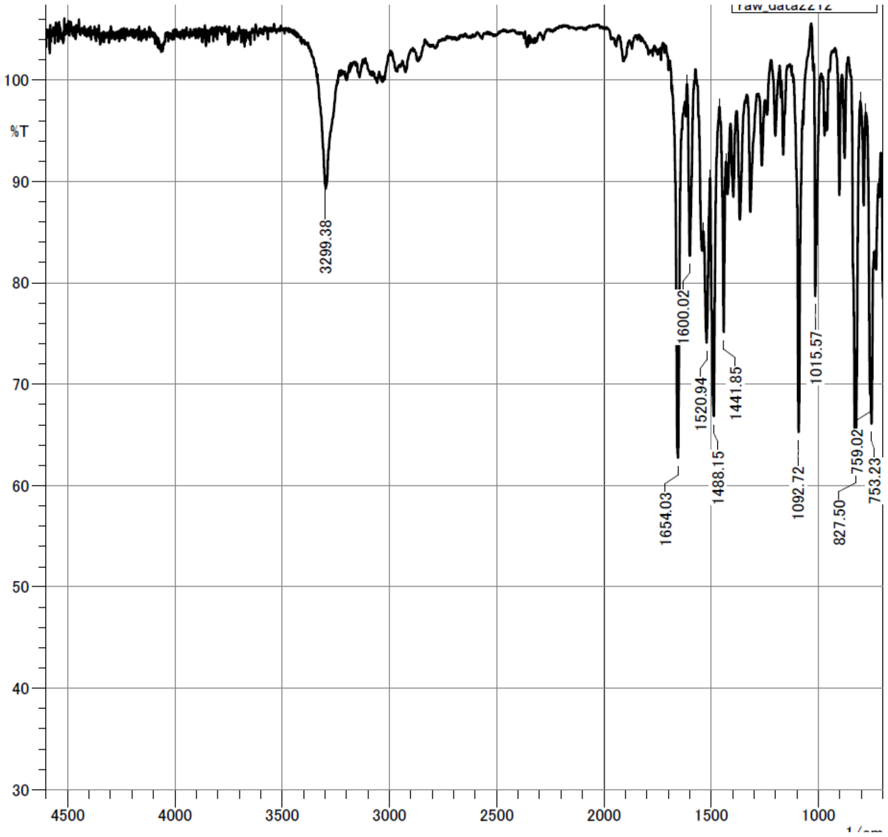

HRMS spectrum of **1e**

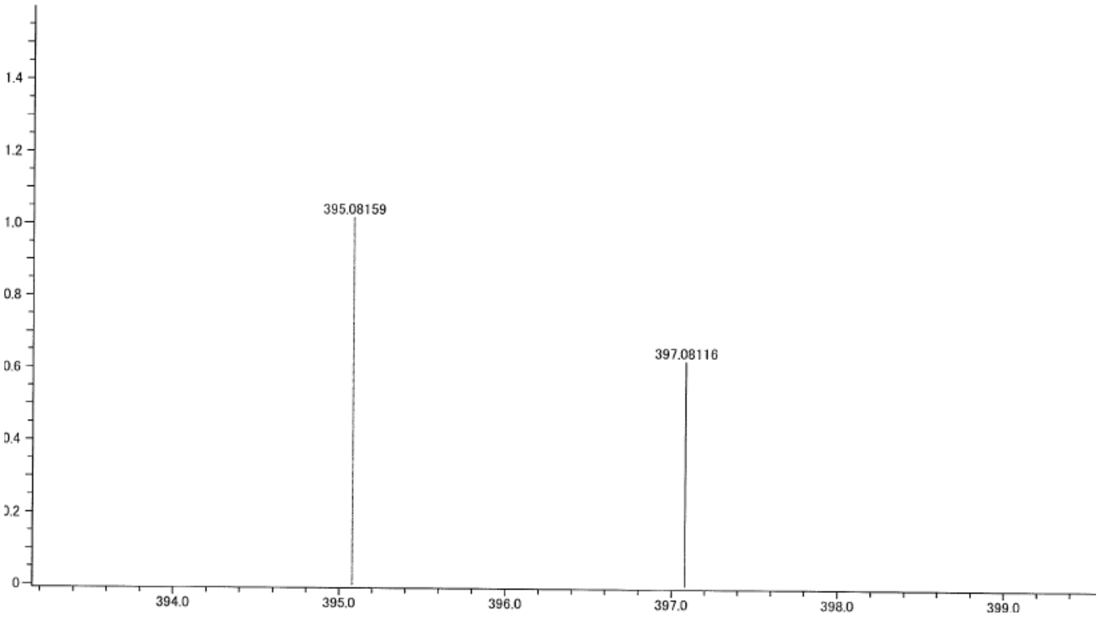

^{1}H-NMR spectrum of **1f** ($CDCl_3$, 600 MHz)

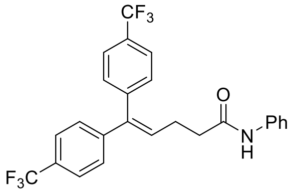

1f

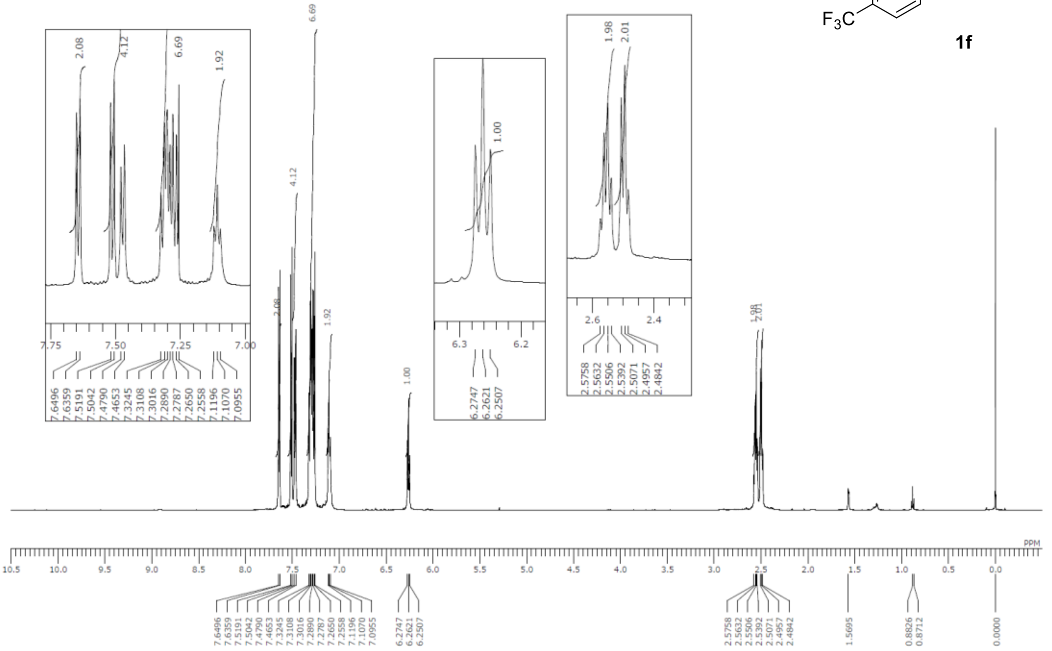

^{13}C-NMR spectrum of **1f** ($CDCl_3$, 150 MHz)

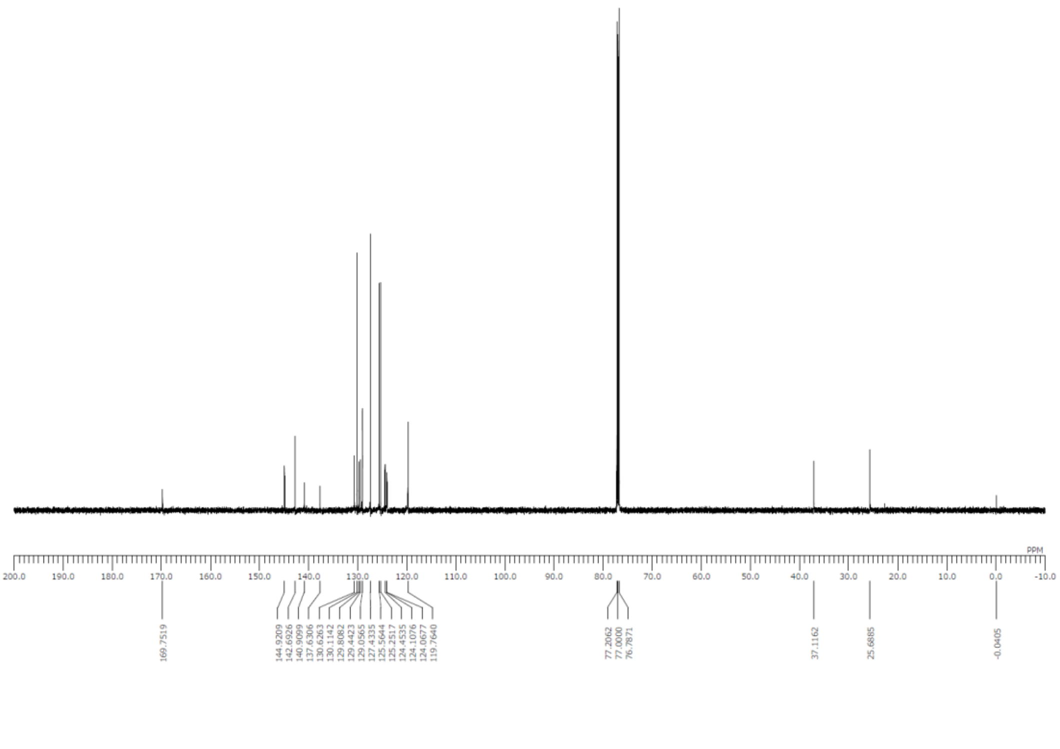

^{19}F-NMR spectrum of **1f** ($CDCl_3$, 565 MHz)

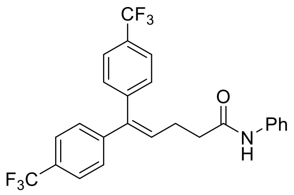

1f

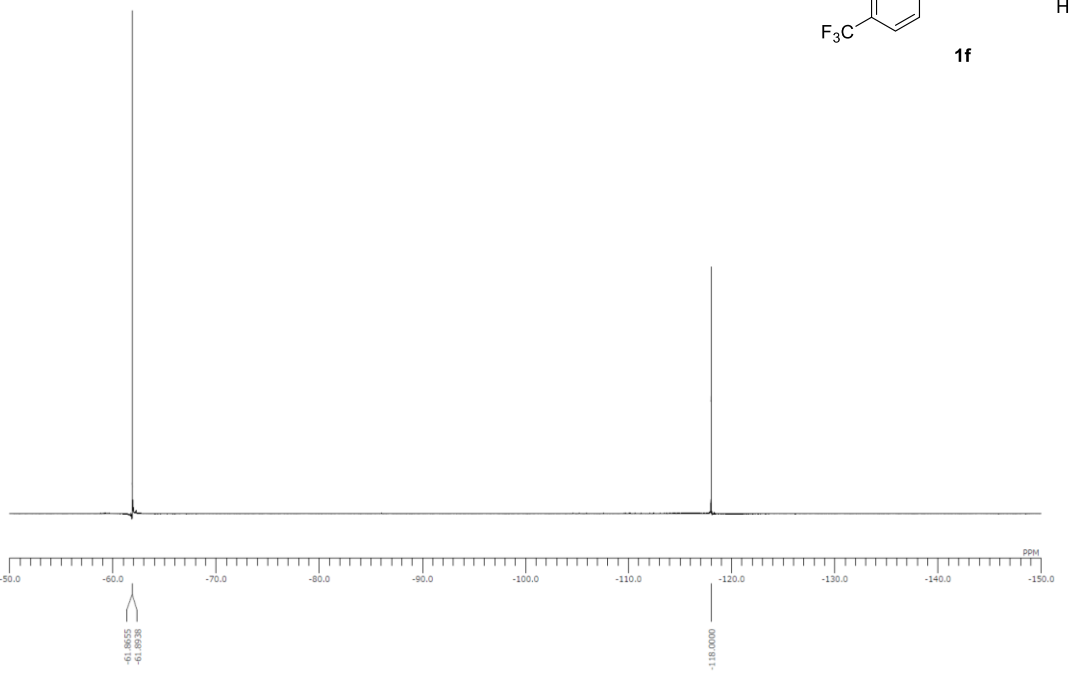

IR spectrum of **1f**

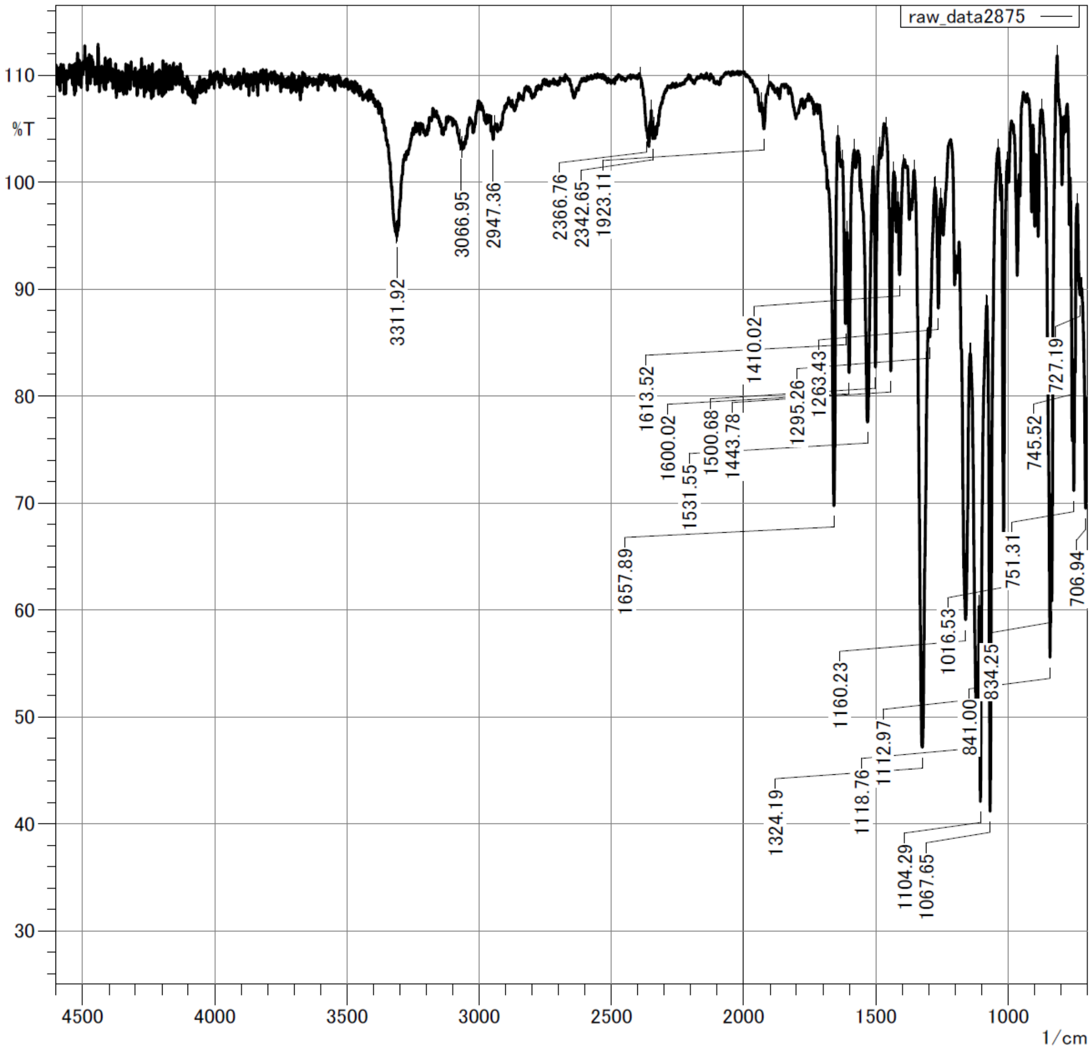

HRMS spectrum of **1f**

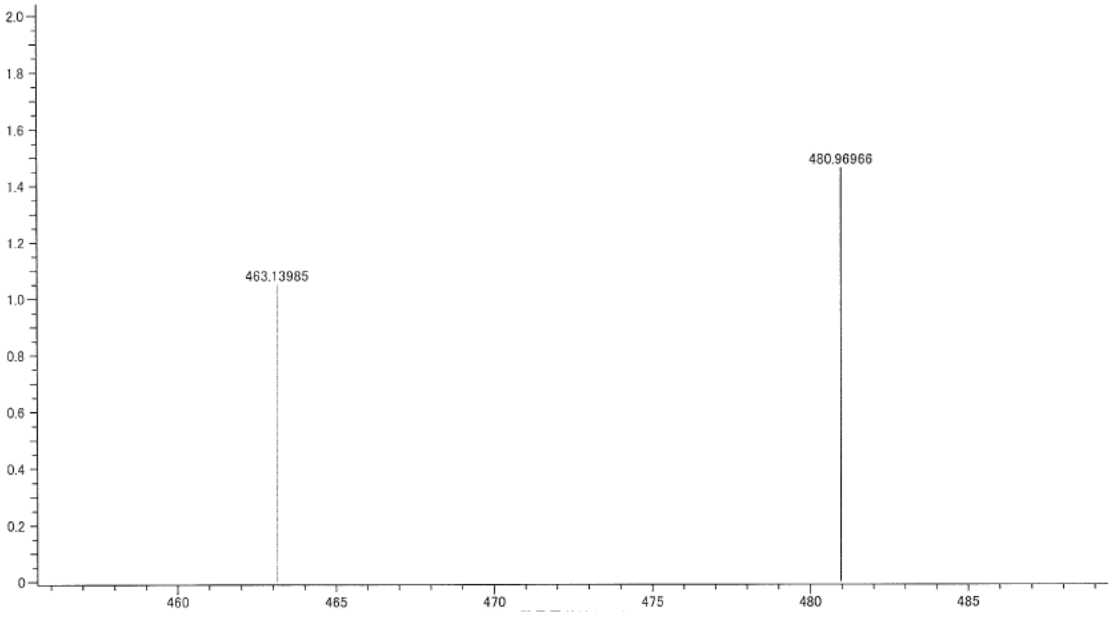

^{1}H-NMR spectrum of **1g** ($CDCl_3$, 600 MHz)

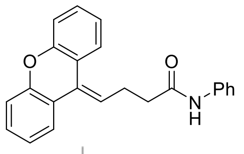

1g

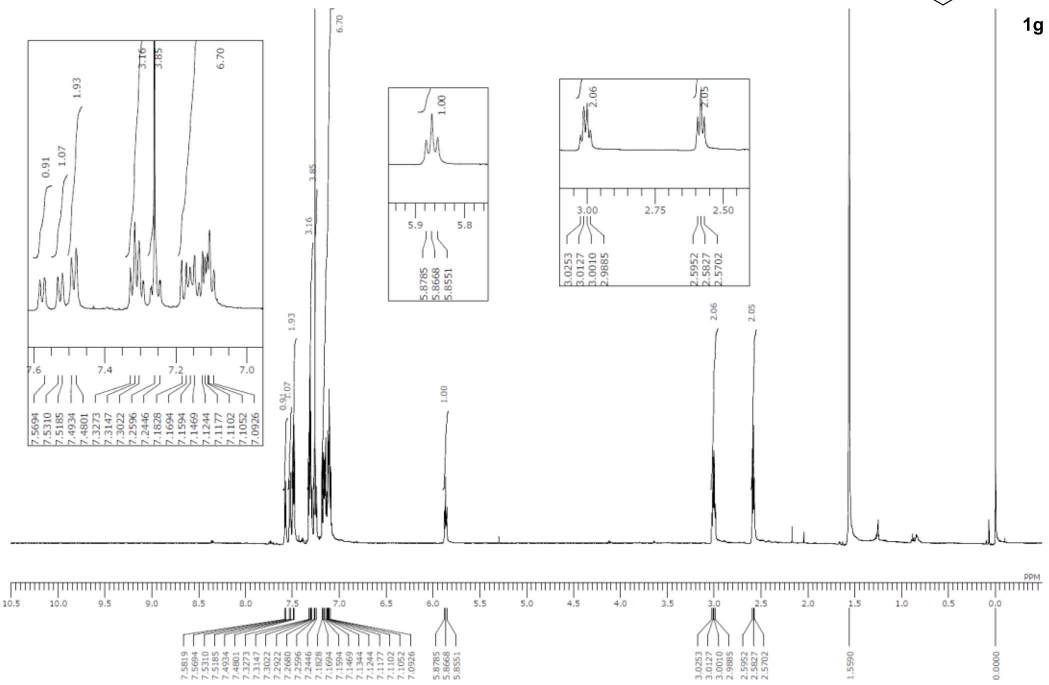

^{13}C-NMR spectrum of **1g** ($CDCl_3$, 150 MHz)

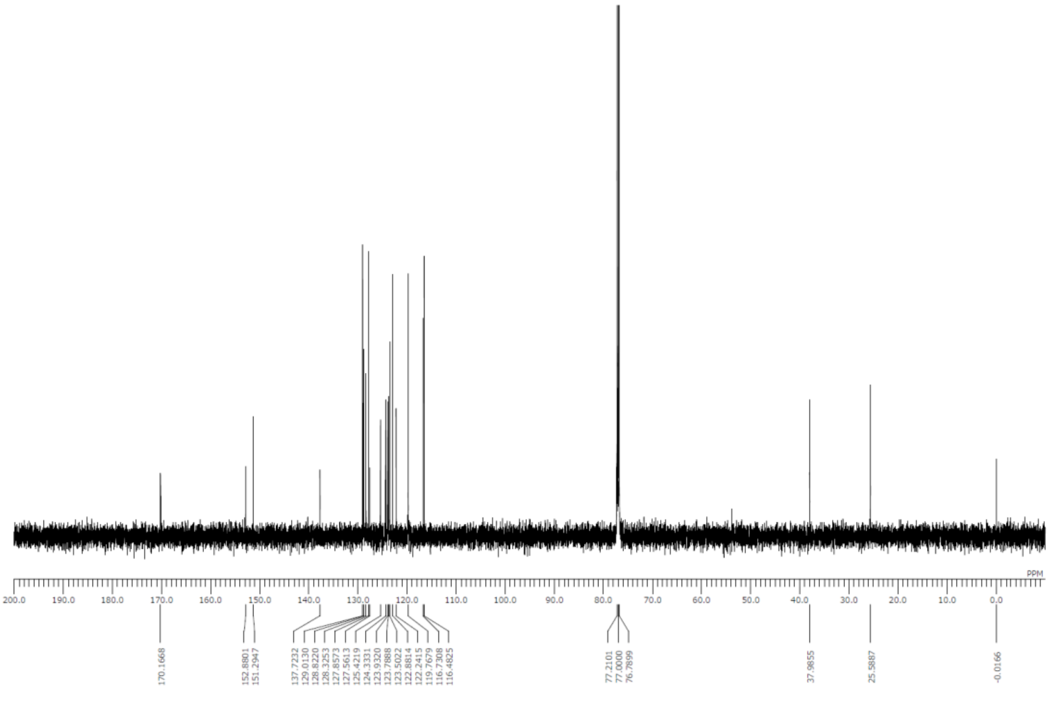

IR spectrum of **1g**

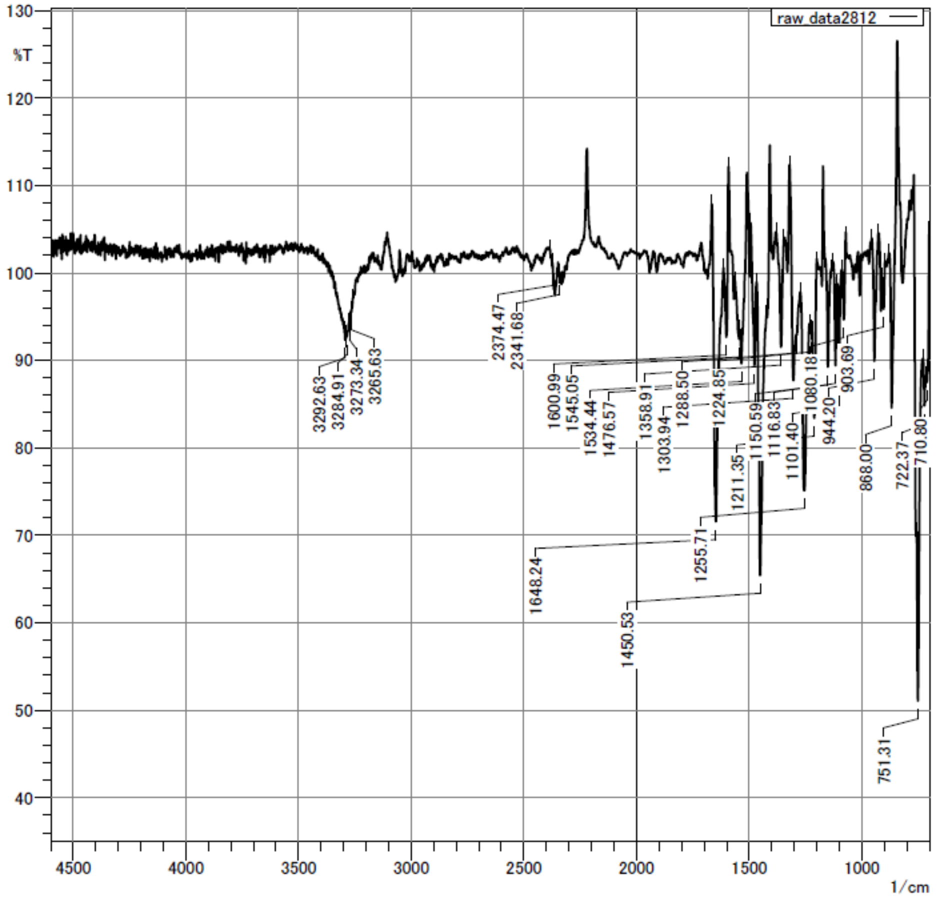

HRMS spectrum of **1g**

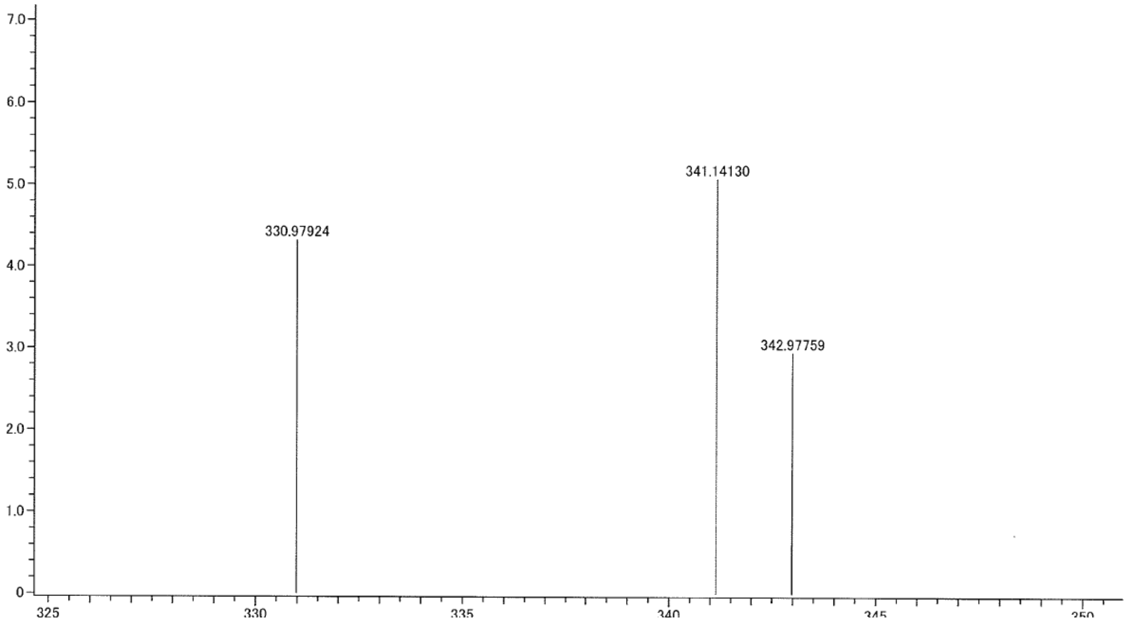

^{1}H-NMR spectrum of **1h** ($CDCl_3$, 600 MHz)

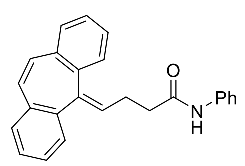

1h

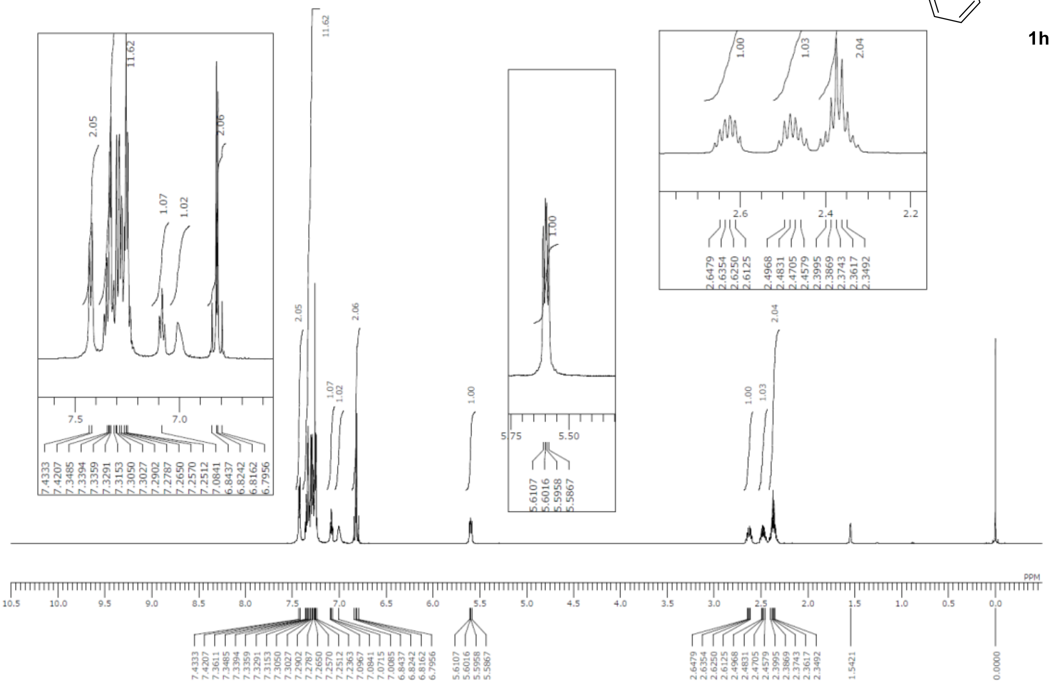

^{13}C-NMR spectrum of **1h** ($CDCl_3$, 150 MHz)

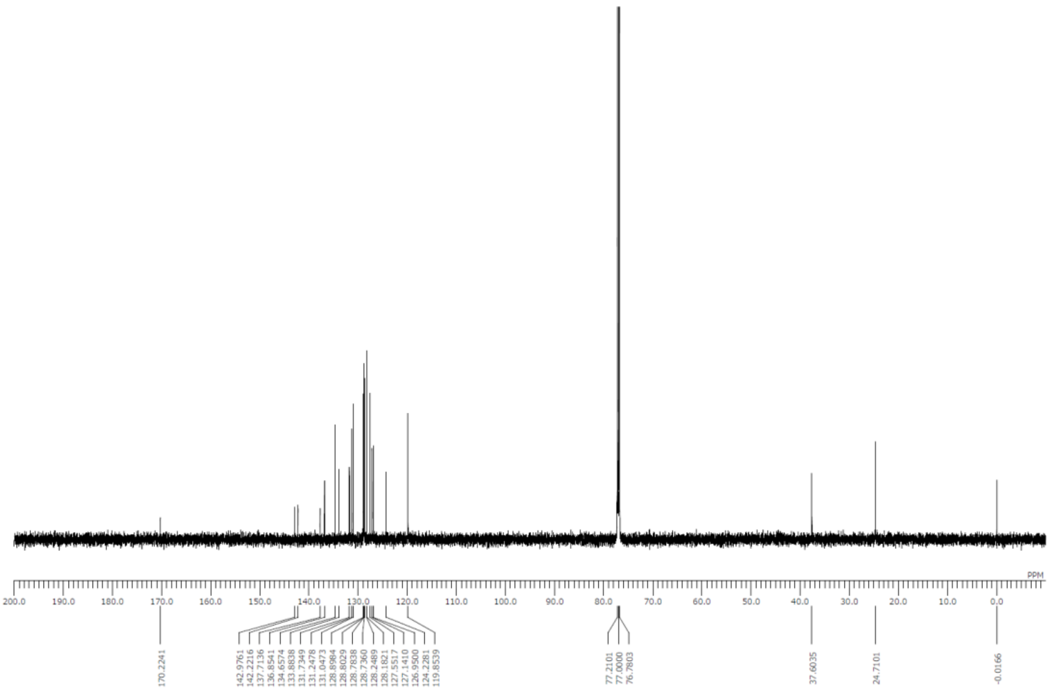

IR spectrum of **1h**

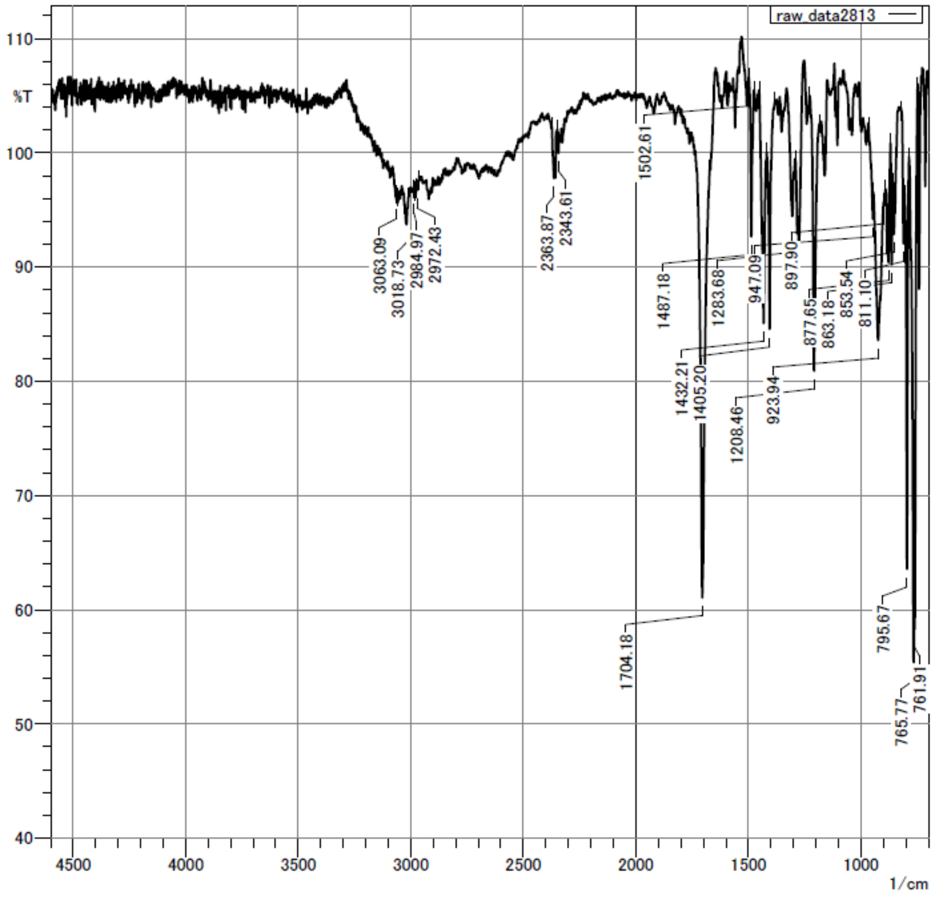

HRMS spectrum of **1h**

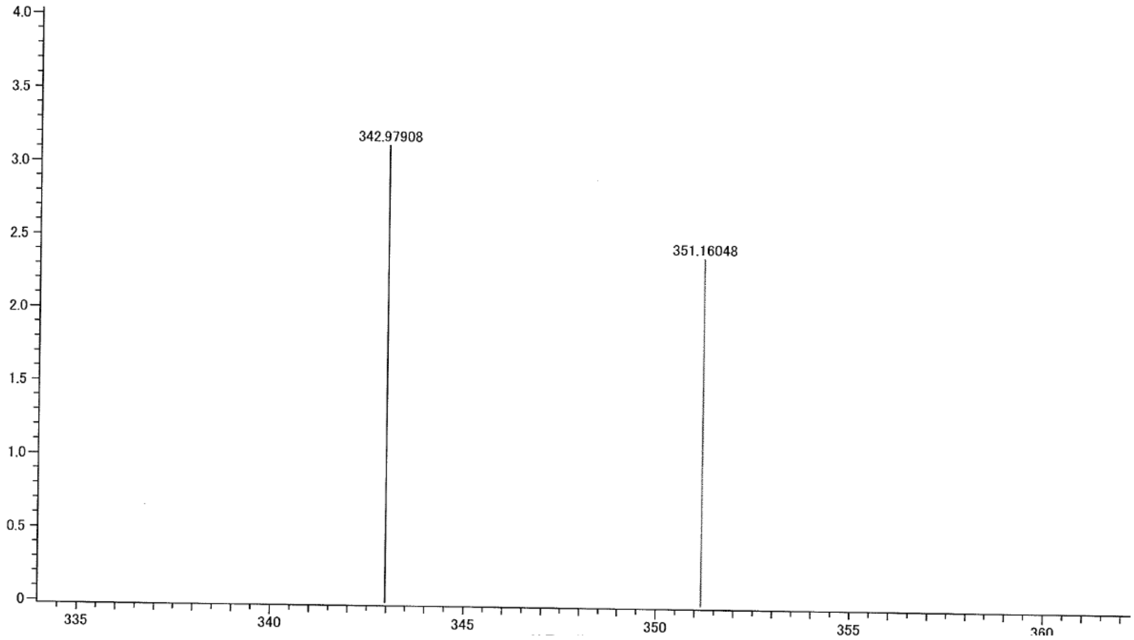

^{1}H-NMR spectrum of **1i** ($CDCl_3$, 600 MHz)

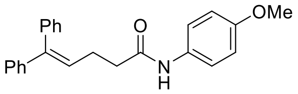

1i

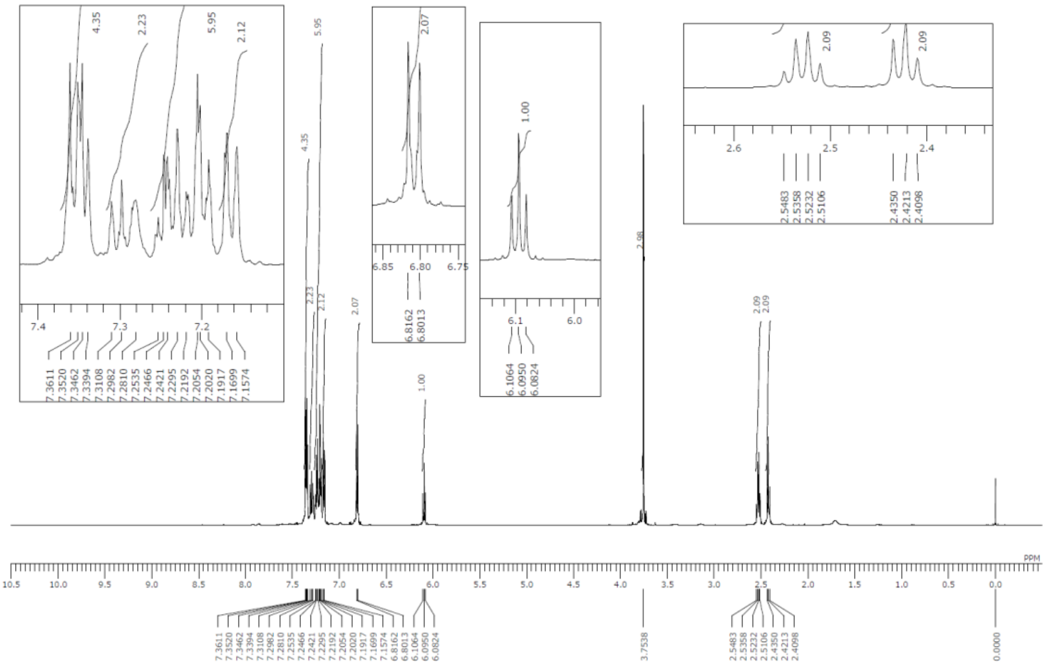

^{13}C-NMR spectrum of **1i** ($CDCl_3$, 150 MHz)

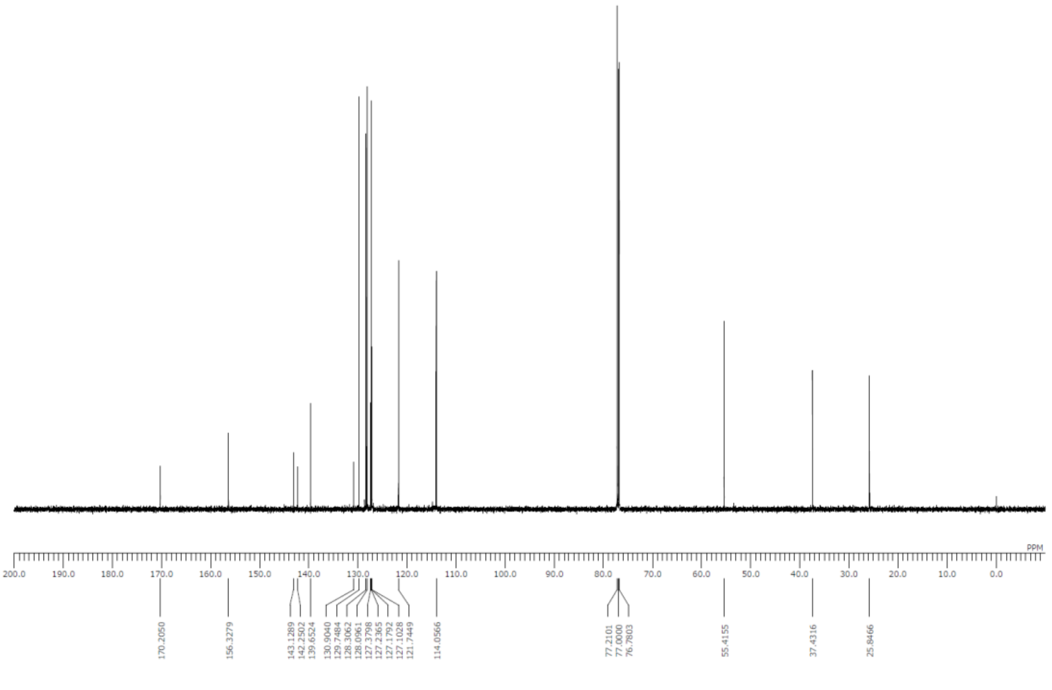

IR spectrum of **1i**

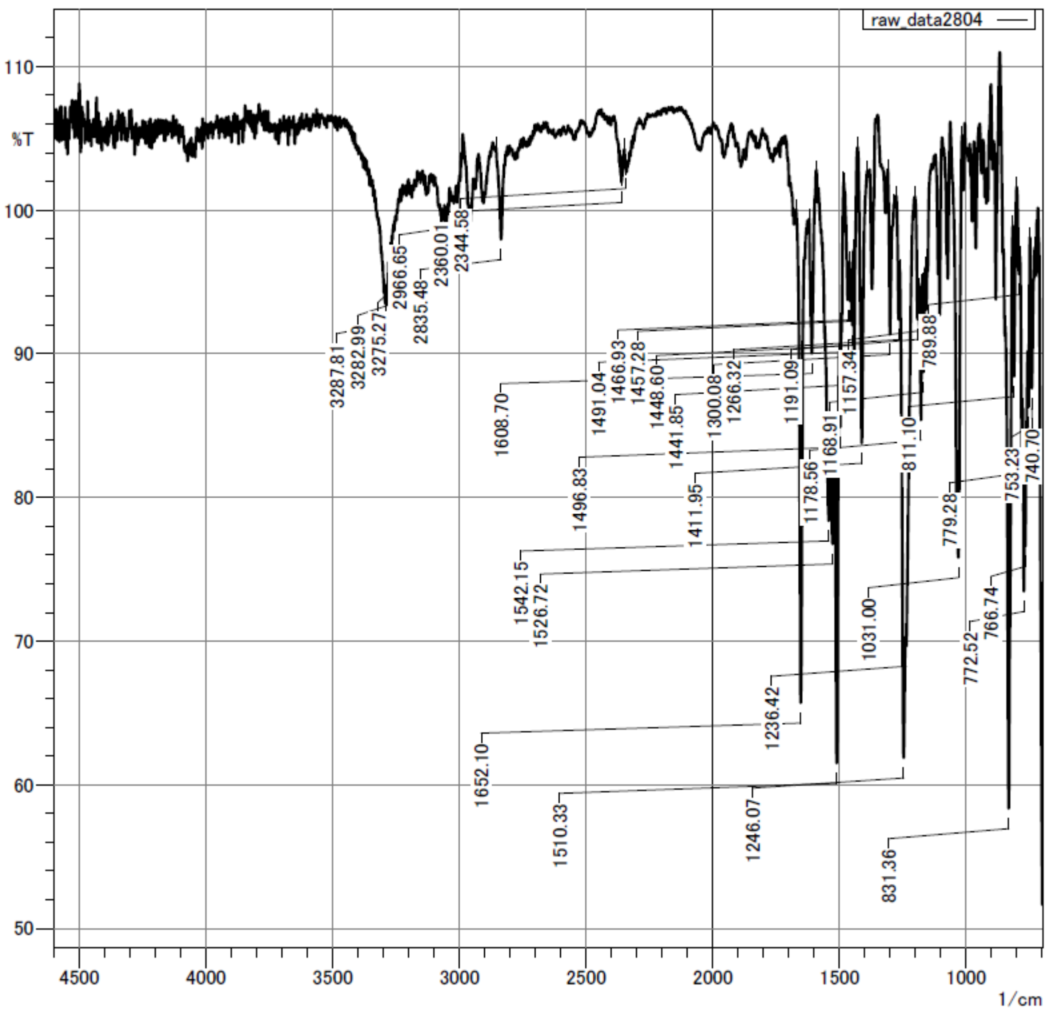

HRMS spectrum of **1i**

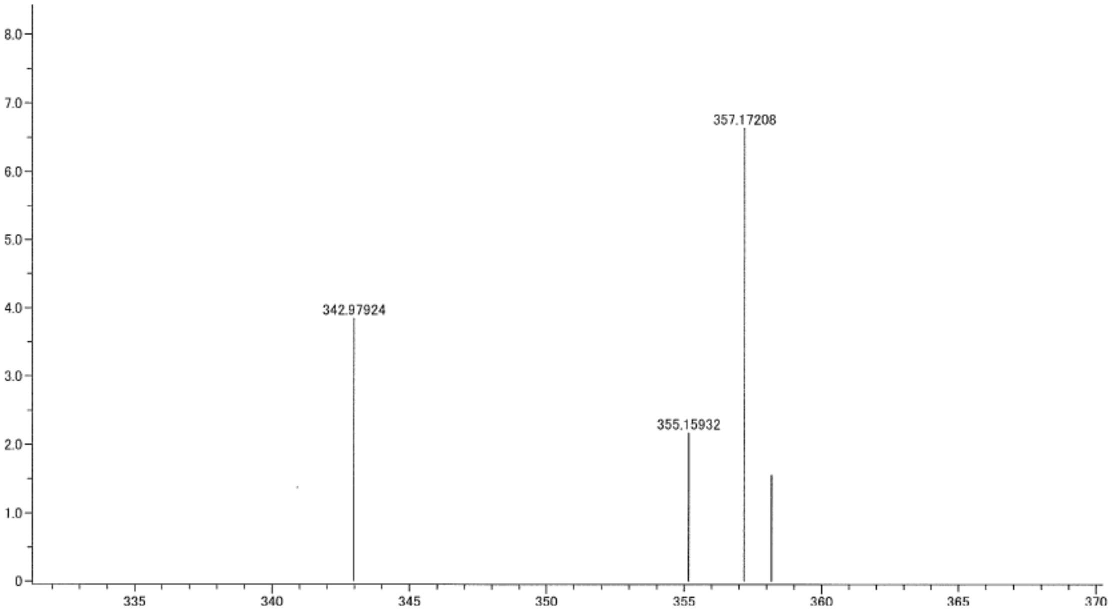

^{1}H-NMR spectrum of **1j** ($CDCl_3$, 600 MHz)

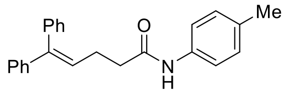

1j

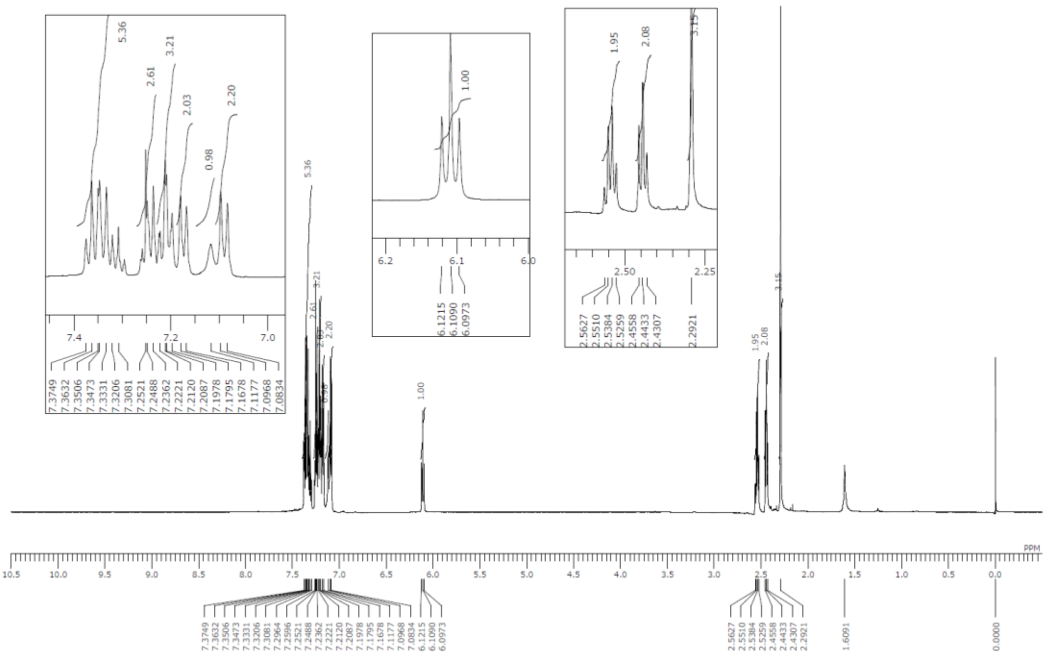

^{13}C-NMR spectrum of **1j** ($CDCl_3$, 150 MHz)

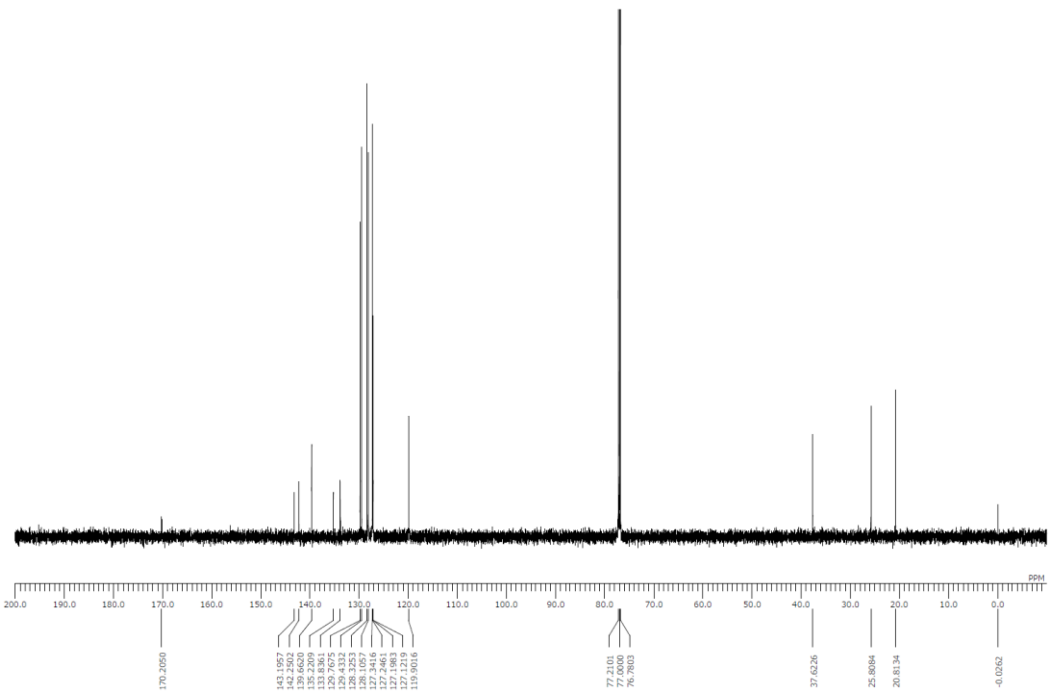

IR spectrum of **1j**

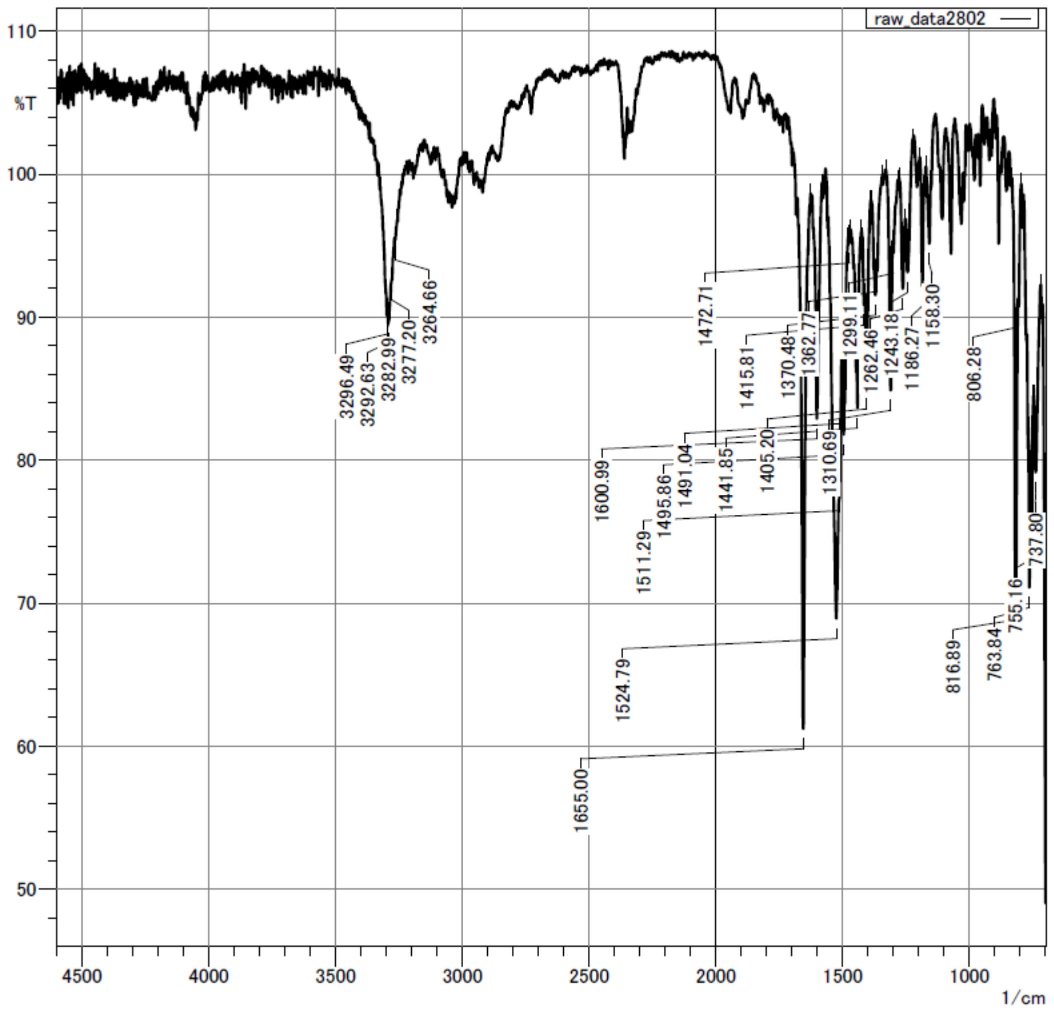

HRMS spectrum of **1j**

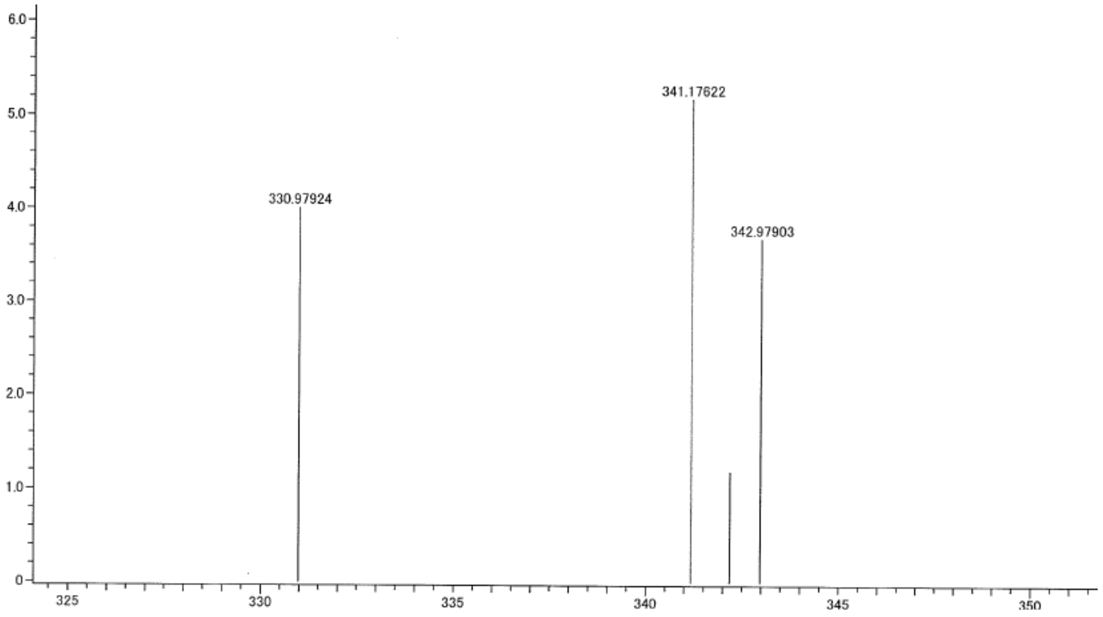

^{1}H-NMR spectrum of **1k** ($CDCl_3$, 600 MHz)

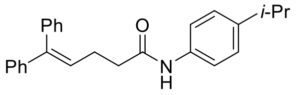

1k

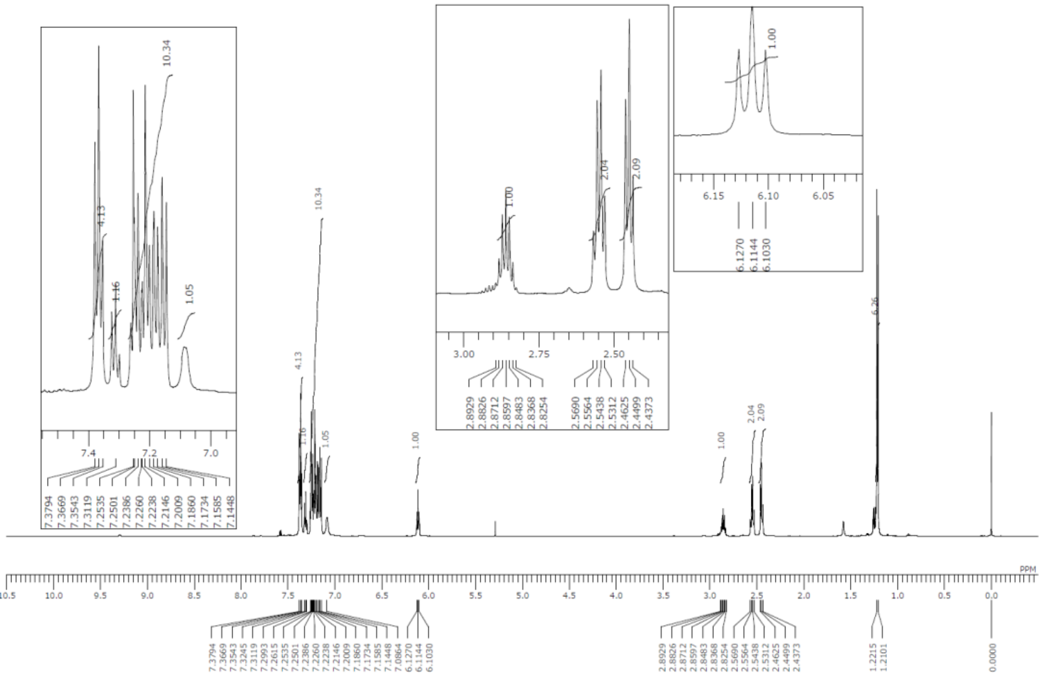

^{13}C-NMR spectrum of **1k** ($CDCl_3$, 150 MHz)

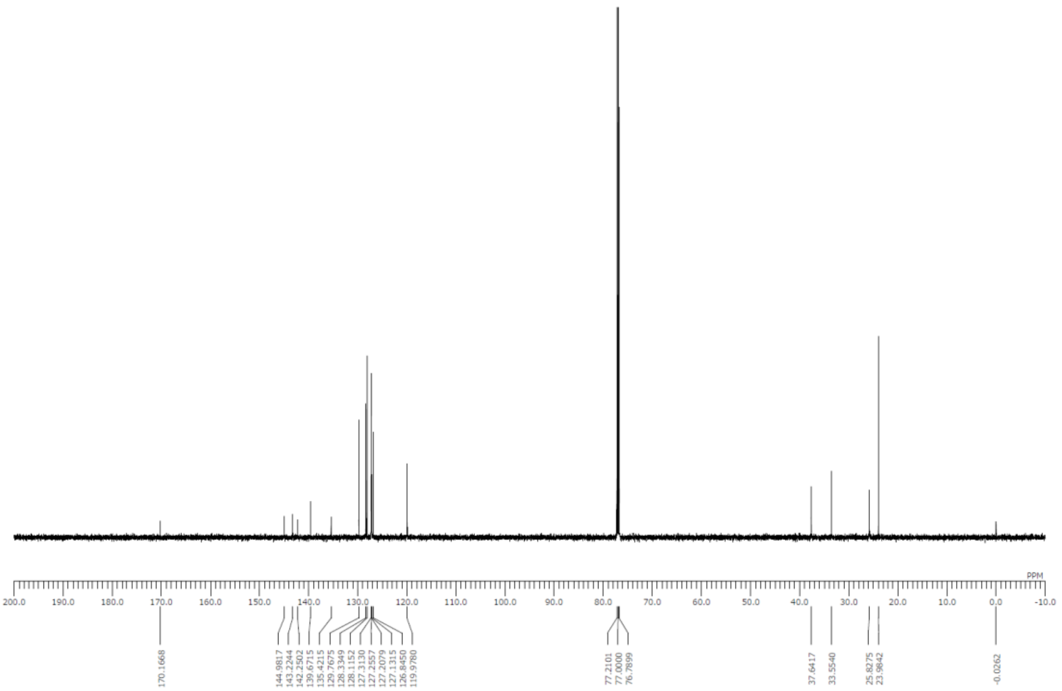

IR spectrum of **1k**

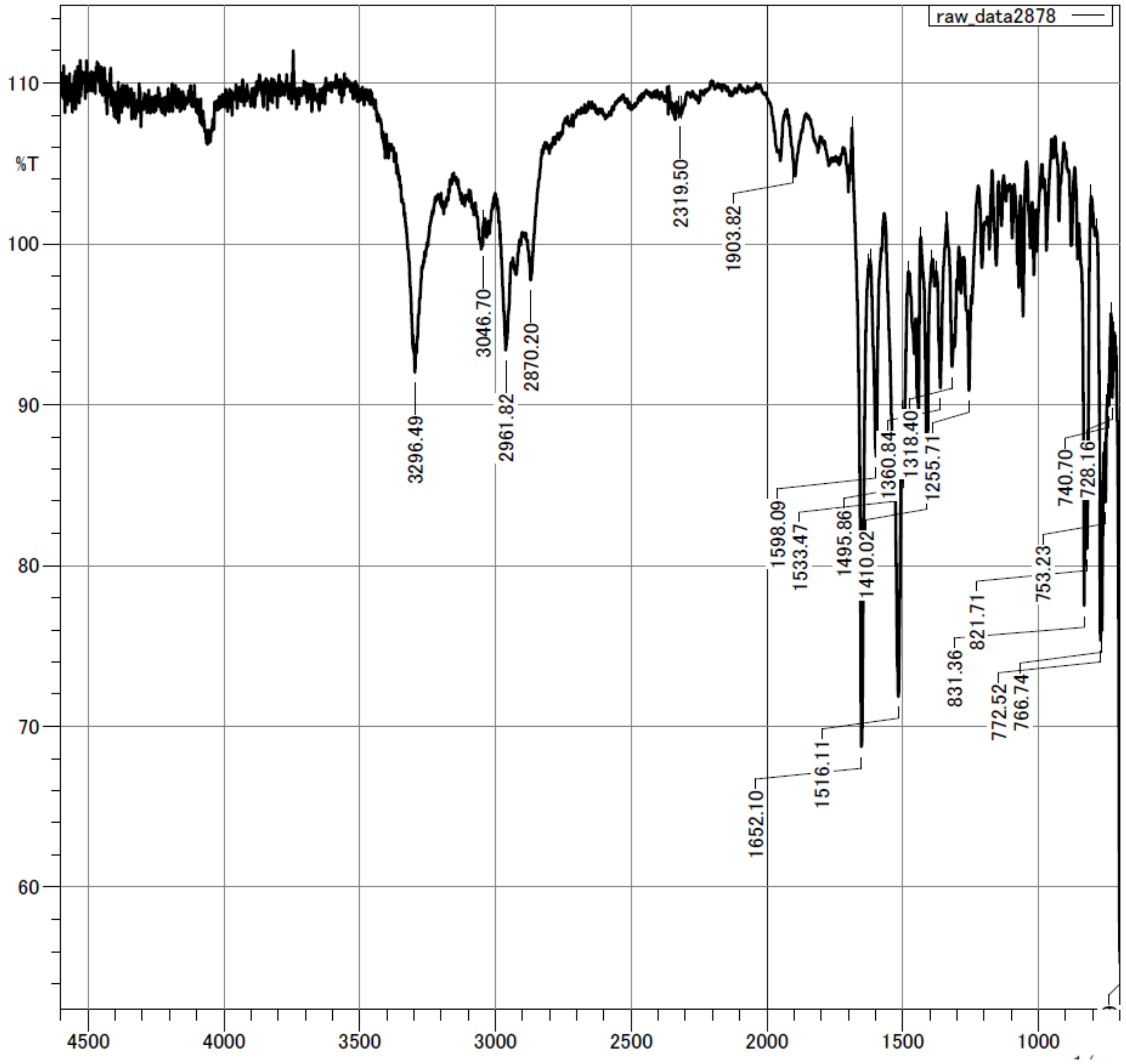

HRMS spectrum of **1k**

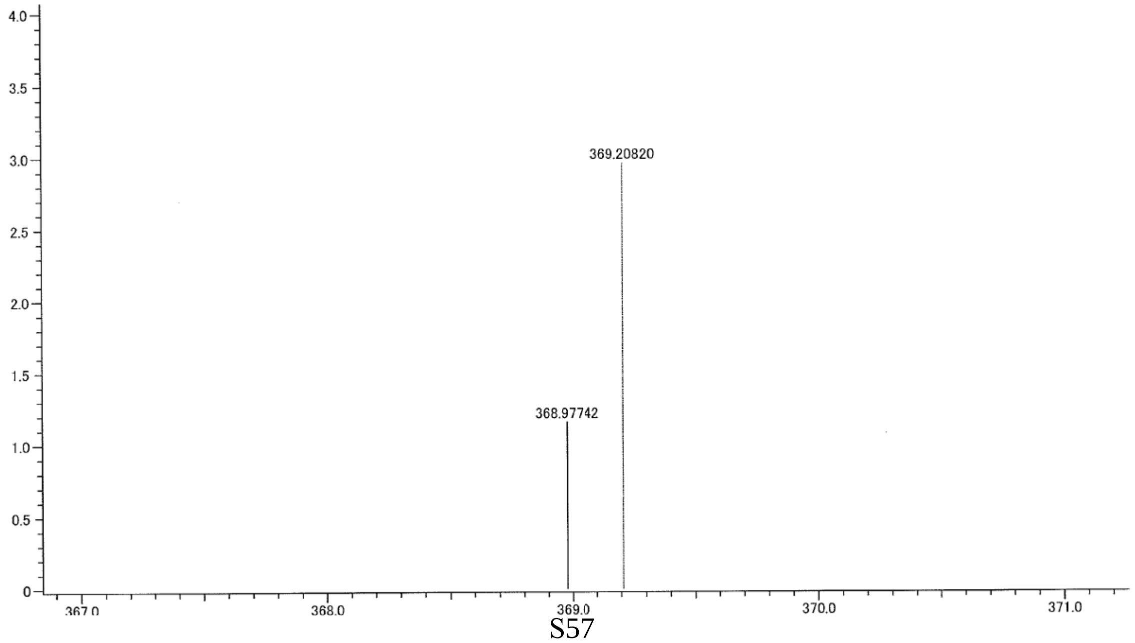

^{1}H-NMR spectrum of **1l** ($CDCl_3$, 600 MHz)

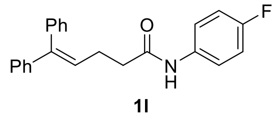

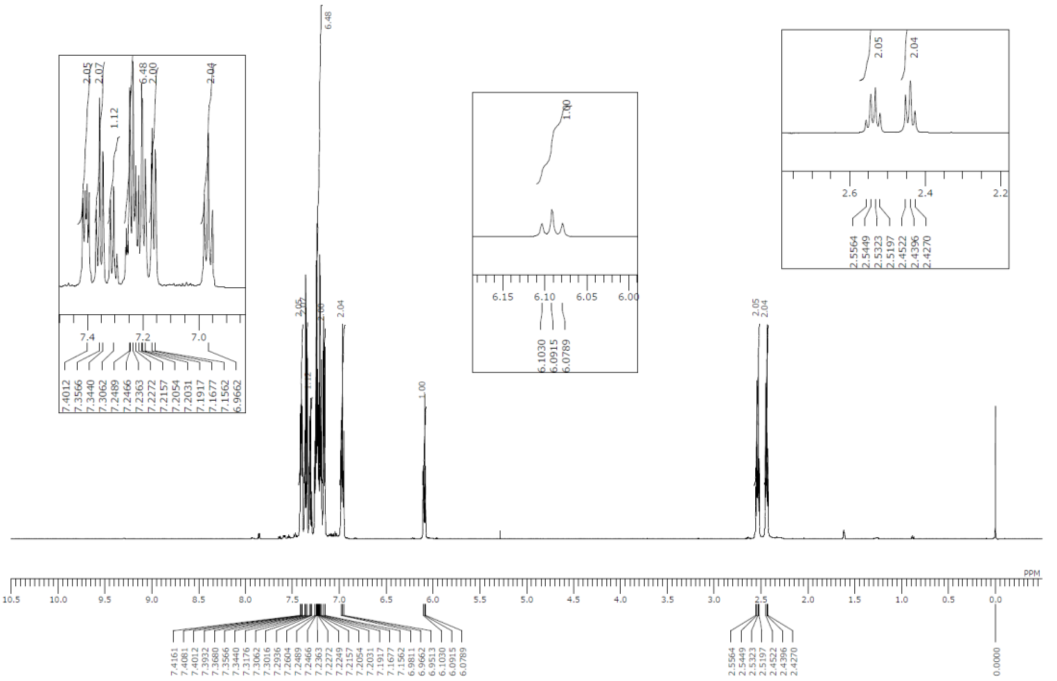

^{13}C-NMR spectrum of **1l** ($CDCl_3$, 150 MHz)

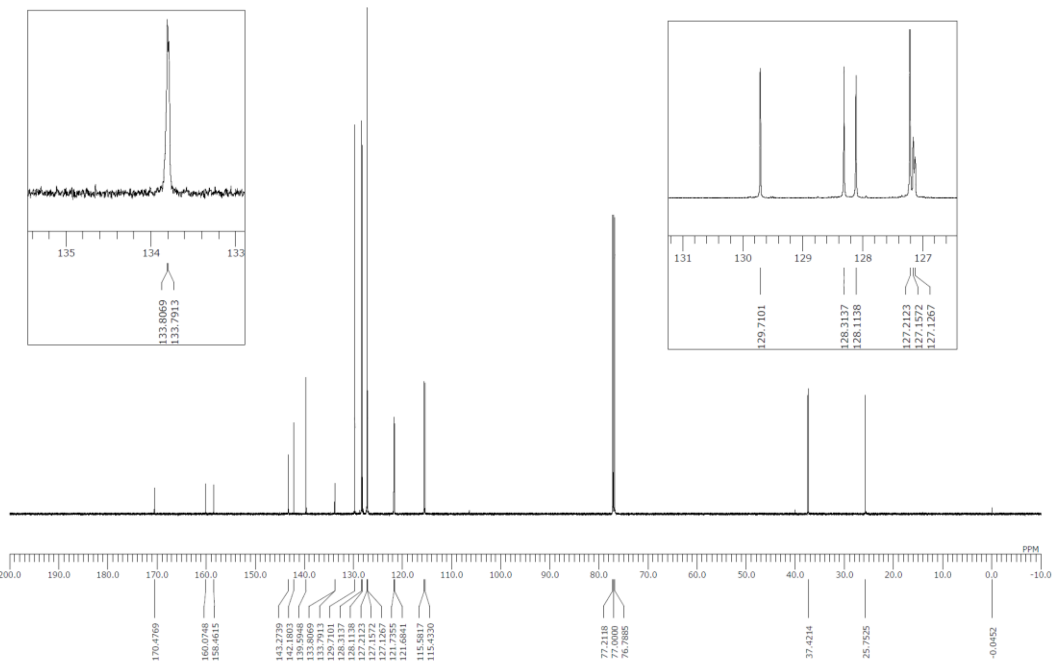

^{19}F-NMR spectrum of **1l** ($CDCl_3$, 565 MHz)

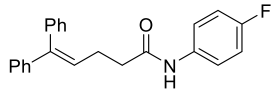

1l

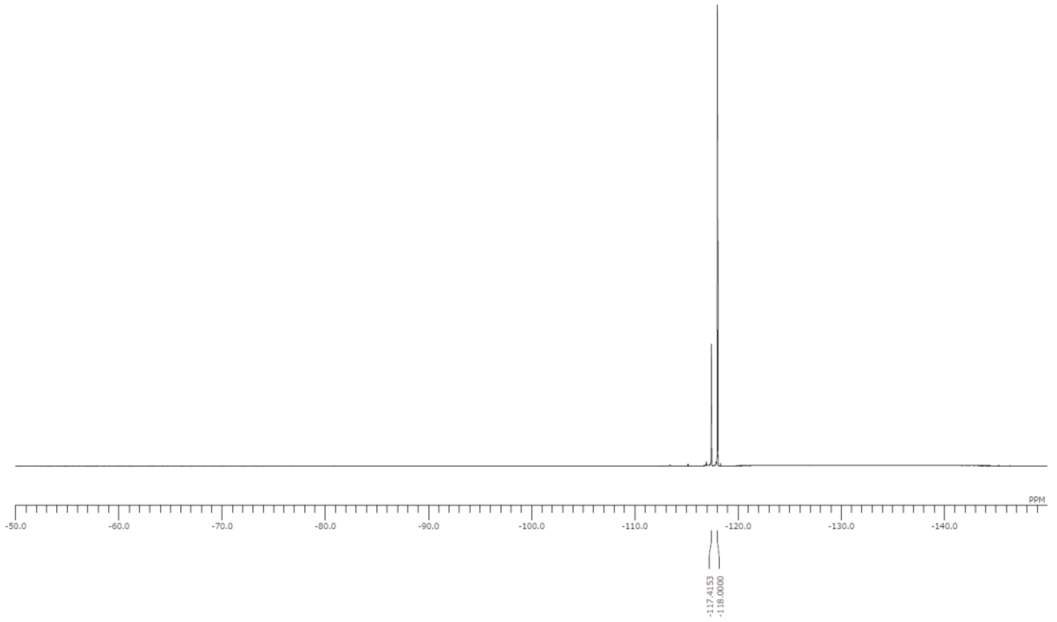

IR spectrum of **1l**

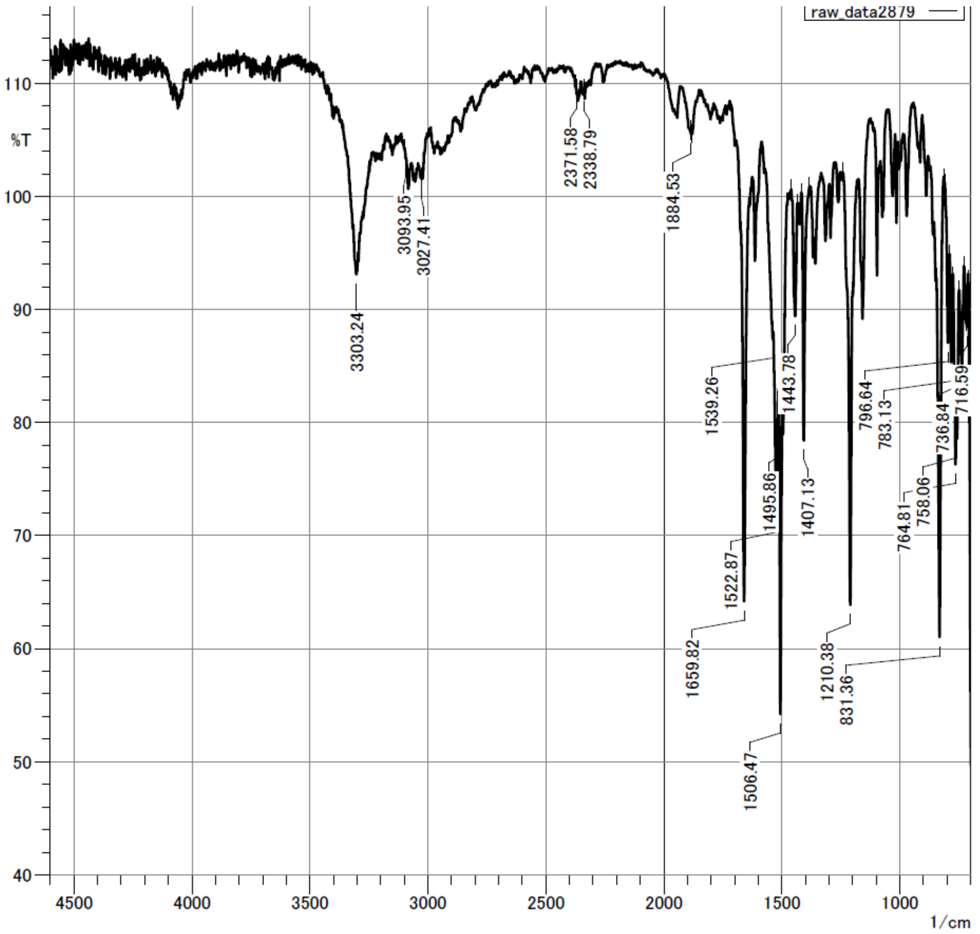

HRMS spectrum of **1l**

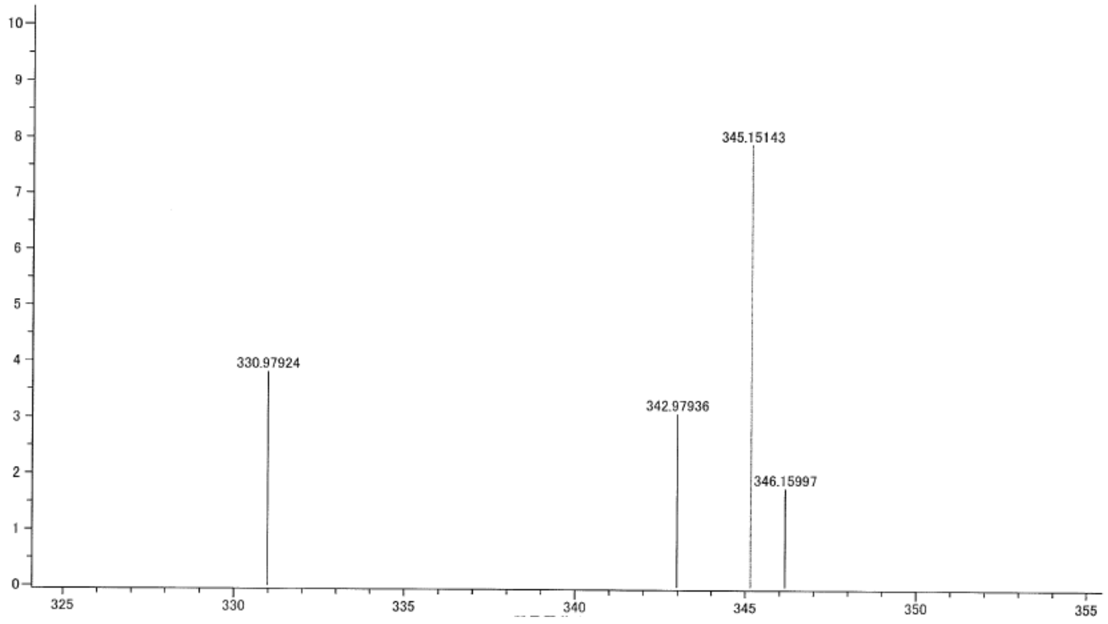

^{1}H-NMR spectrum of **1m** ($CDCl_3$, 600 MHz)

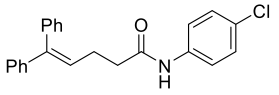

1m

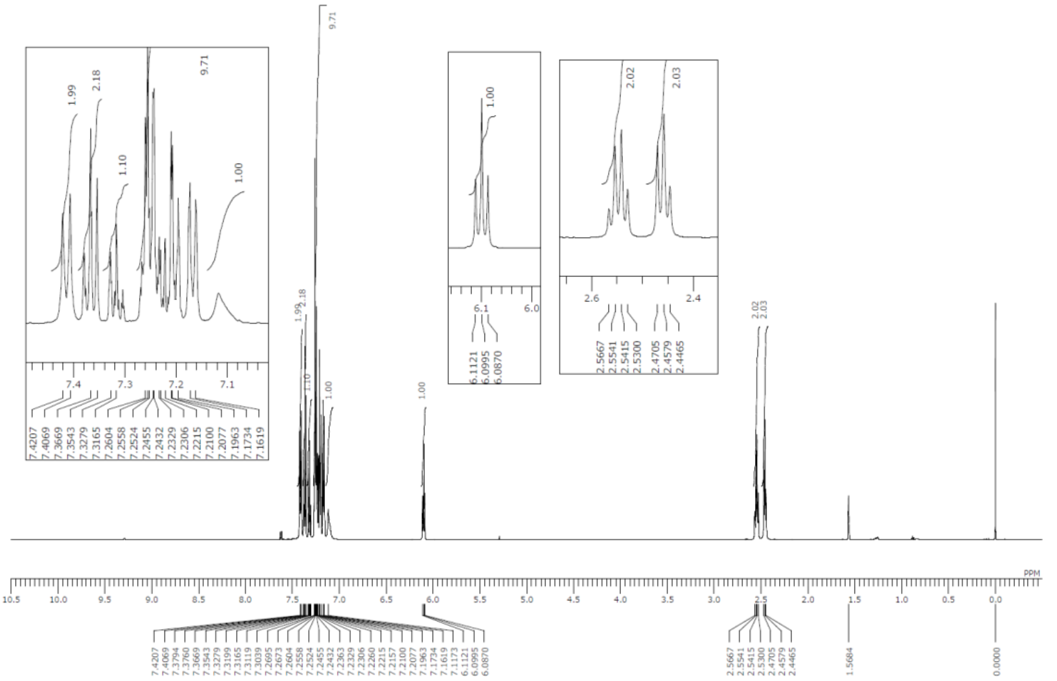

^{13}C-NMR spectrum of **1m** ($CDCl_3$, 150 MHz)

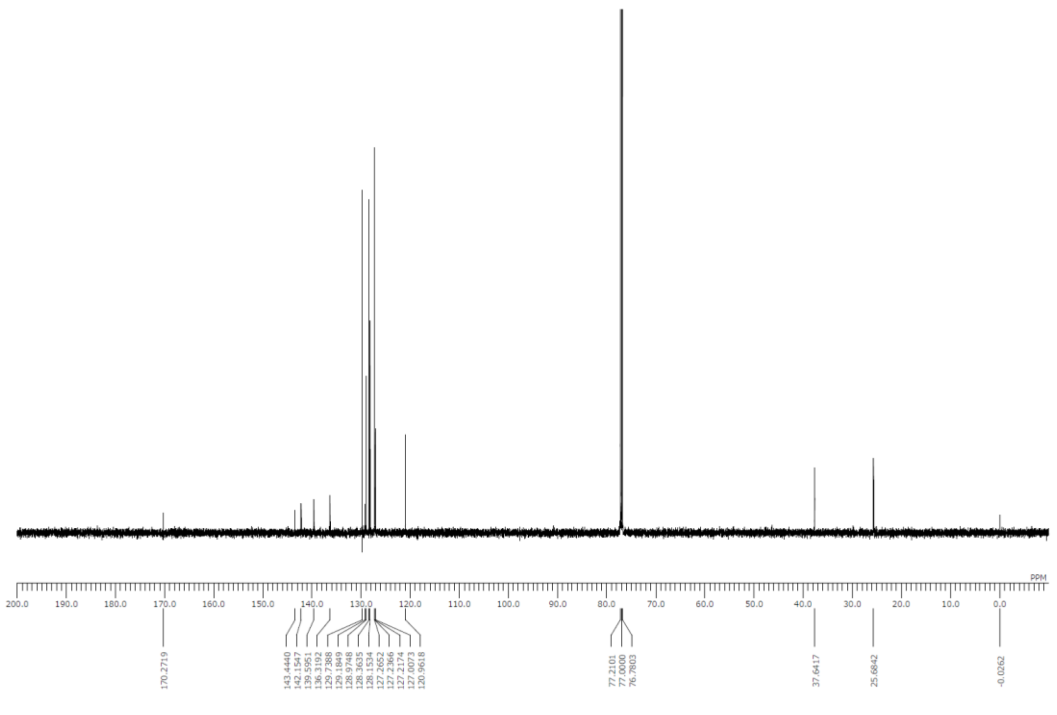

IR spectrum of **1m**

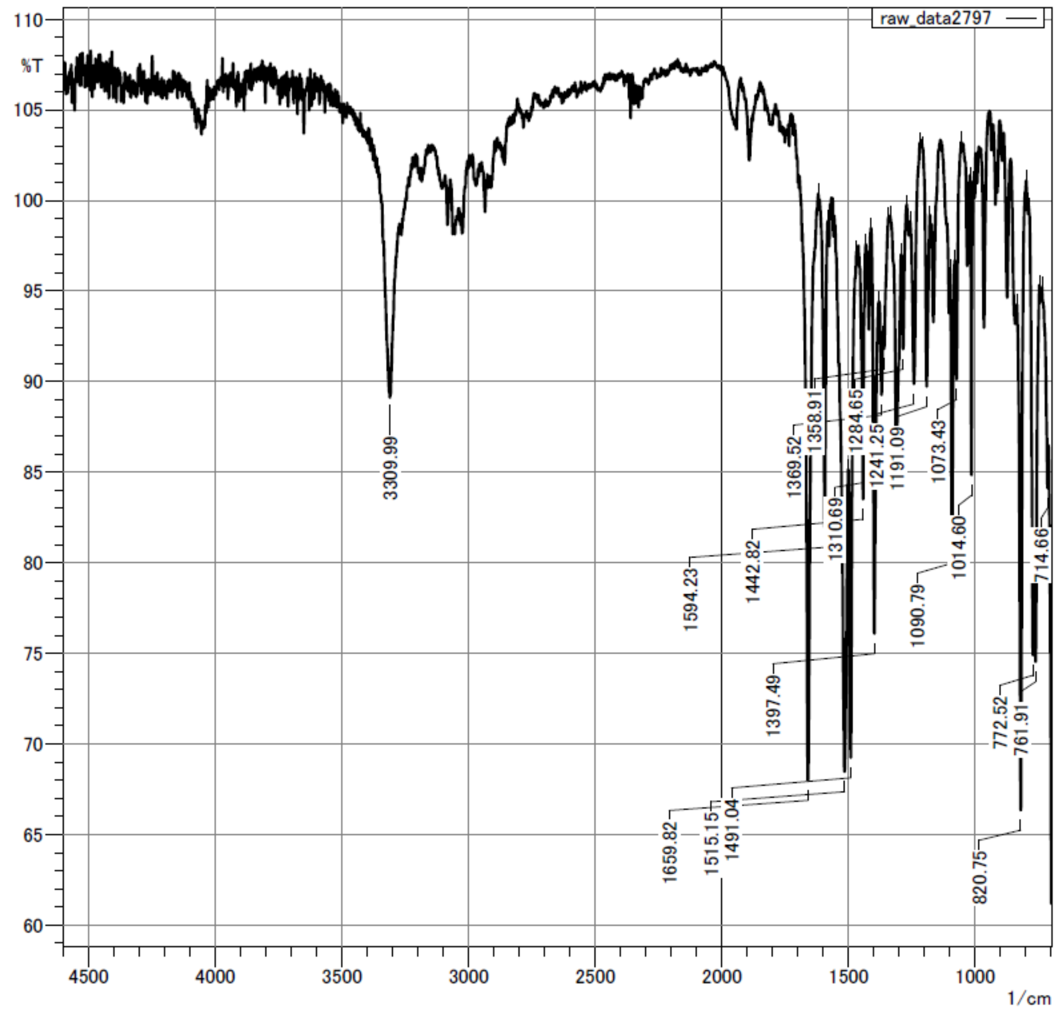

HRMS spectrum of **1m**

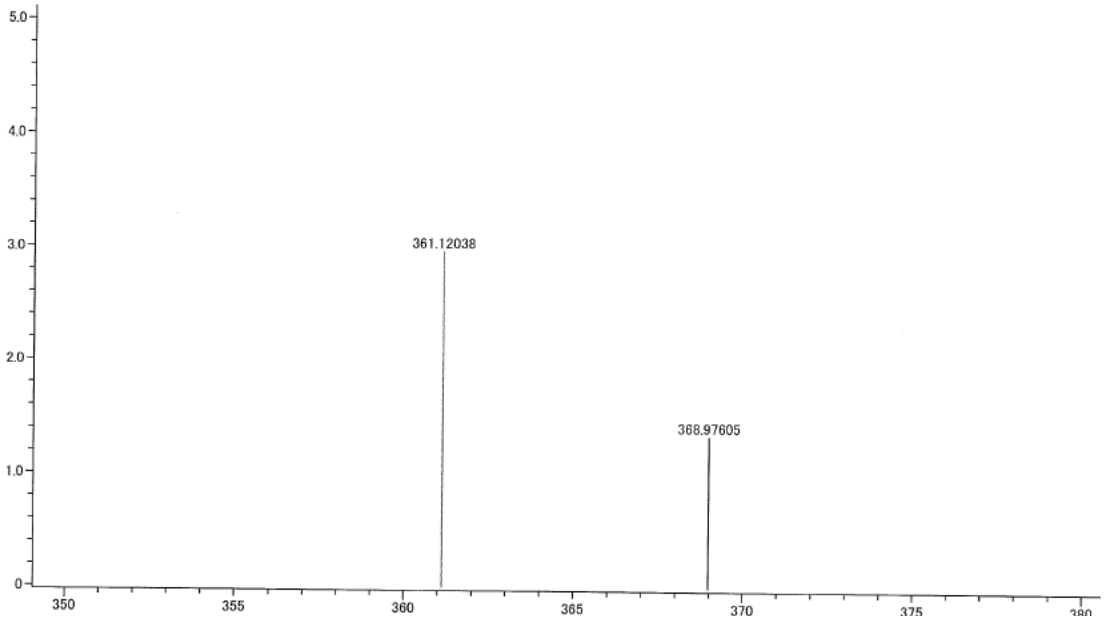

^{1}H-NMR spectrum of **1n** ($CDCl_3$, 600 MHz)

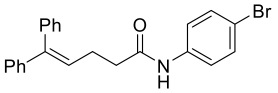

1n

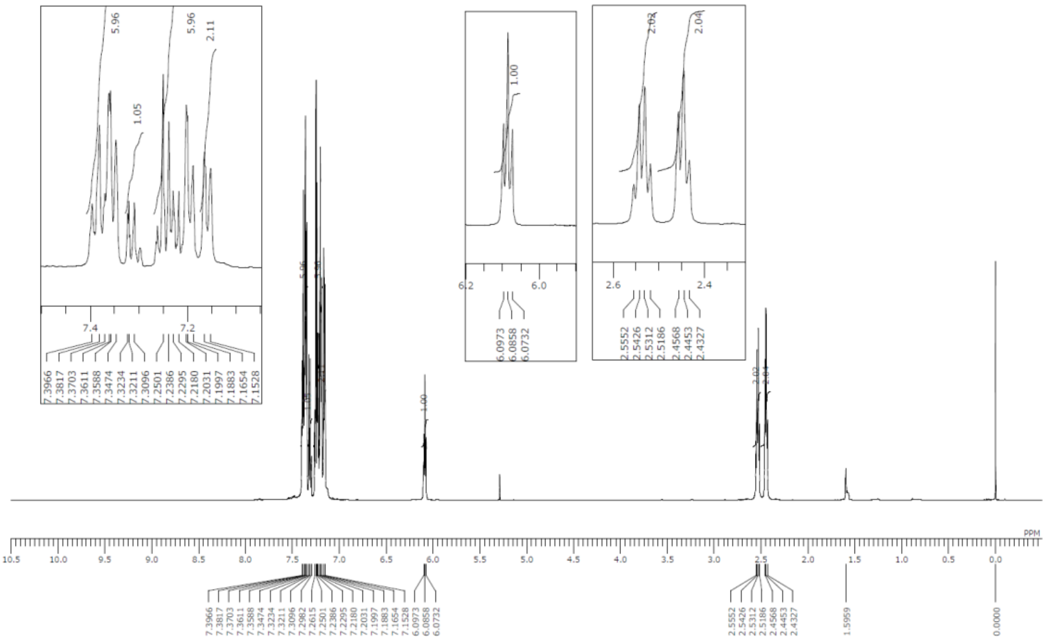

^{13}C-NMR spectrum of **1n** ($CDCl_3$, 150 MHz)

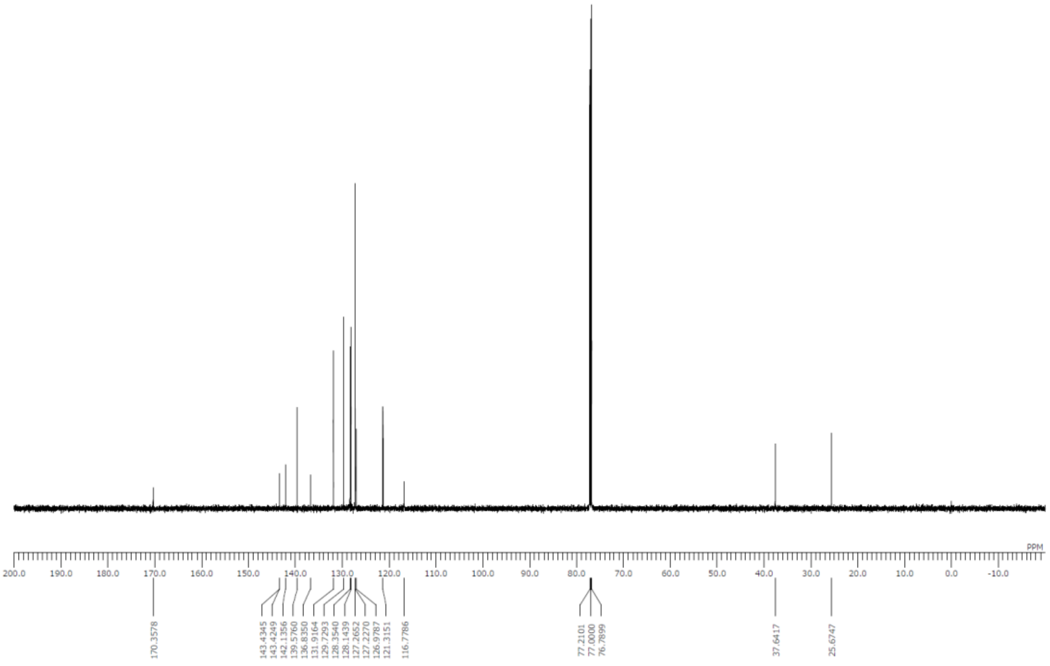

IR spectrum of **1n**

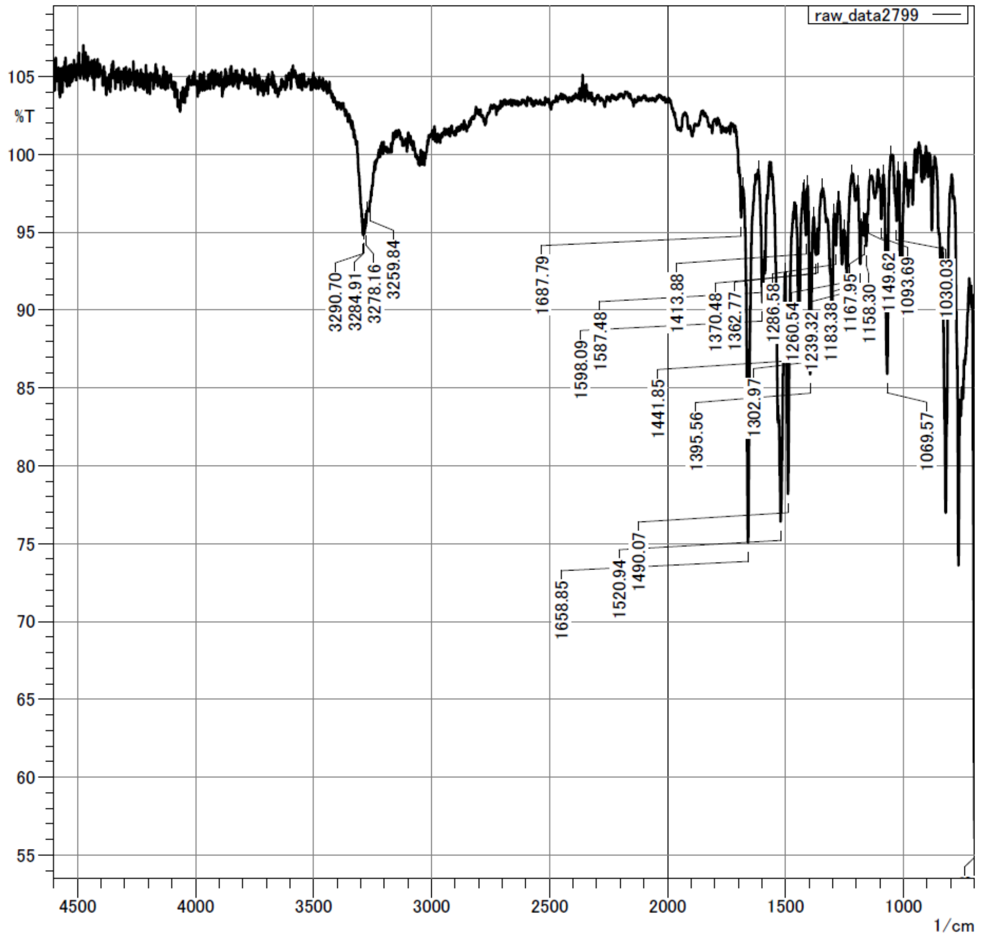

HRMS spectrum of **1n**

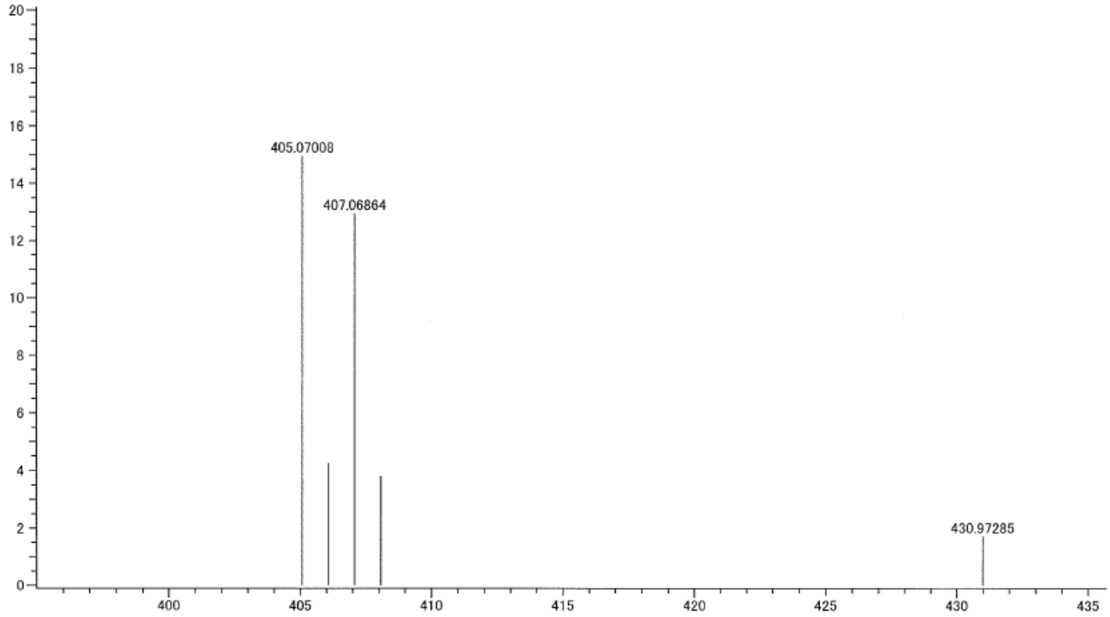

^{1}H-NMR spectrum of **1o** ($CDCl_3$, 600 MHz)

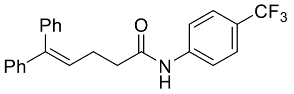

1o

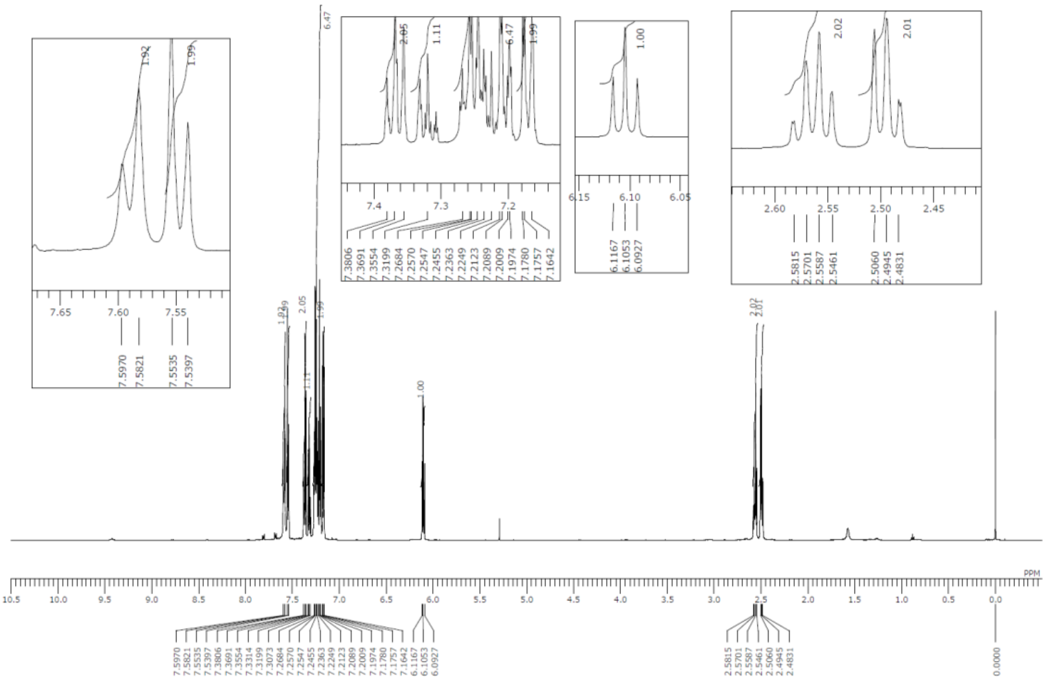

^{13}C-NMR spectrum of **1o** ($CDCl_3$, 150 MHz)

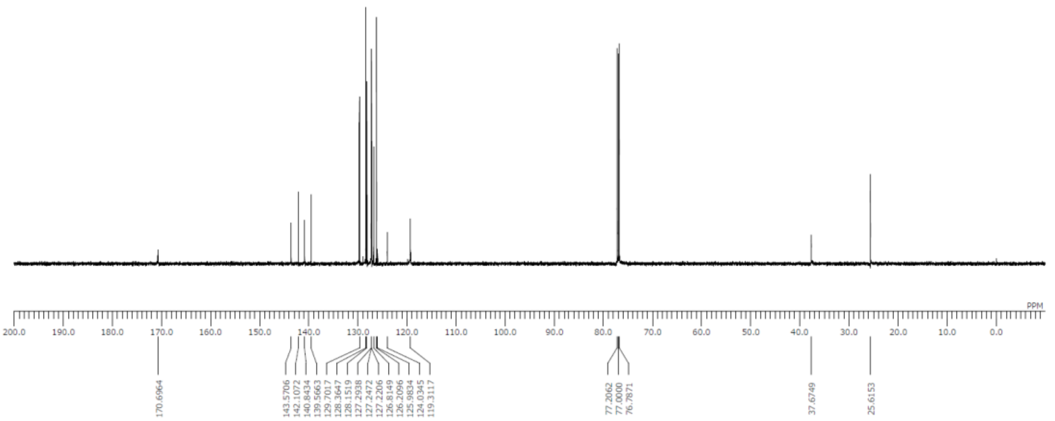

^{19}F-NMR spectrum of **1o** ($CDCl_3$, 565 MHz)

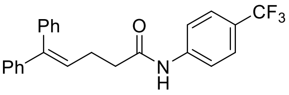

1o

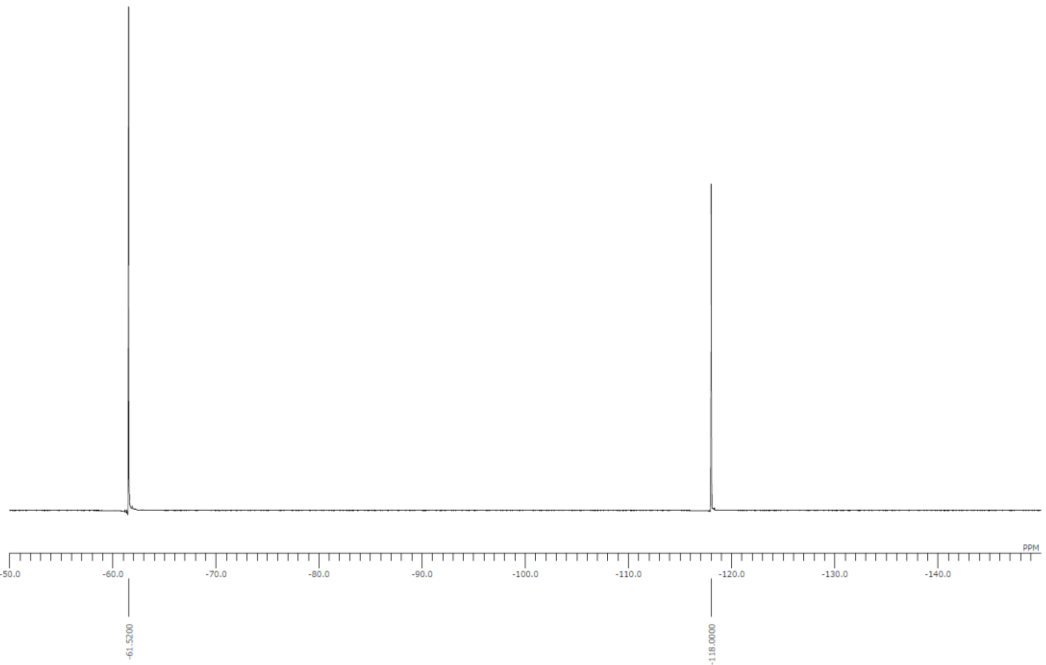

IR spectrum of **1o**

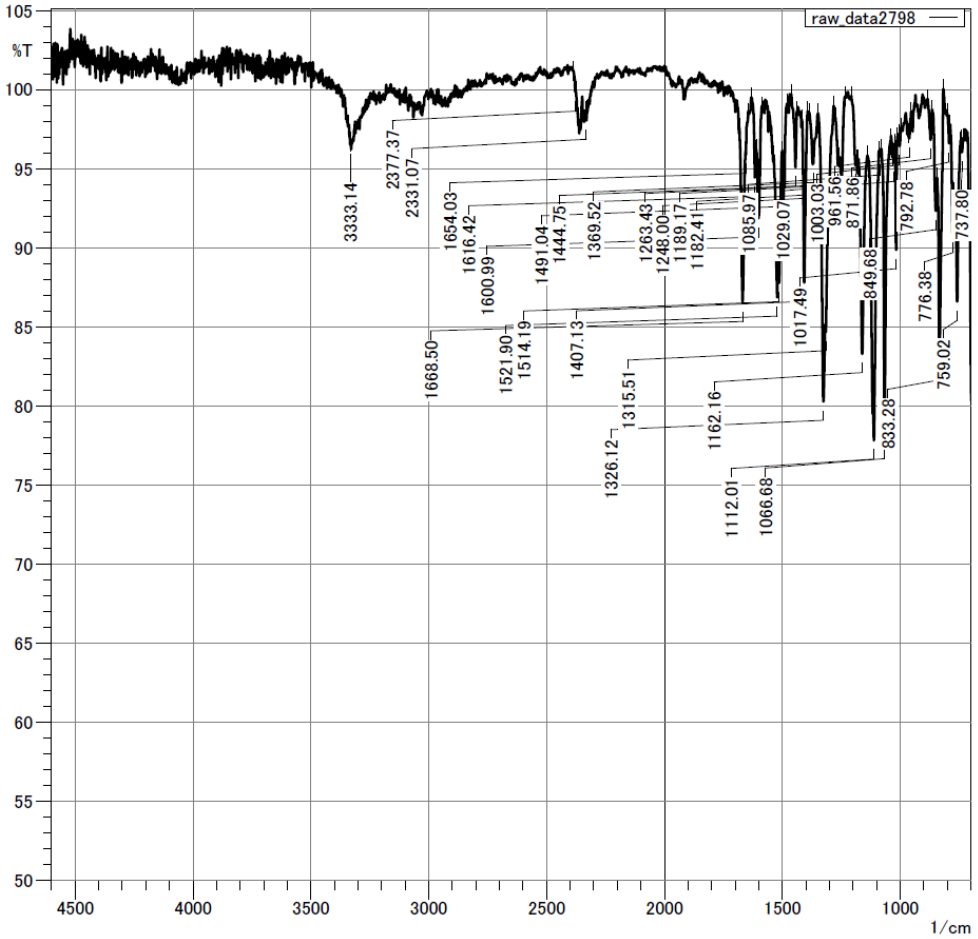

HRMS spectrum of **1o**

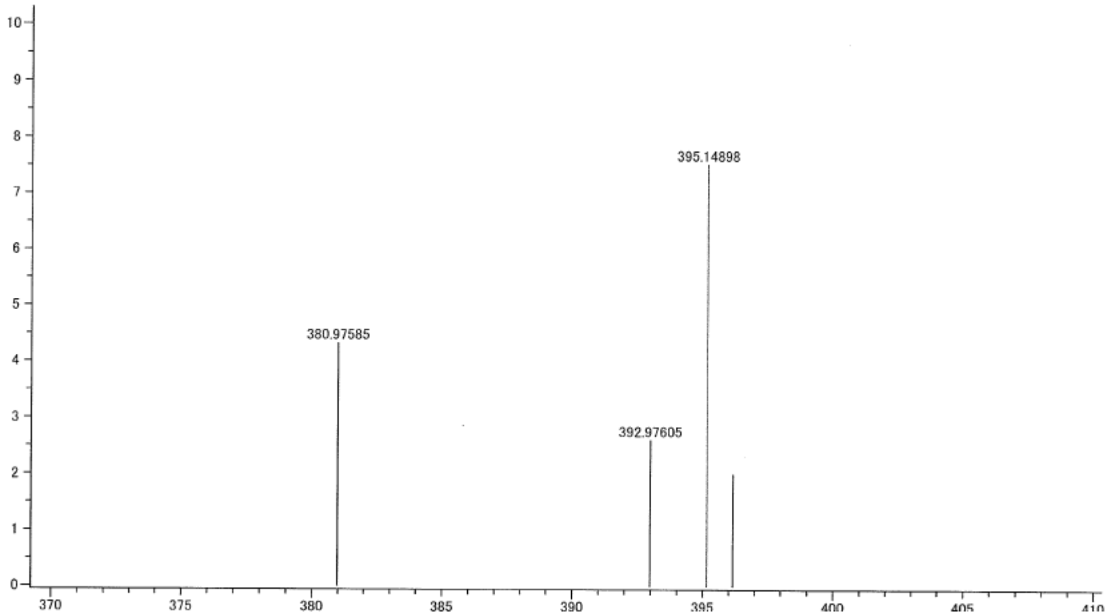

^{1}H-NMR spectrum of **1p** ($CDCl_3$, 600 MHz)

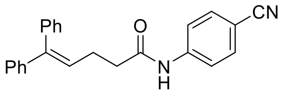

1p

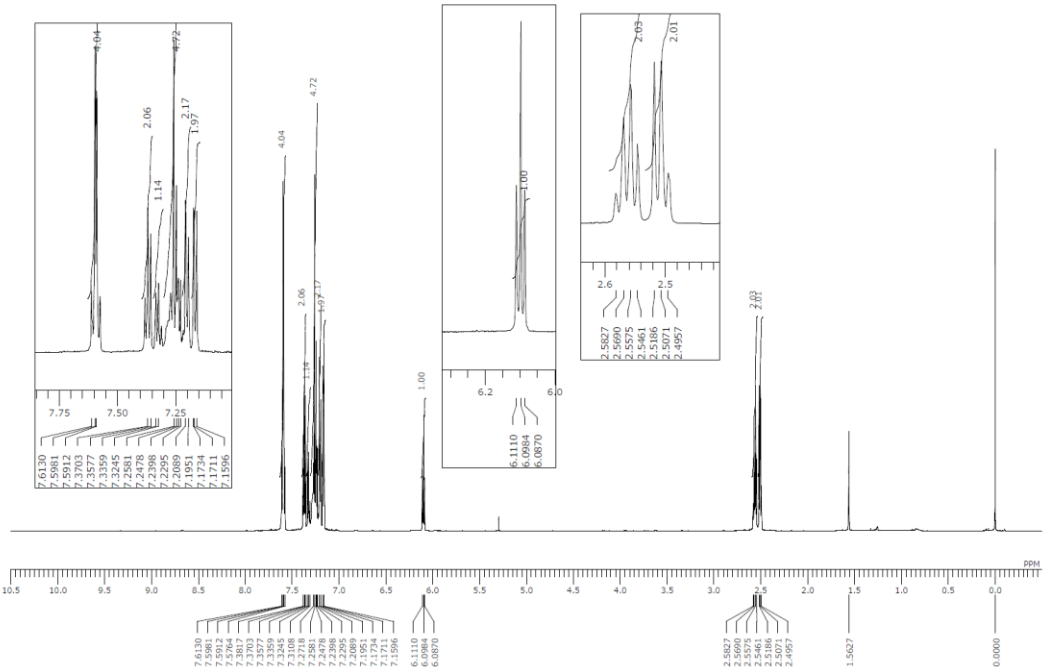

^{13}C-NMR spectrum of **1p** ($CDCl_3$, 150 MHz)

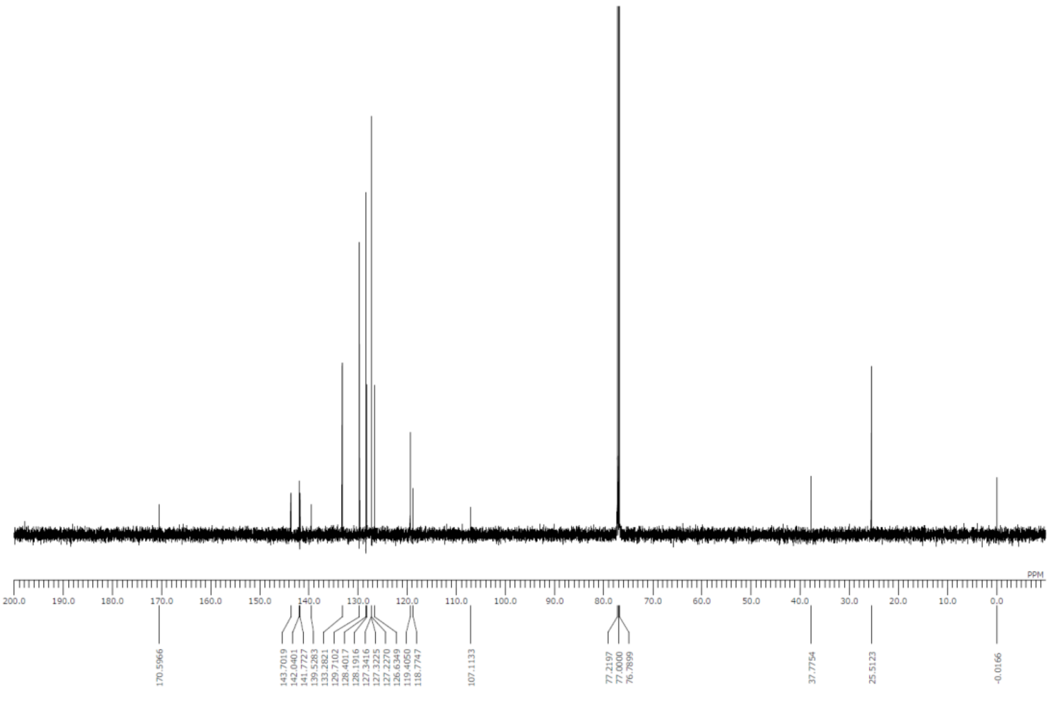

IR spectrum of **1p**

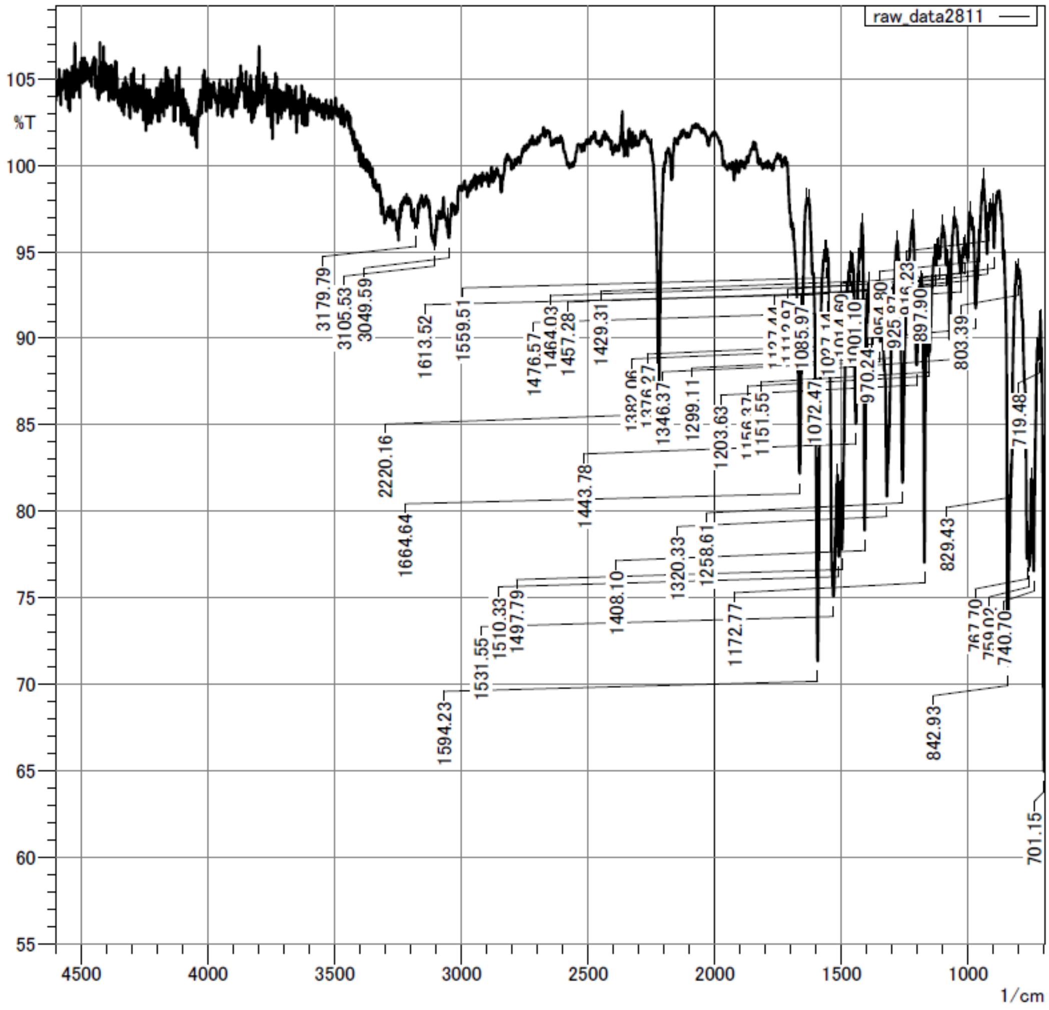

HRMS spectrum of **1p**

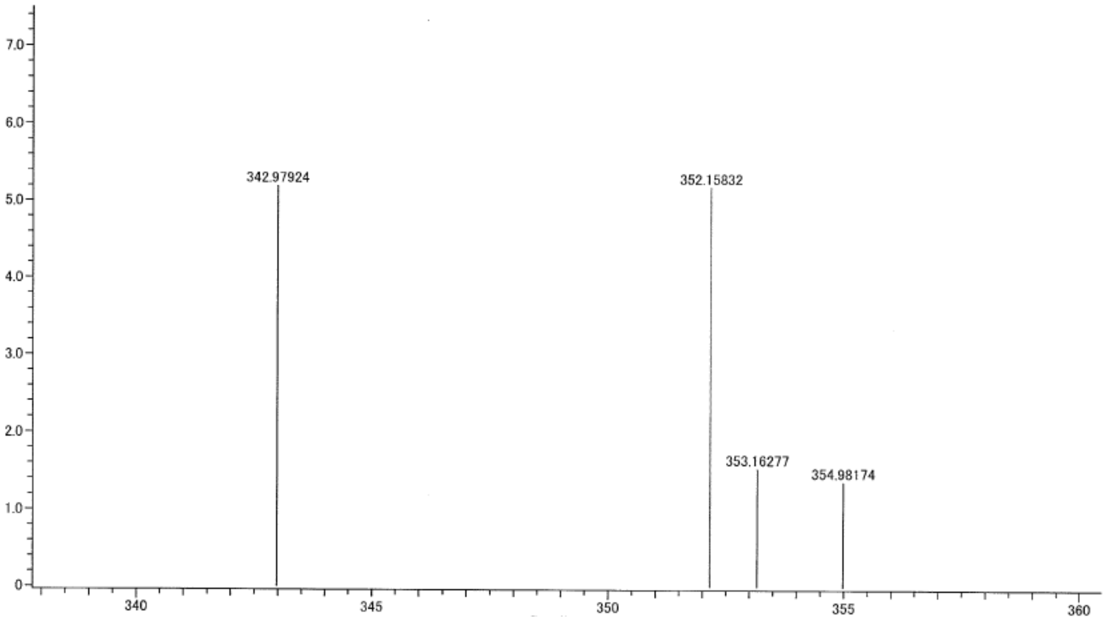

^{1}H-NMR spectrum of **1q** (DMSO-d_6, 600 MHz)

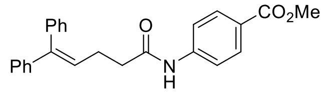

1q

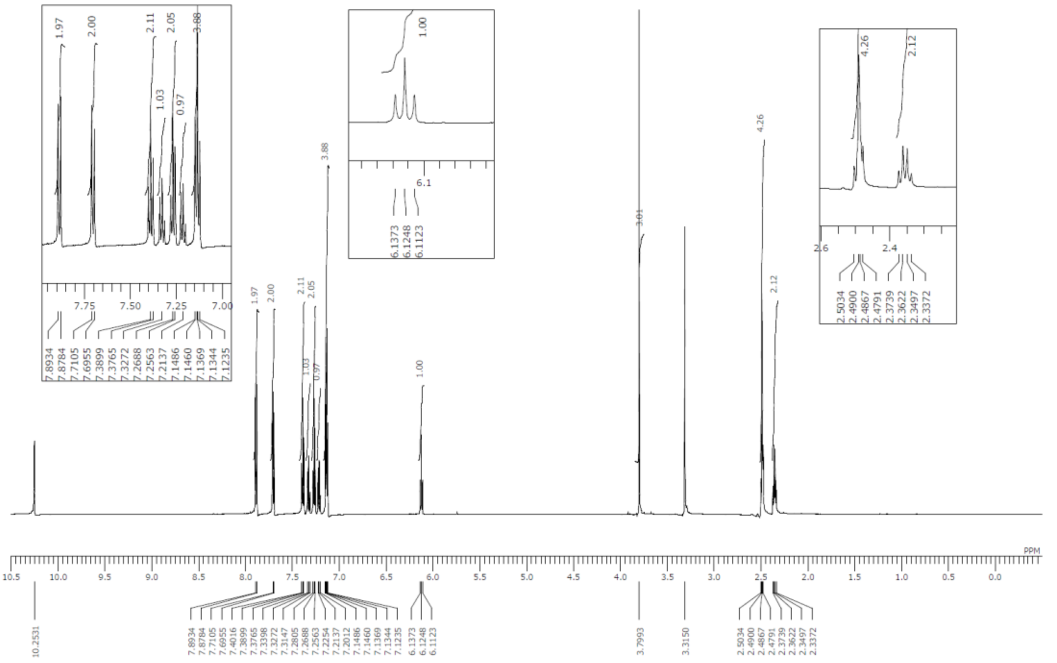

^{13}C-NMR spectrum of **1q** (DMSO-d_6, 150 MHz)

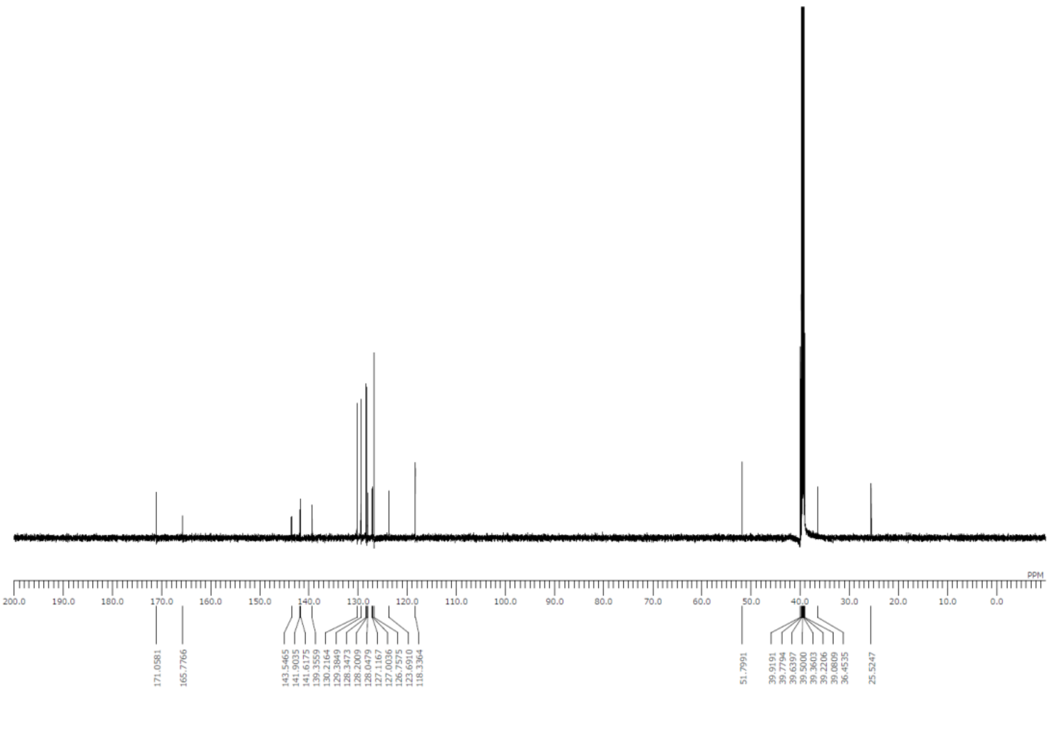

IR spectrum of **1q**

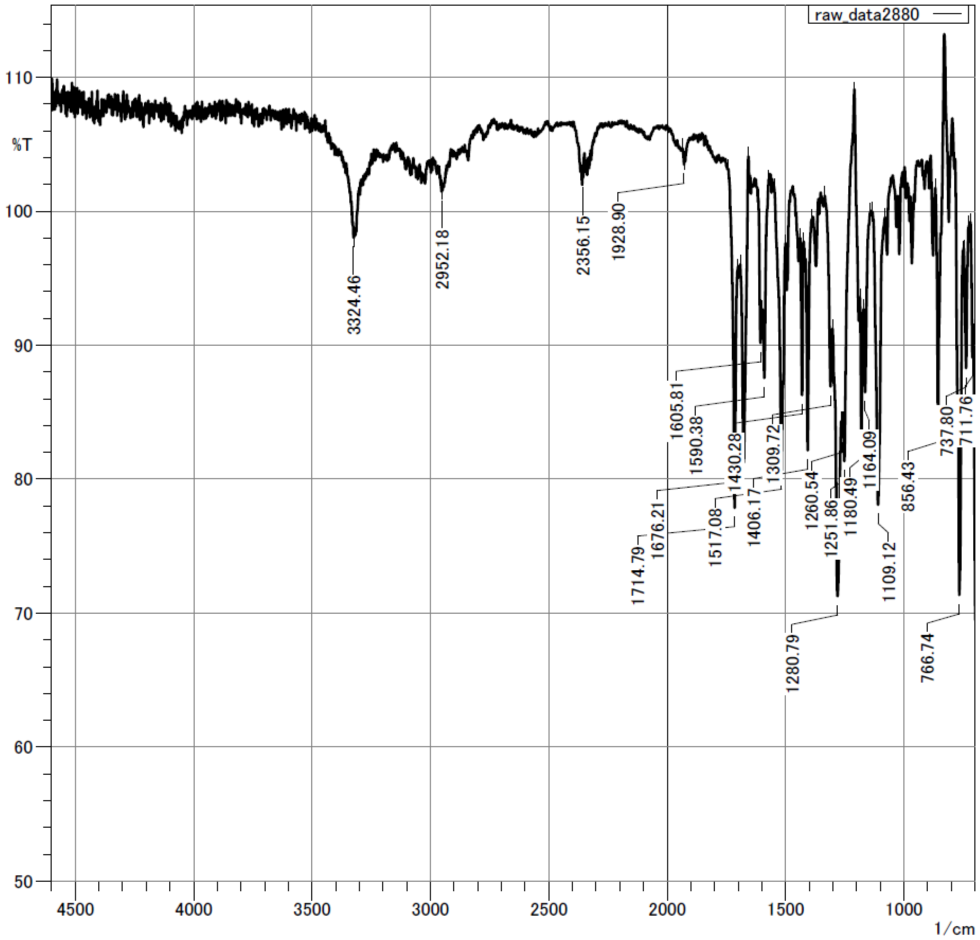

HRMS spectrum of **1q**

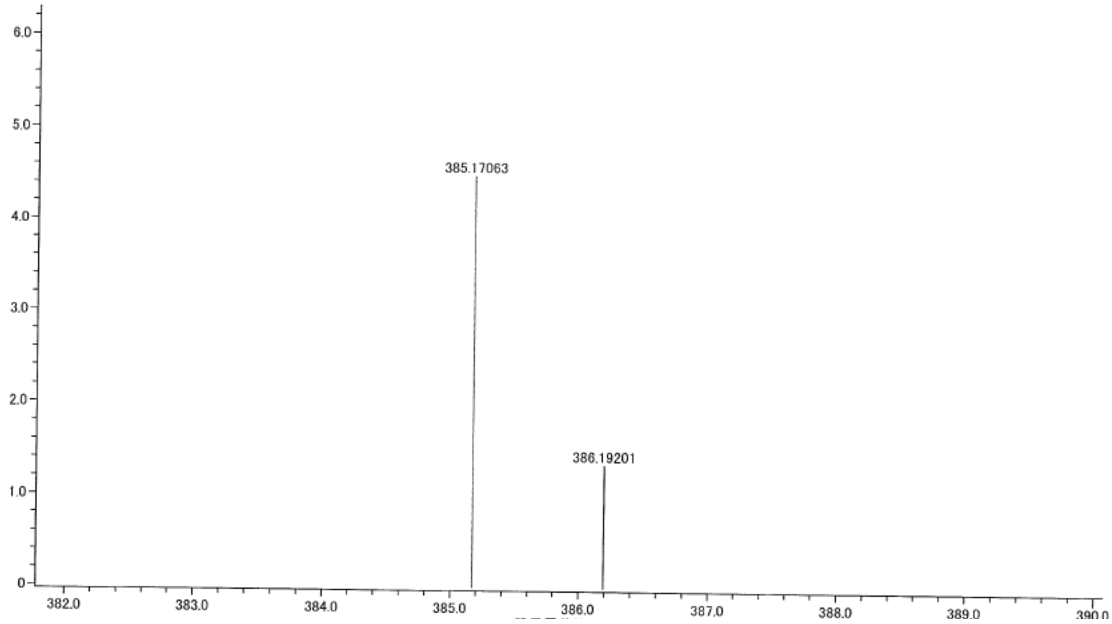

^{1}H-NMR spectrum of **1r** ($CDCl_3$, 600 MHz)

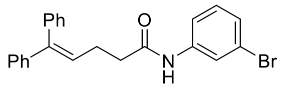

1r

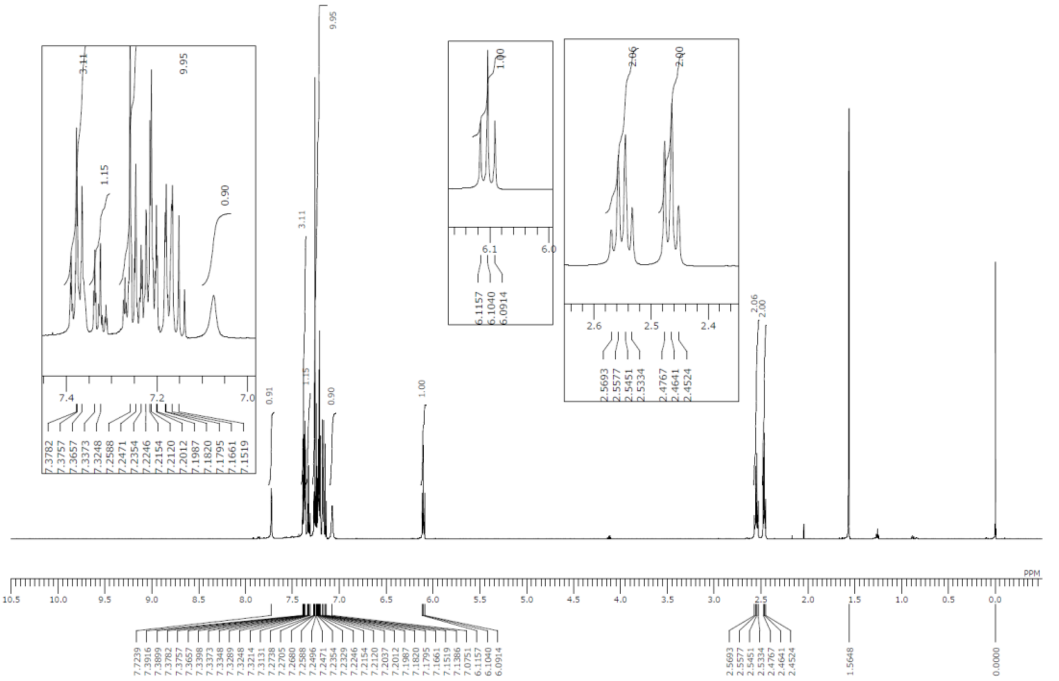

^{13}C-NMR spectrum of **1r** ($CDCl_3$, 150 MHz)

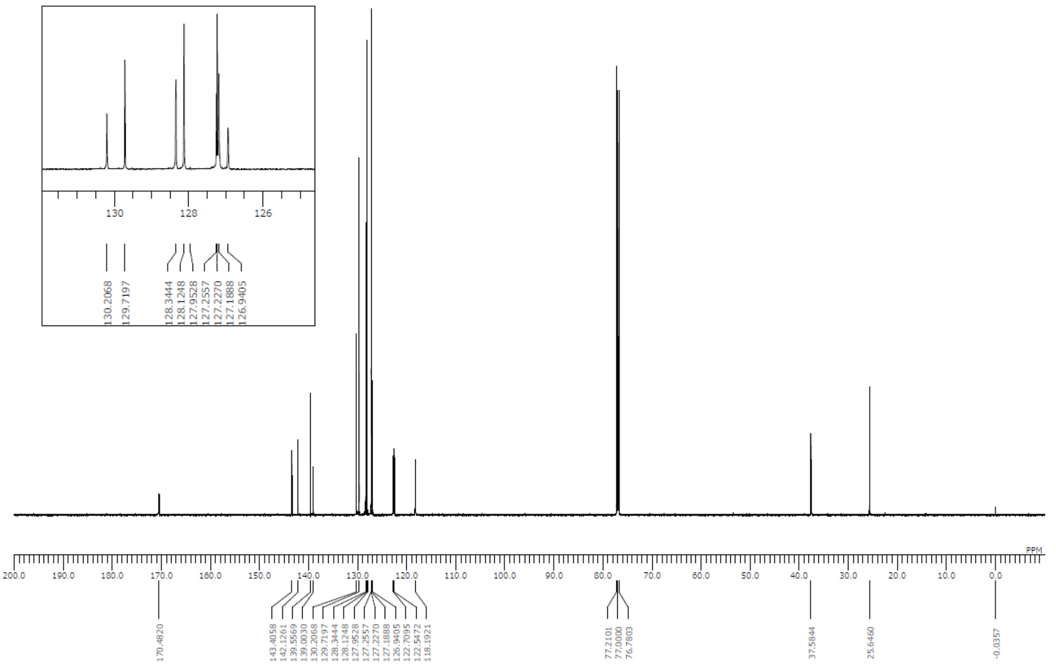

IR spectrum of **1r**

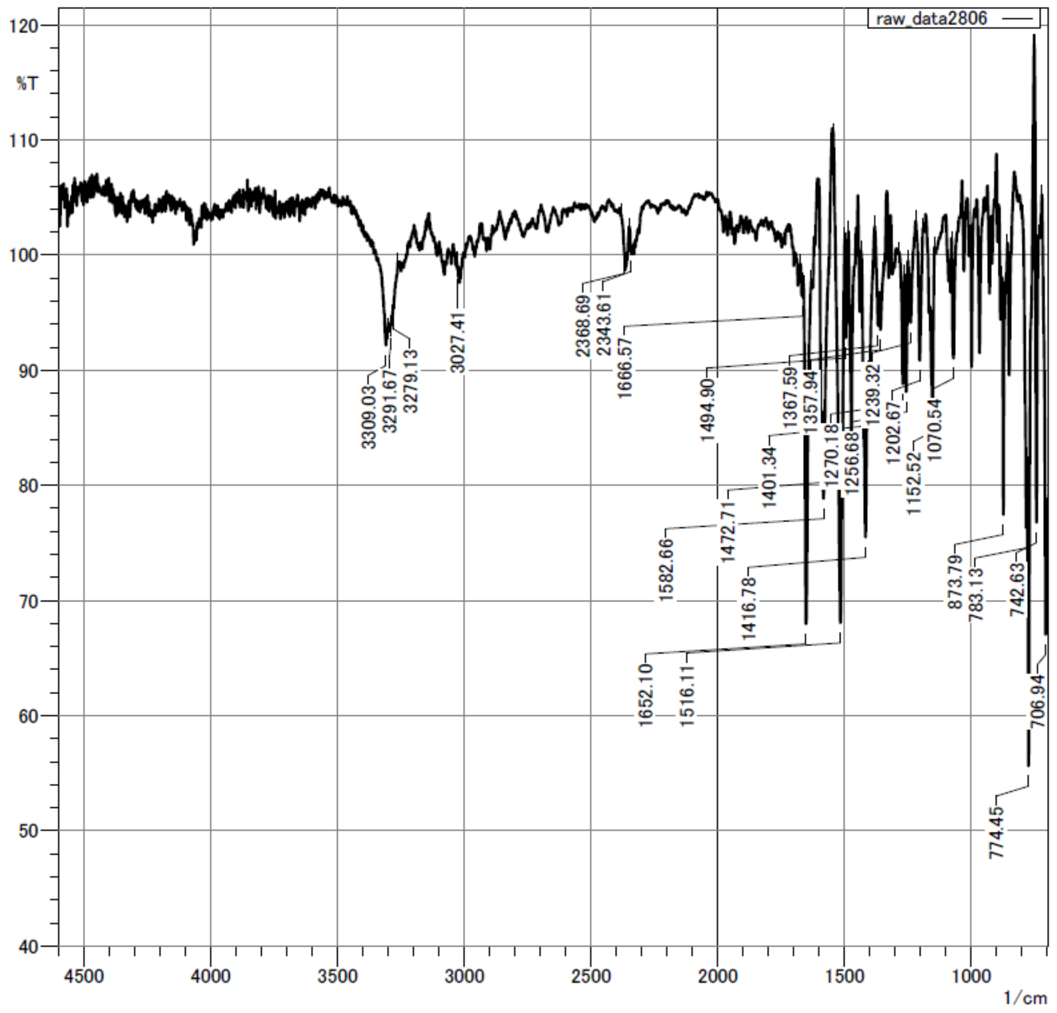

HRMS spectrum of **1r**

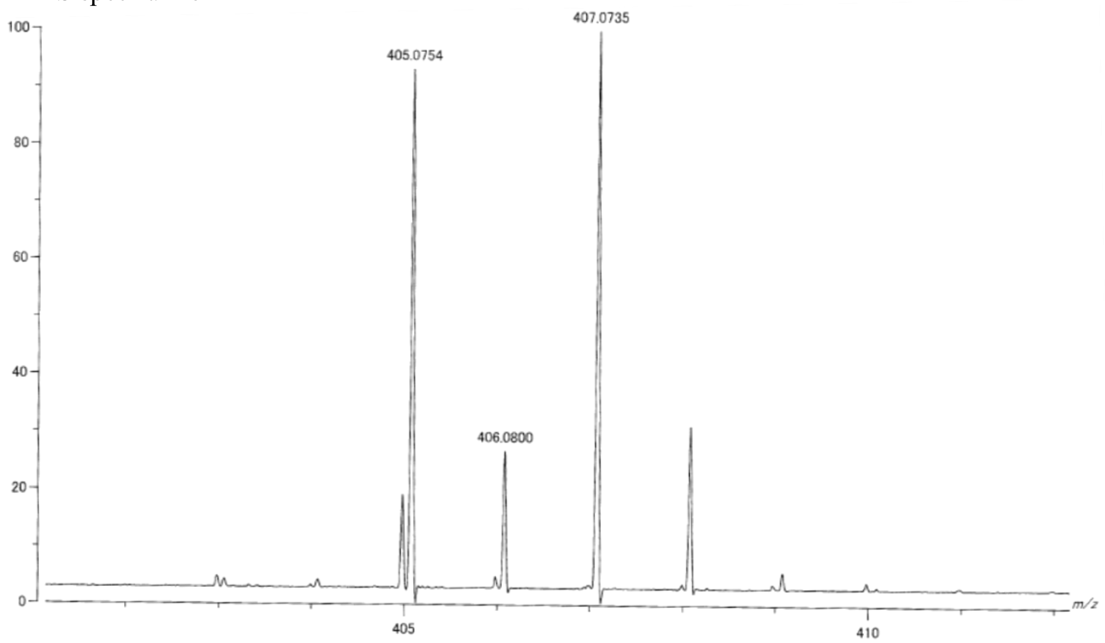

^{1}H-NMR spectrum of **1s** ($CDCl_3$, 600 MHz)

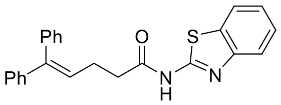

1s

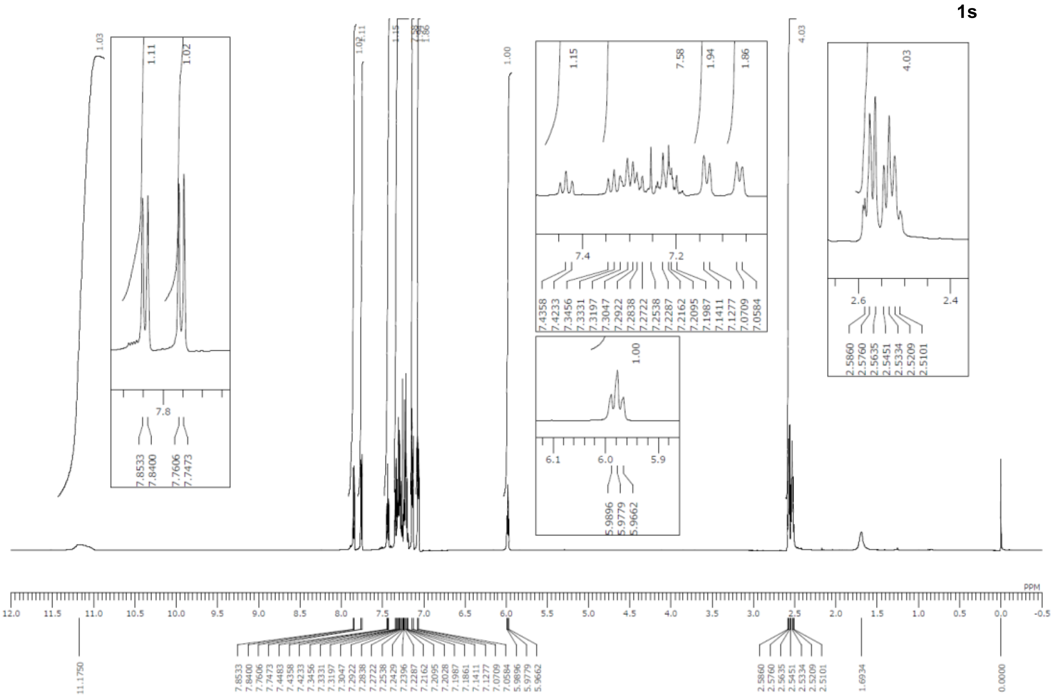

^{13}C-NMR spectrum of **1s** ($CDCl_3$, 150 MHz)

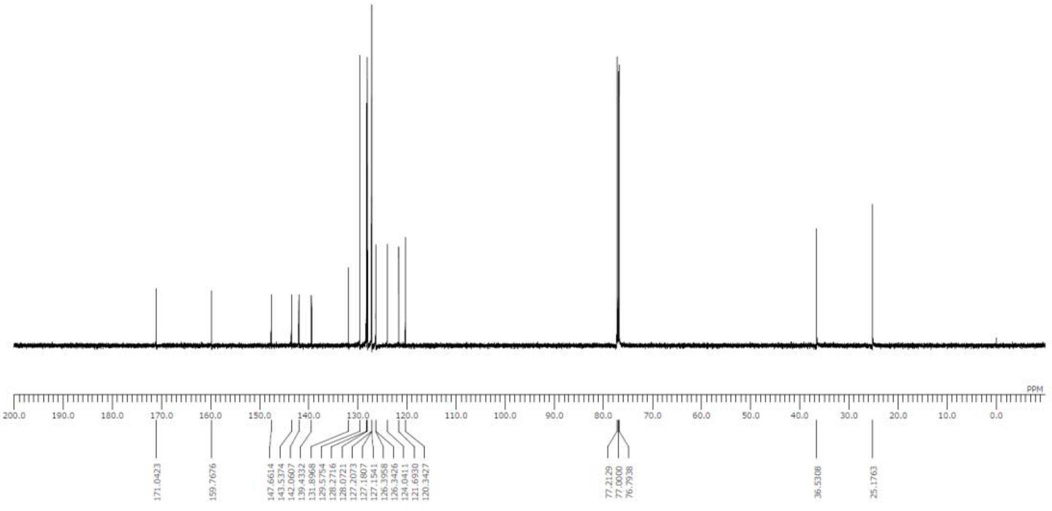

IR spectrum of **1s**

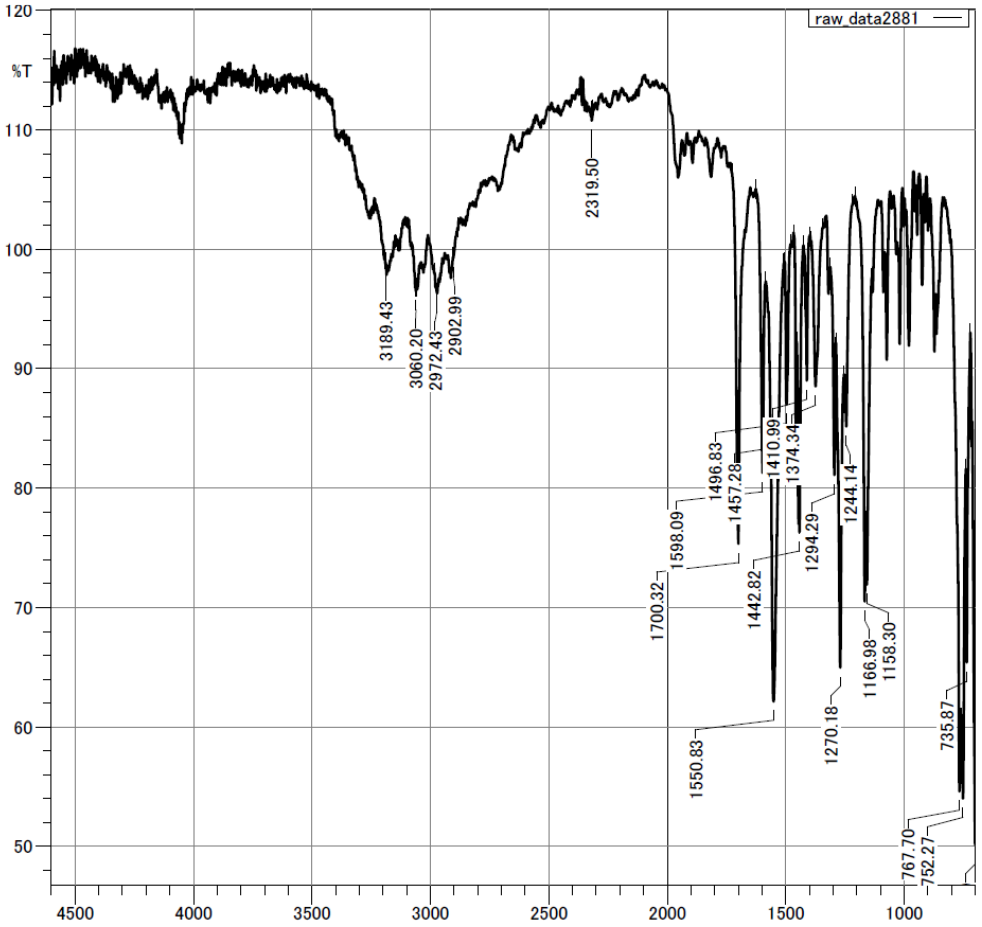

HRMS spectrum of **1s**

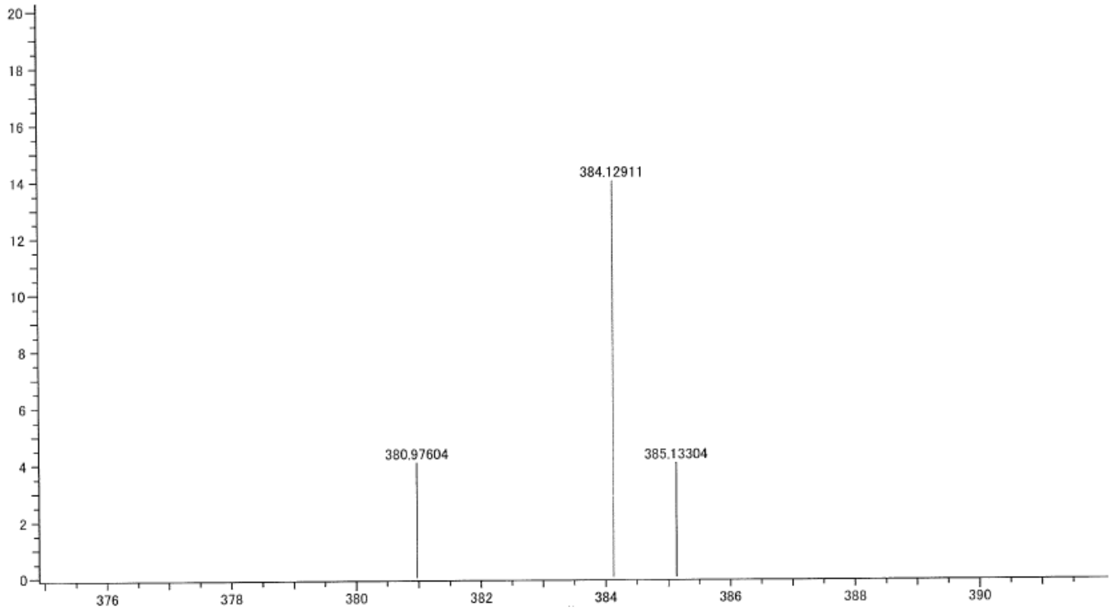

^{1}H-NMR spectrum of **2a** ($CDCl_3$, 400 MHz)

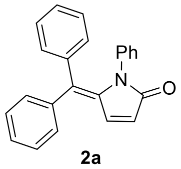

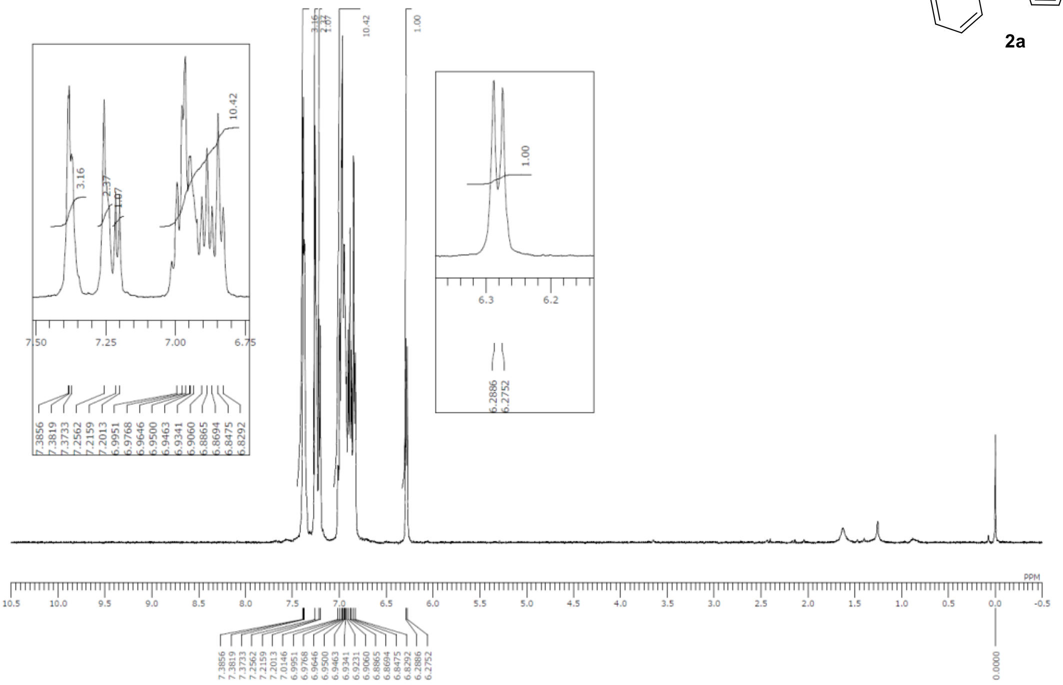

^{13}C-NMR spectrum of **2a** ($CDCl_3$, 150 MHz)

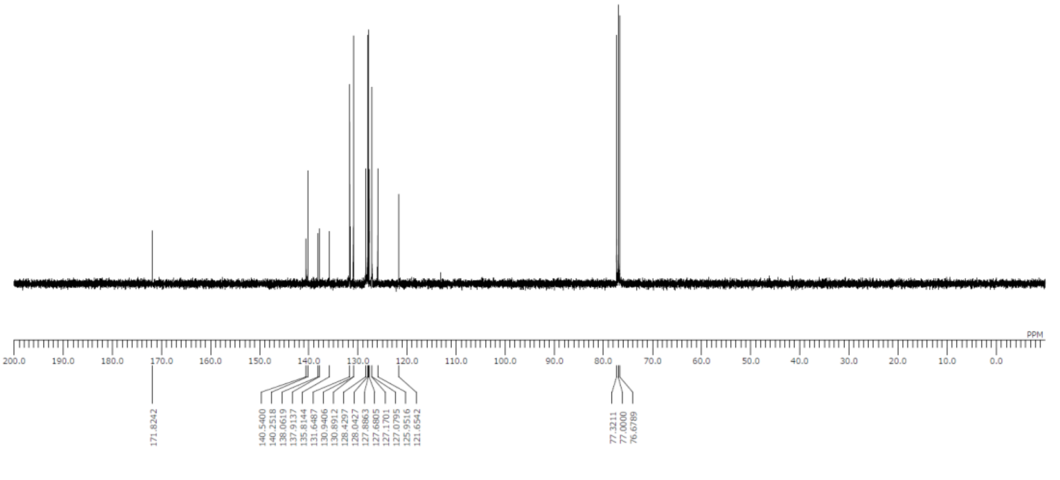

IR spectrum of **2a**

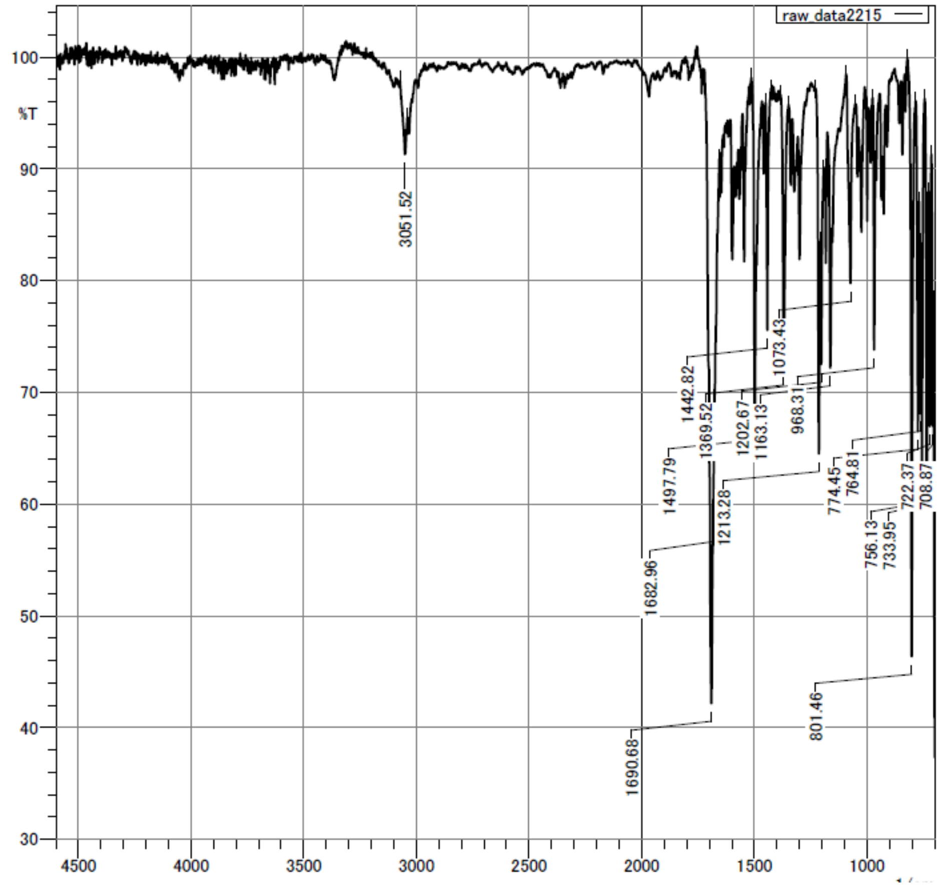

HRMS spectrum of **2a**

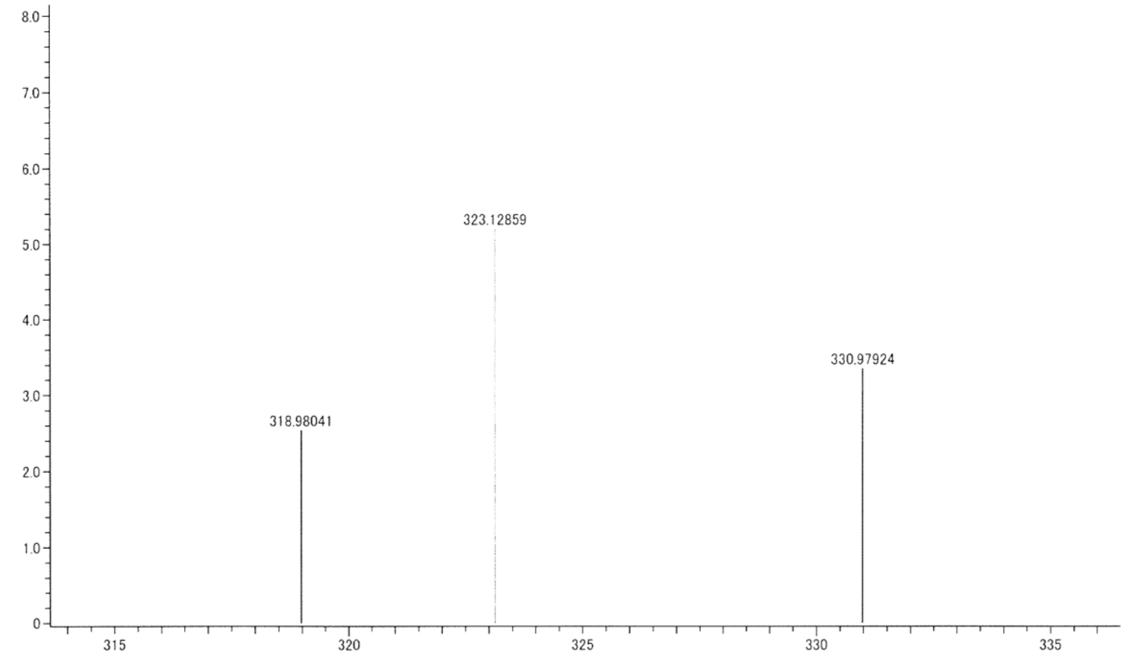

^{1}H-NMR spectrum of **2b** ($CDCl_3$, 600 MHz)

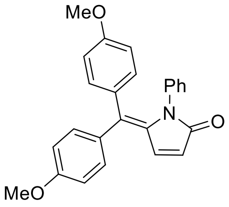

2b

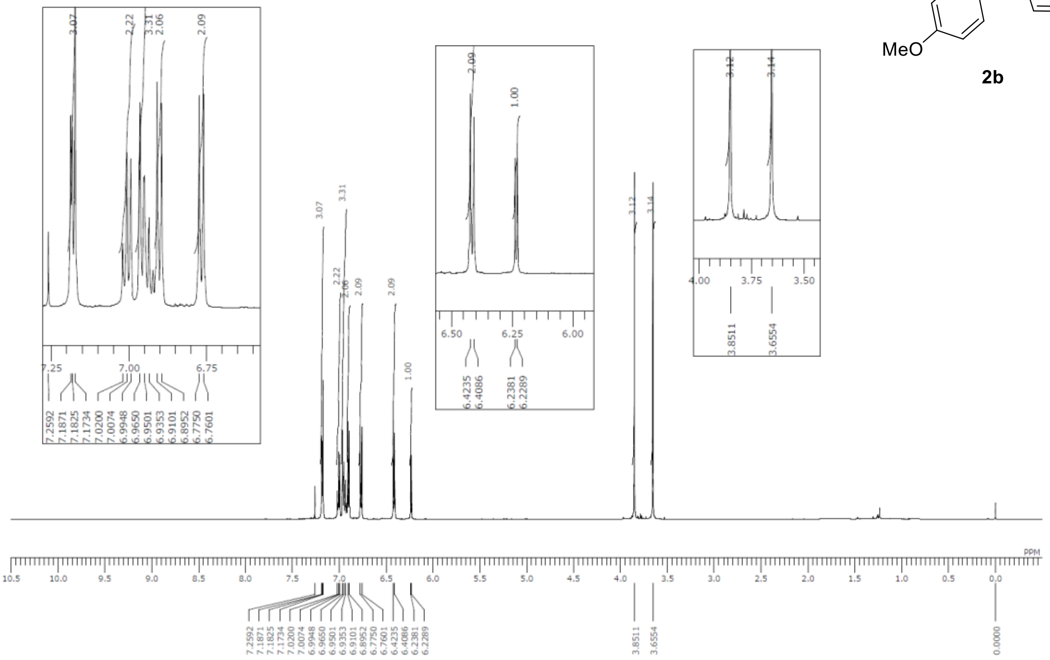

^{13}C-NMR spectrum of **2b** ($CDCl_3$, 150 MHz)

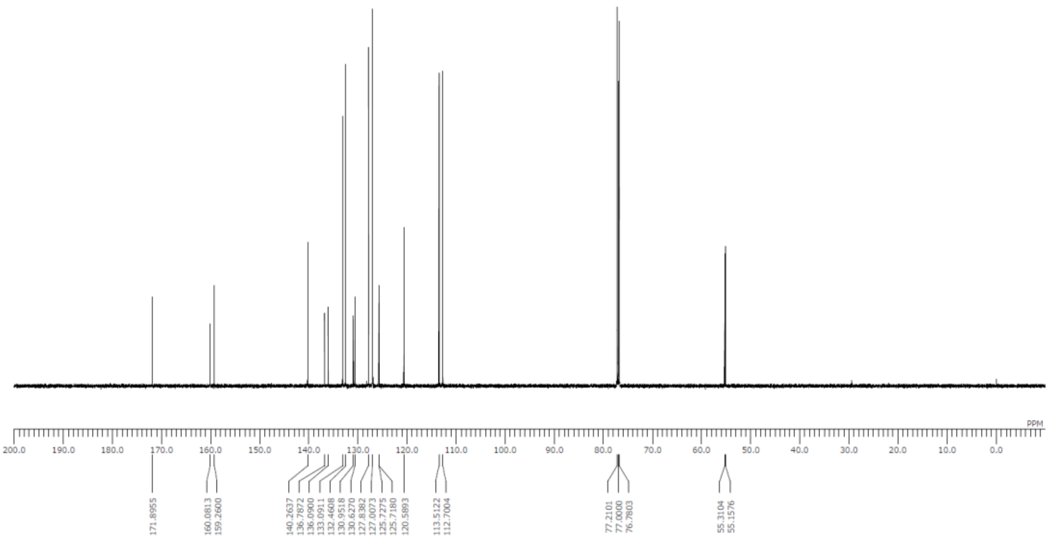

IR spectrum of **2b**

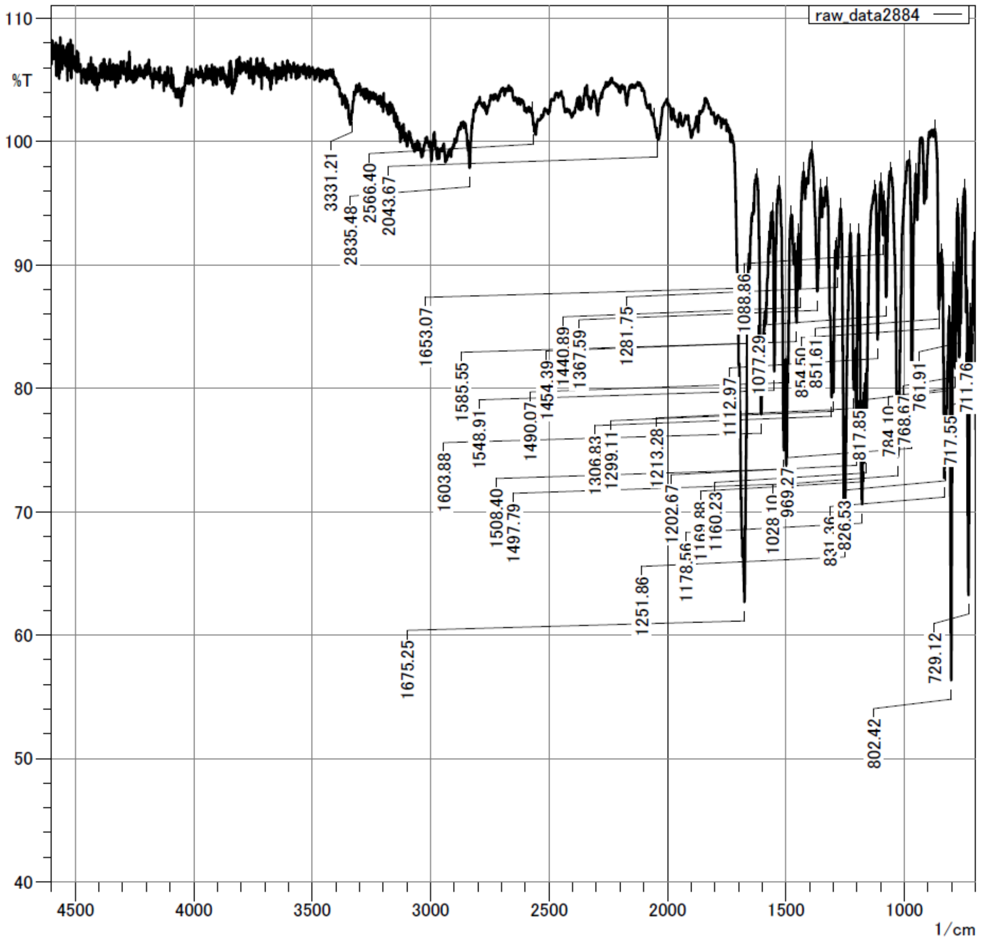

HRMS spectrum of **2b**

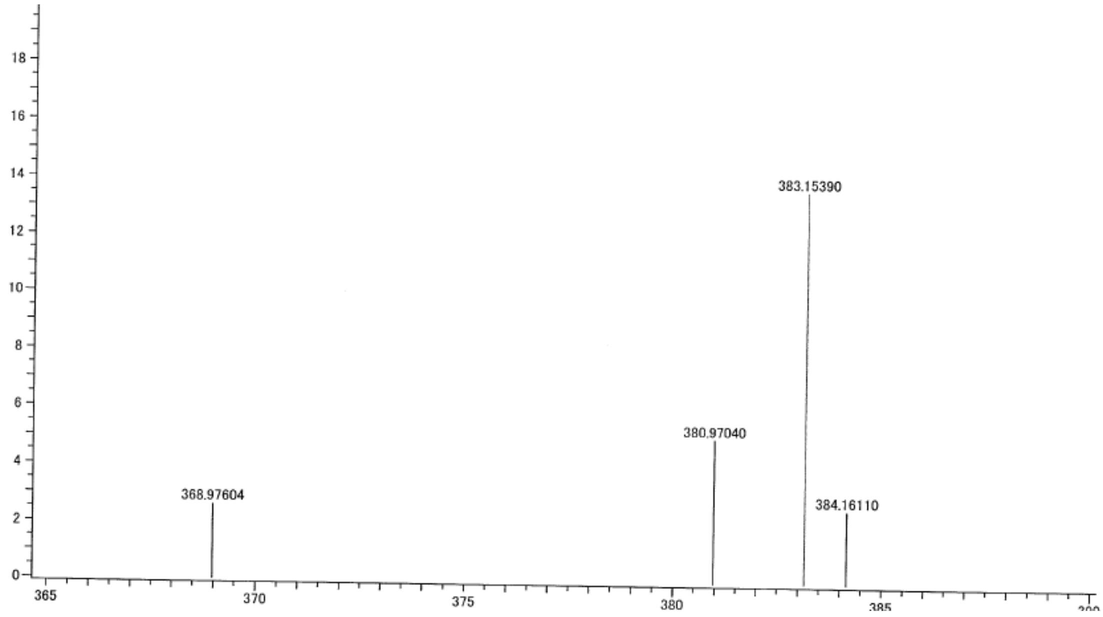

^{1}H-NMR spectrum of **2c** (DMSO-d_6, 600 MHz)

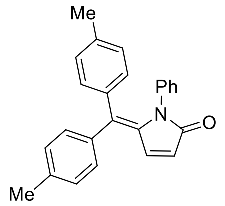

2c

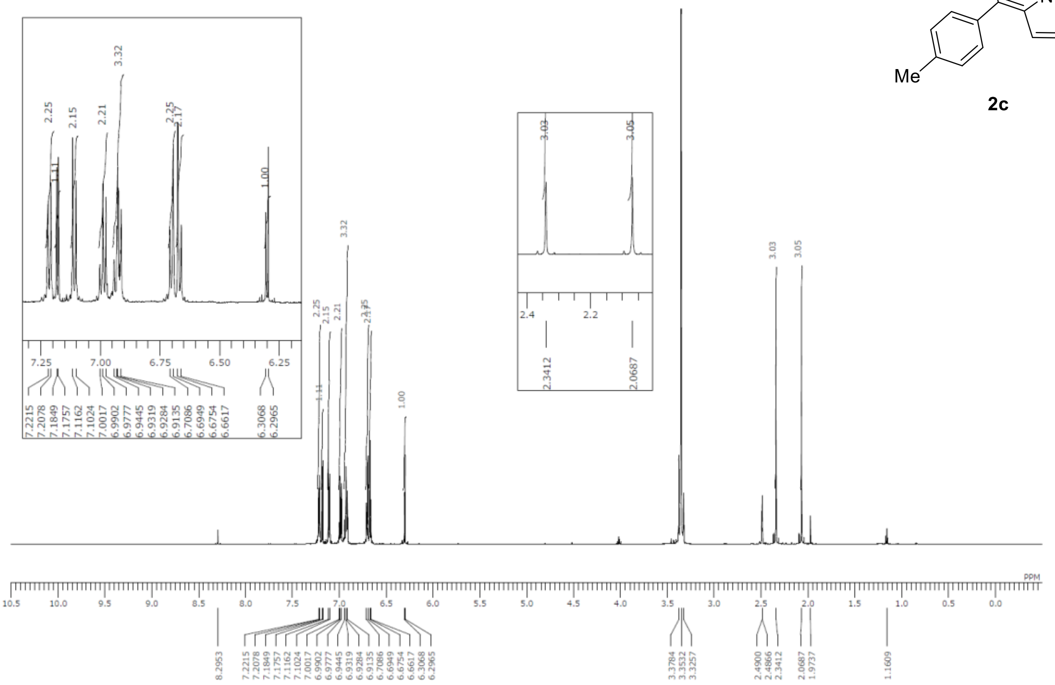

^{13}C-NMR spectrum of **2c** (DMSO-d_6, 150 MHz)

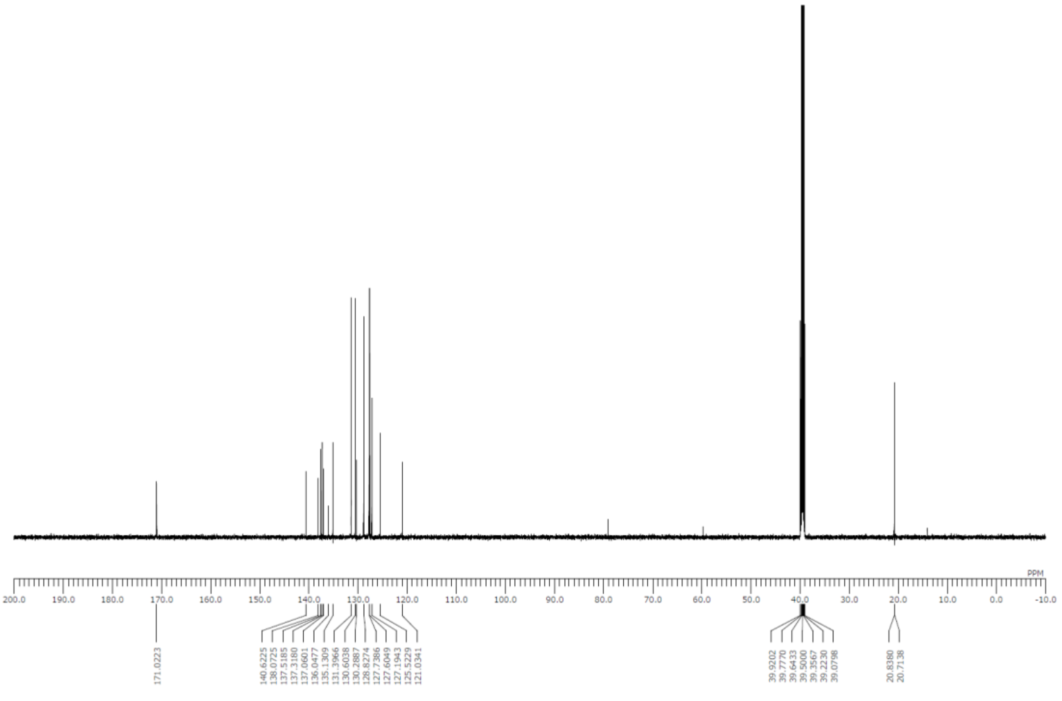

IR spectrum of **2c**

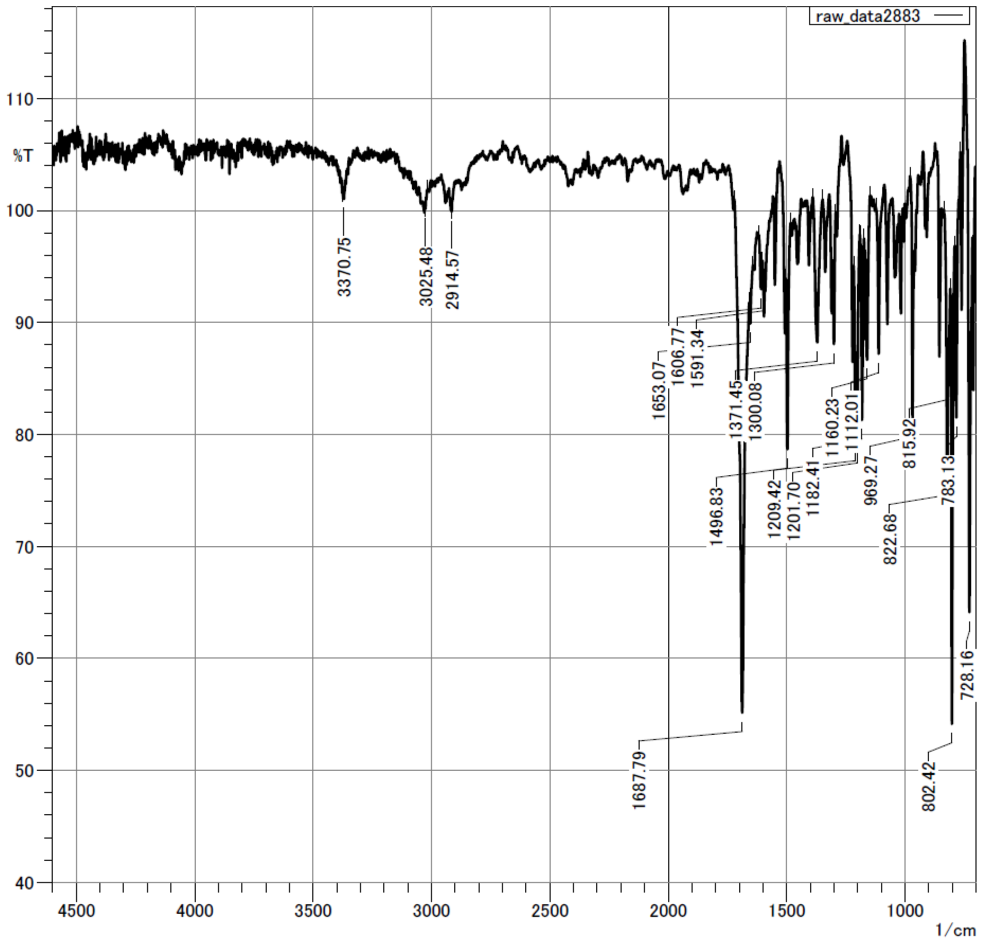

HRMS spectrum of **2c**

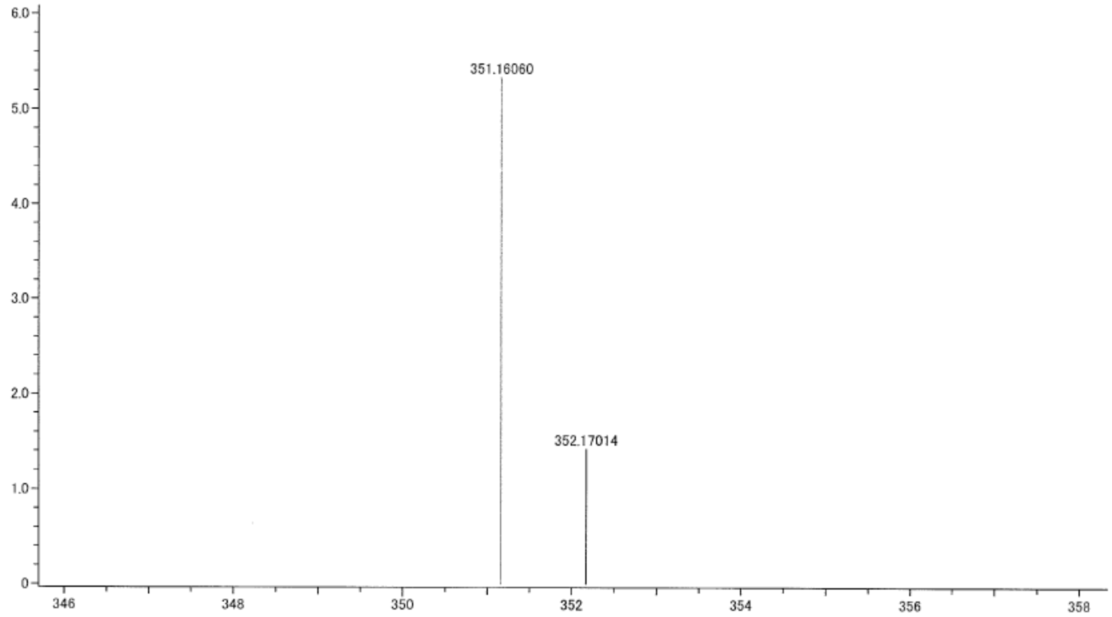

^{1}H-NMR spectrum of **2d** ($CDCl_3$, 600 MHz)

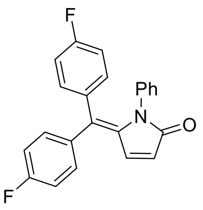

2d

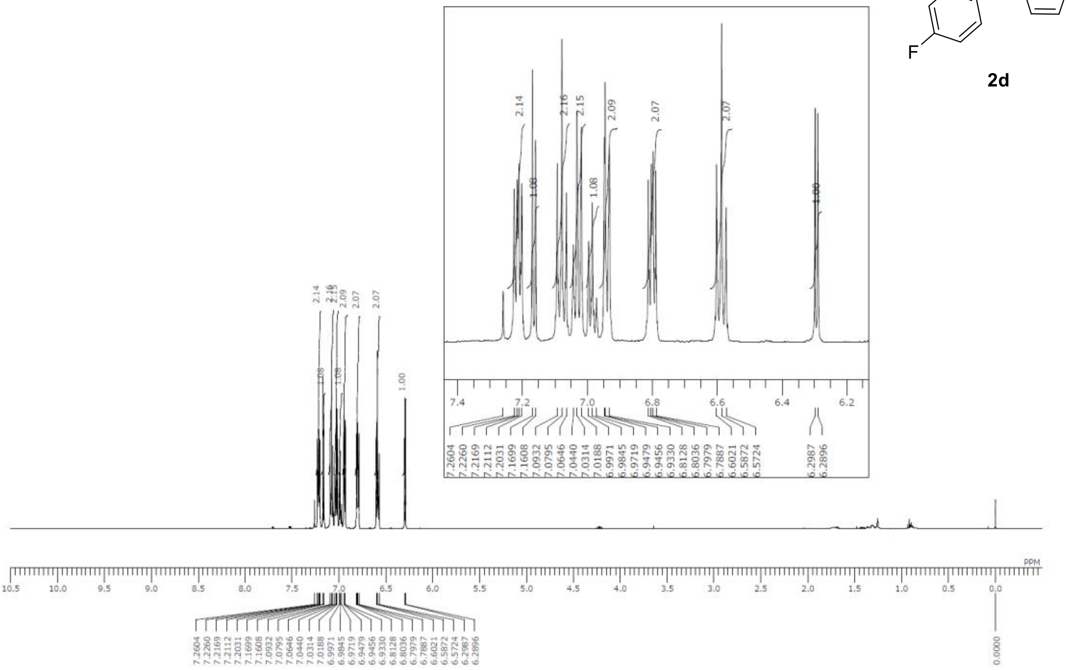

^{13}C-NMR spectrum of **2d** ($CDCl_3$, 150 MHz)

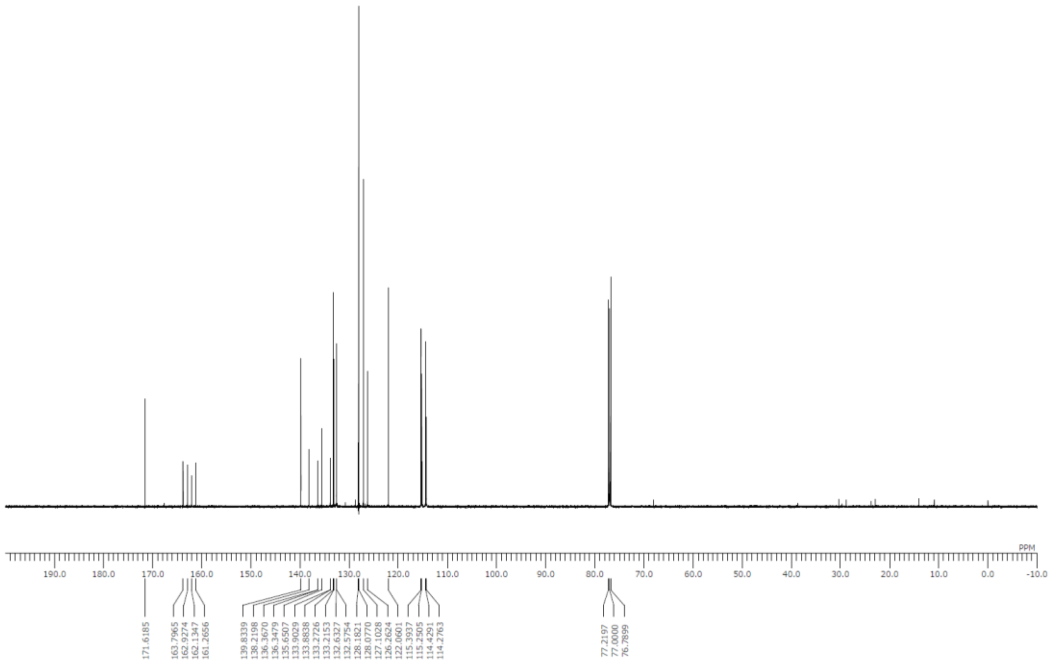

^{19}F-NMR spectrum of **2d** ($CDCl_3$, 565 MHz)

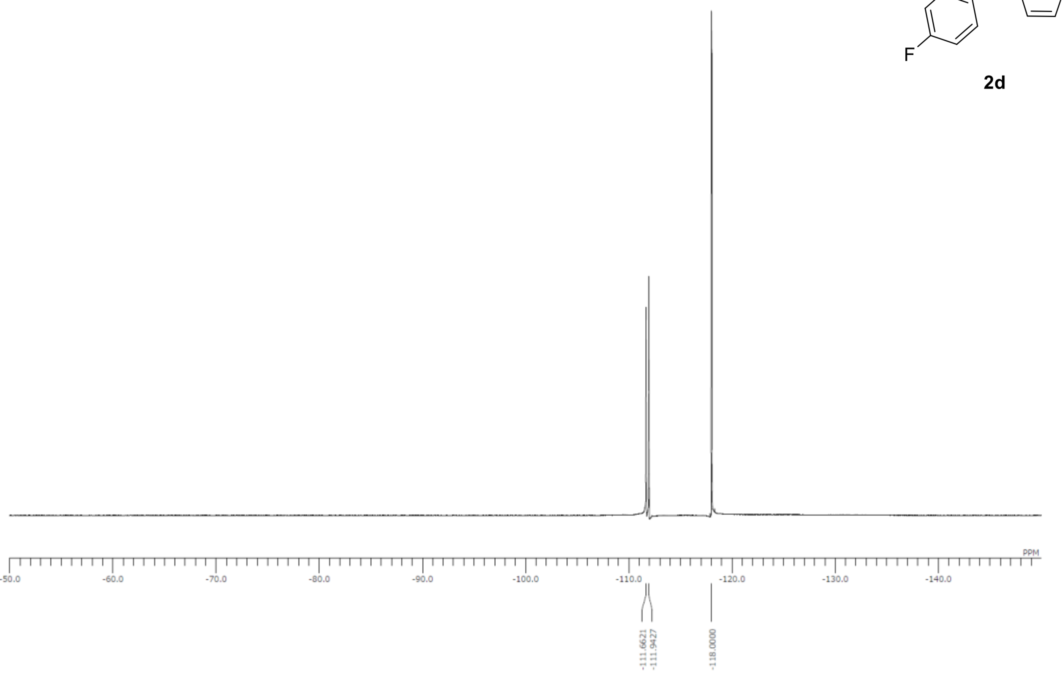

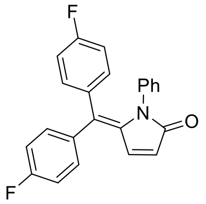

2d

IR spectrum of **2d**

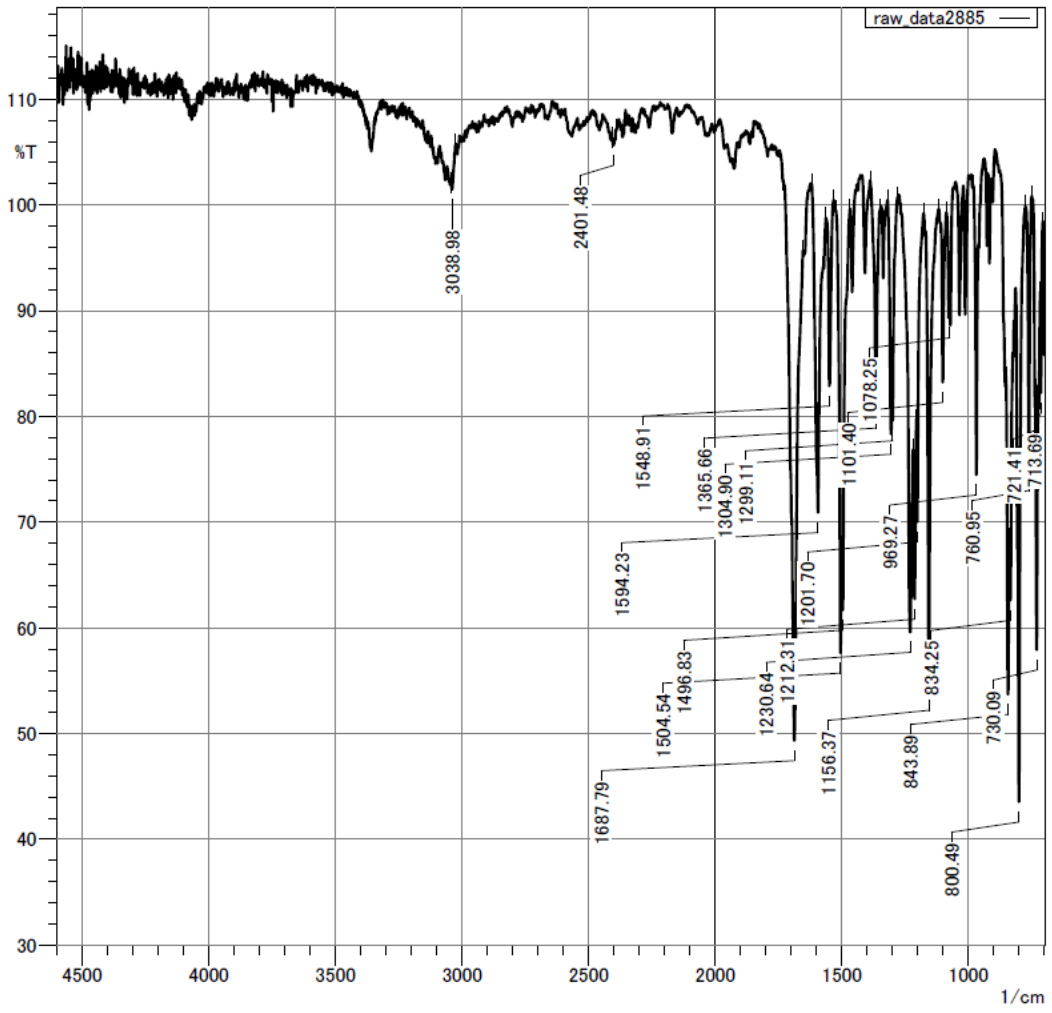

HRMS spectrum of **2d**

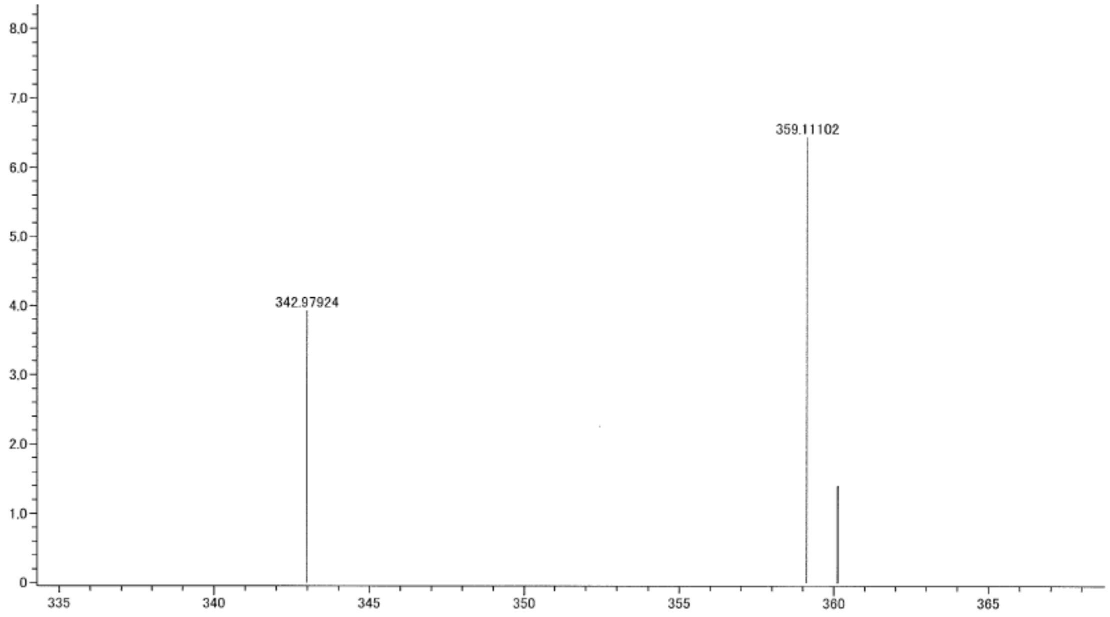

^{1}H-NMR spectrum of **2e** ($CDCl_3$, 600 MHz)

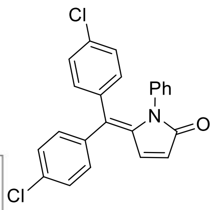

2e

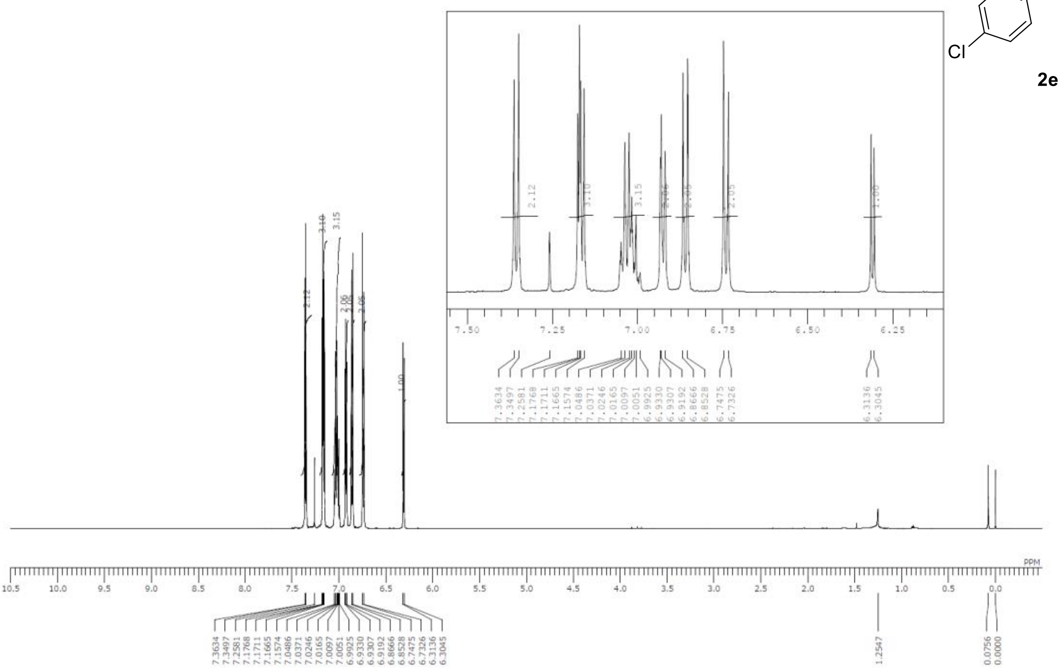

^{13}C-NMR spectrum of **2e** ($CDCl_3$, 150 MHz)

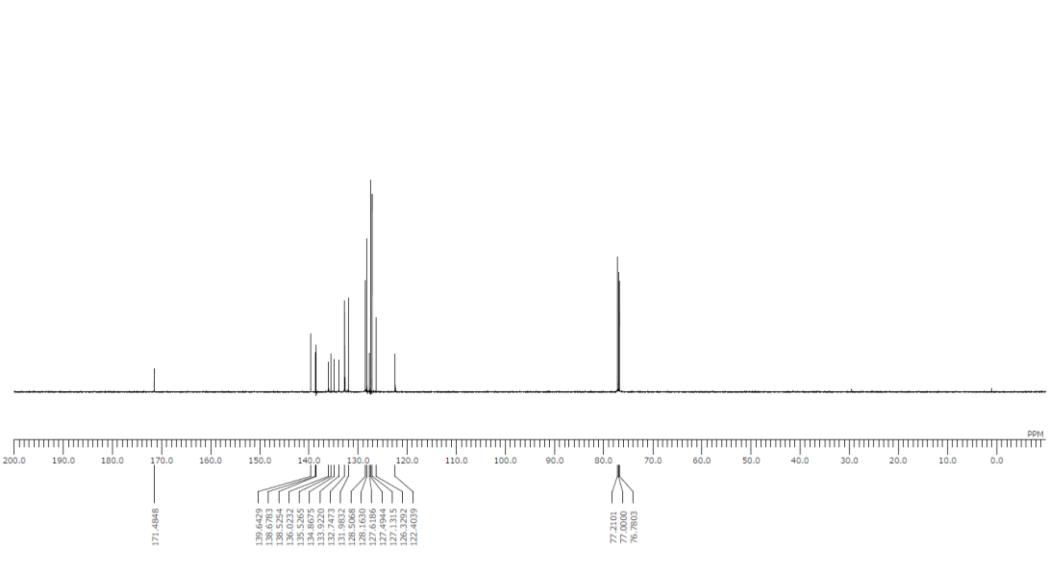

IR spectrum of **2e**

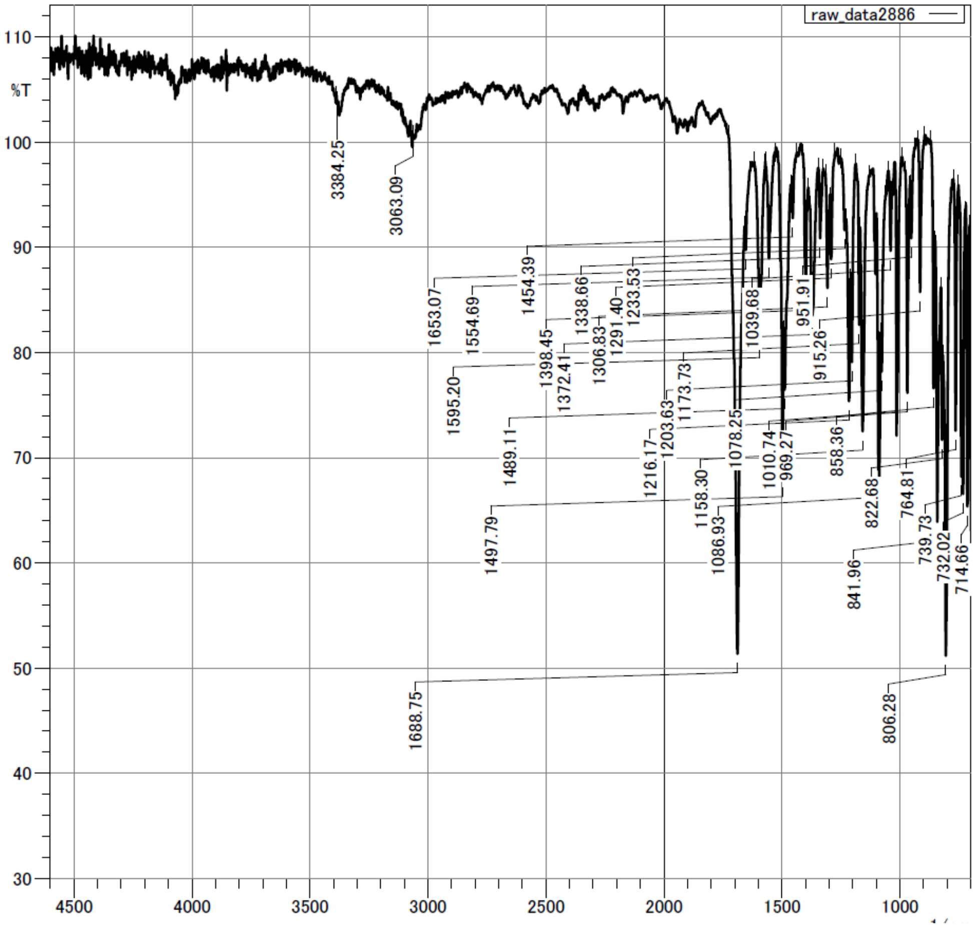

HRMS spectrum of **2e**

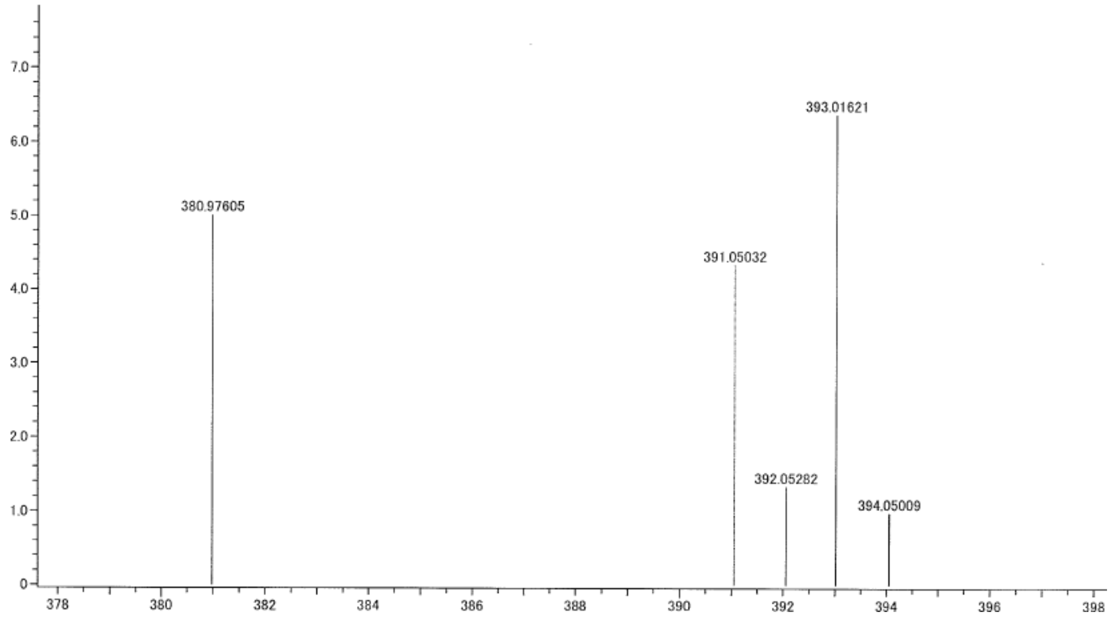

^{1}H-NMR spectrum of **2f** ($CDCl_3$, 600 MHz)

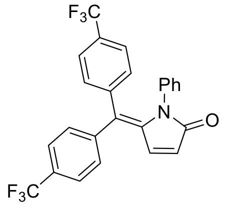

2f

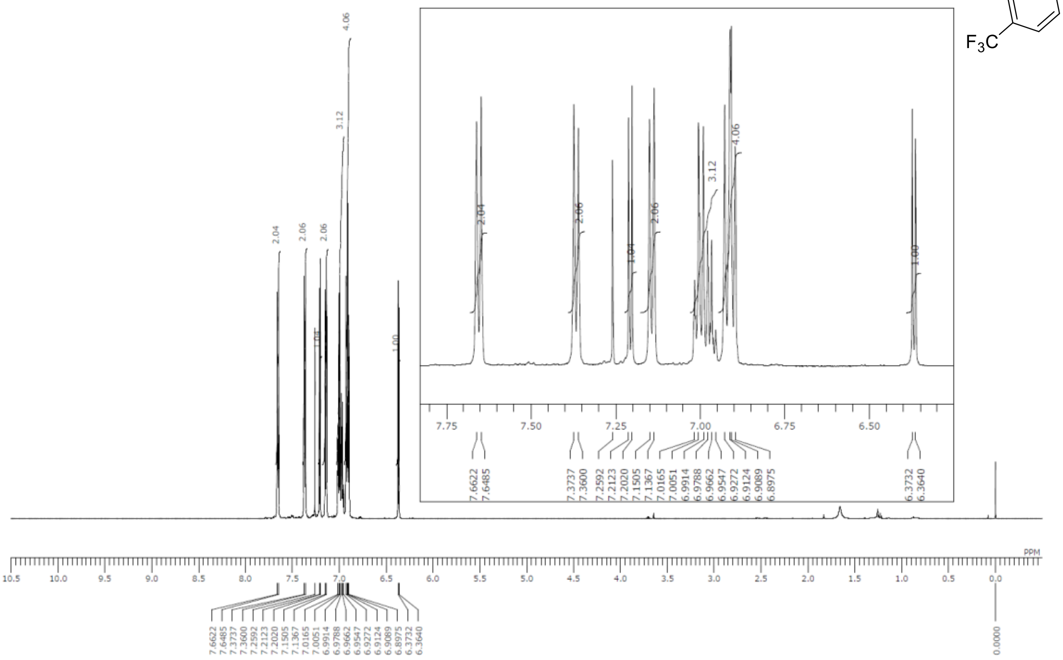

^{13}C-NMR spectrum of **2f** ($CDCl_3$, 150 MHz)

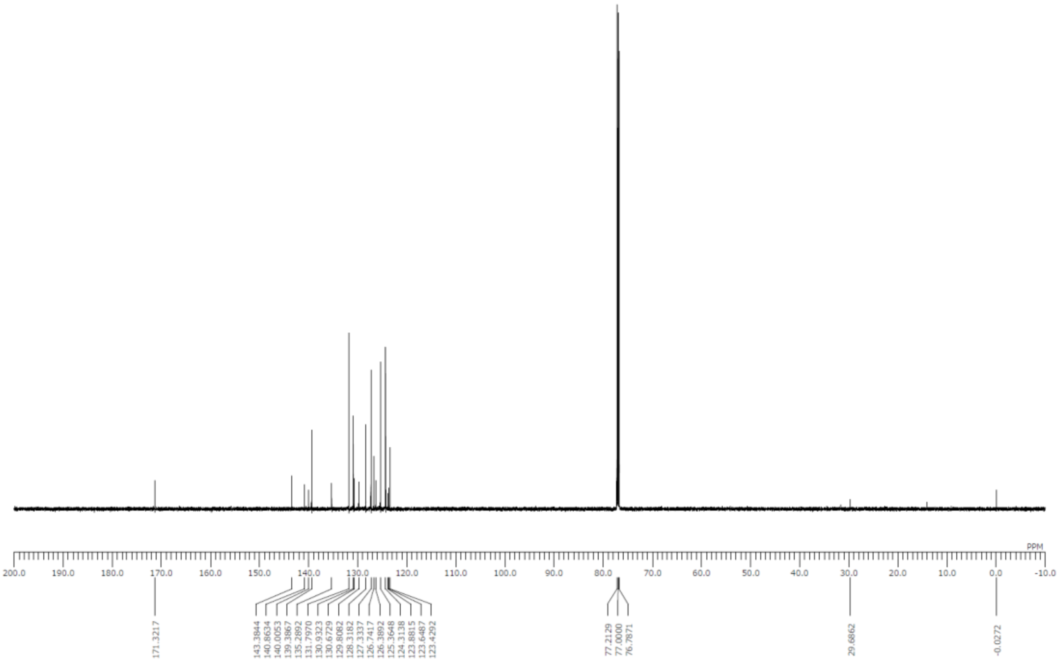

^{19}F-NMR spectrum of **2f** ($CDCl_3$, 565 MHz)

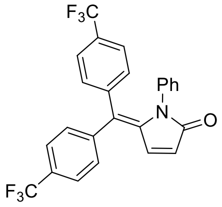

2f

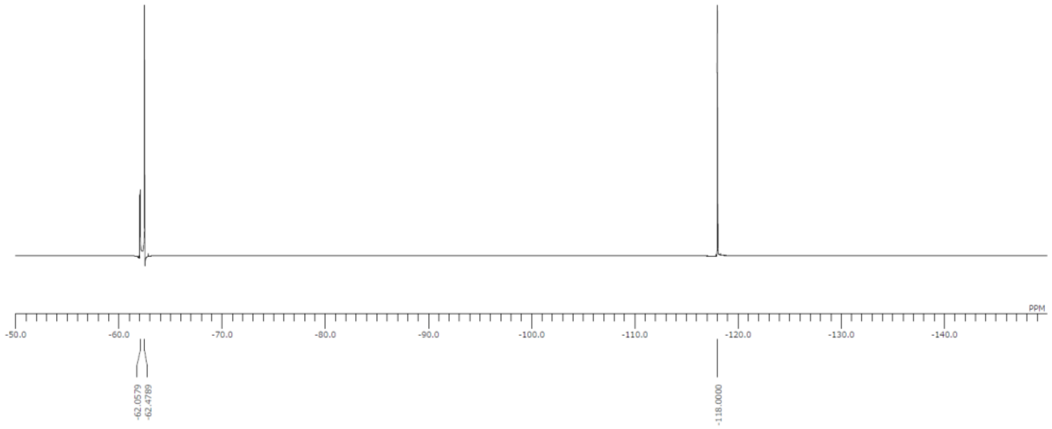

IR spectrum of **2f**

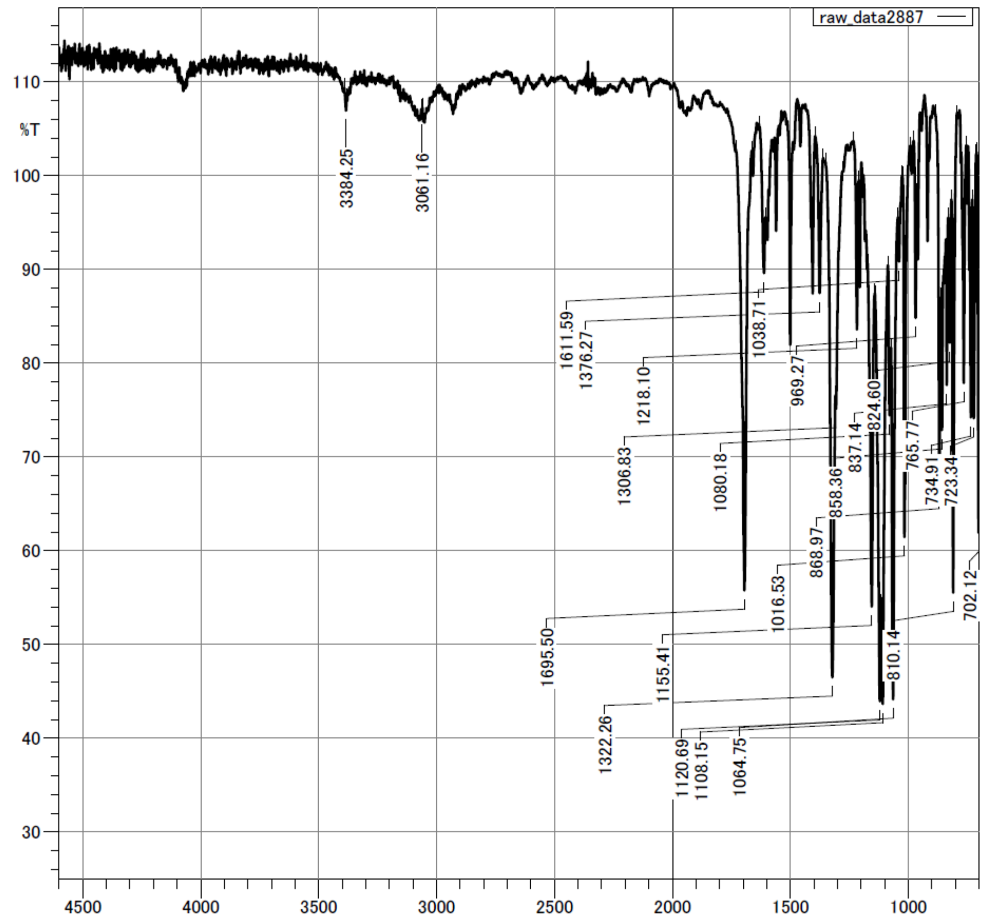

HRMS spectrum of **2f**

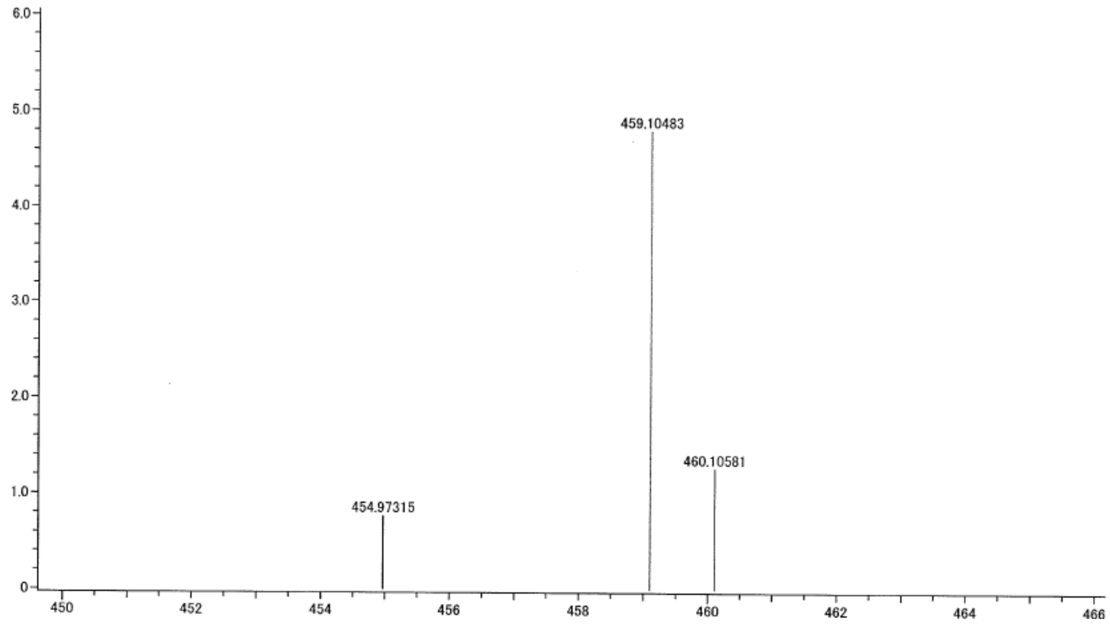

^{1}H-NMR spectrum of **2g** ($CDCl_3$, 600 MHz)

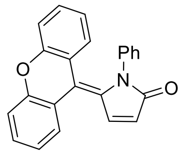

2g

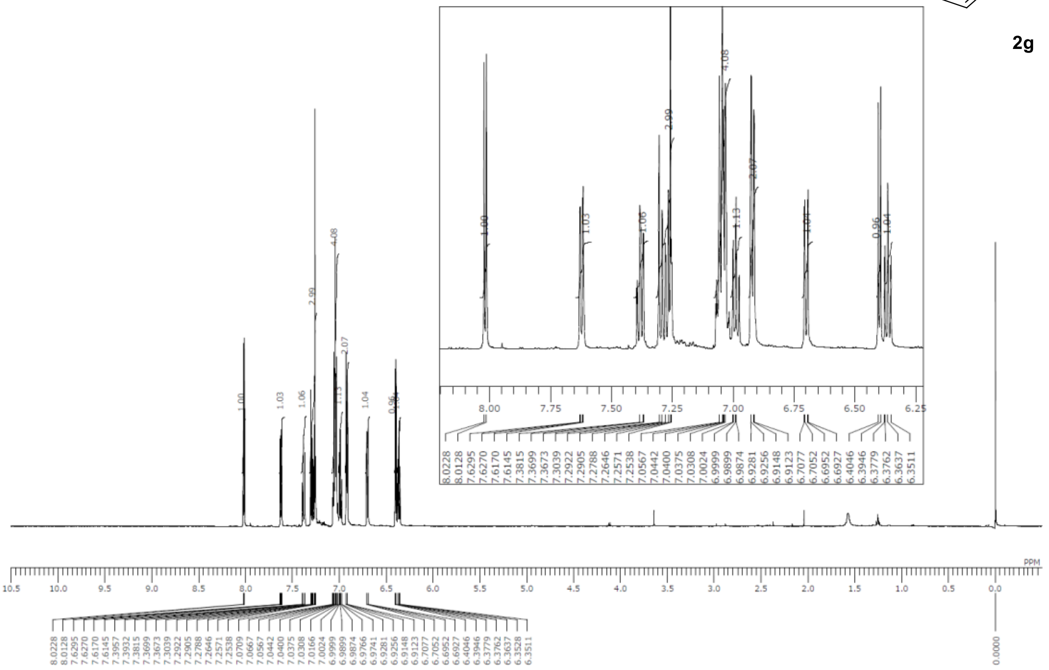

^{13}C-NMR spectrum of **2g** ($CDCl_3$, 150 MHz)

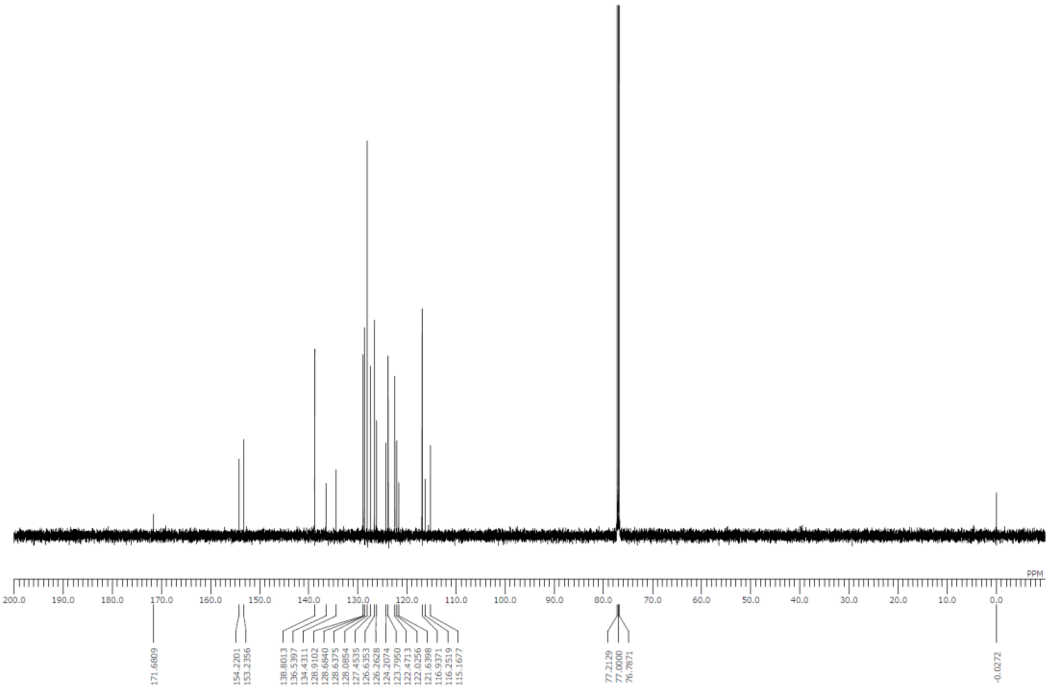

IR spectrum of **2g**

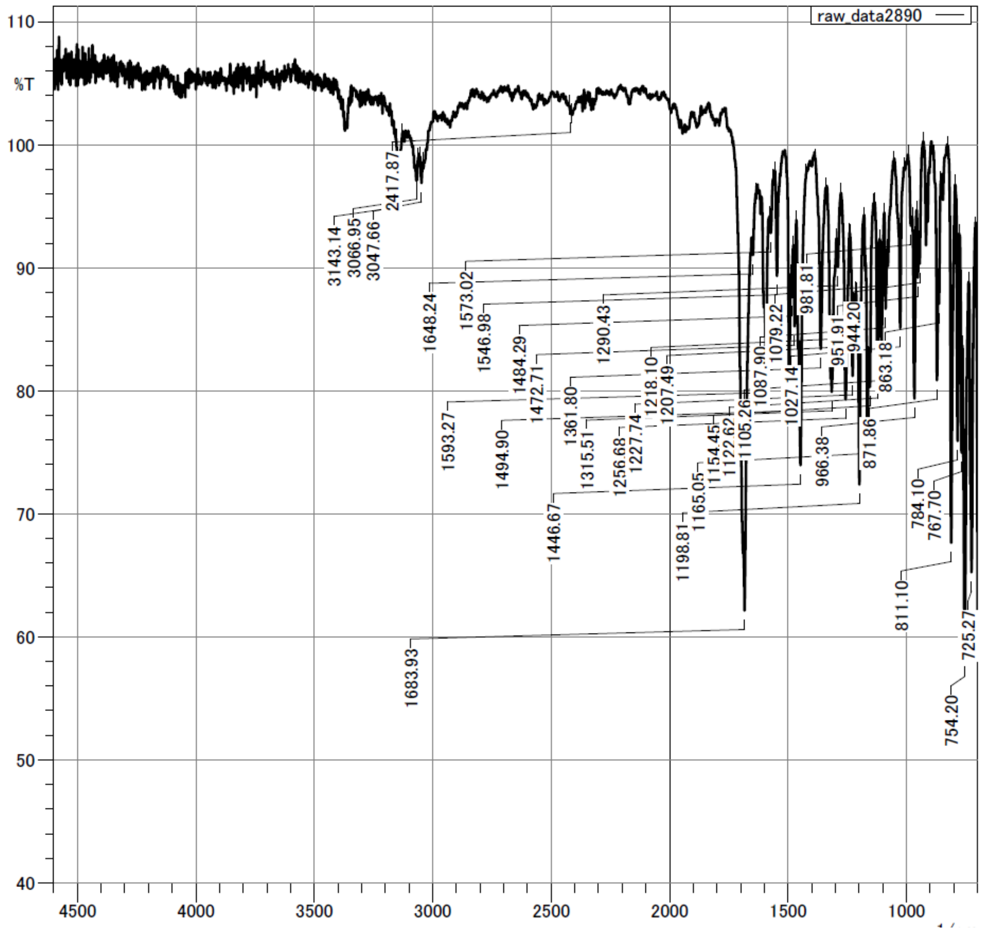

HRMS spectrum of **2g**

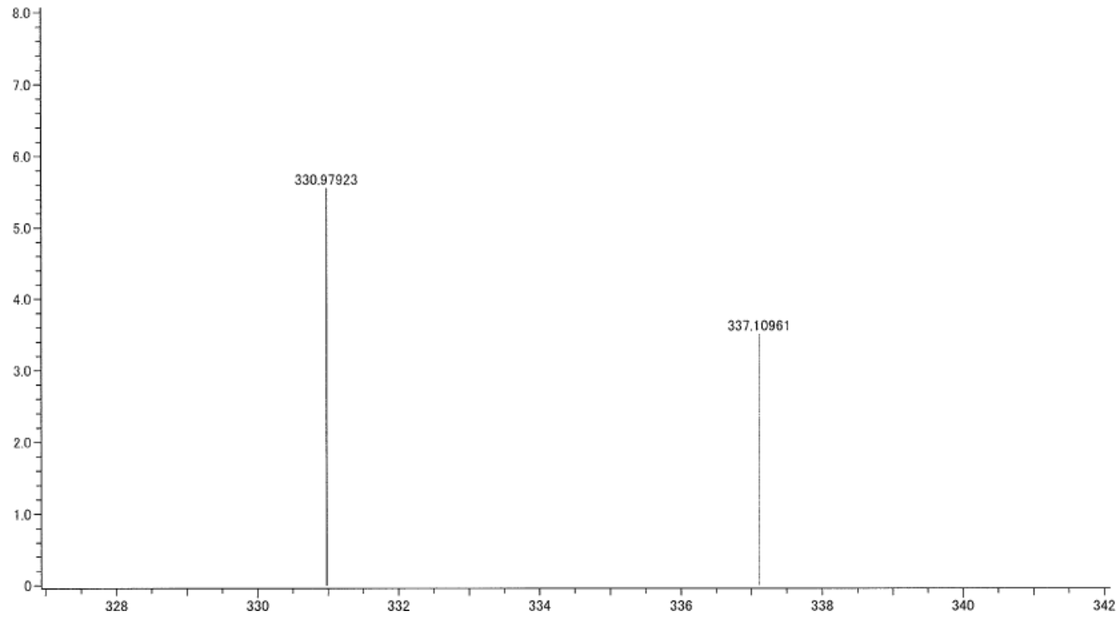

^{1}H-NMR spectrum of **2h** ($CDCl_3$, 600 MHz)

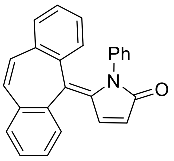

2h

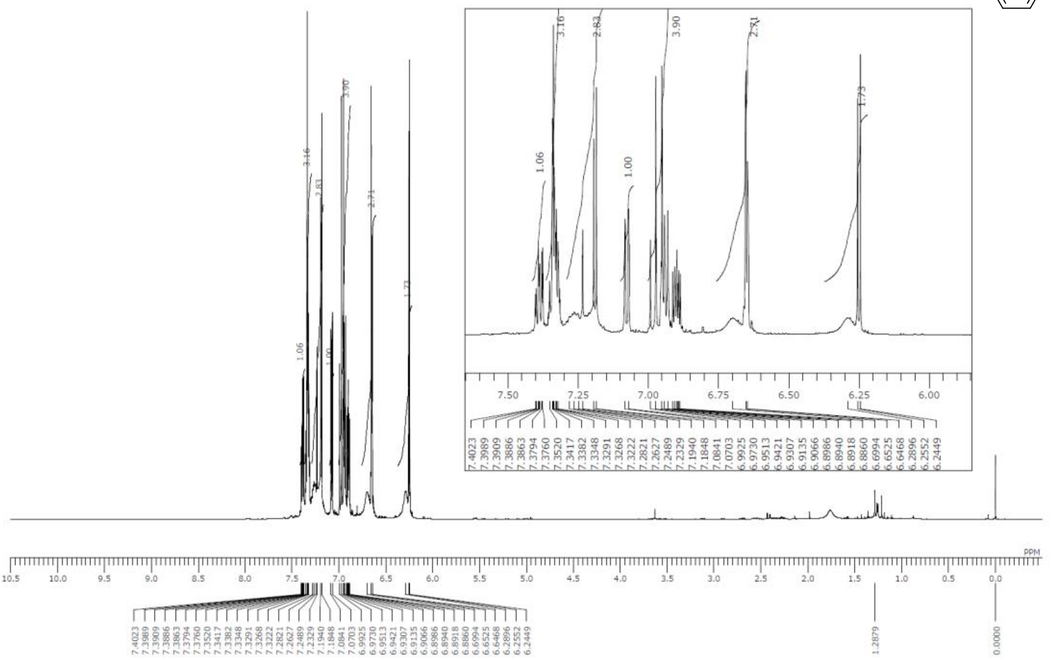

^{13}C-NMR spectrum of **2h** ($CDCl_3$, 150 MHz)

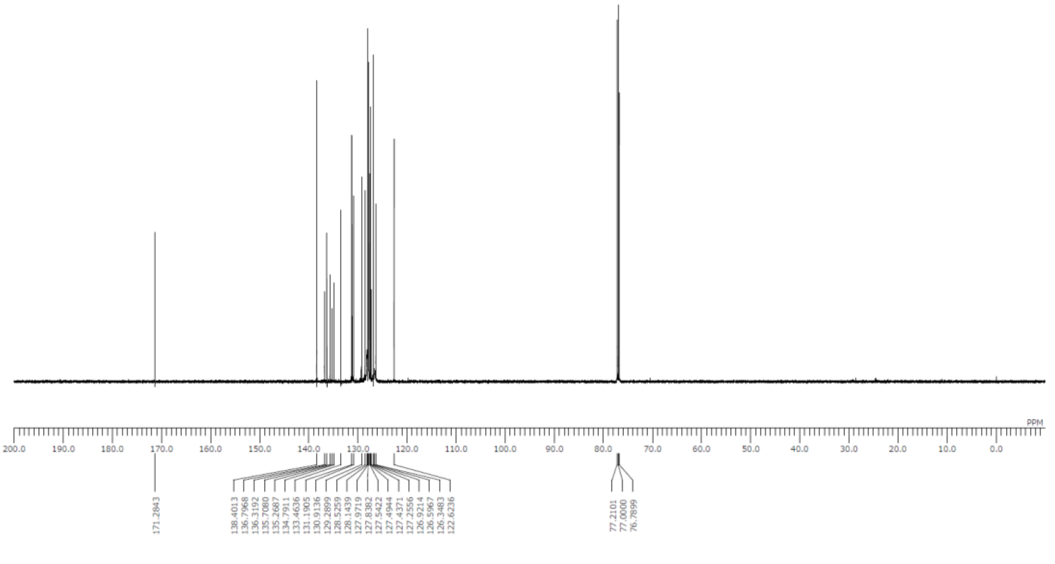

IR spectrum of **2h**

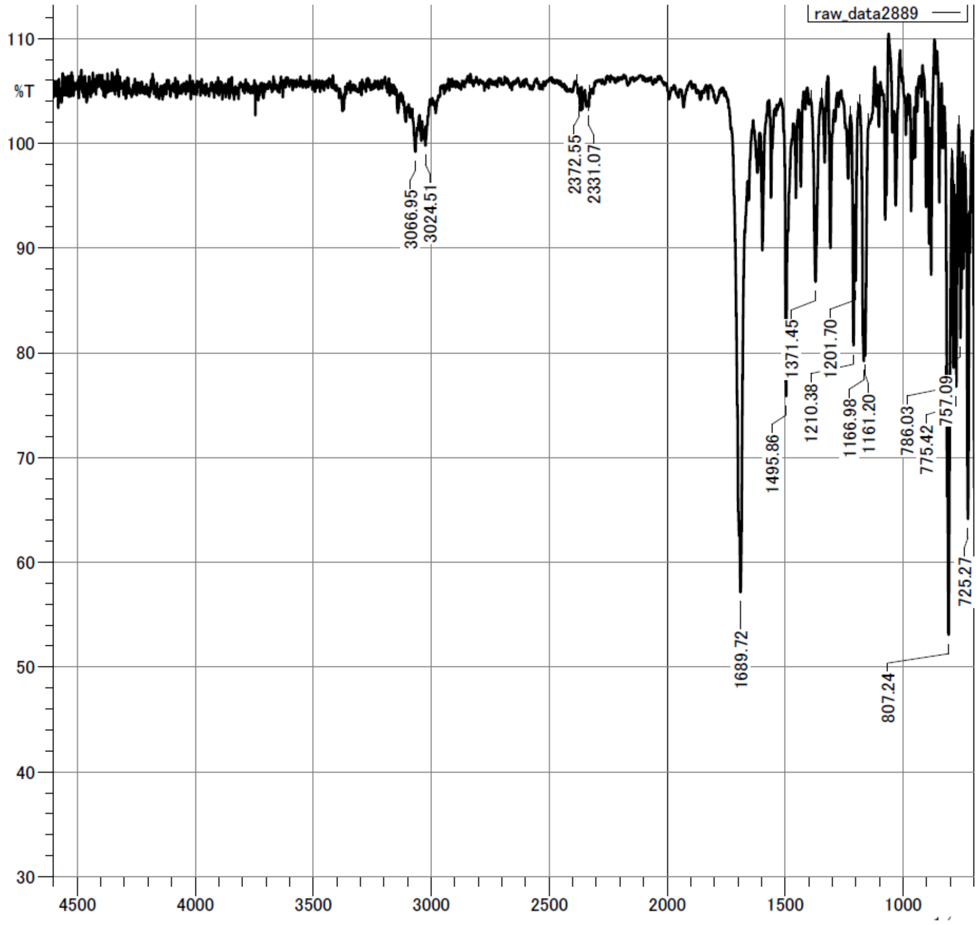

HRMS spectrum of **2h**

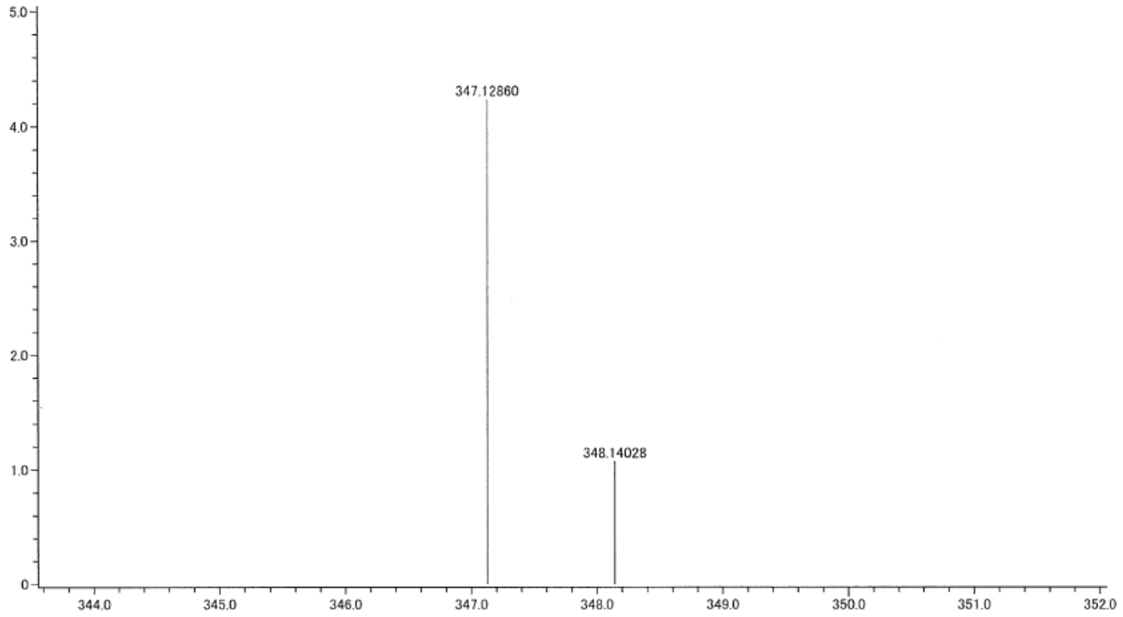

^{1}H-NMR spectrum of **2i** ($CDCl_3$, 600 MHz)

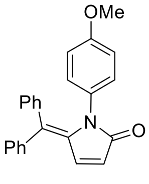

2i

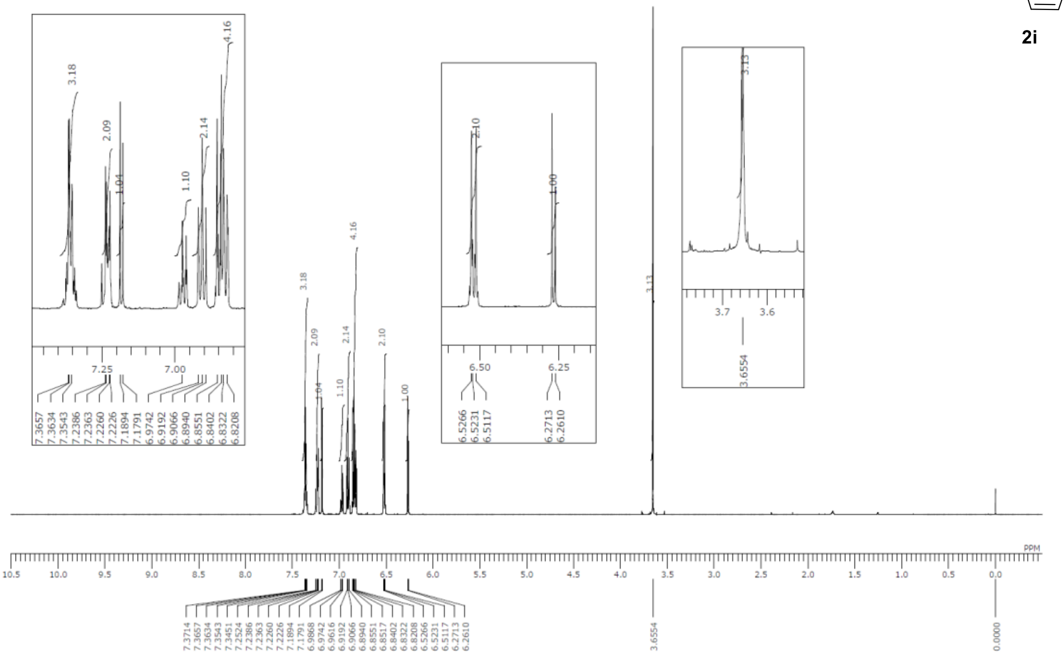

^{13}C-NMR spectrum of **2i** ($CDCl_3$, 150 MHz)

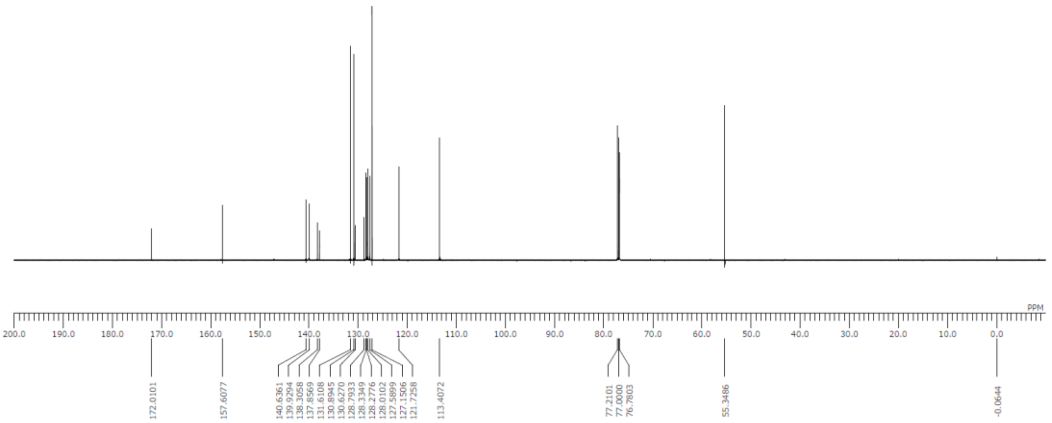

IR spectrum of **2i**

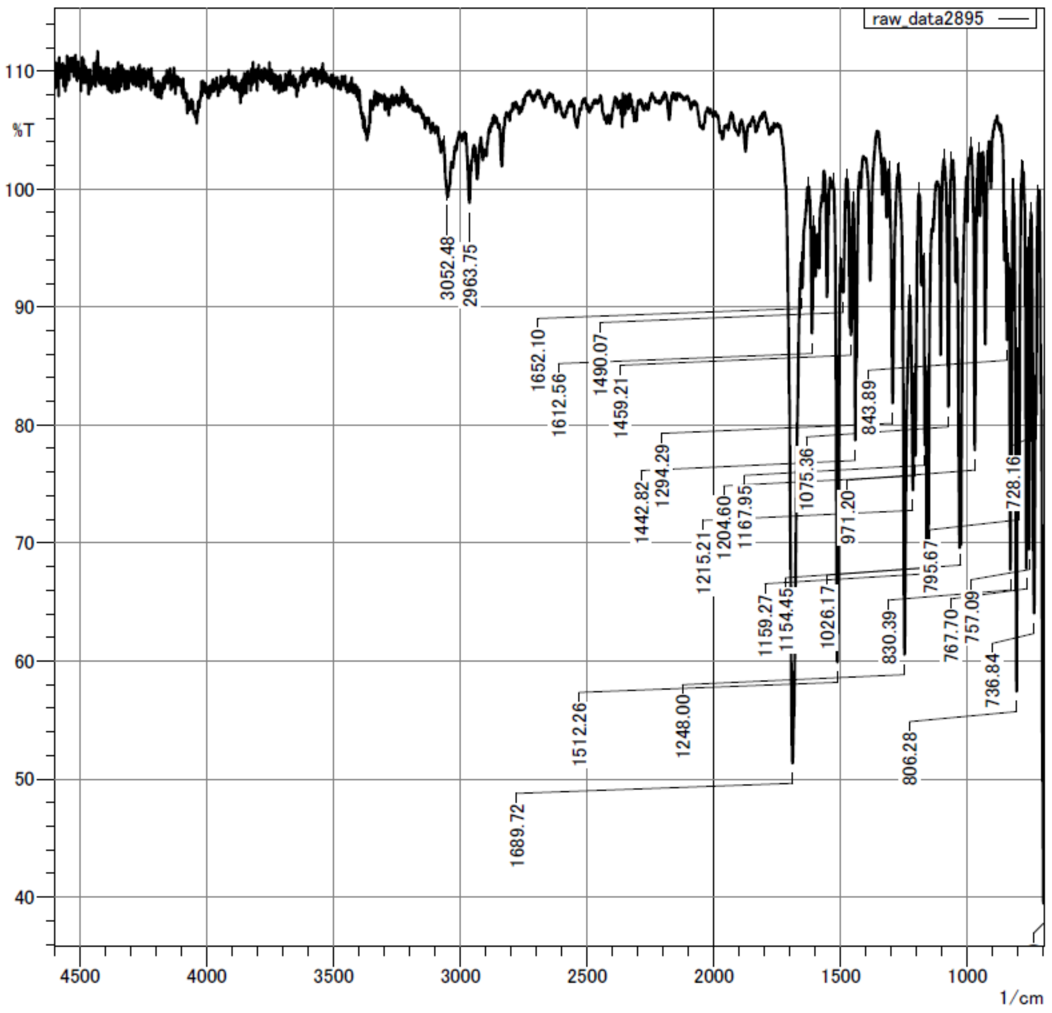

HRMS spectrum of **2i**

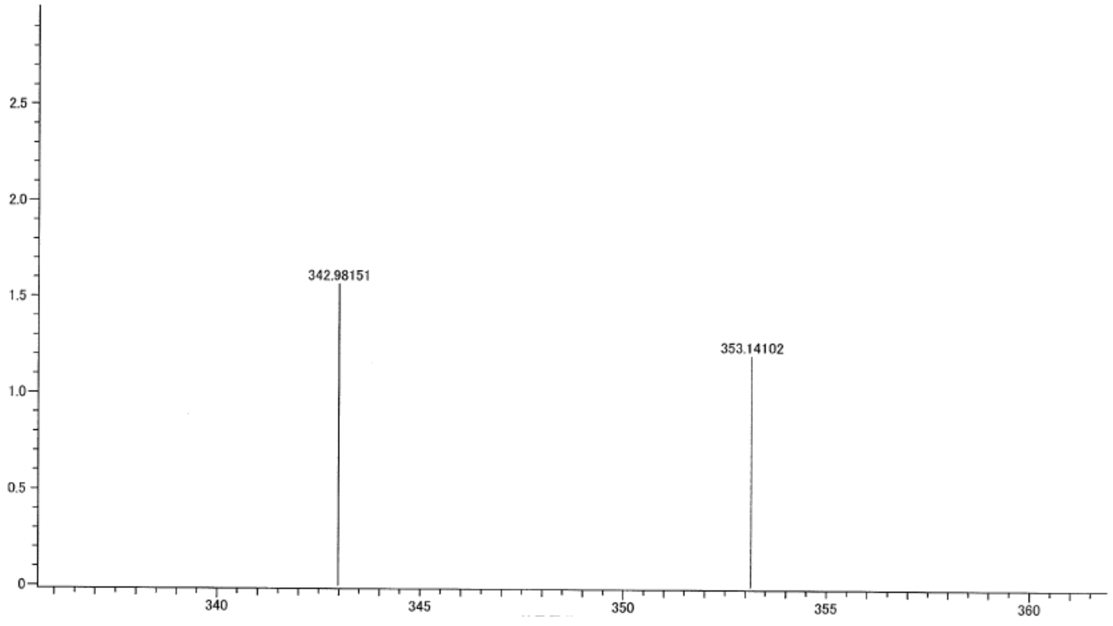

^{1}H-NMR spectrum of **2j** ($CDCl_3$, 600 MHz)

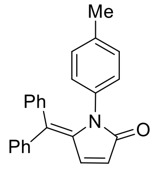

2j

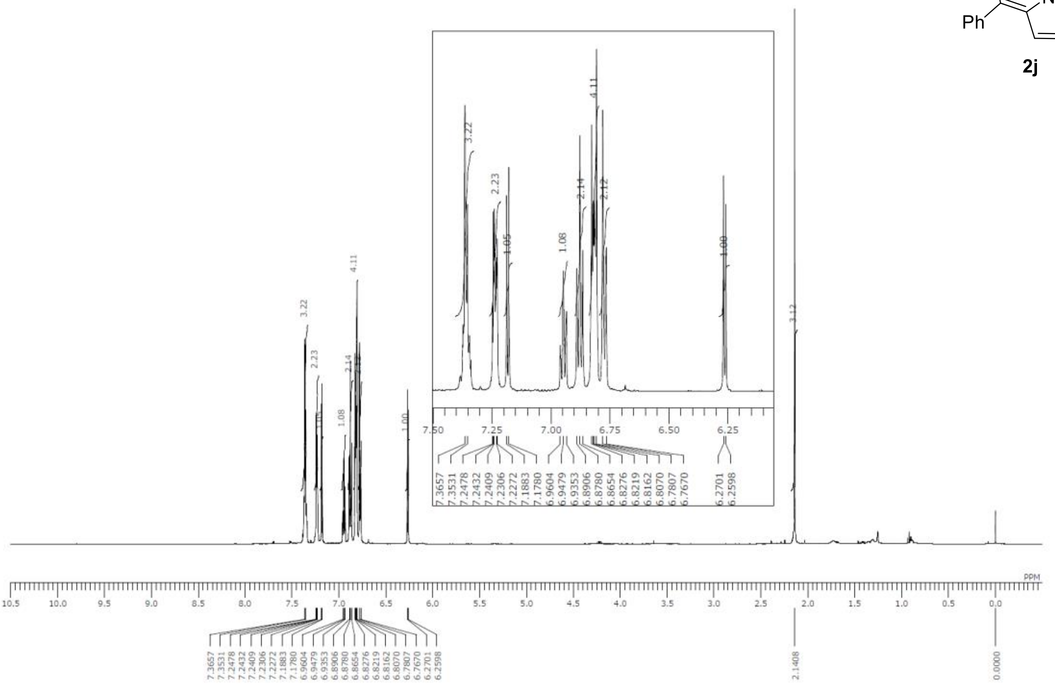

^{13}C-NMR spectrum of **2j** ($CDCl_3$, 150 MHz)

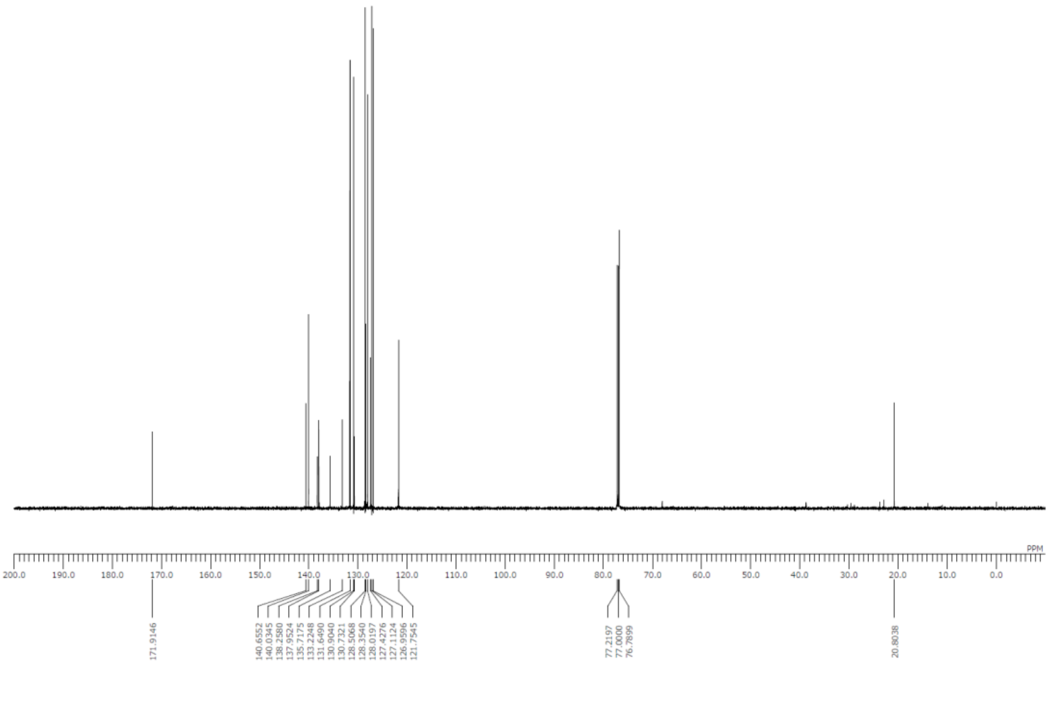

IR spectrum of **2j**

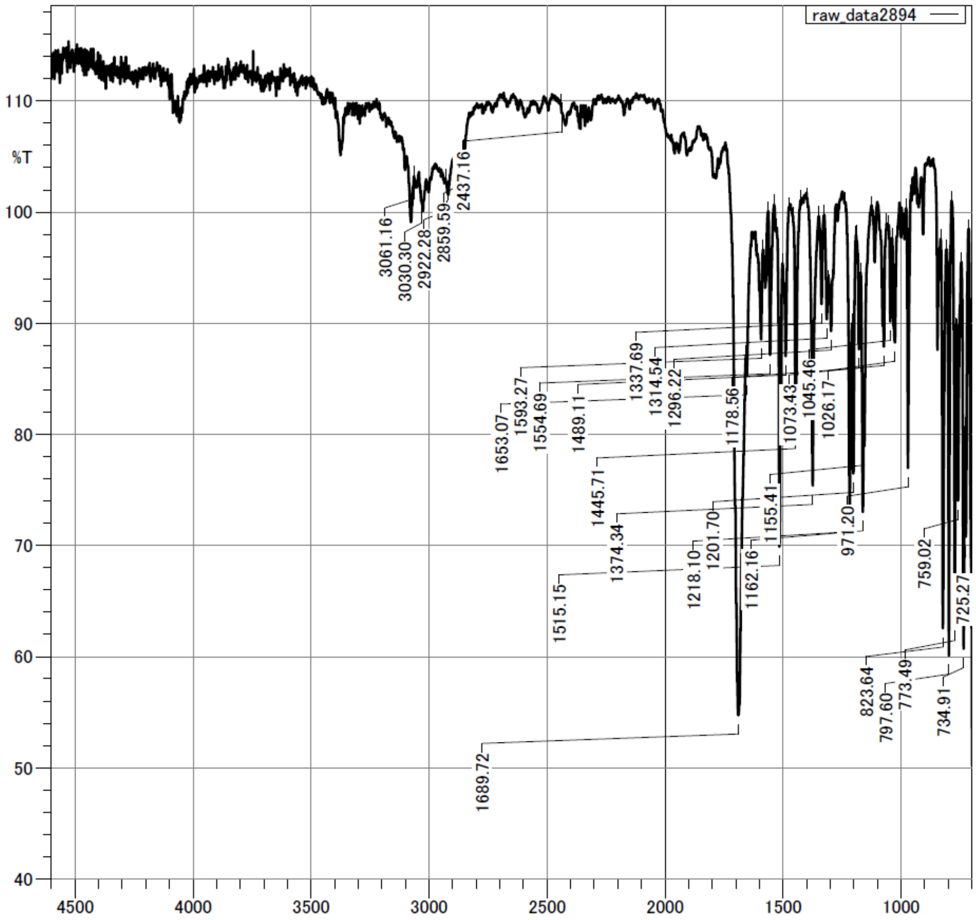

HRMS spectrum of **2j**

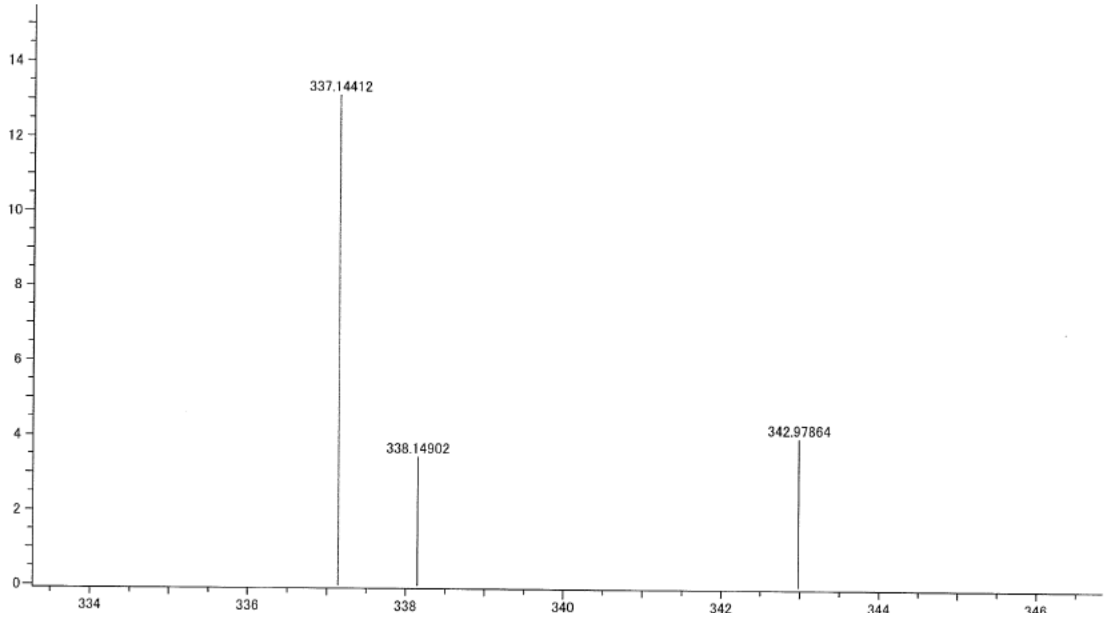

^{1}H-NMR spectrum of **2k** ($CDCl_3$, 600 MHz)

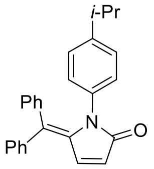

2k

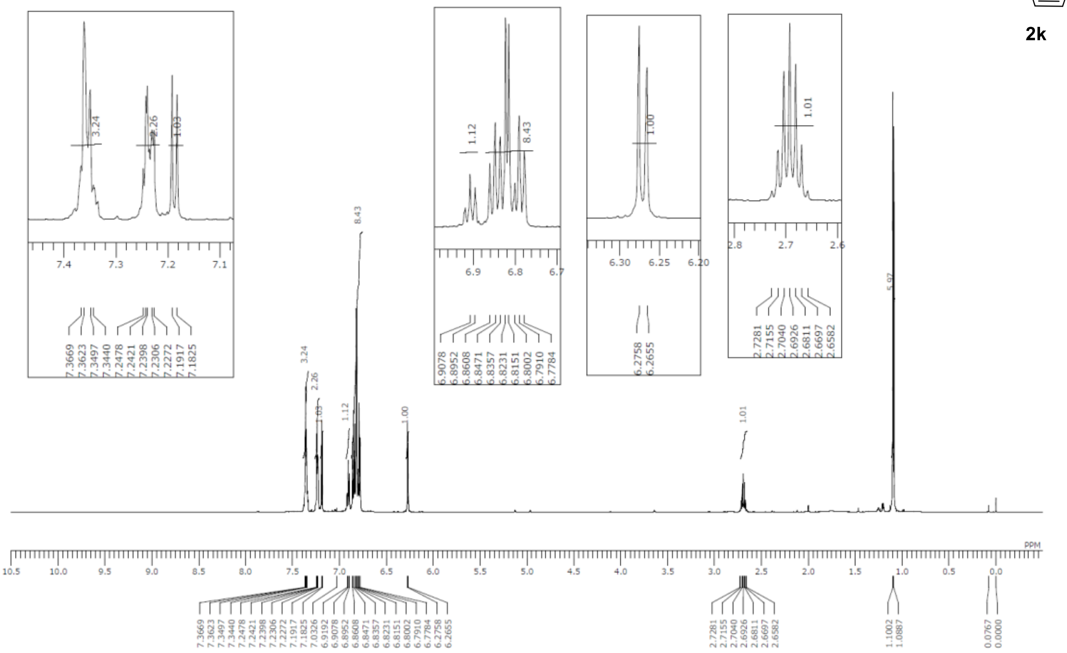

^{13}C-NMR spectrum of **2k** ($CDCl_3$, 150 MHz)

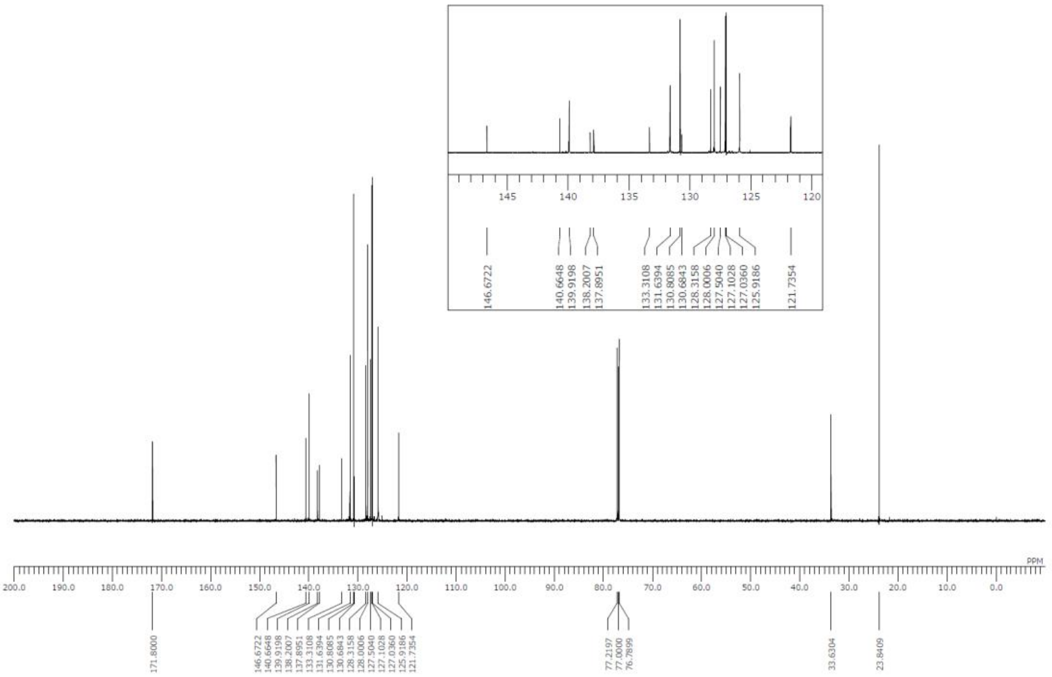

IR spectrum of **2k**

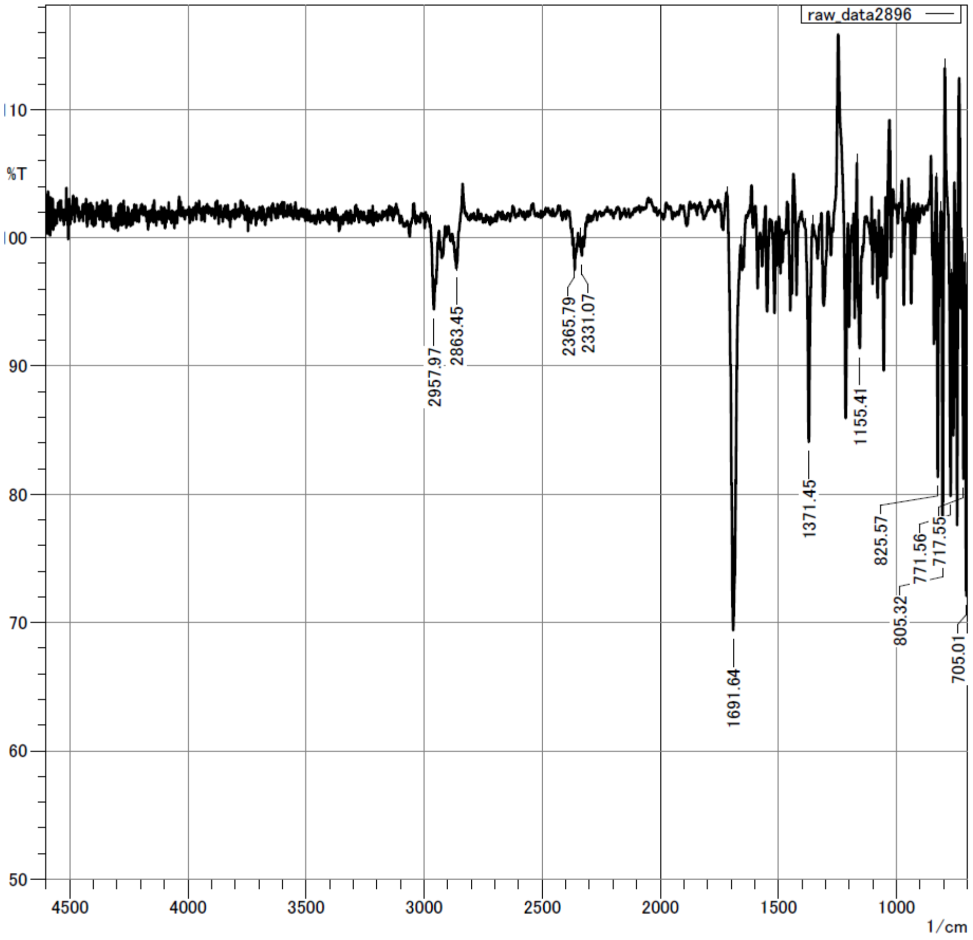

HRMS spectrum of **2k**

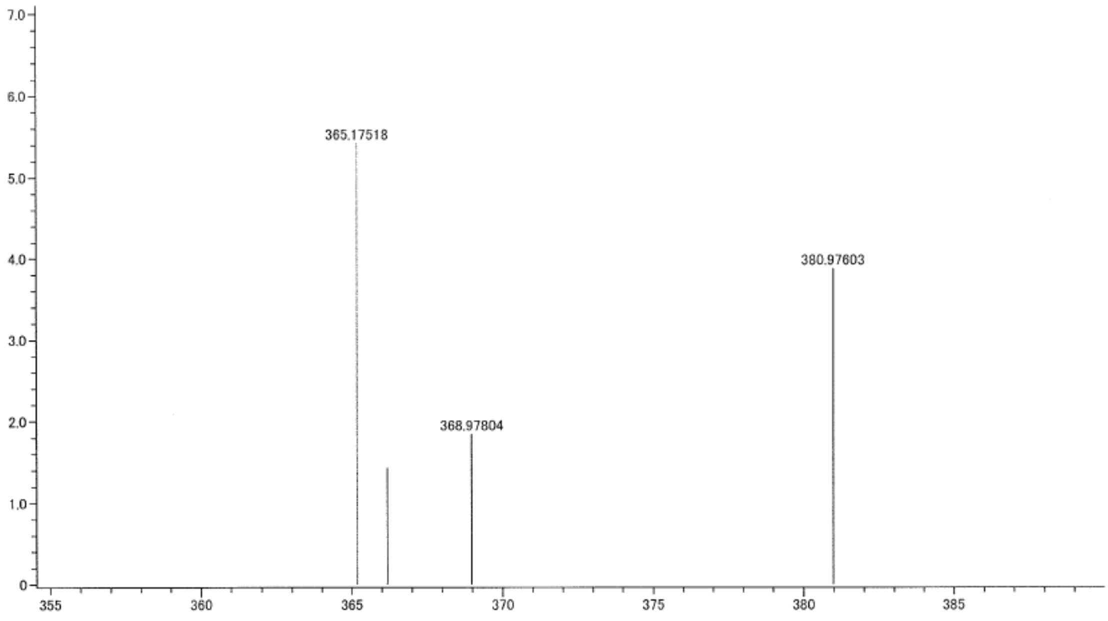

^{1}H-NMR spectrum of **2l** ($CDCl_3$, 600 MHz)

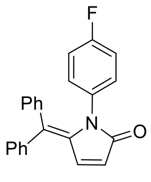

2l

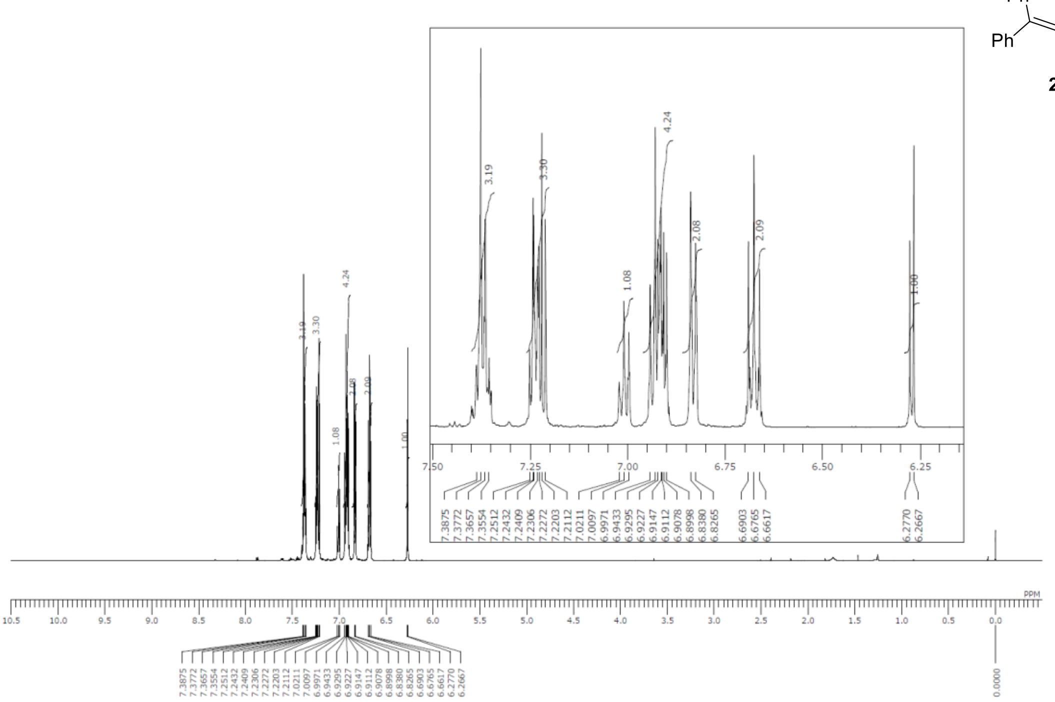

^{13}C-NMR spectrum of **2l** ($CDCl_3$, 150 MHz)

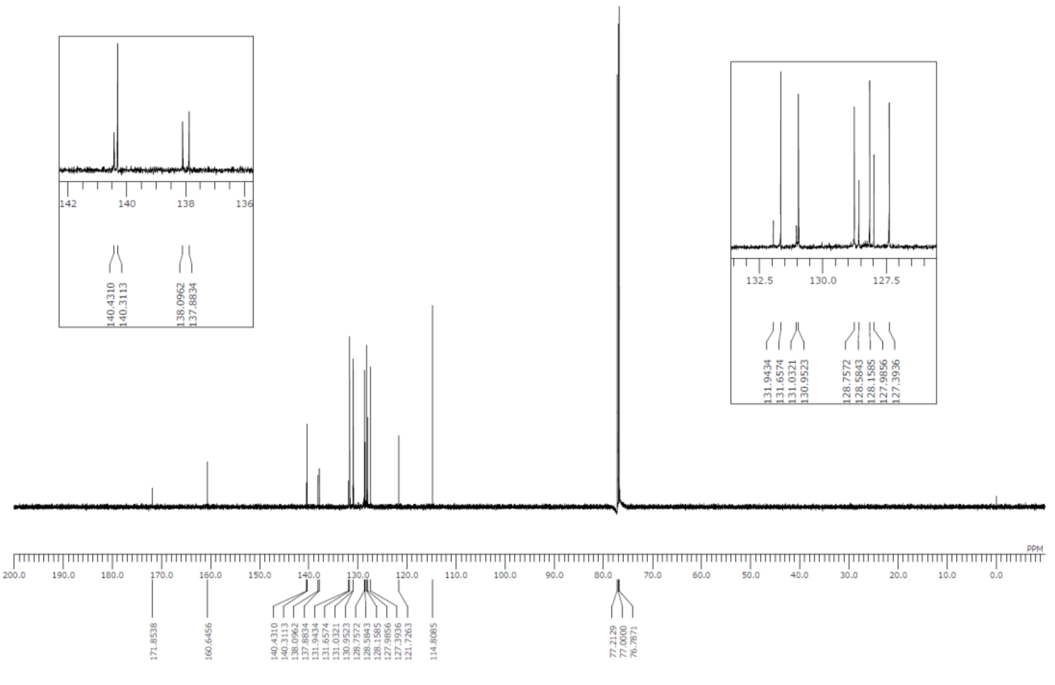

^{19}F-NMR spectrum of **2l** ($CDCl_3$, 565 MHz)

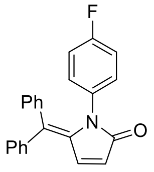

2l

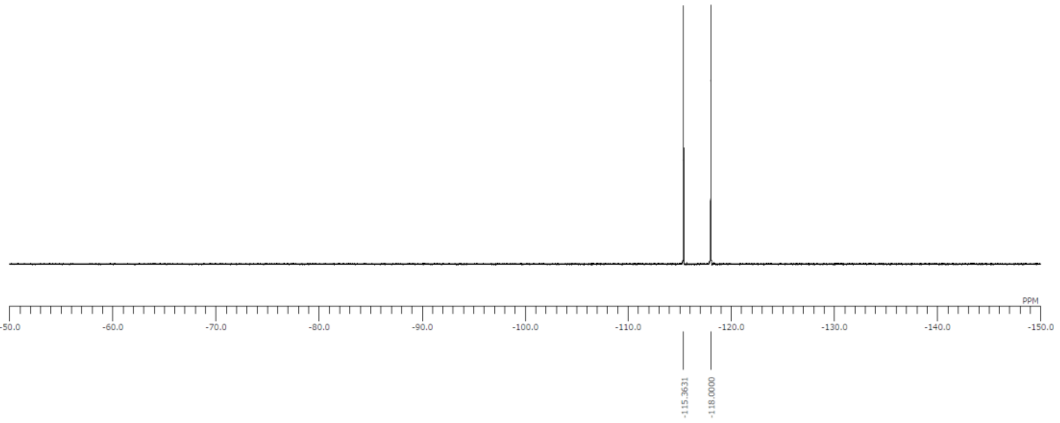

IR spectrum of **2l**

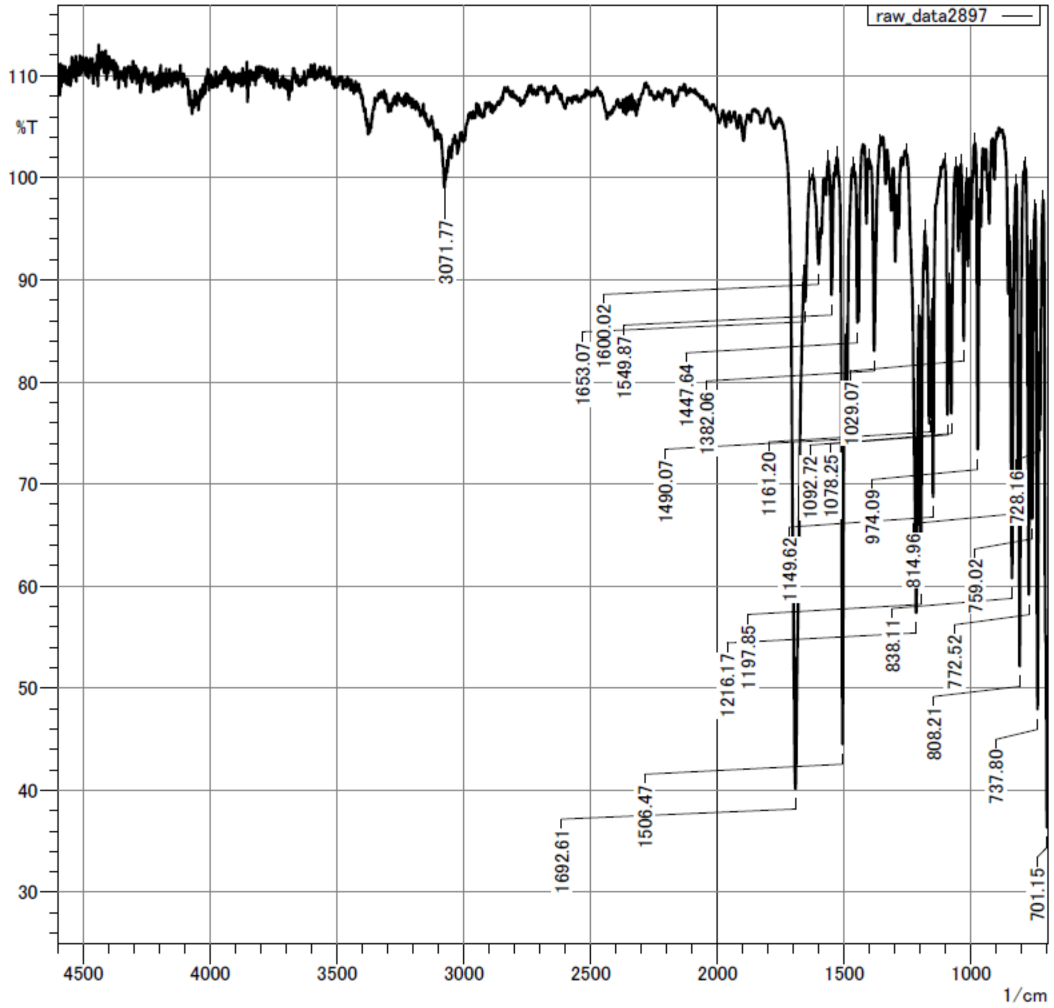

HRMS spectrum of **2l**

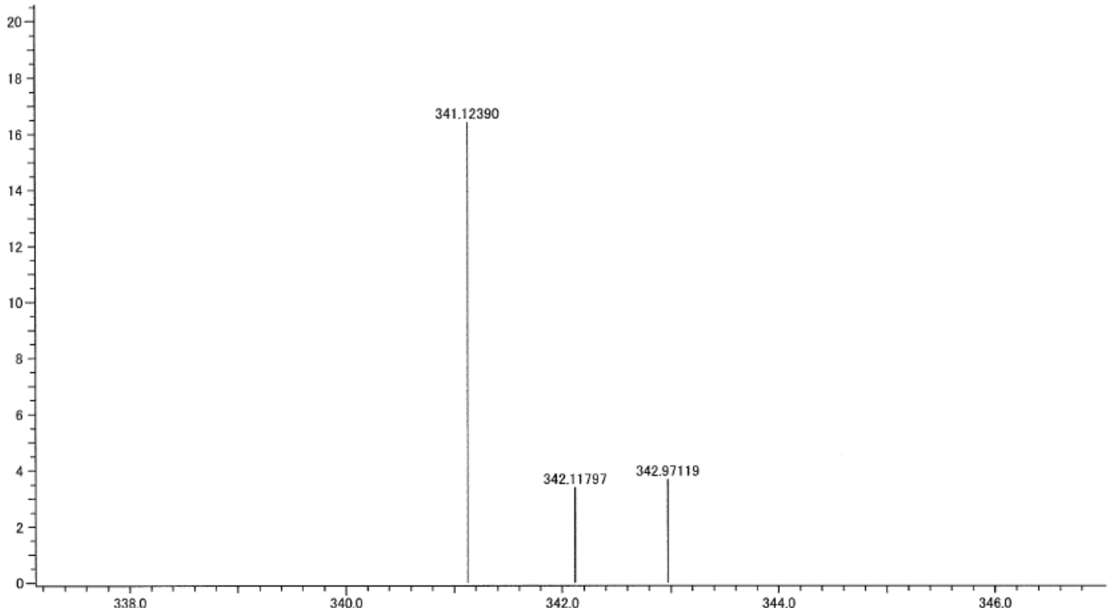

^{1}H-NMR spectrum of **2m** ($CDCl_3$, 600 MHz)

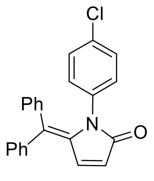

2m

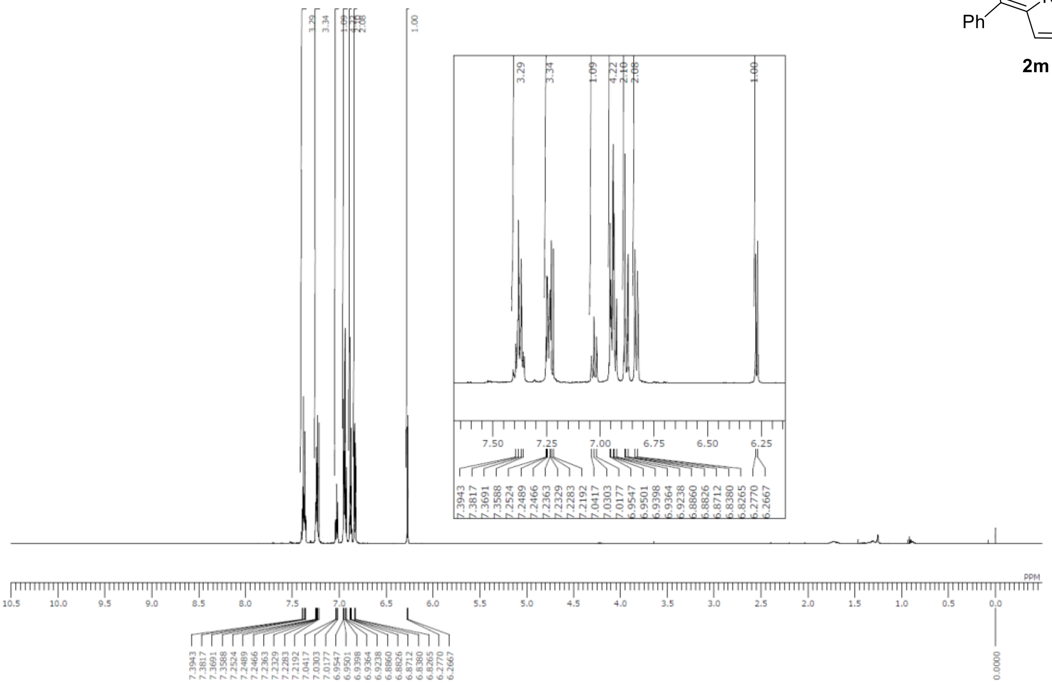

^{13}C-NMR spectrum of **2m** ($CDCl_3$, 150 MHz)

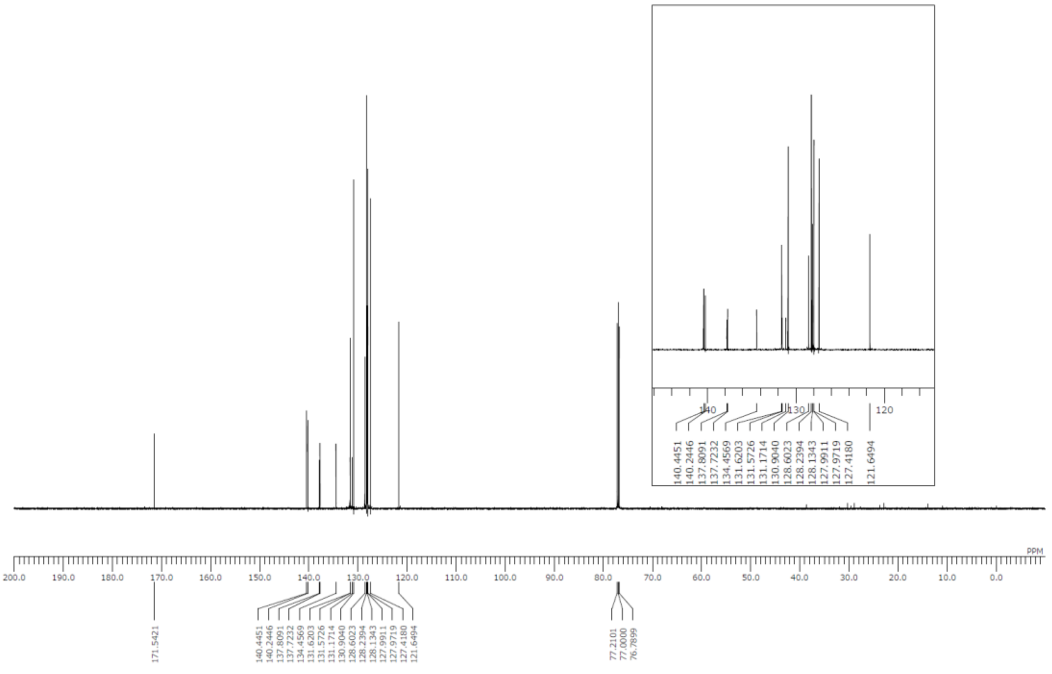

IR spectrum of **2m**

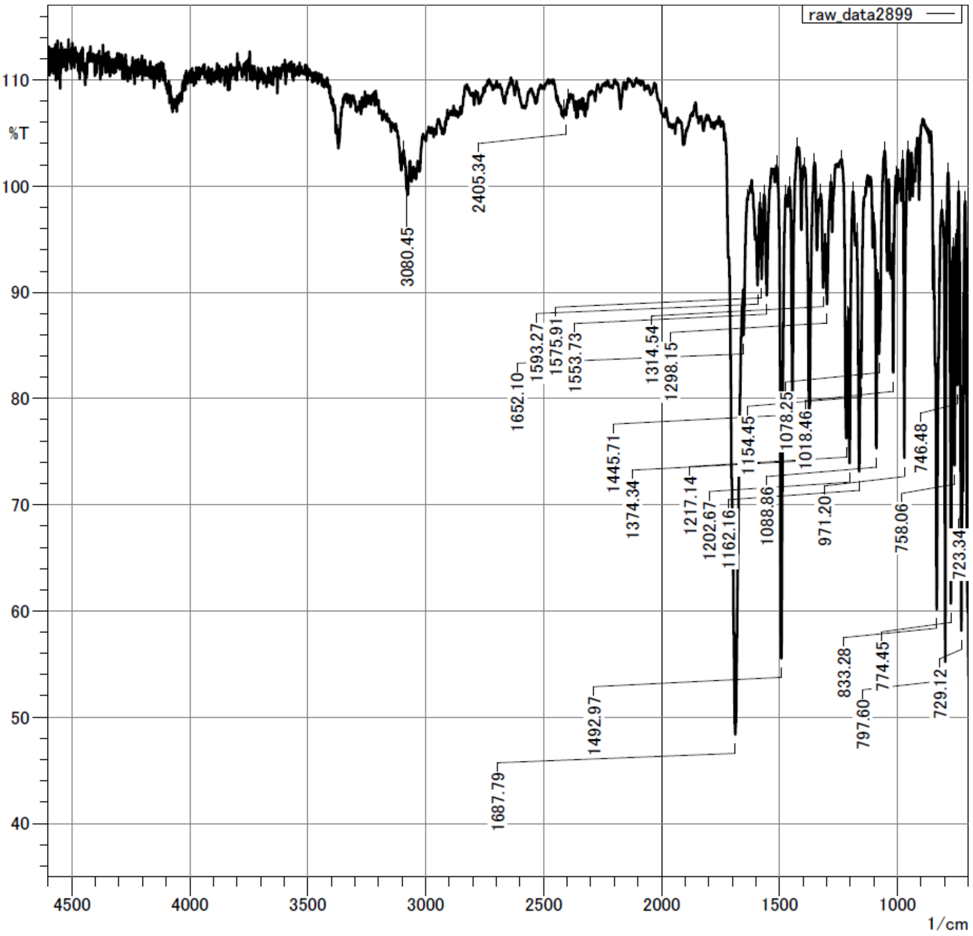

HRMS spectrum of **2m**

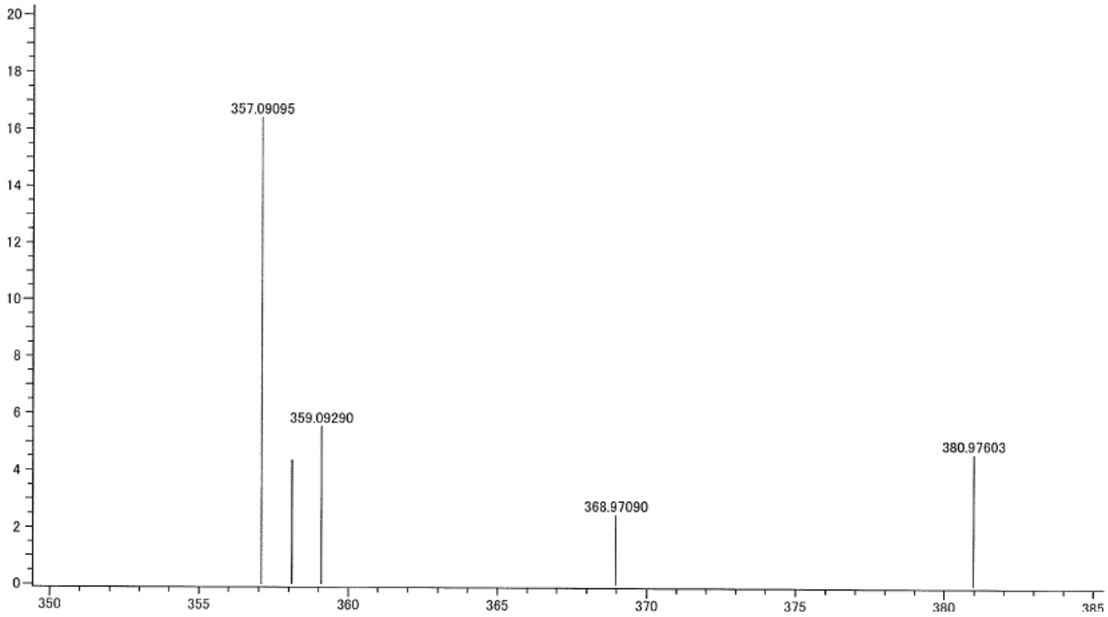

^{1}H-NMR spectrum of **2n** ($CDCl_3$, 600 MHz)

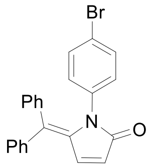

2n

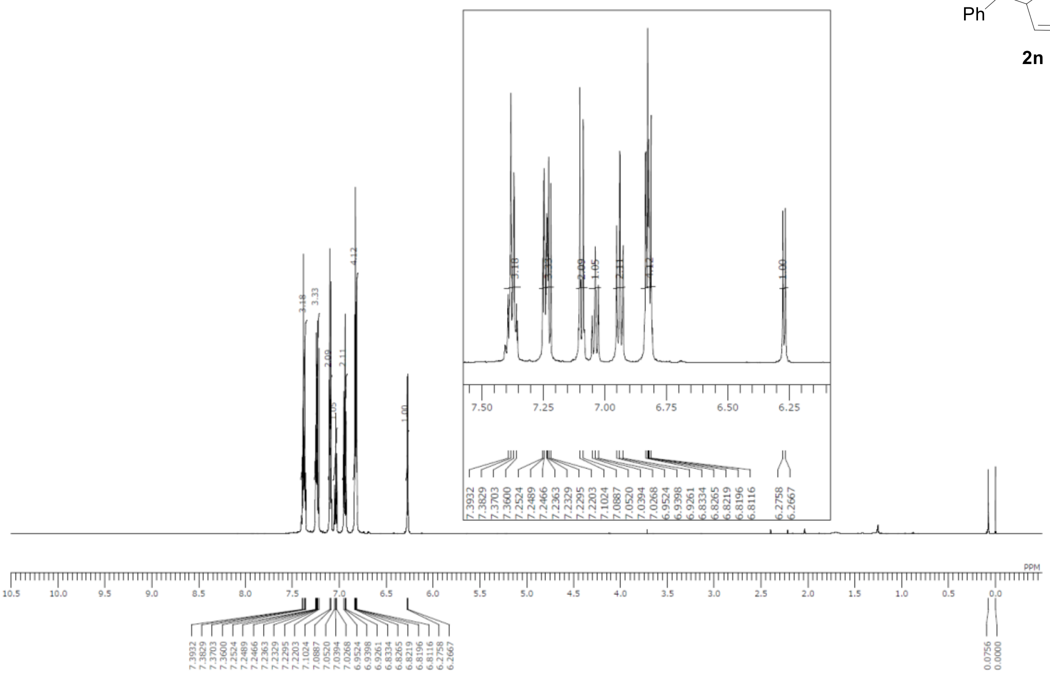

^{13}C-NMR spectrum of **2n** ($CDCl_3$, 150 MHz)

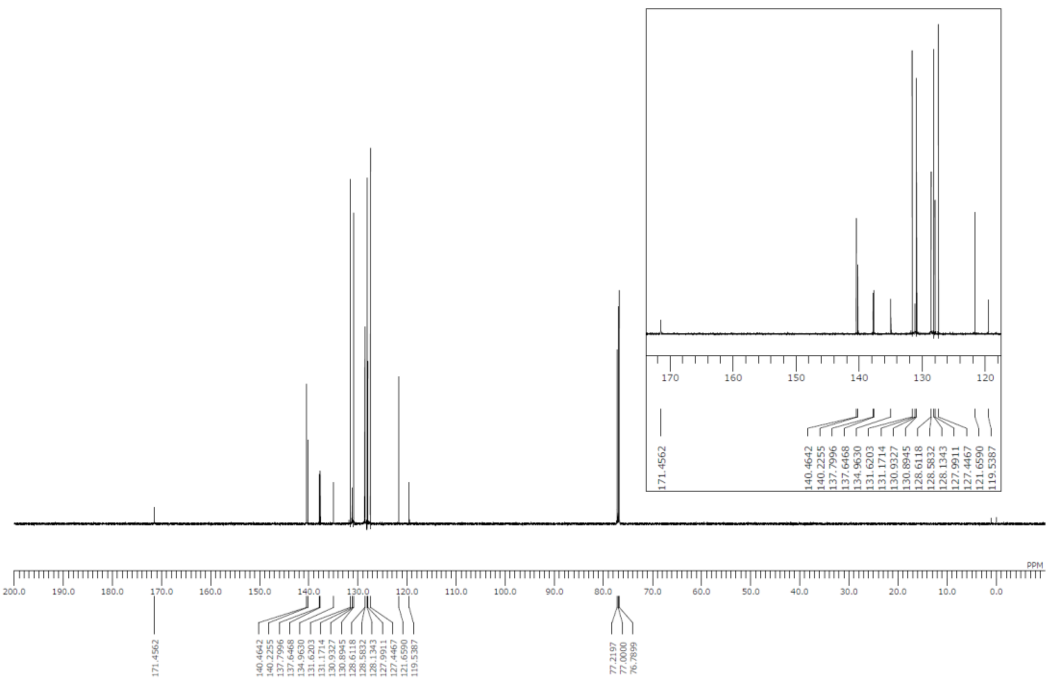

IR spectrum of **2n**

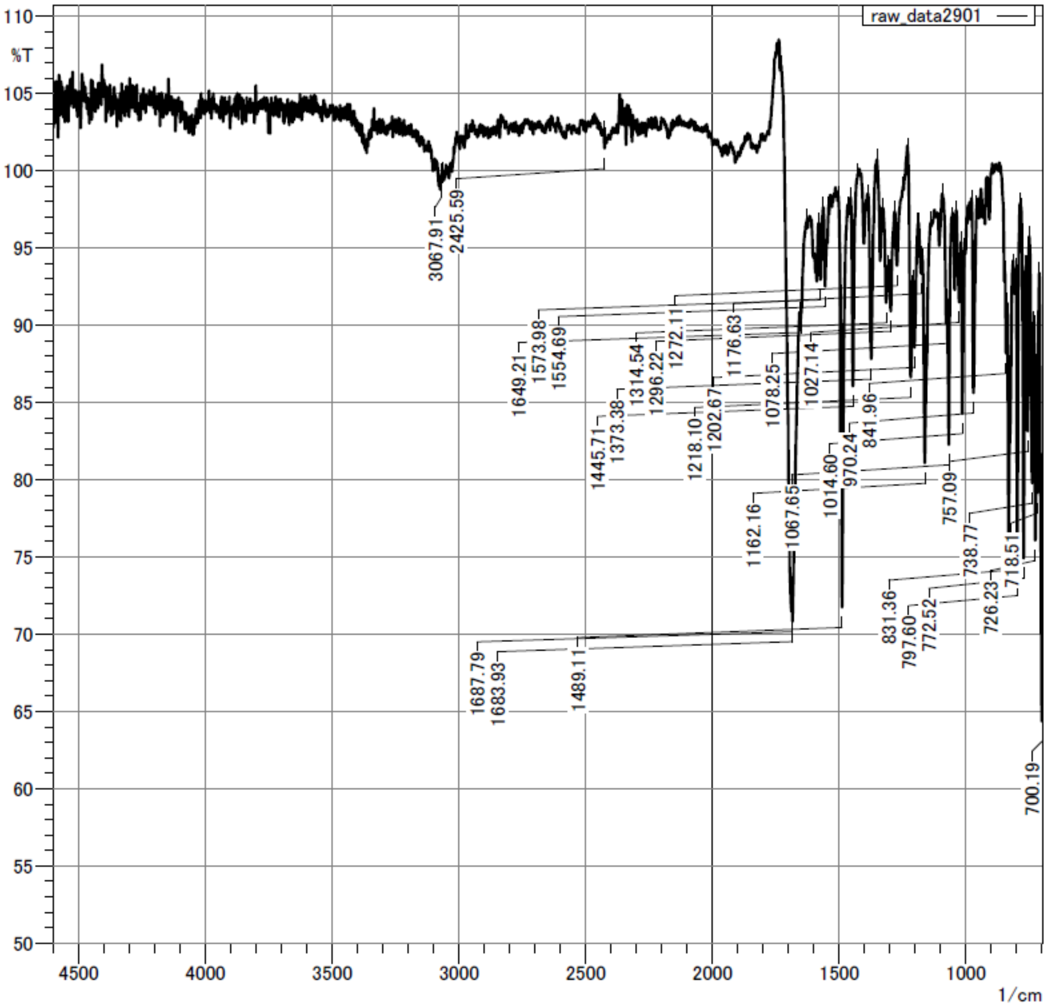

HRMS spectrum of **2n**

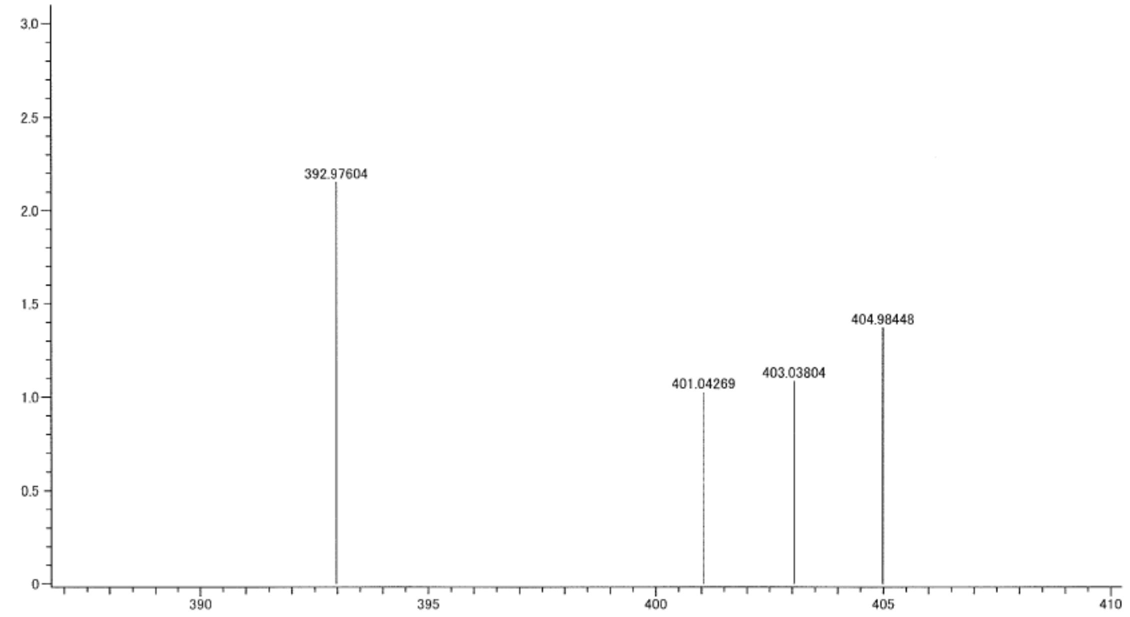

^{1}H-NMR spectrum of **2o** ($CDCl_3$, 600 MHz)

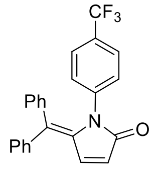

2o

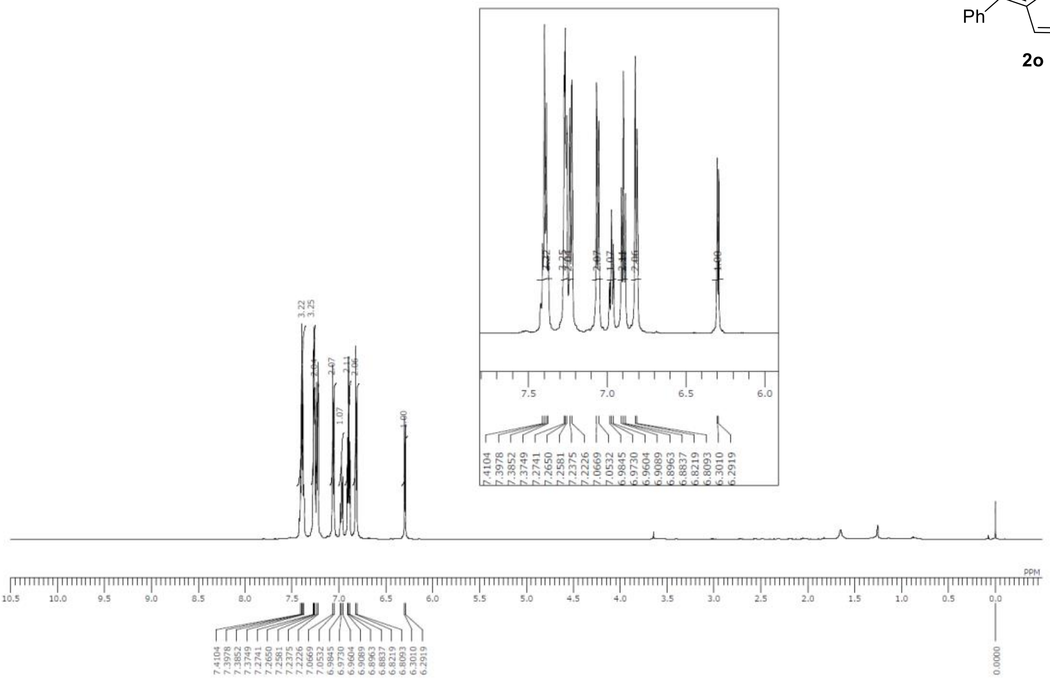

^{13}C-NMR spectrum of **2o** ($CDCl_3$, 150 MHz)

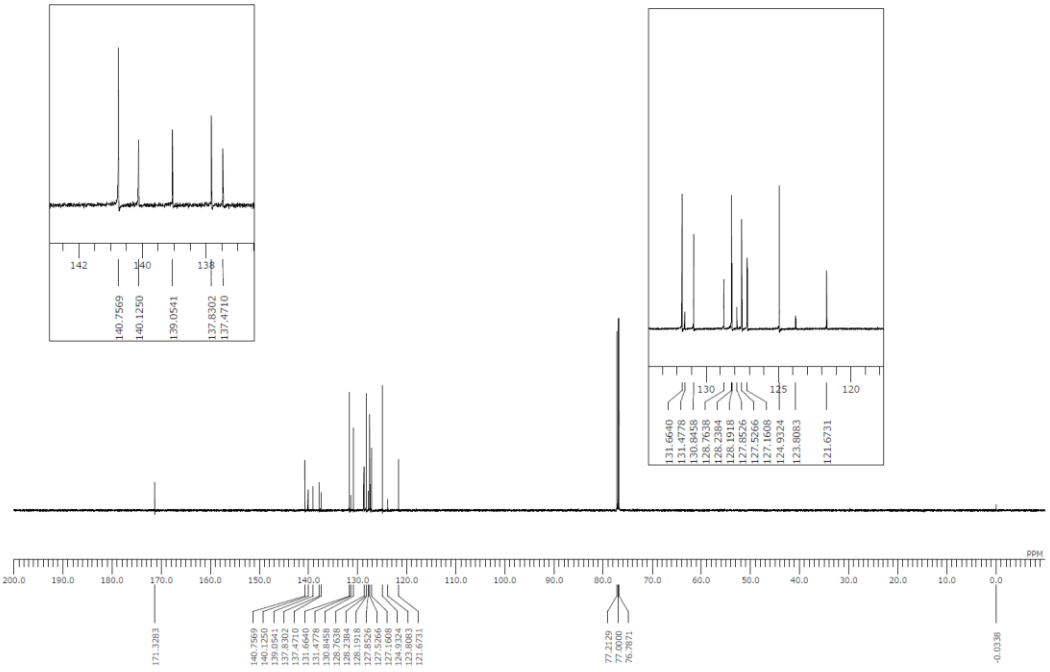

^{19}F-NMR spectrum of **2o** ($CDCl_3$, 565 MHz)

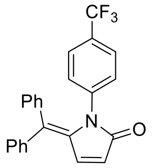

2o

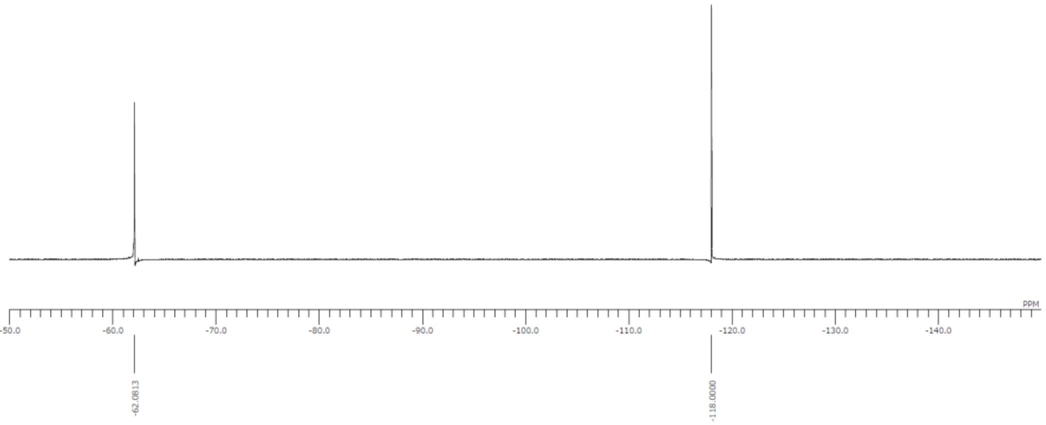

IR spectrum of **2o**

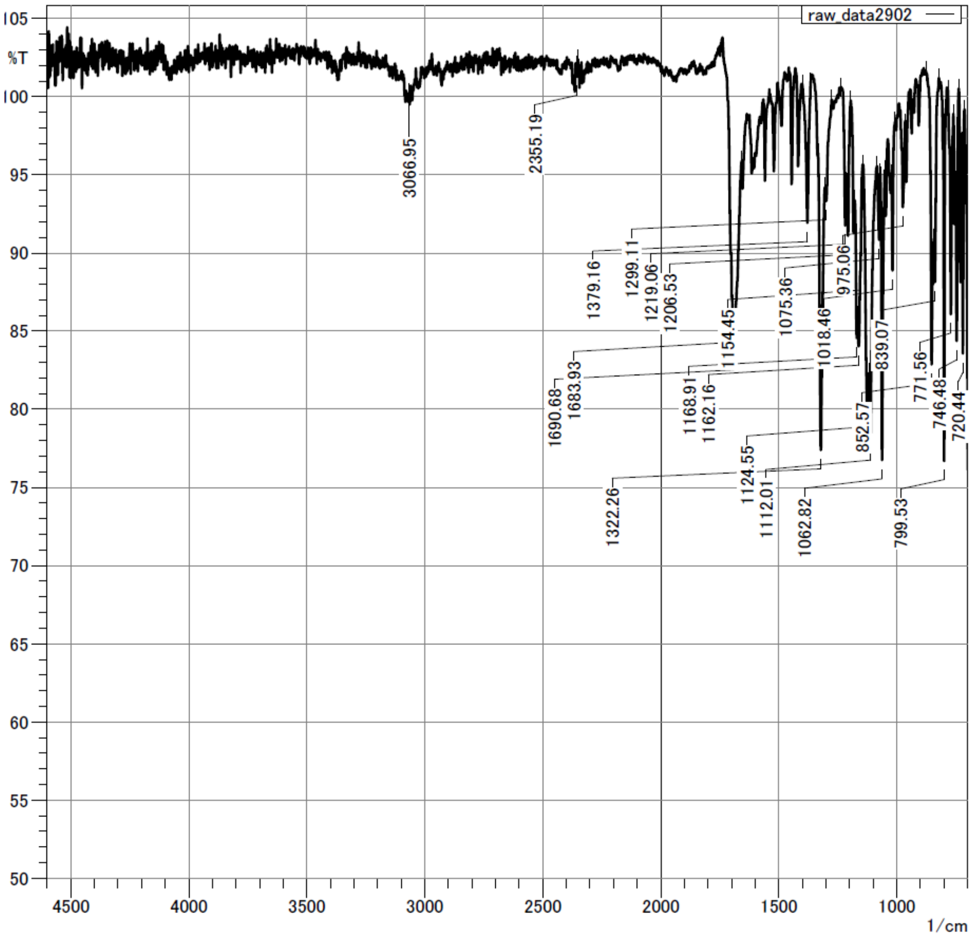

HRMS spectrum of **2o**

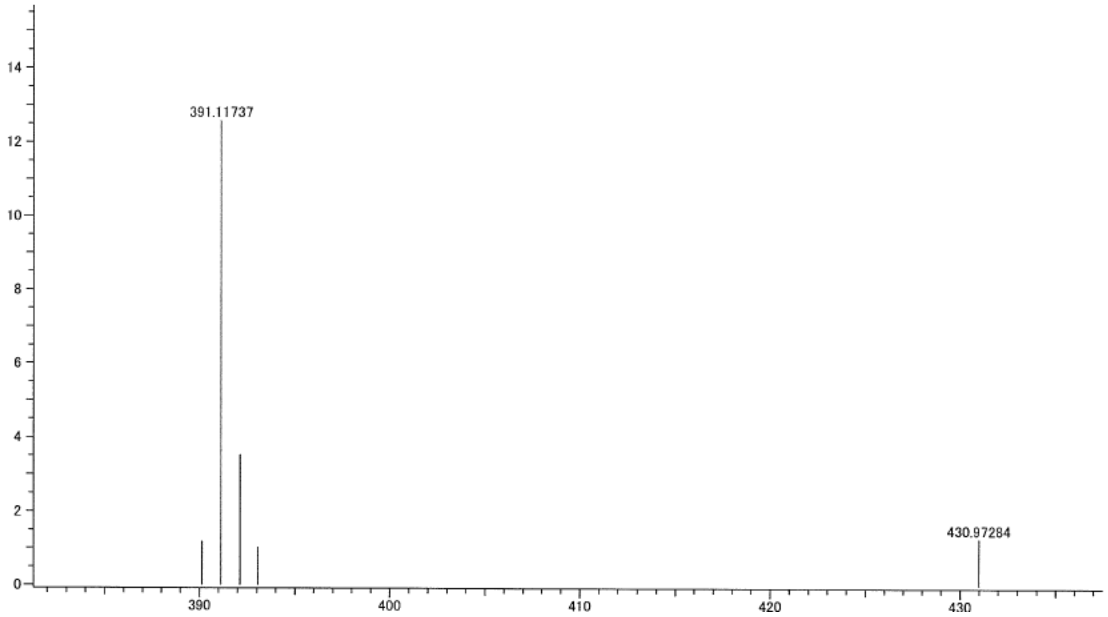

^{1}H-NMR spectrum of **2p** ($CDCl_3$, 600 MHz)

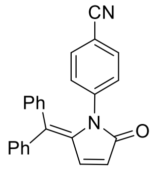

2p

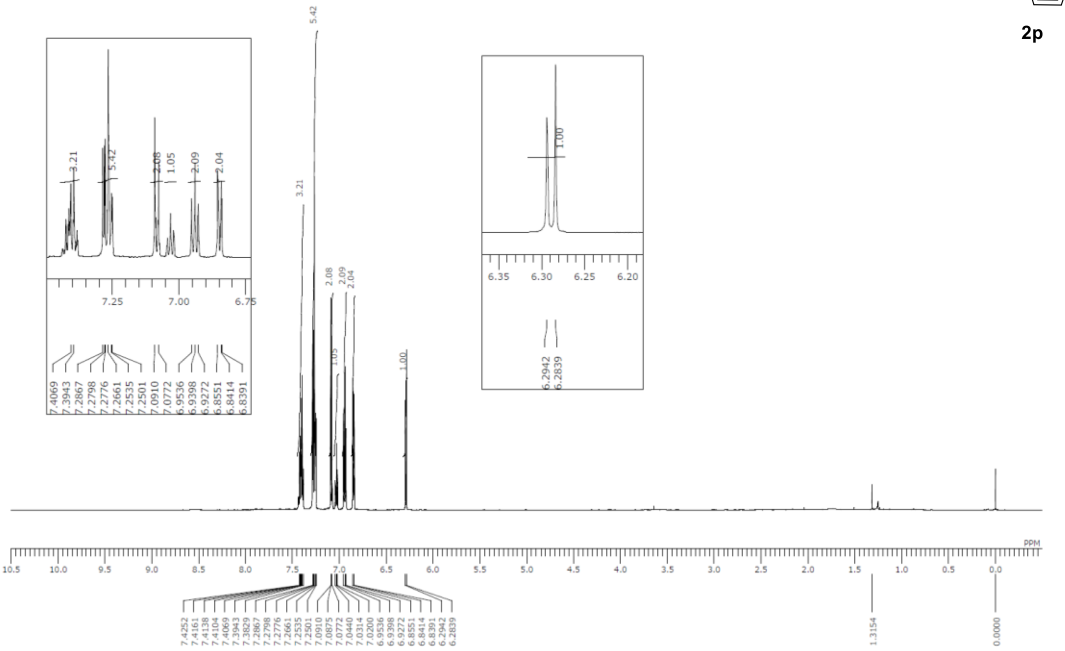

^{13}C-NMR spectrum of **2p** ($CDCl_3$, 150 MHz)

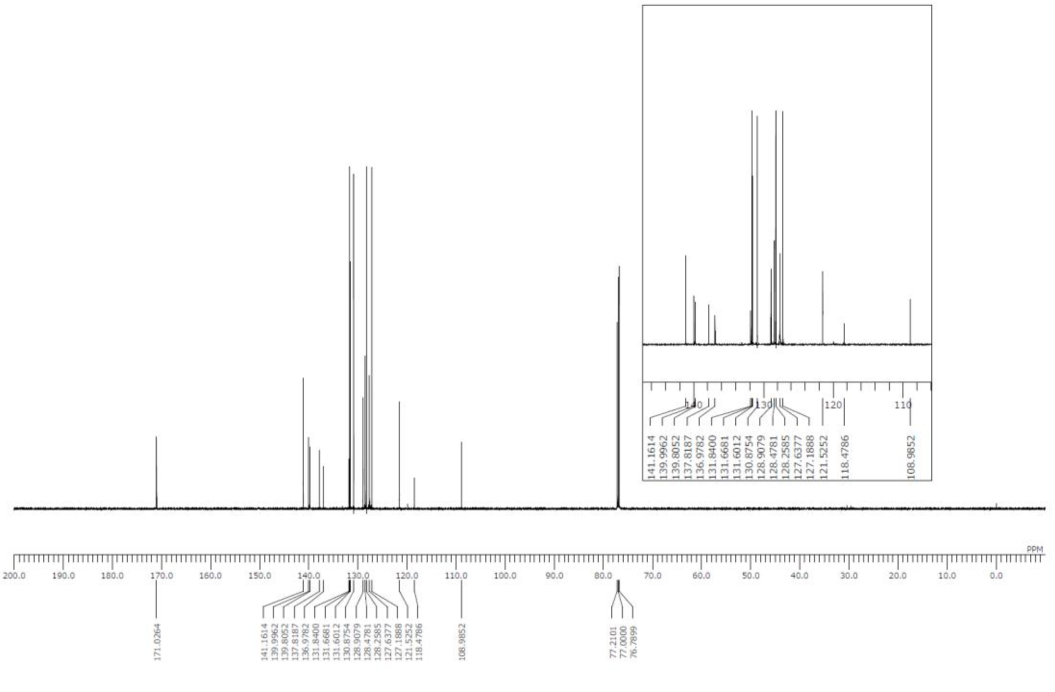

IR spectrum of **2p**

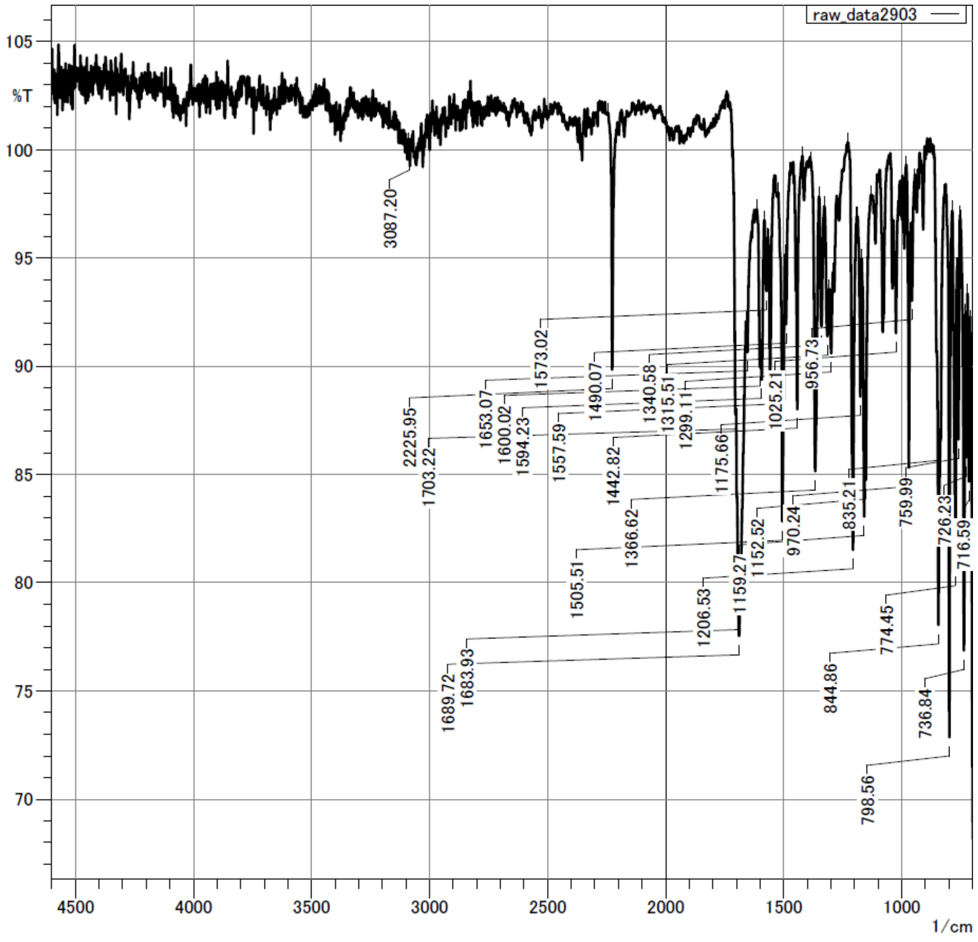

HRMS spectrum of **2p**

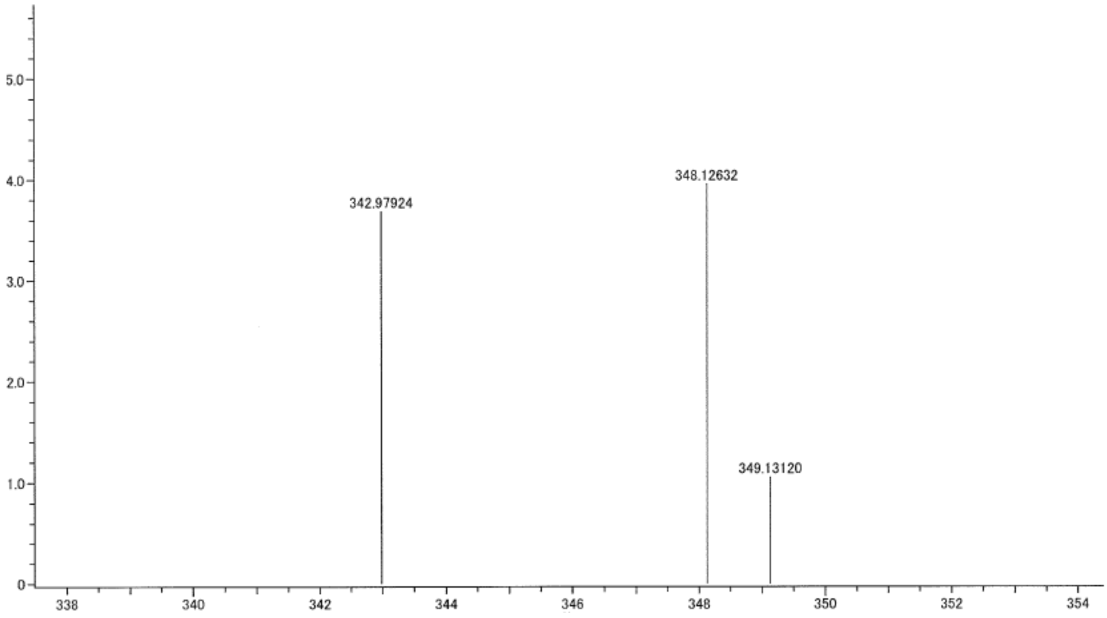

^{1}H-NMR spectrum of **2q** ($CDCl_3$, 600 MHz)

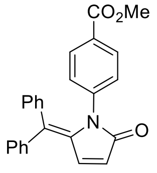

2q

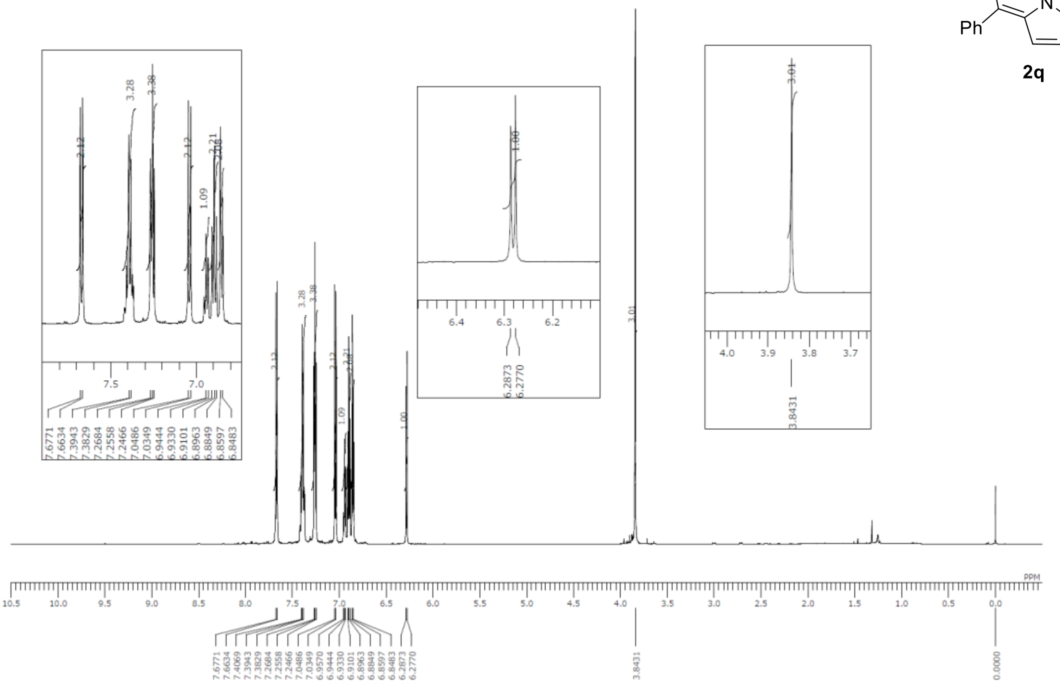

^{13}C-NMR spectrum of **2q** ($CDCl_3$, 150 MHz)

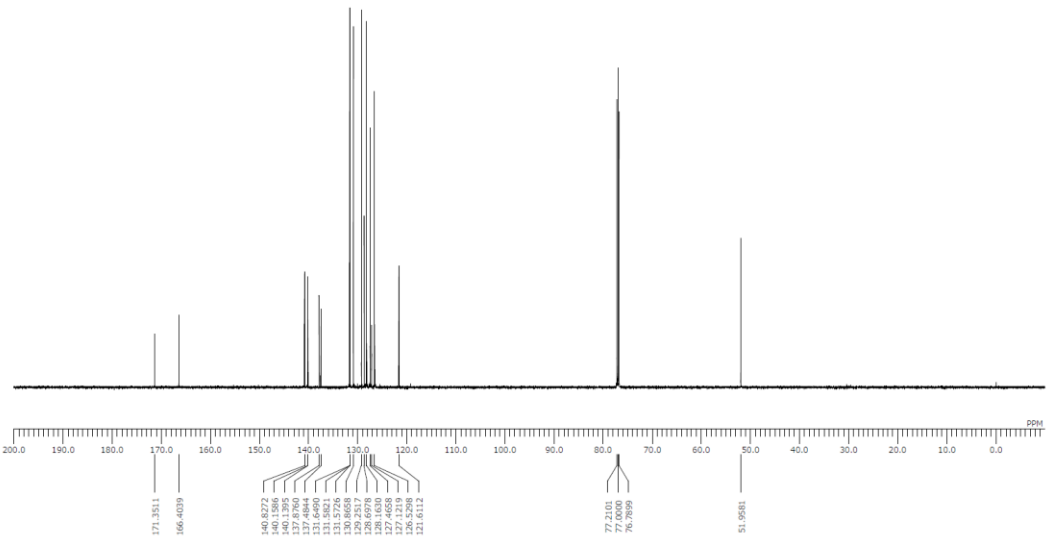

IR spectrum of **2q**

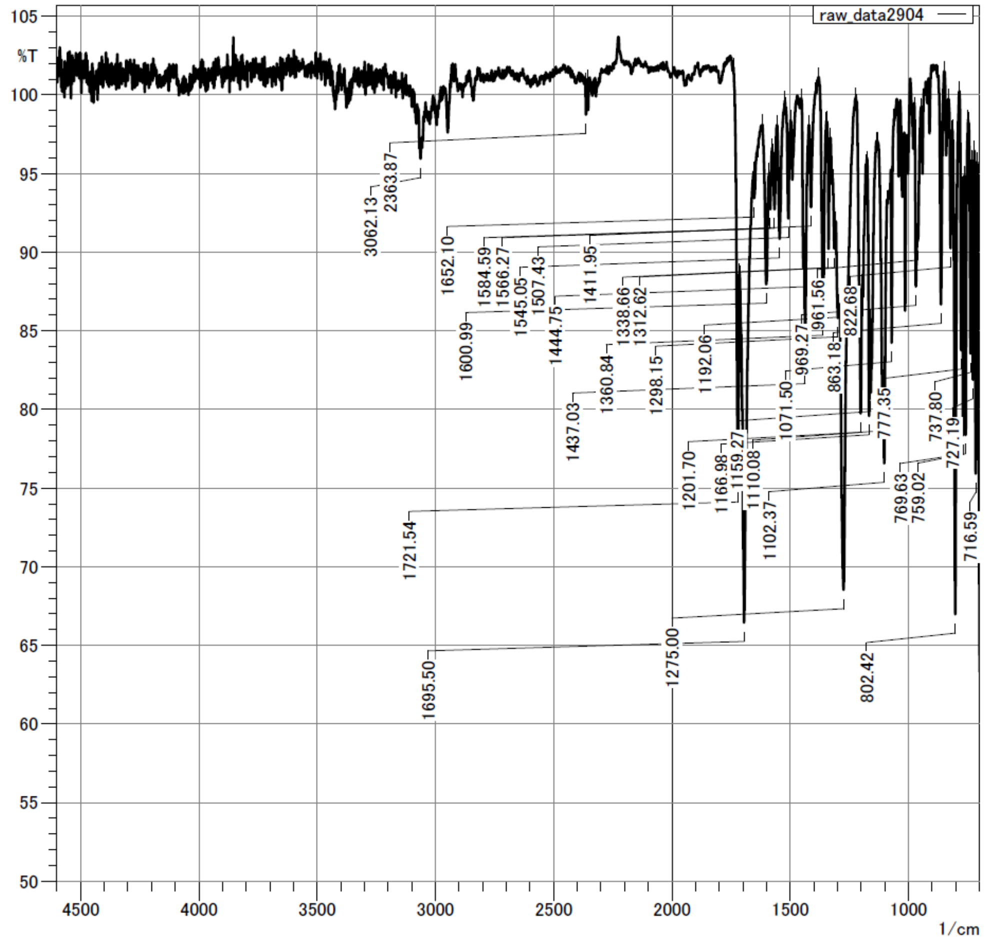

HRMS spectrum of **2q**

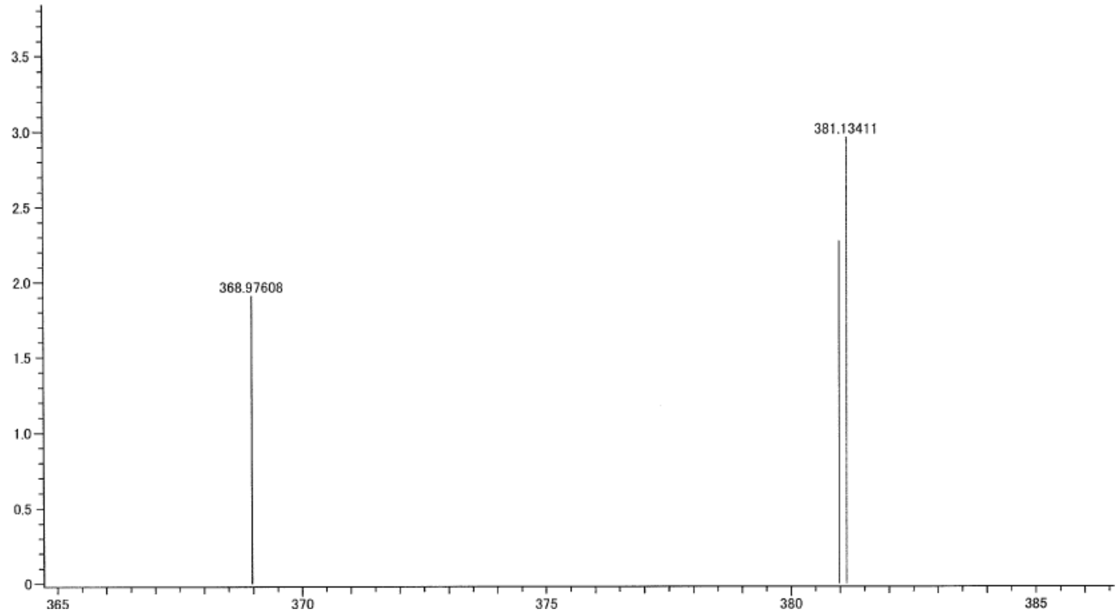

^{1}H-NMR spectrum of **2r** ($CDCl_3$, 600 MHz)

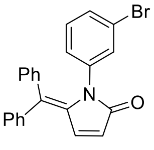

2r

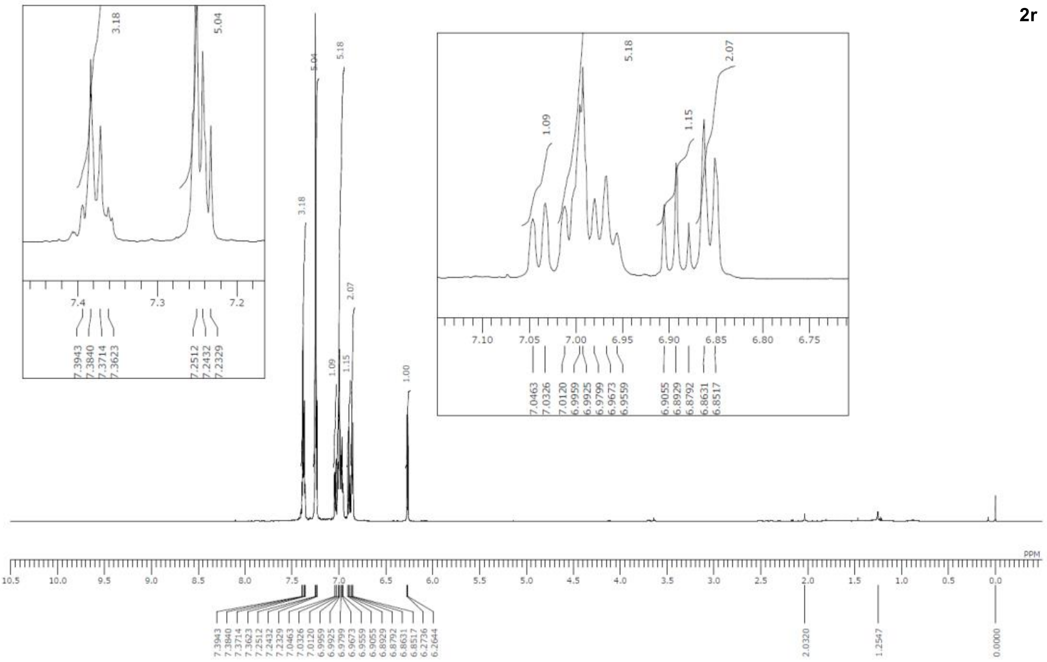

^{13}C-NMR spectrum of **2r** ($CDCl_3$, 150 MHz)

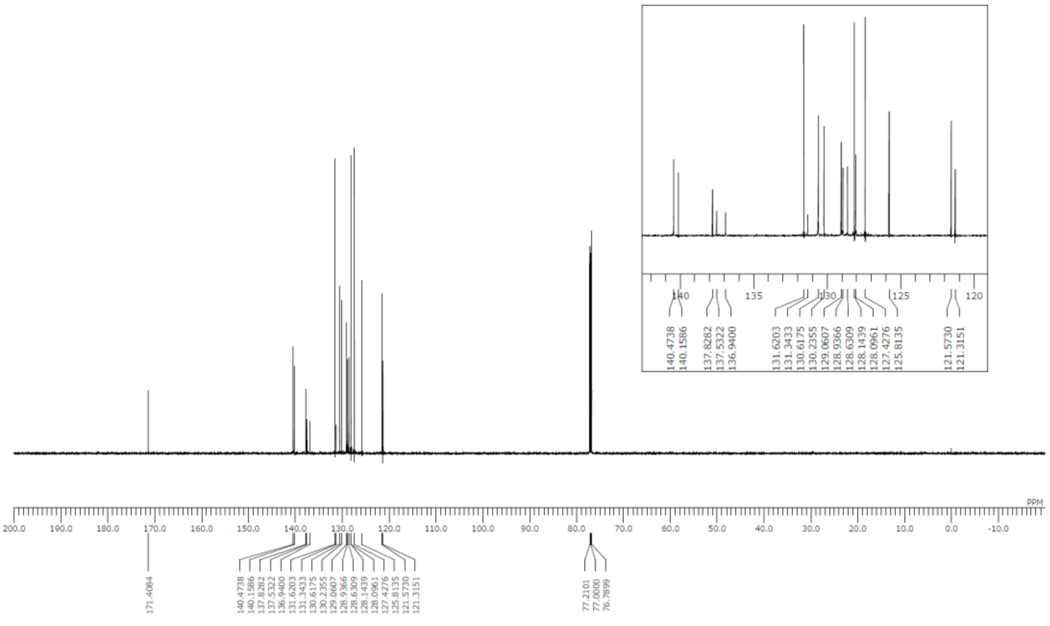

IR spectrum of **2r**

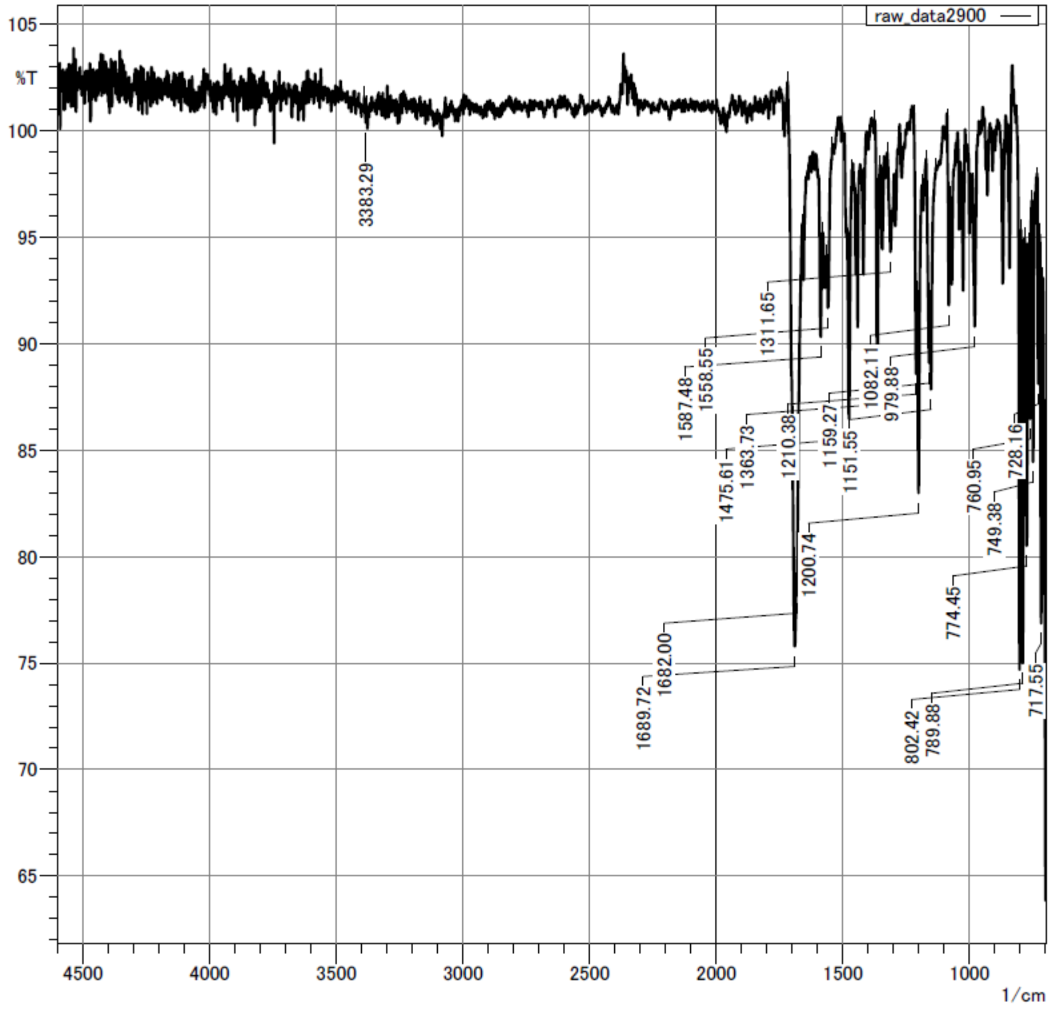

HRMS spectrum of **2r**

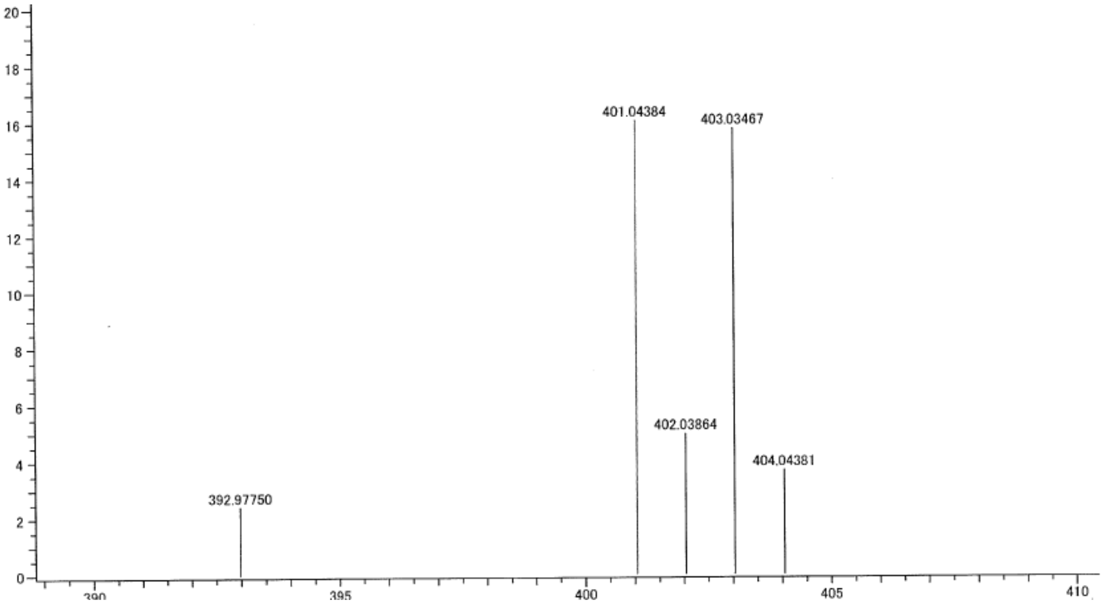

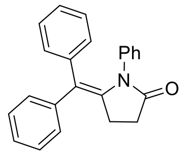
3a

^{1}H-NMR spectrum of **3a** ($CDCl_3$, 600 MHz)

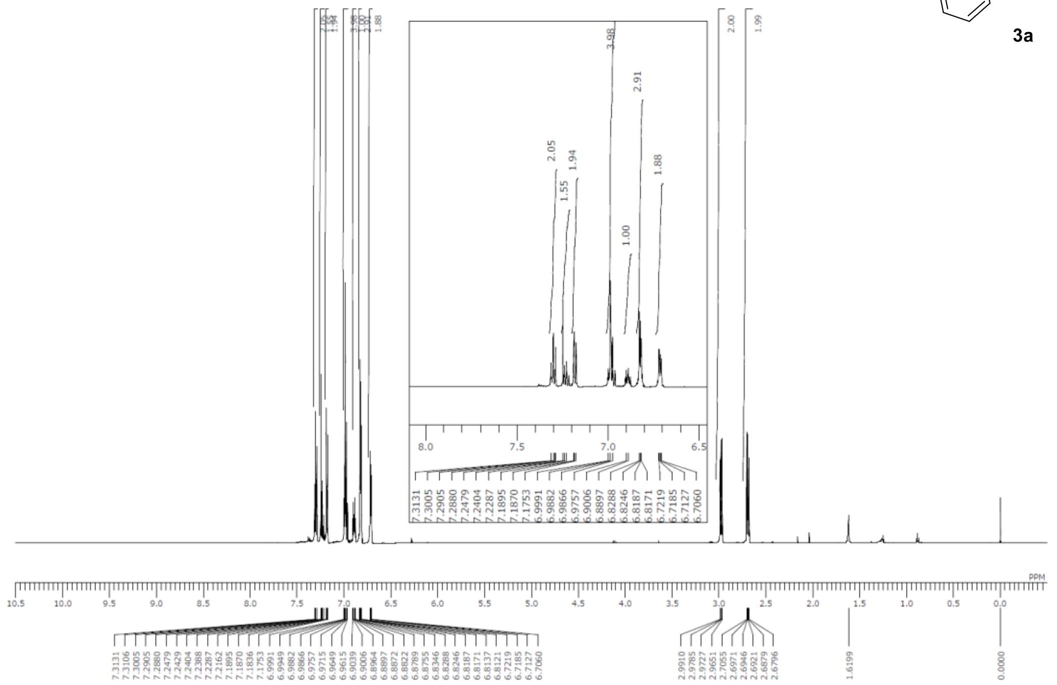

^{13}C-NMR spectrum of **3a** ($CDCl_3$, 150 MHz)

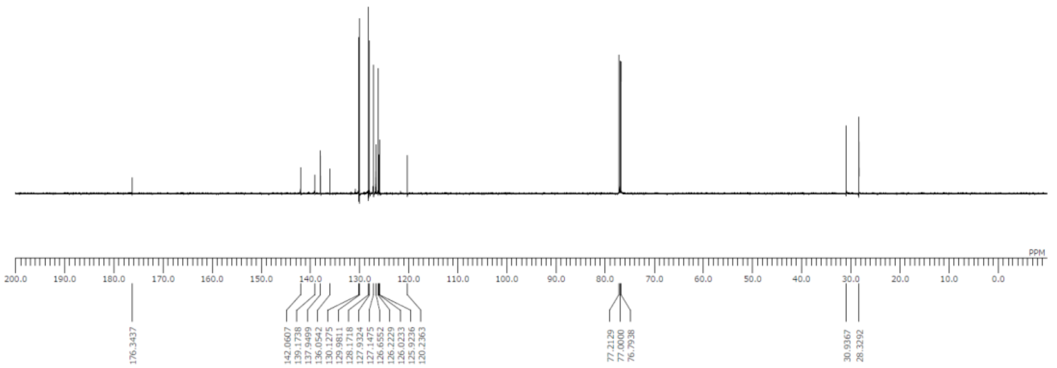

IR spectrum of **3a**

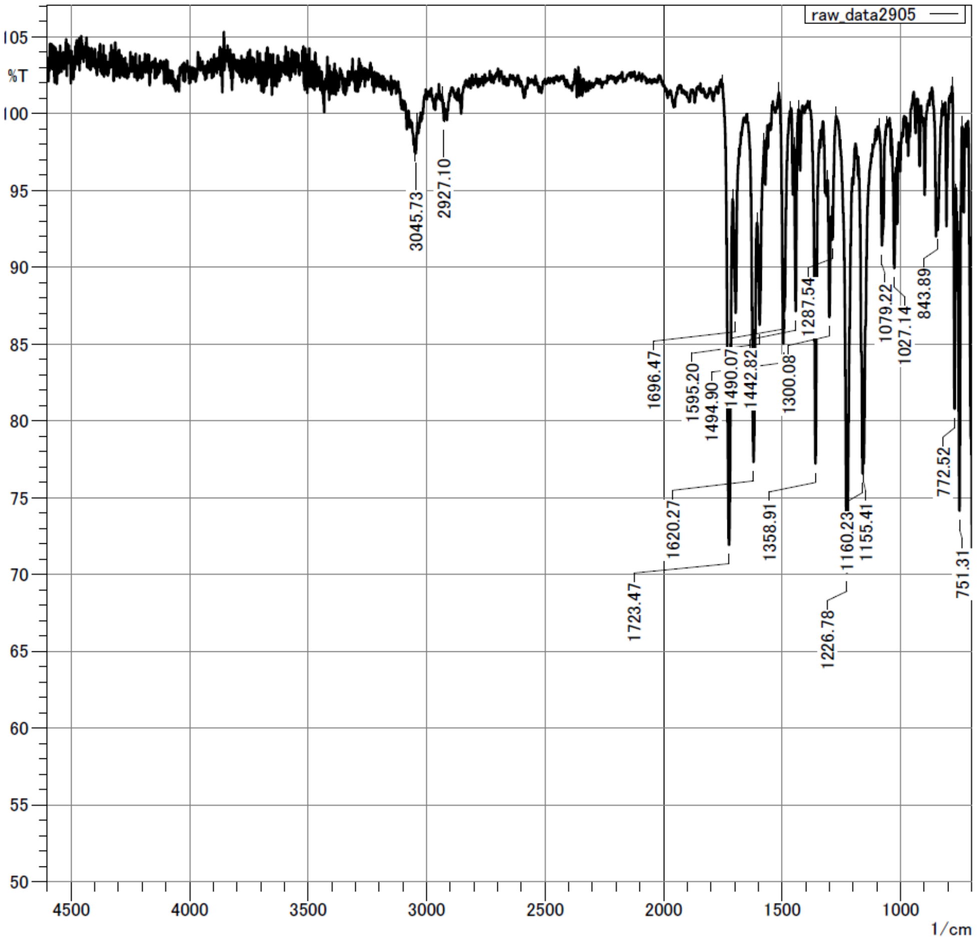

HRMS spectrum of **3a**

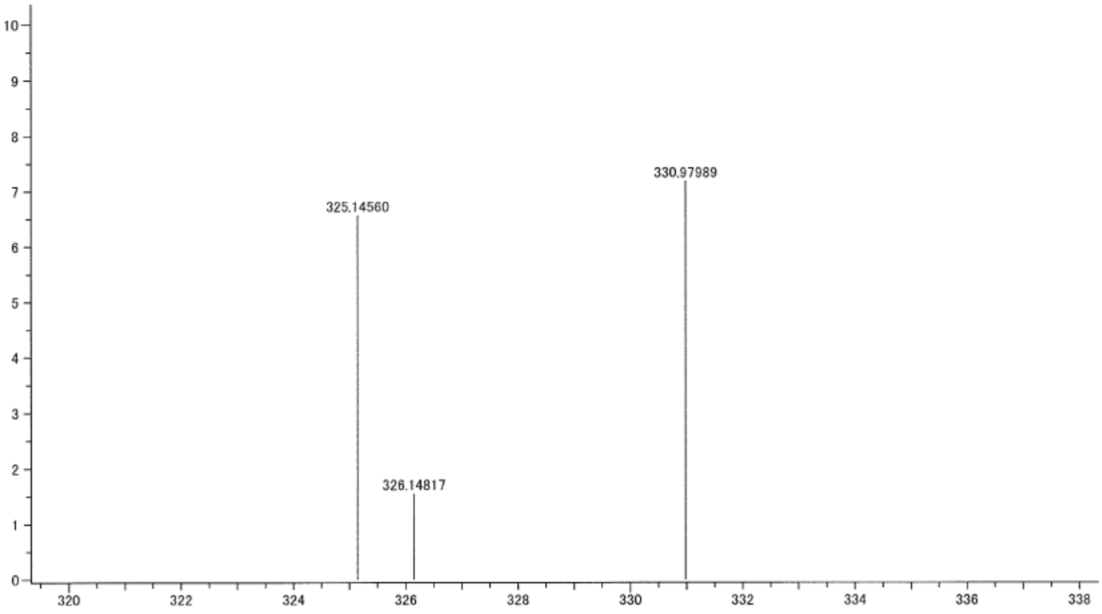

^{1}H-NMR spectrum of **3b** ($CDCl_3$, 600 MHz)

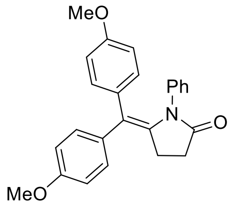

3b

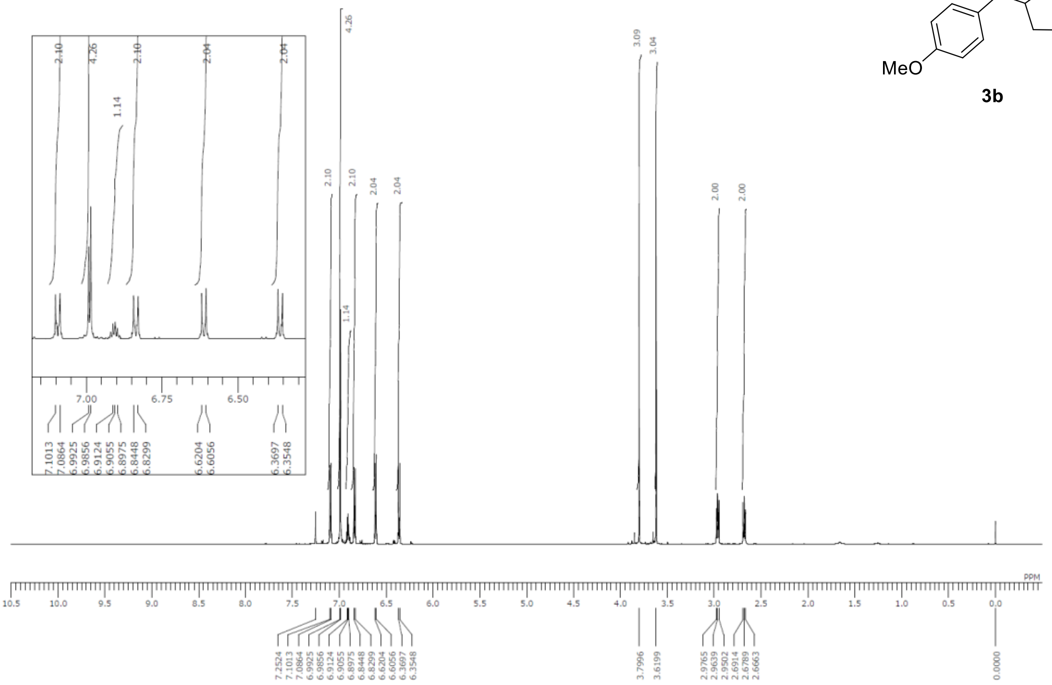

^{13}C-NMR spectrum of **3b** ($CDCl_3$, 150 MHz)

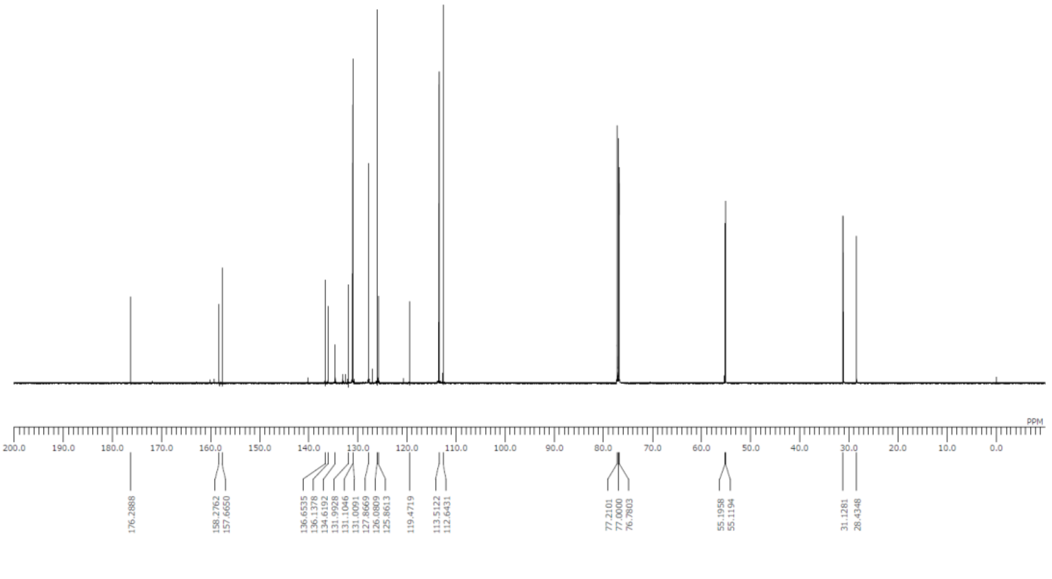

IR spectrum of **3b**

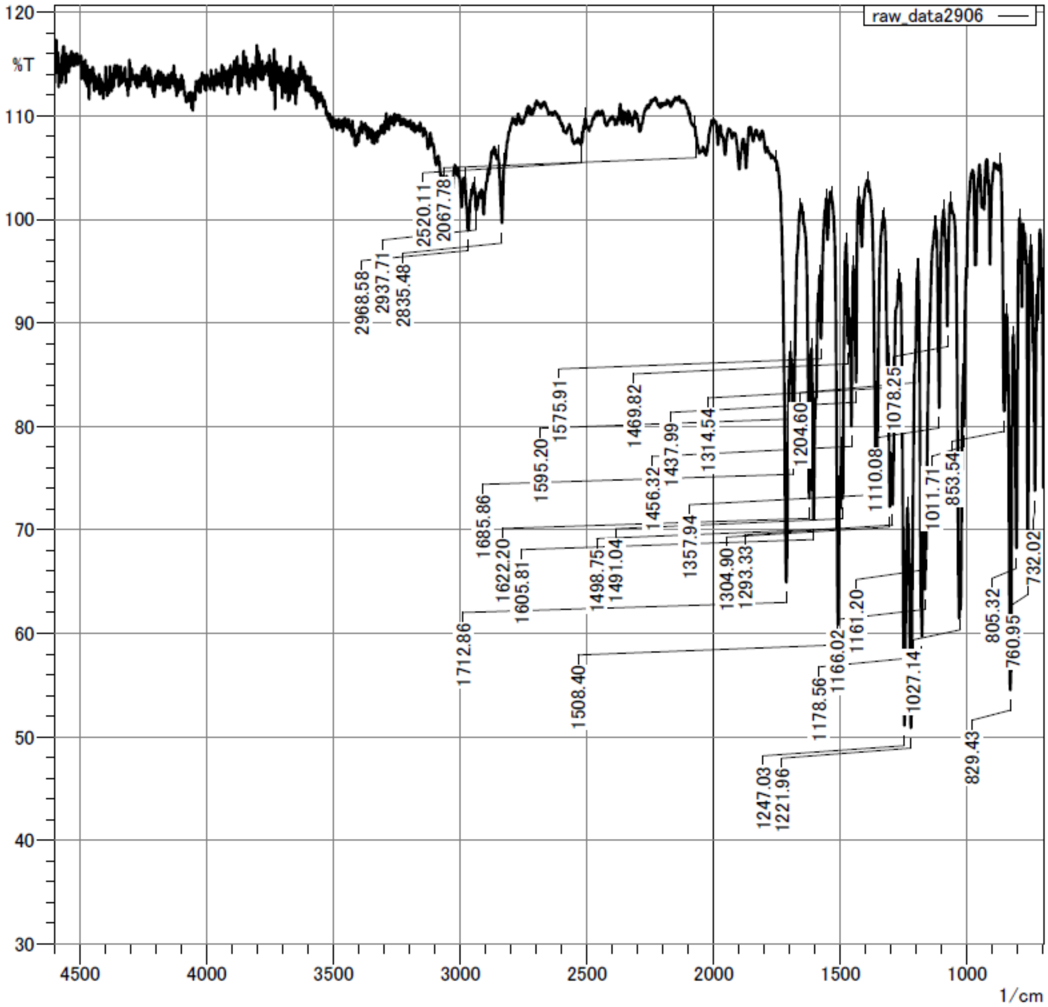

HRMS spectrum of **3b**

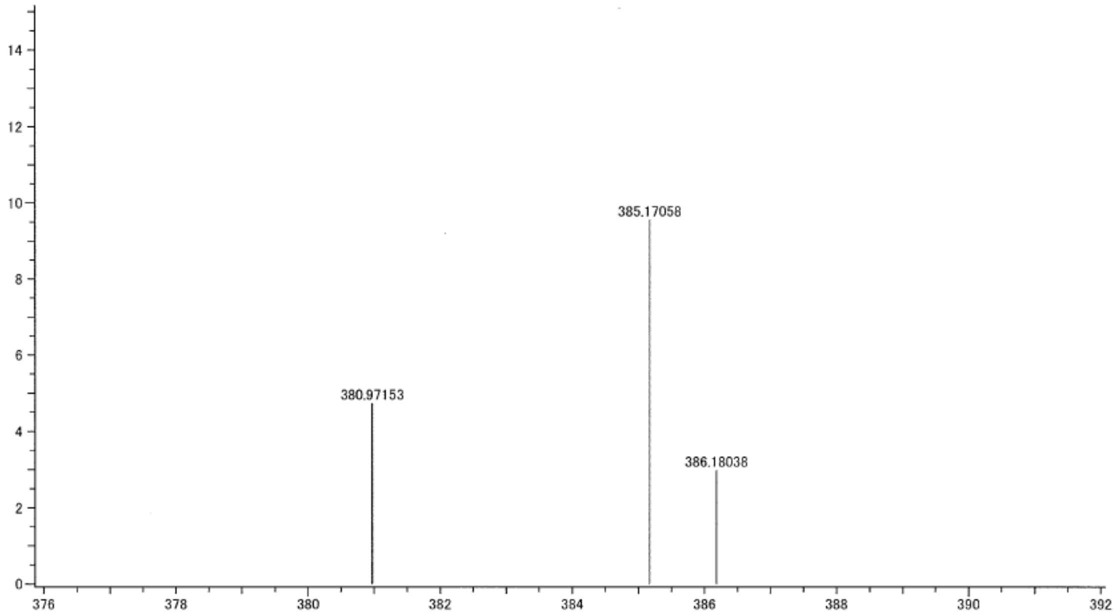

^{1}H-NMR spectrum of **3c** (**3c** : **2c** = 93 : 7, $CDCl_3$, 400 MHz)

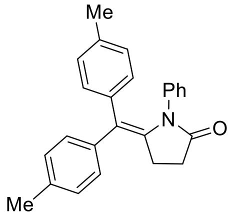
3c

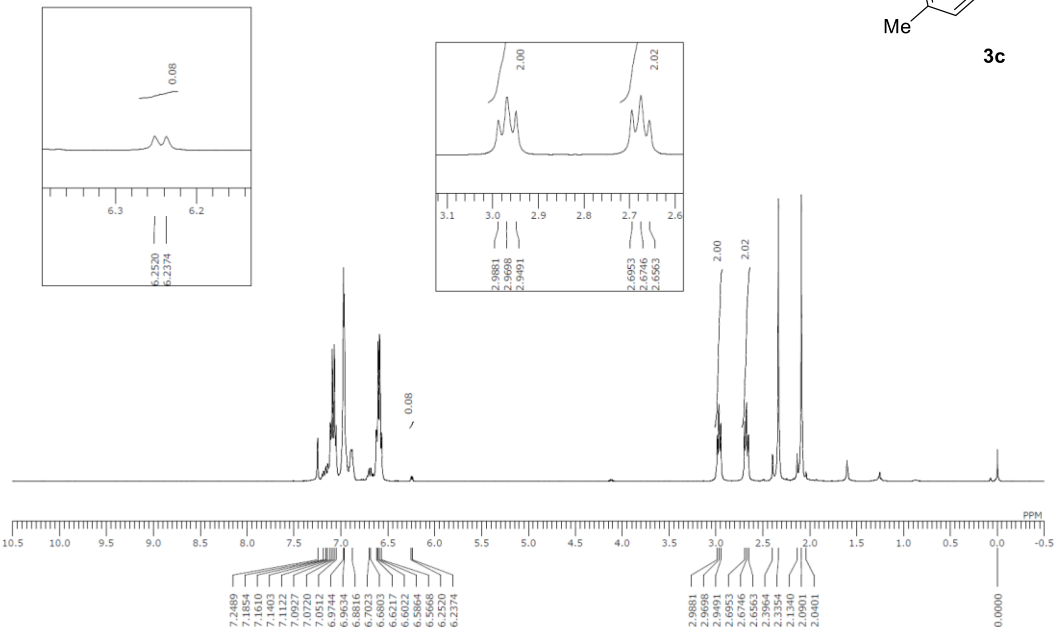

^{1}H-NMR spectrum of **3c** ($CDCl_3$, 600 MHz)

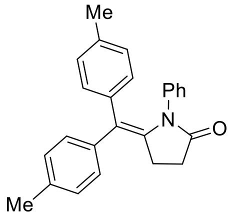

3c

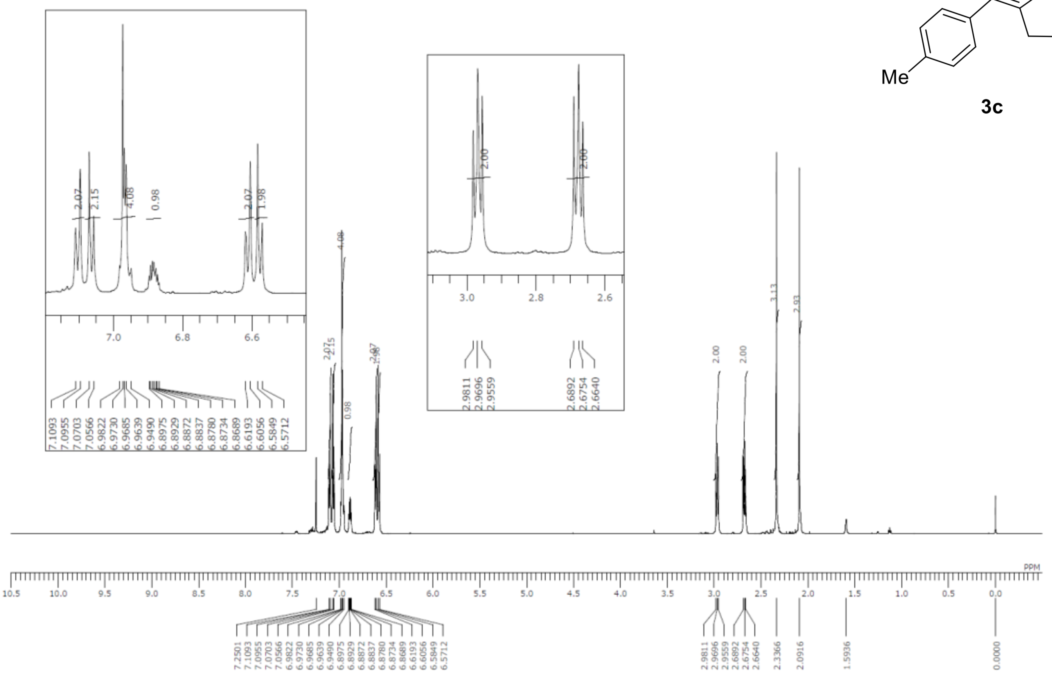

^{13}C-NMR spectrum of **3c** ($CDCl_3$, 150 MHz)

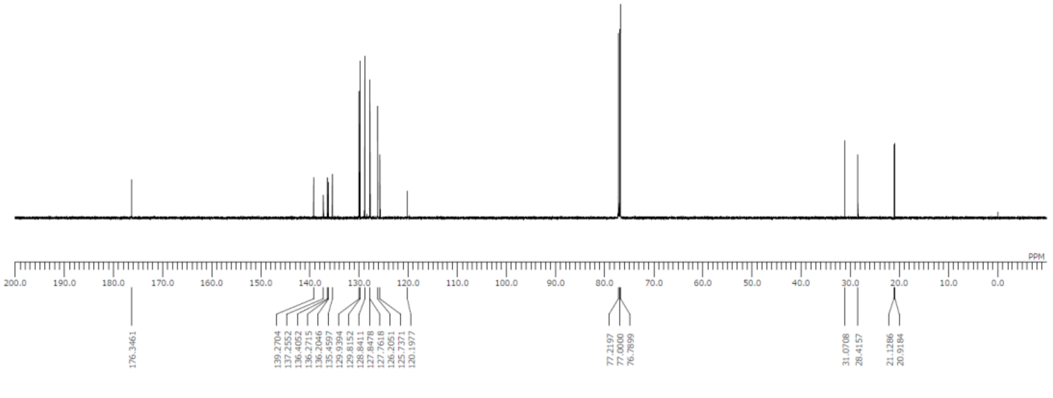

IR spectrum of **3c**

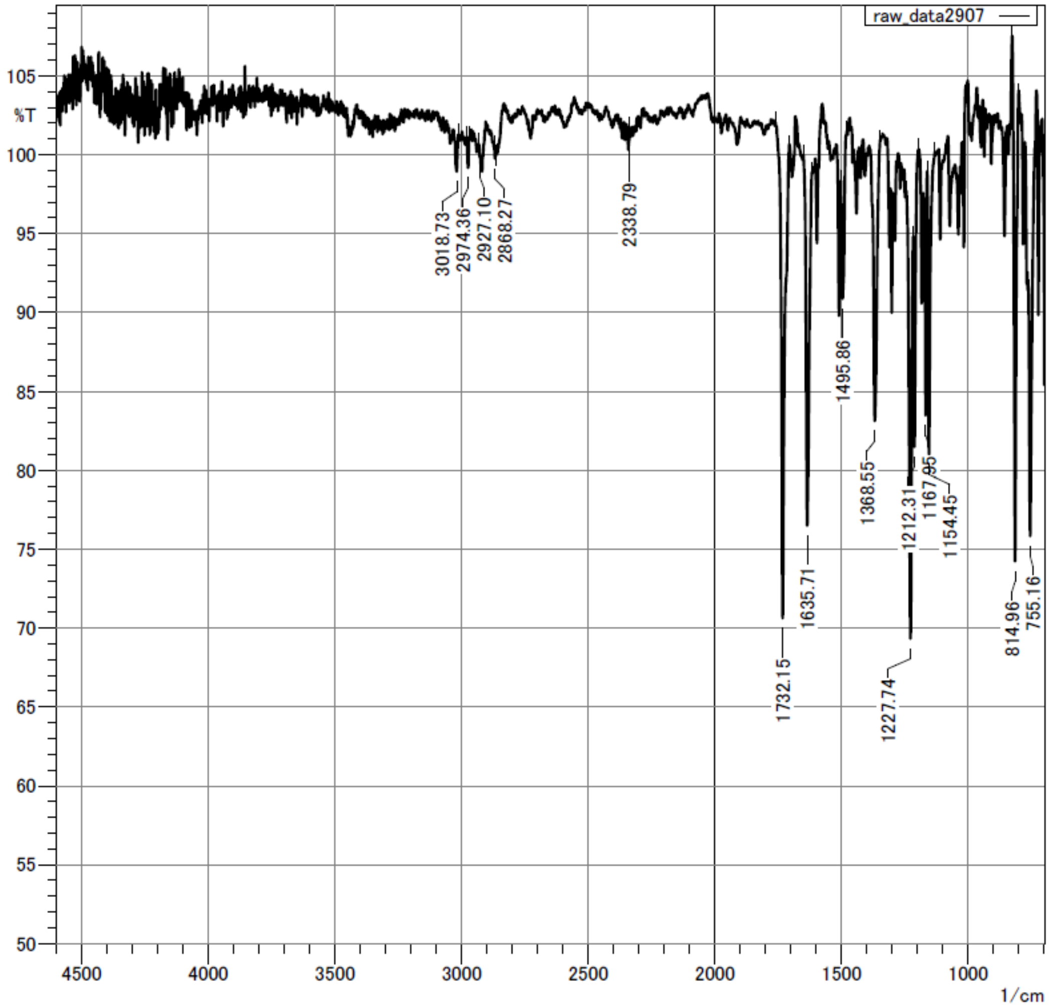

HRMS spectrum of **3c**

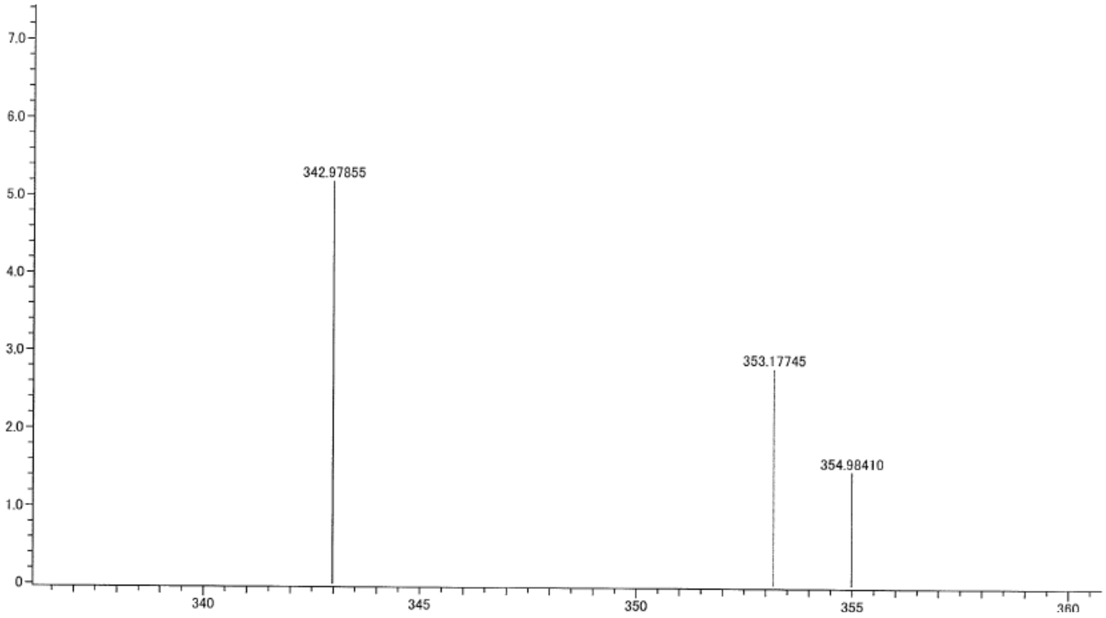

^{1}H-NMR spectrum of **3d** (**3d** : **2d** = 95 : 5, $CDCl_3$, 400 MHz)

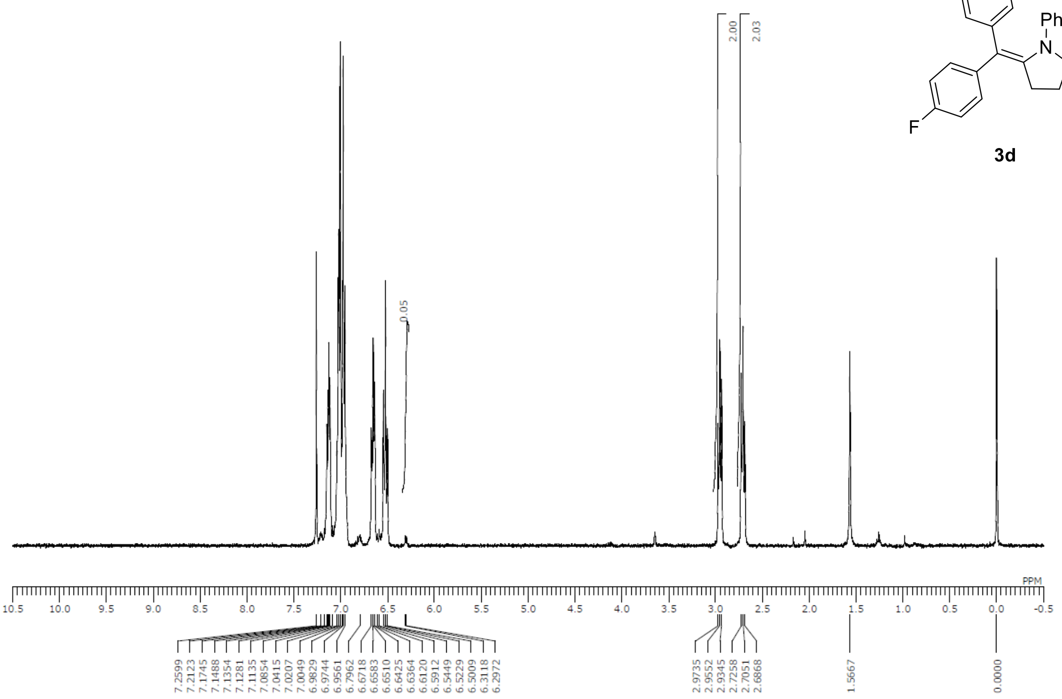

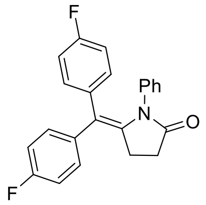

3d

^{1}H-NMR spectrum of **3d** ($CDCl_3$, 600 MHz)

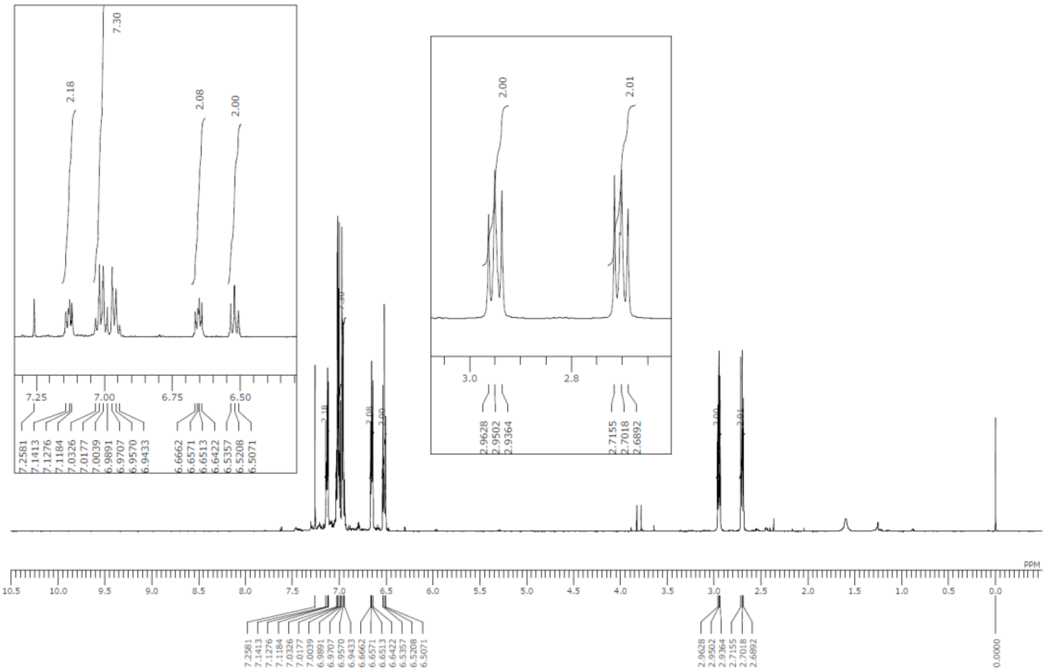

^{13}C-NMR spectrum of **3d** ($CDCl_3$, 150 MHz)

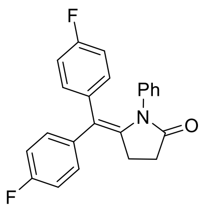

3d

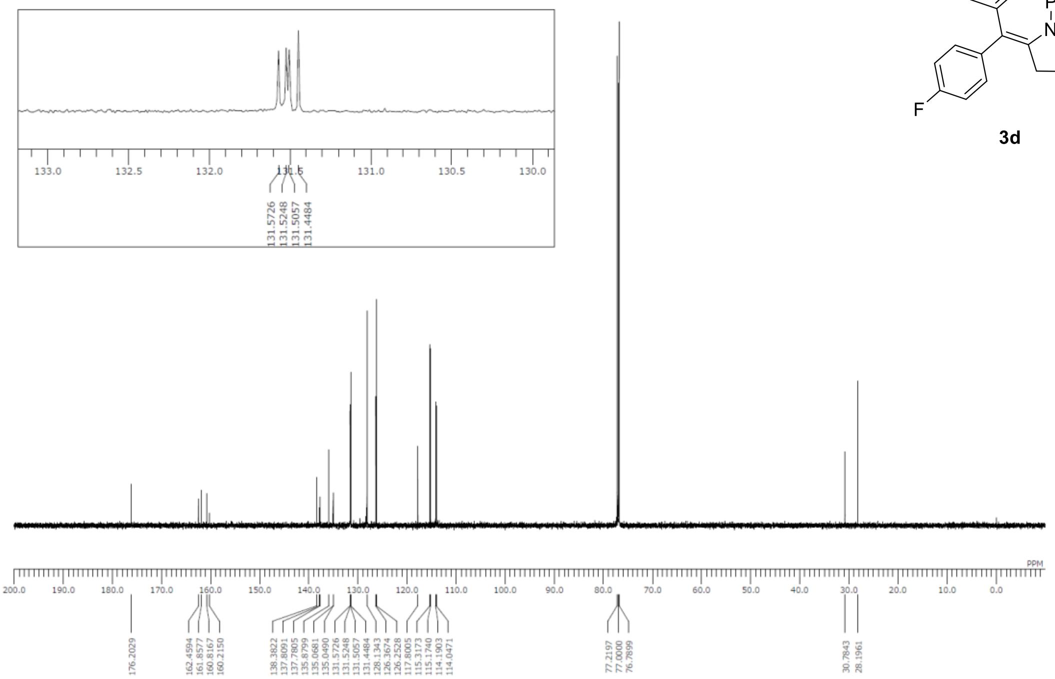

^{19}F-NMR spectrum of **3d** ($CDCl_3$, 565 MHz)

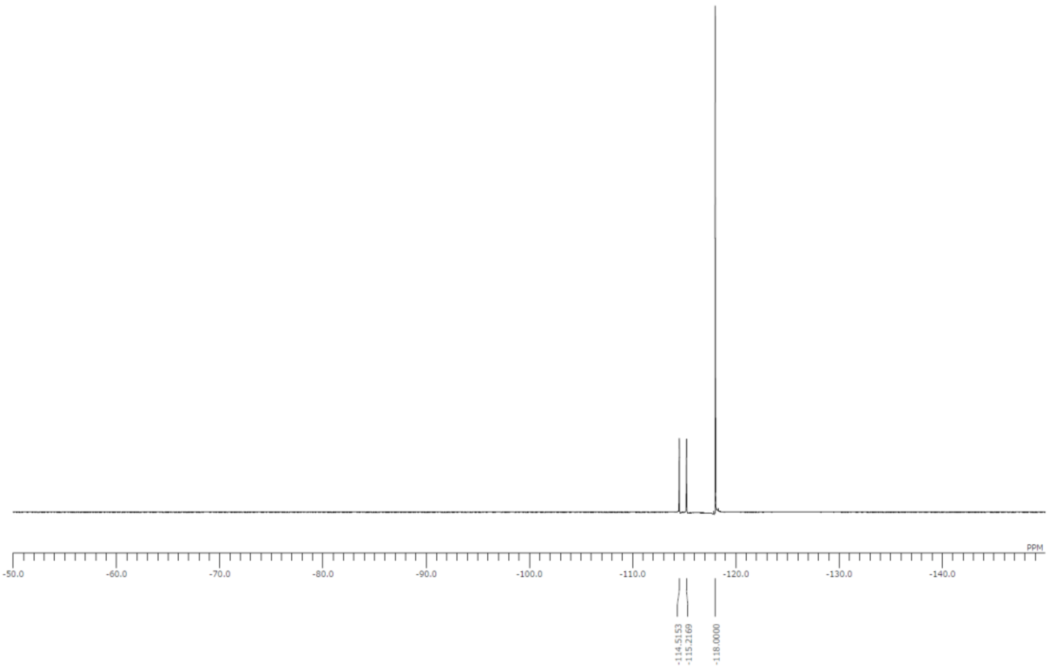

IR spectrum of **3d**

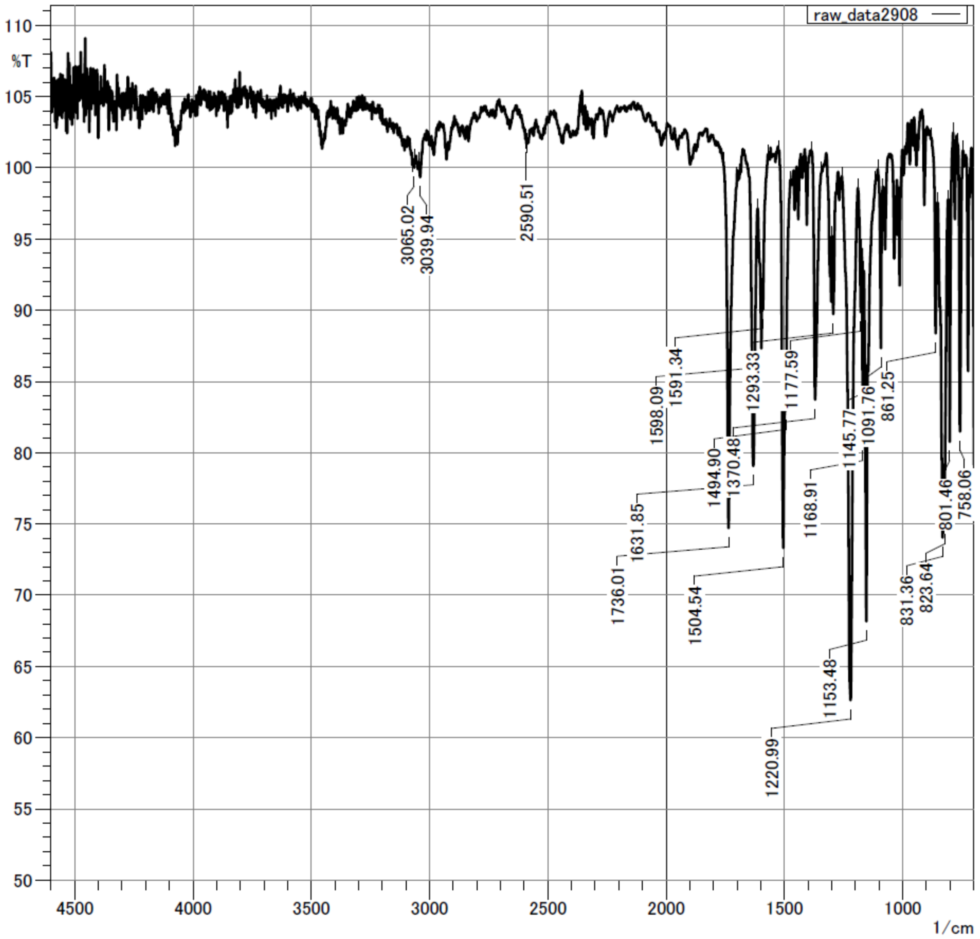

HRMS spectrum of **3d**

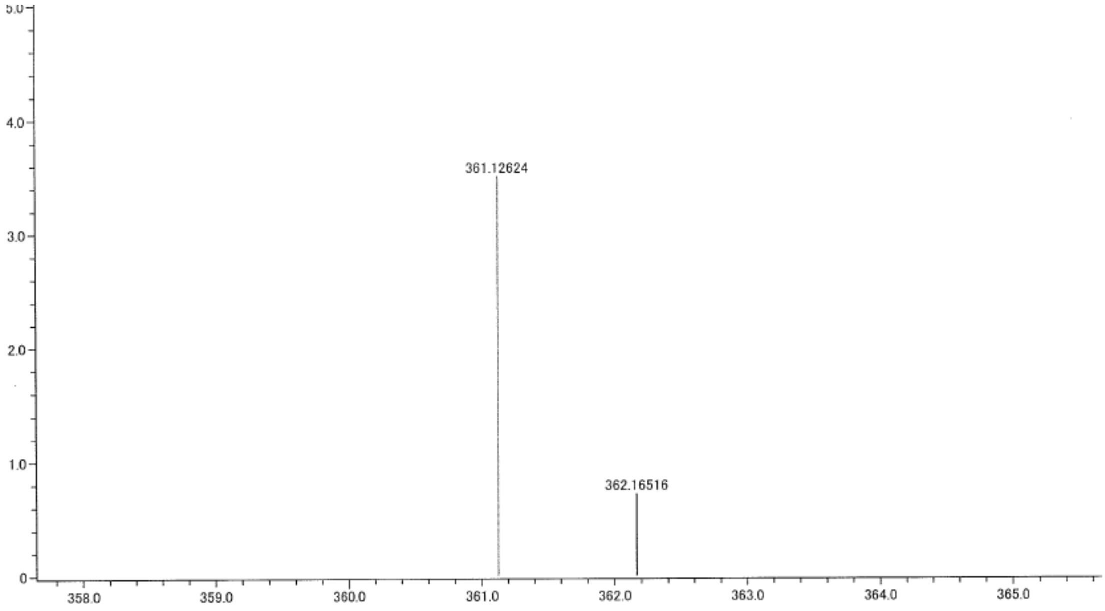

^{1}H-NMR spectrum of **3e** (**3e** : **2e** = 94 : 6, $CDCl_3$, 400 MHz)

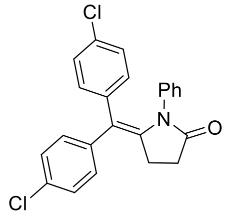

3e

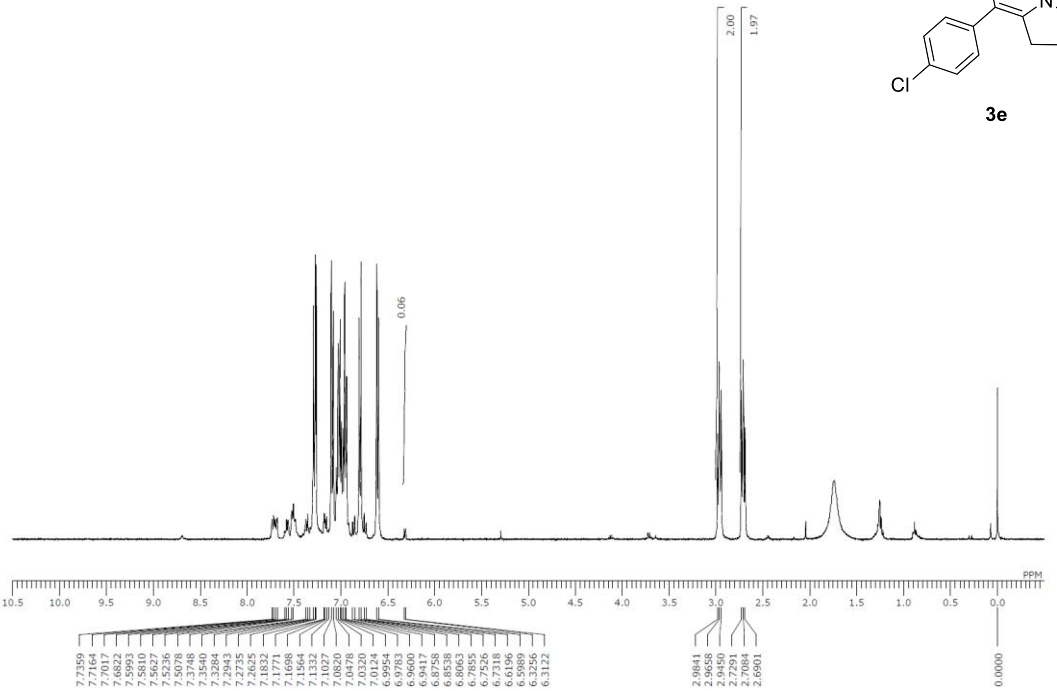

^{1}H-NMR spectrum of **3e** ($CDCl_3$, 600 MHz)

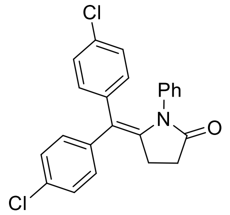

3e

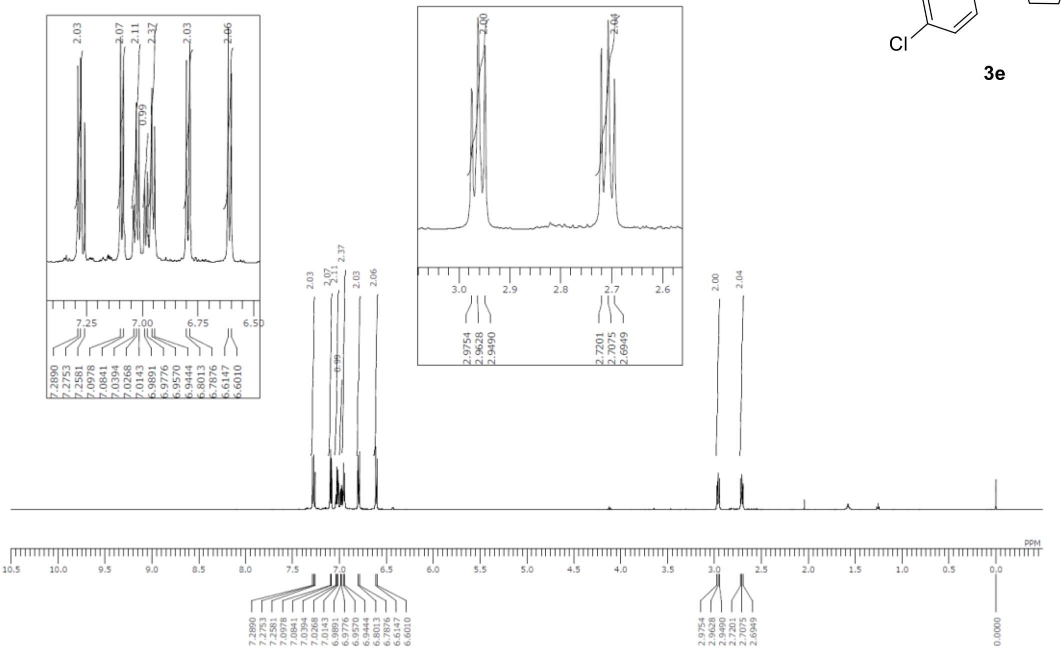

^{13}C-NMR spectrum of **3e** ($CDCl_3$, 150 MHz)

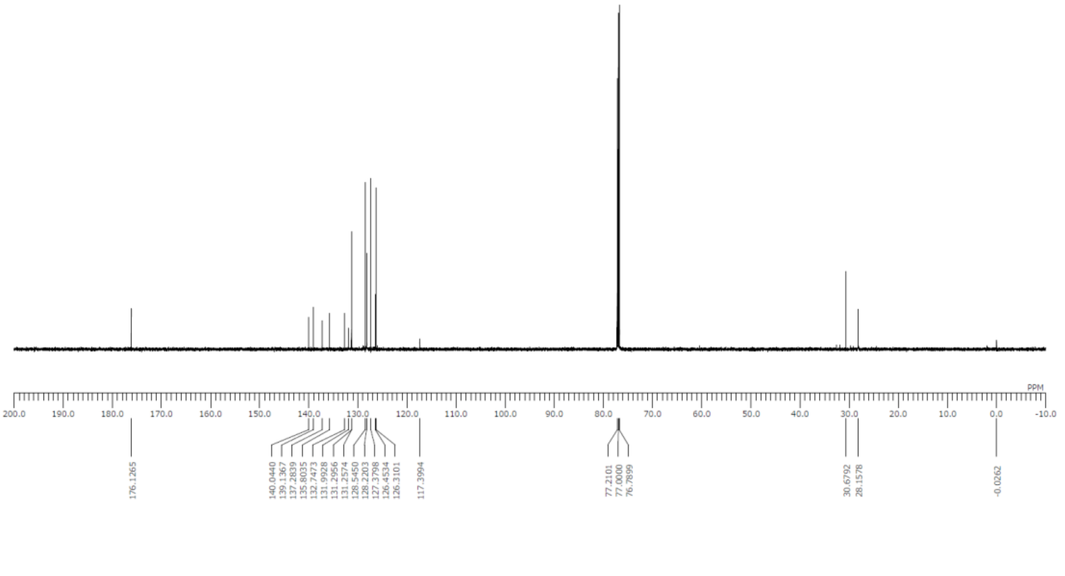

IR spectrum of **3e**

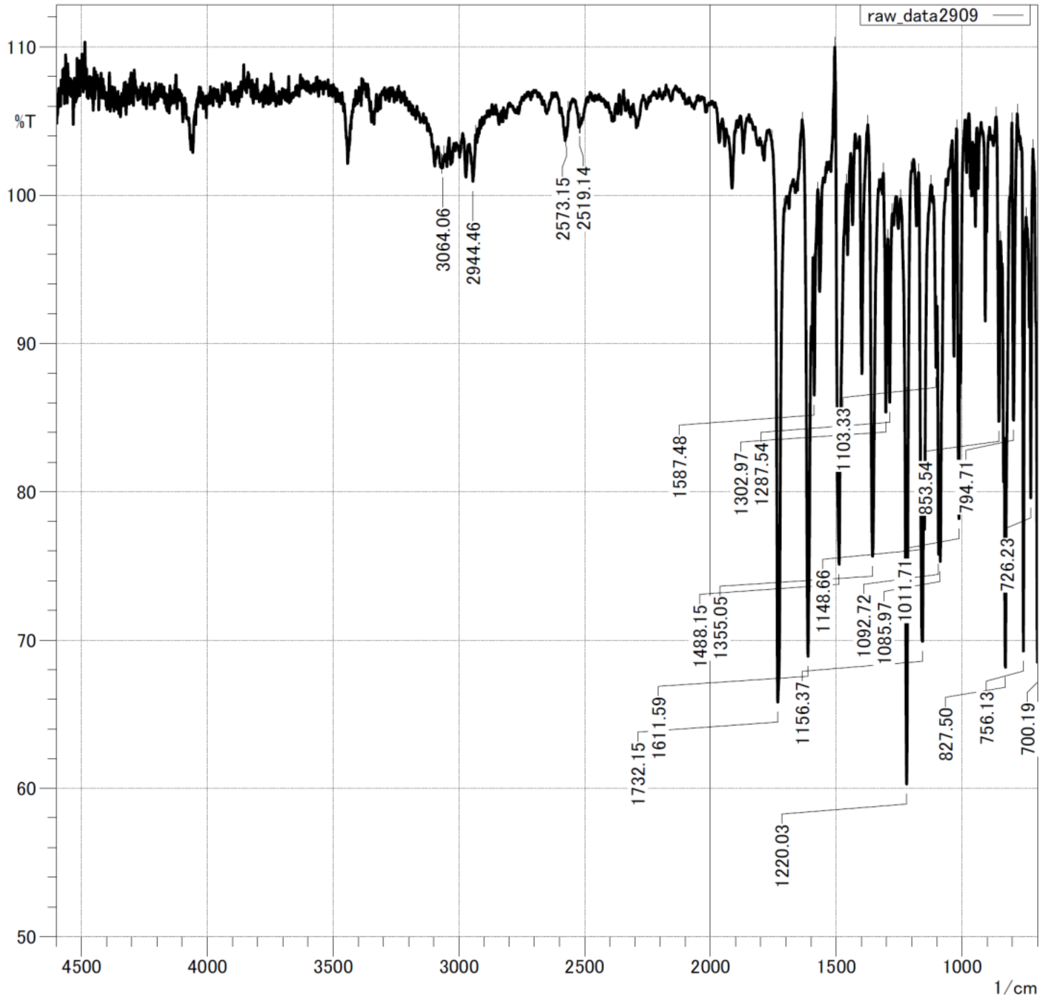

HRMS spectrum of **3e**

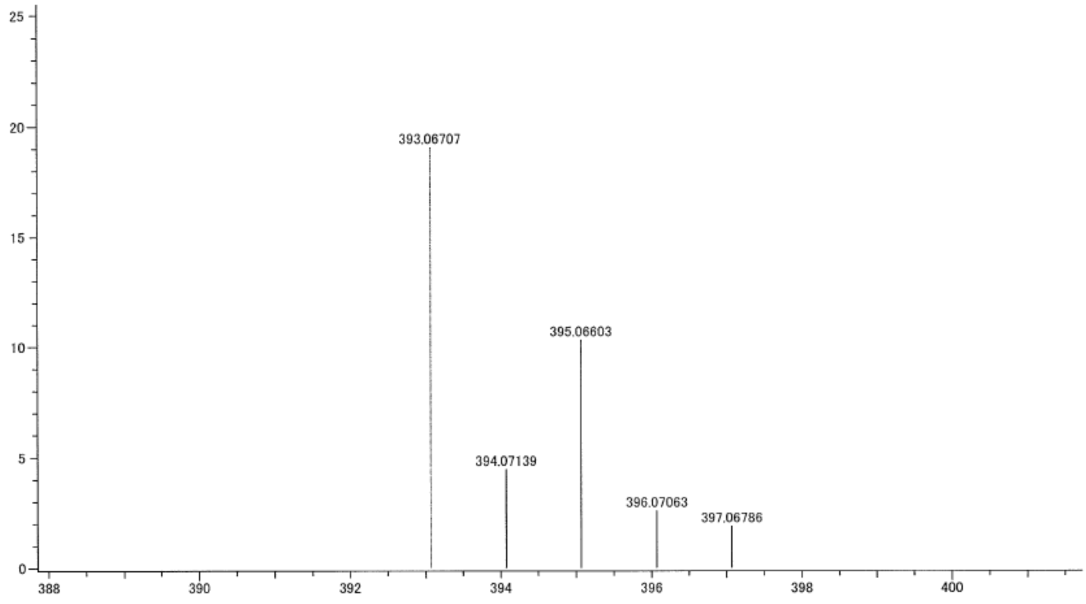

^{1}H-NMR spectrum of **3h** (DMSO-d_6, 600 MHz)

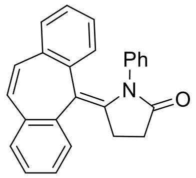

3h

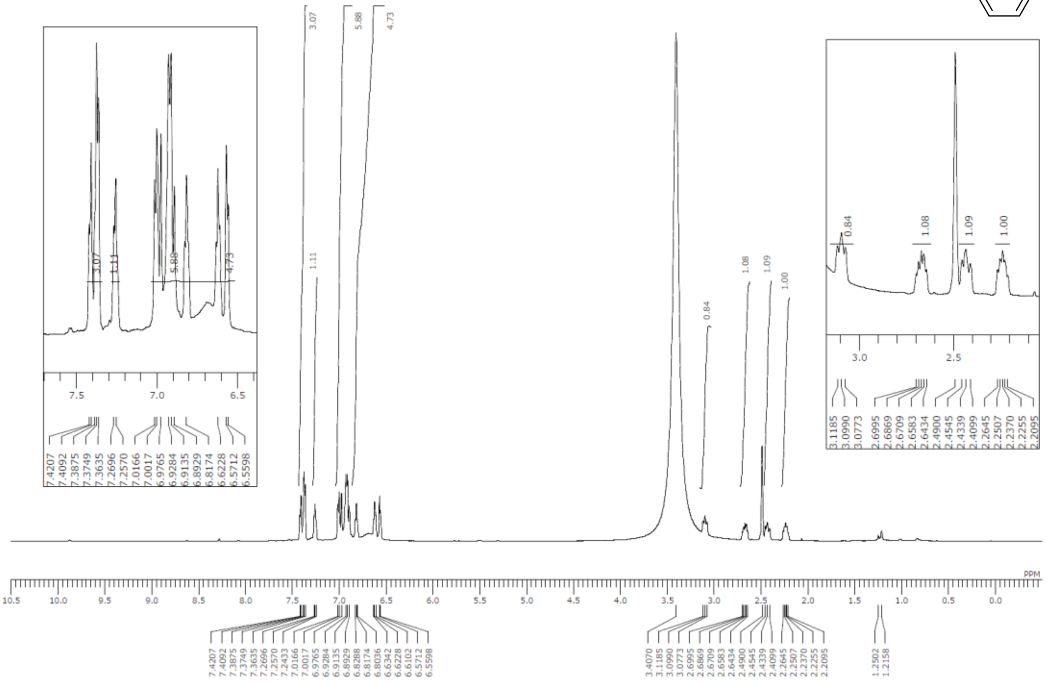

^{13}C-NMR spectrum of **3h** (DMSO-d_6, 150 MHz)

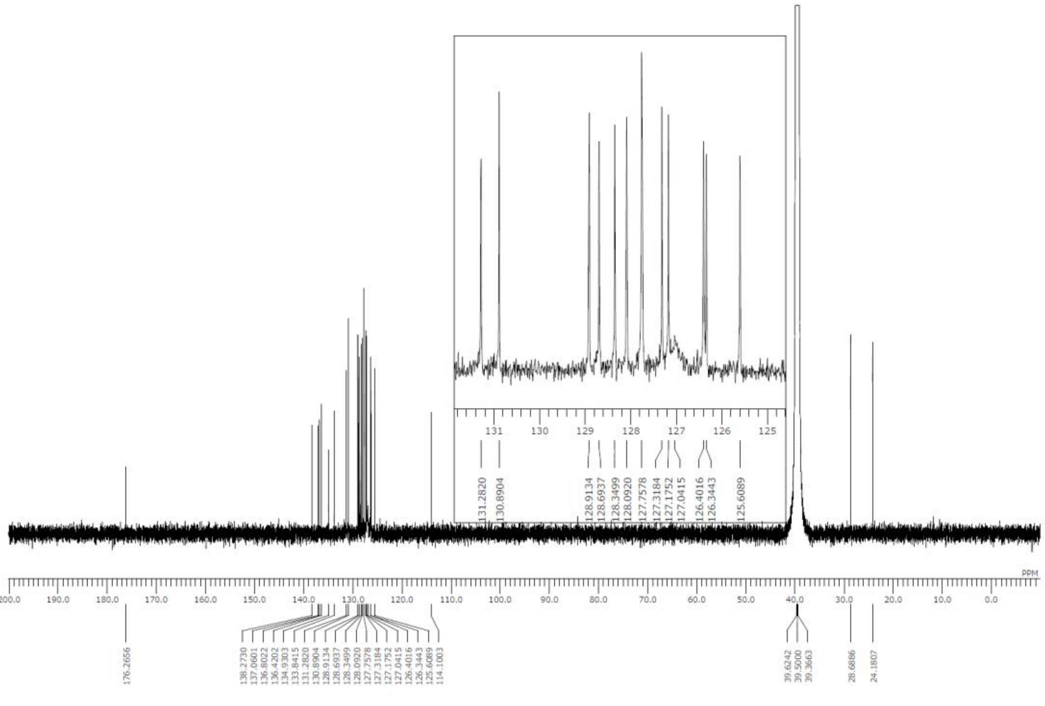

IR spectrum of **3h**

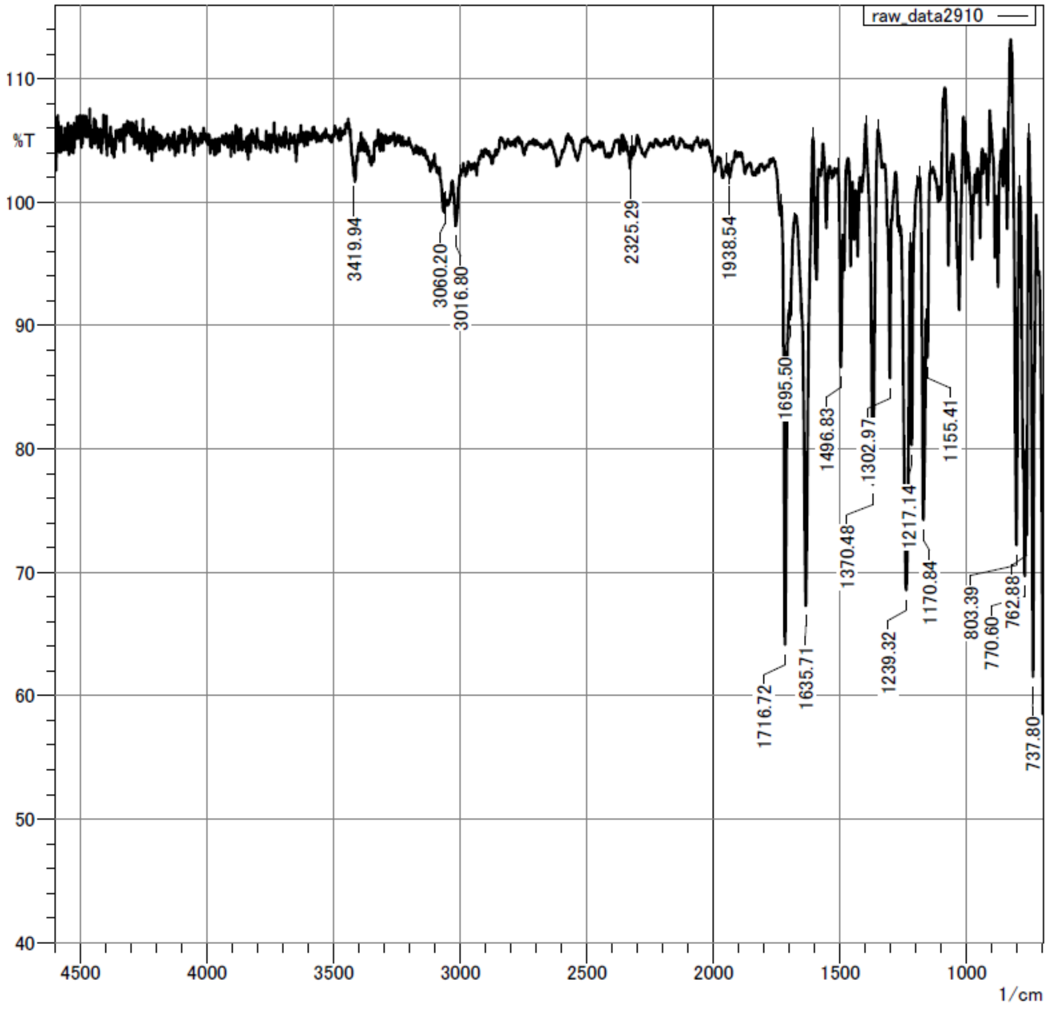

HRMS spectrum of **3h**

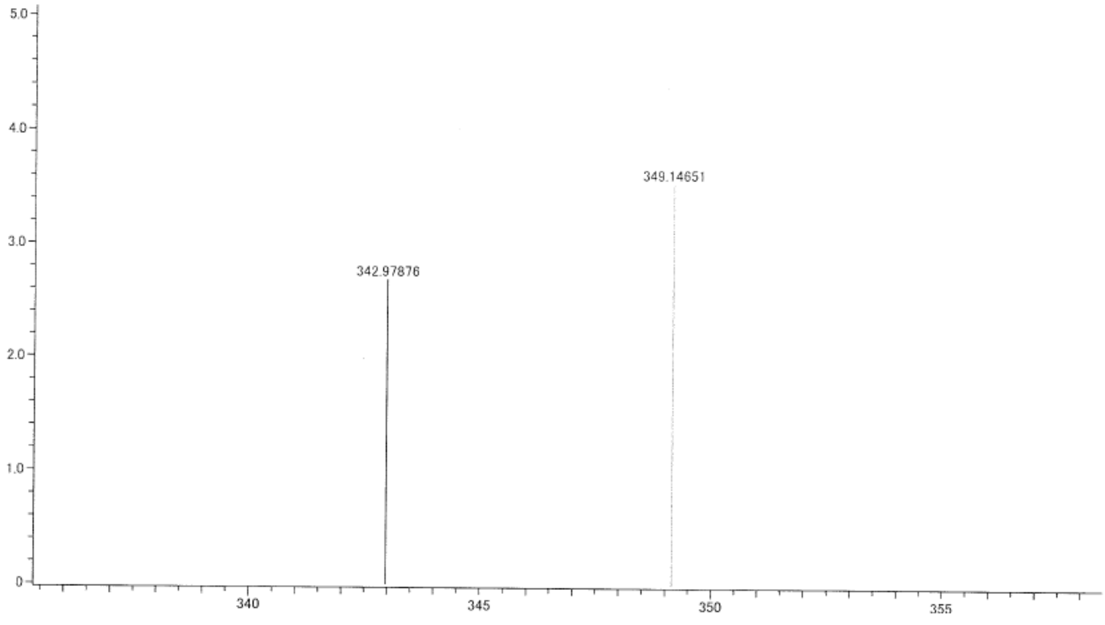

^{1}H-NMR spectrum of **3i** (**3i** : **2i** = 93 : 7, $CDCl_3$, 400 MHz)

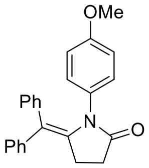

3i

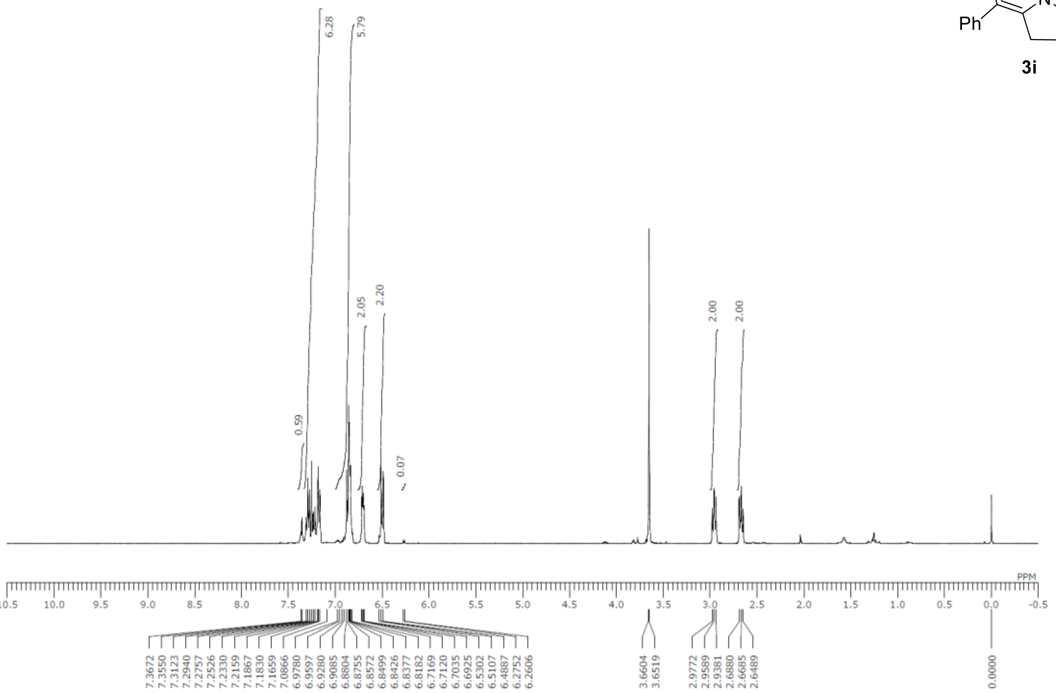

^{1}H-NMR spectrum of **3i** ($CDCl_3$, 600 MHz)

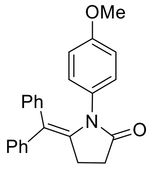

3i

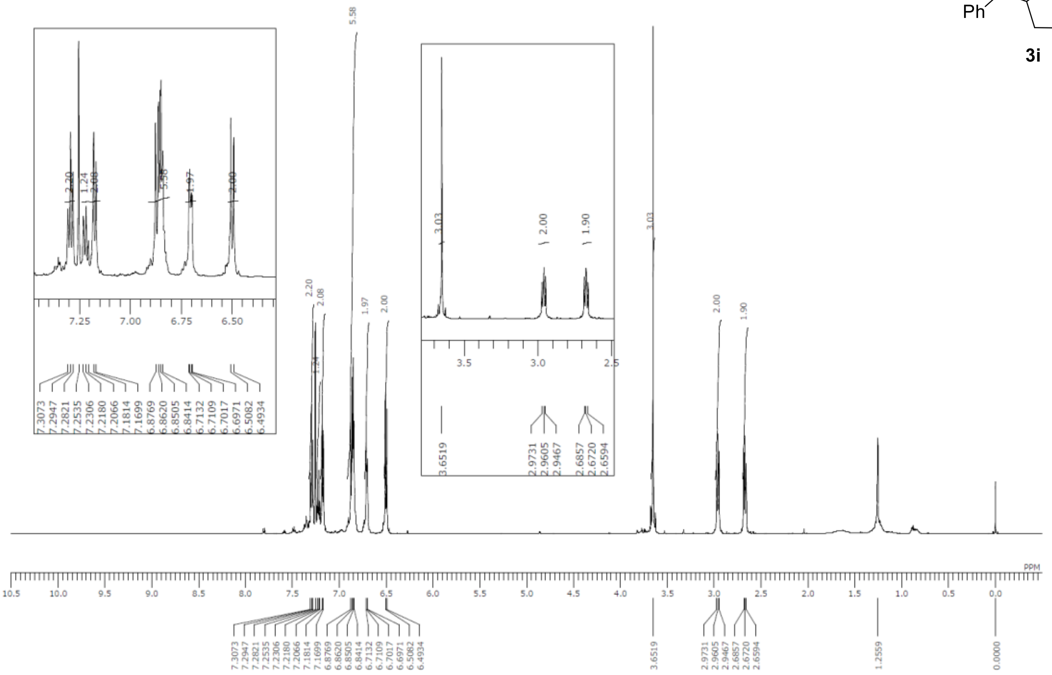

^{13}C-NMR spectrum of **3i** ($CDCl_3$, 150 MHz)

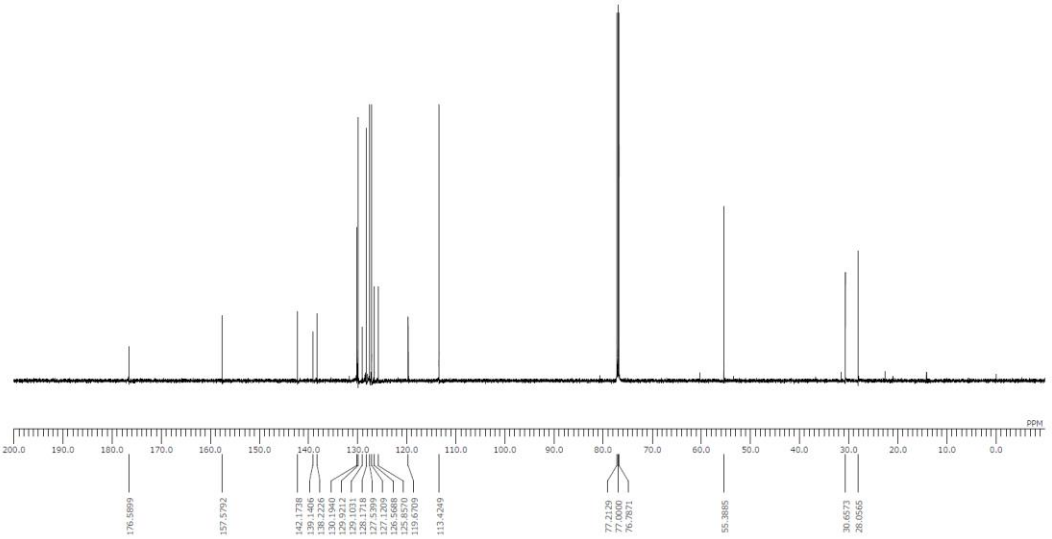

IR spectrum of **3i**

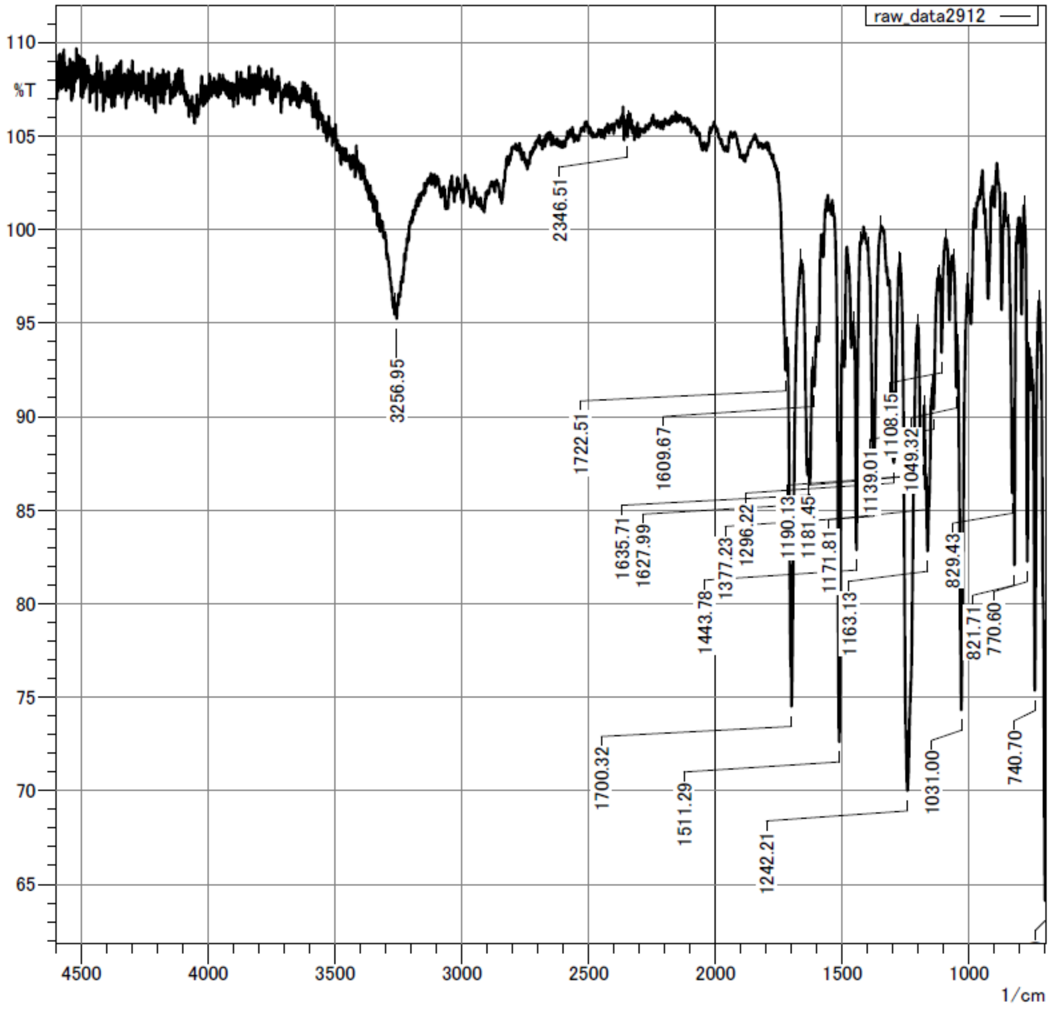

HRMS spectrum of **3i**

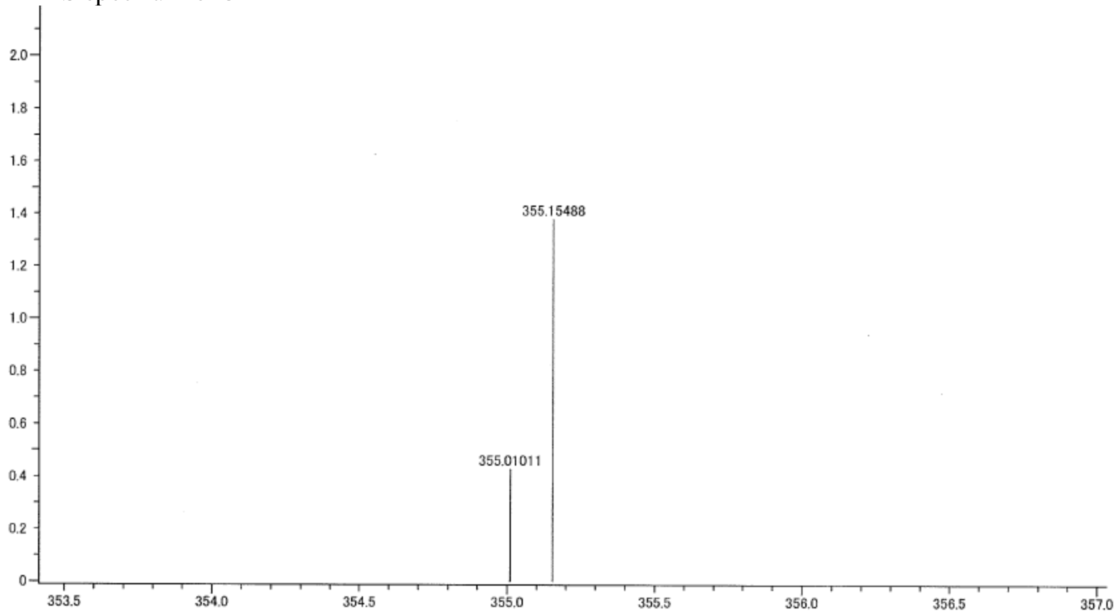

^{1}H-NMR spectrum of **3j** (**3j** : **2j** = 93 : 7, $CDCl_3$, 400 MHz)

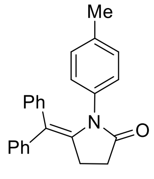

3j

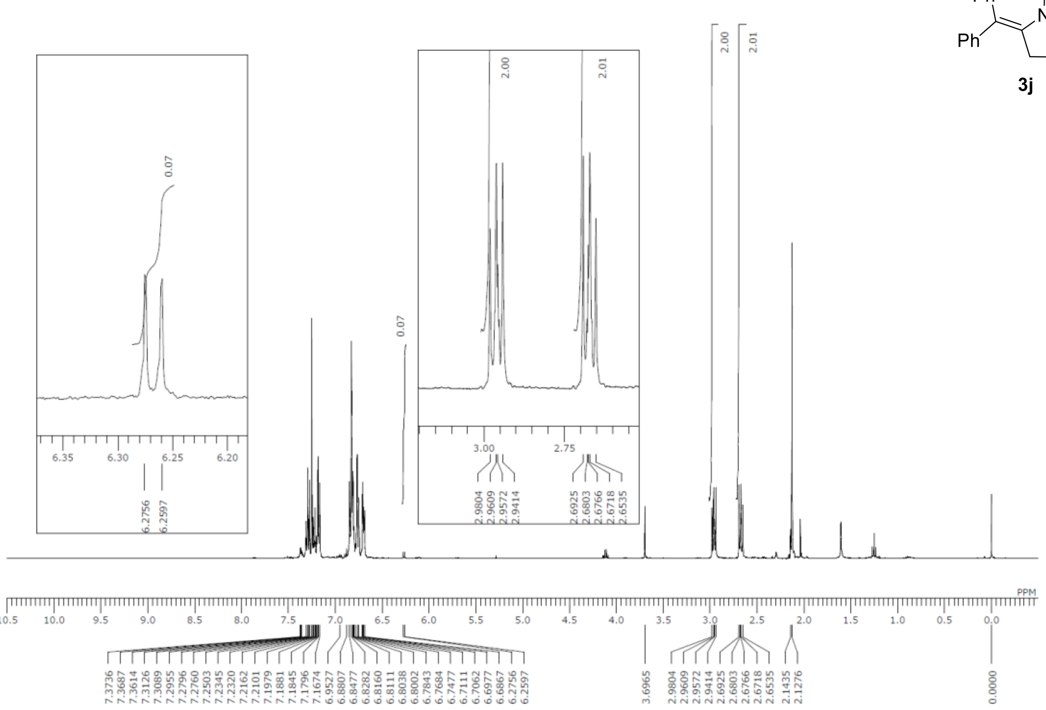

^{1}H-NMR spectrum of **3j** ($CDCl_3$, 600 MHz)

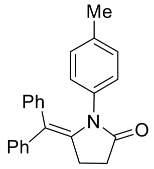

3j

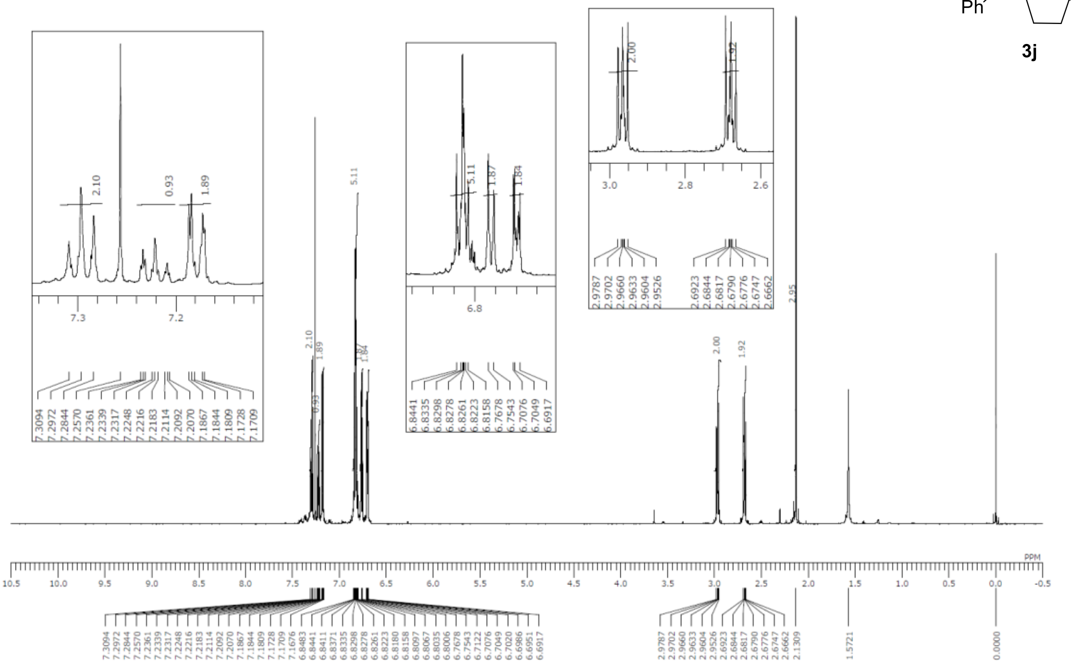

^{13}C-NMR spectrum of **3j** ($CDCl_3$, 150 MHz)

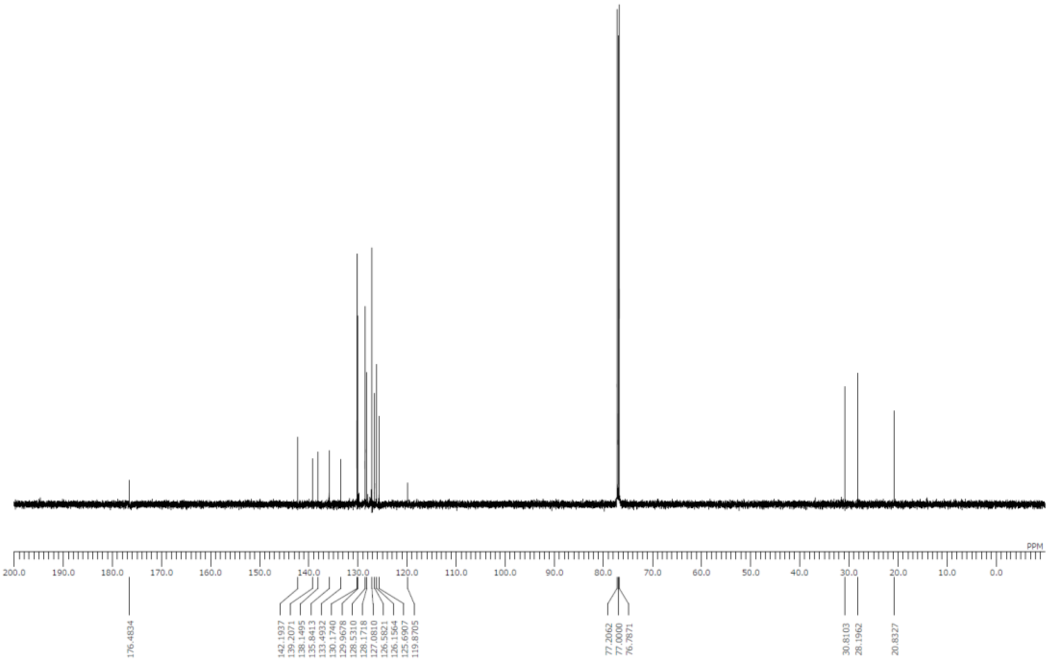

IR spectrum of **3j**

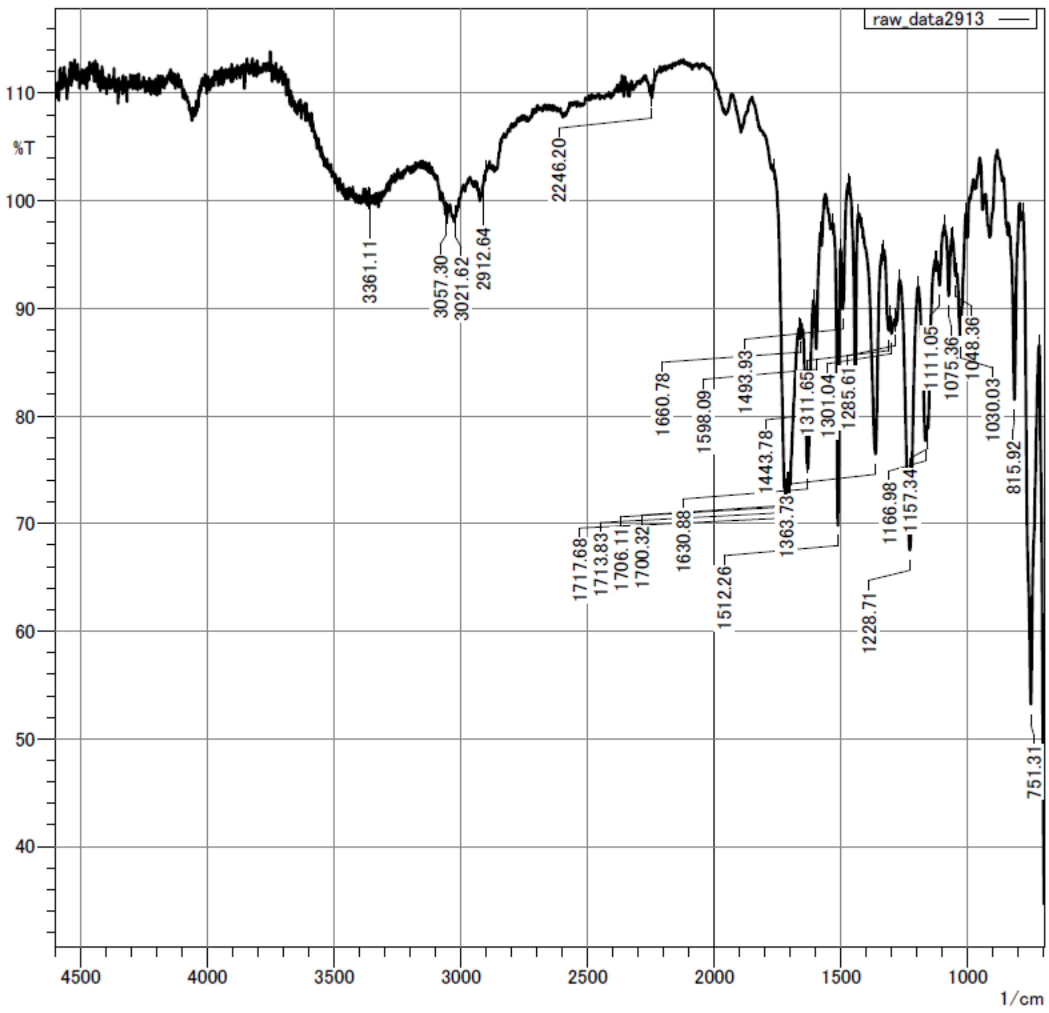

HRMS spectrum of **3j**

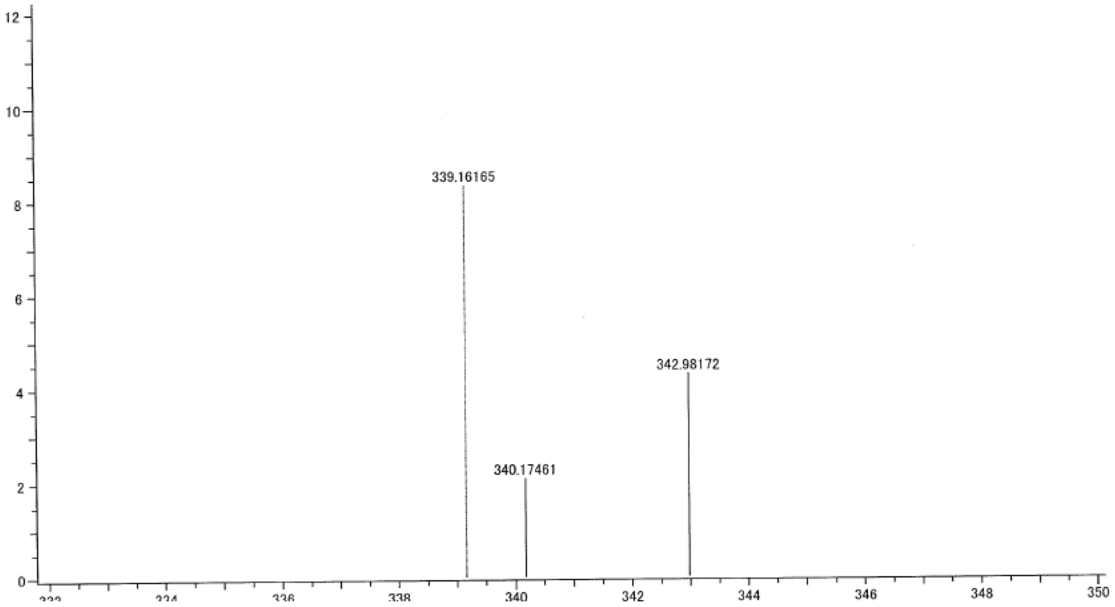

^{1}H-NMR spectrum of **3k** (**3k** : **2k** = 92 : 8, $CDCl_3$, 400 MHz)

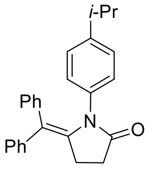

3k

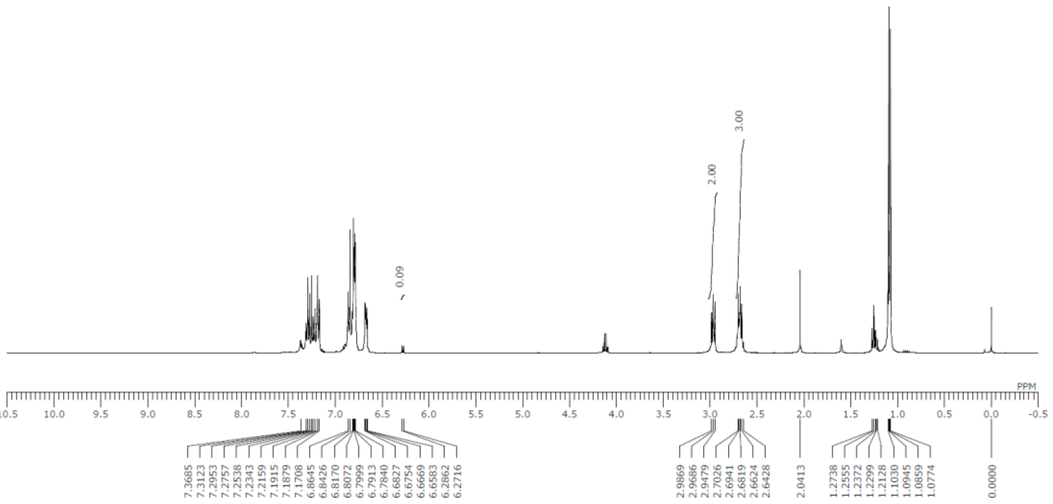

^{1}H-NMR spectrum of **3k** ($CDCl_3$, 600 MHz)

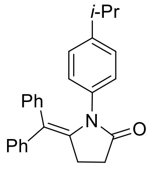

3k

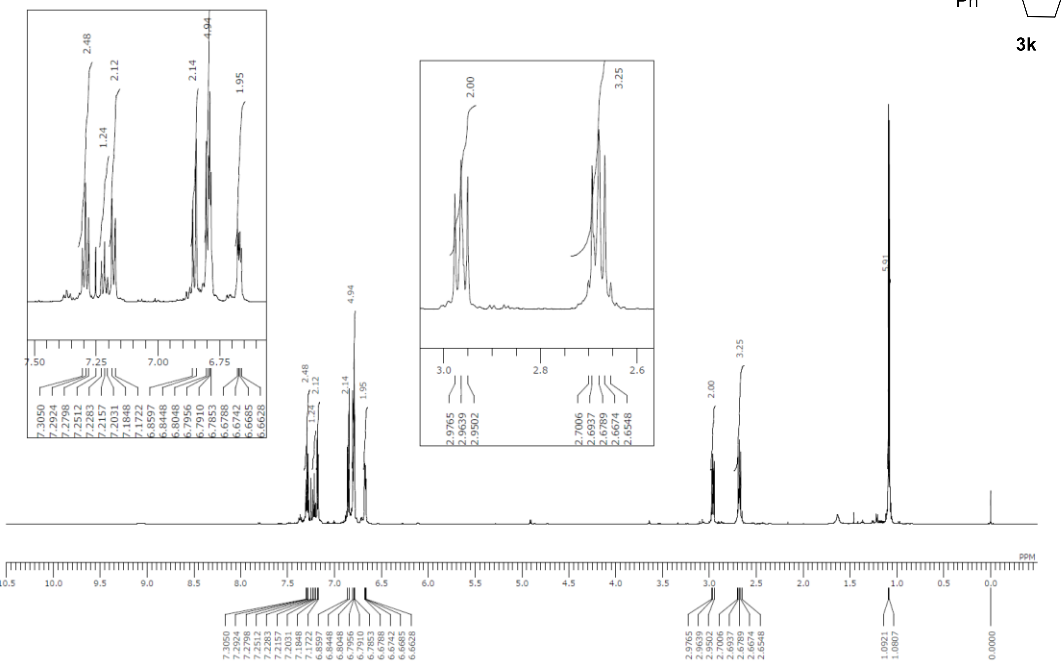

^{13}C-NMR spectrum of **3k** ($CDCl_3$, 150 MHz)

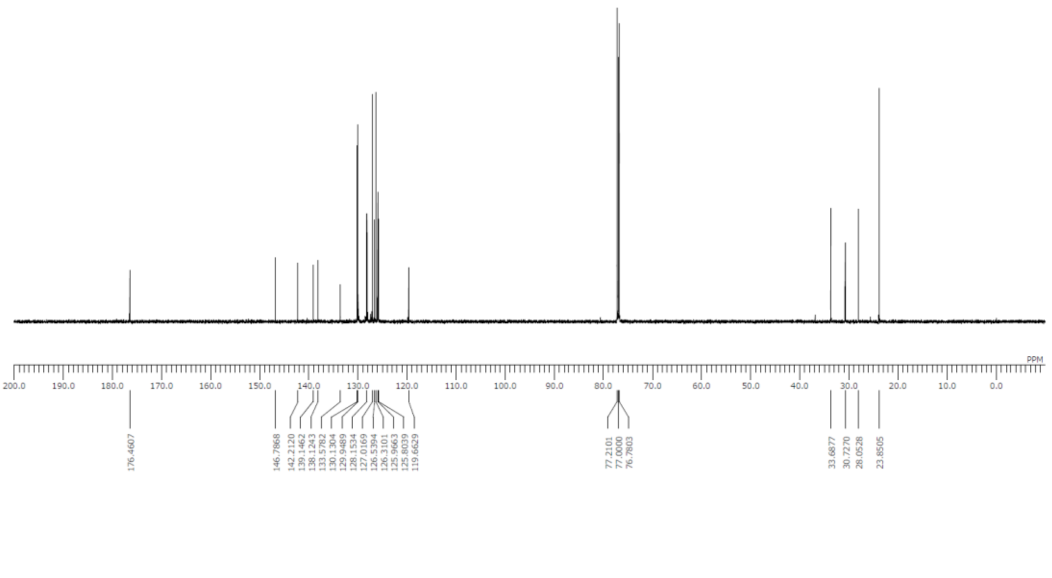

IR spectrum of **3k**

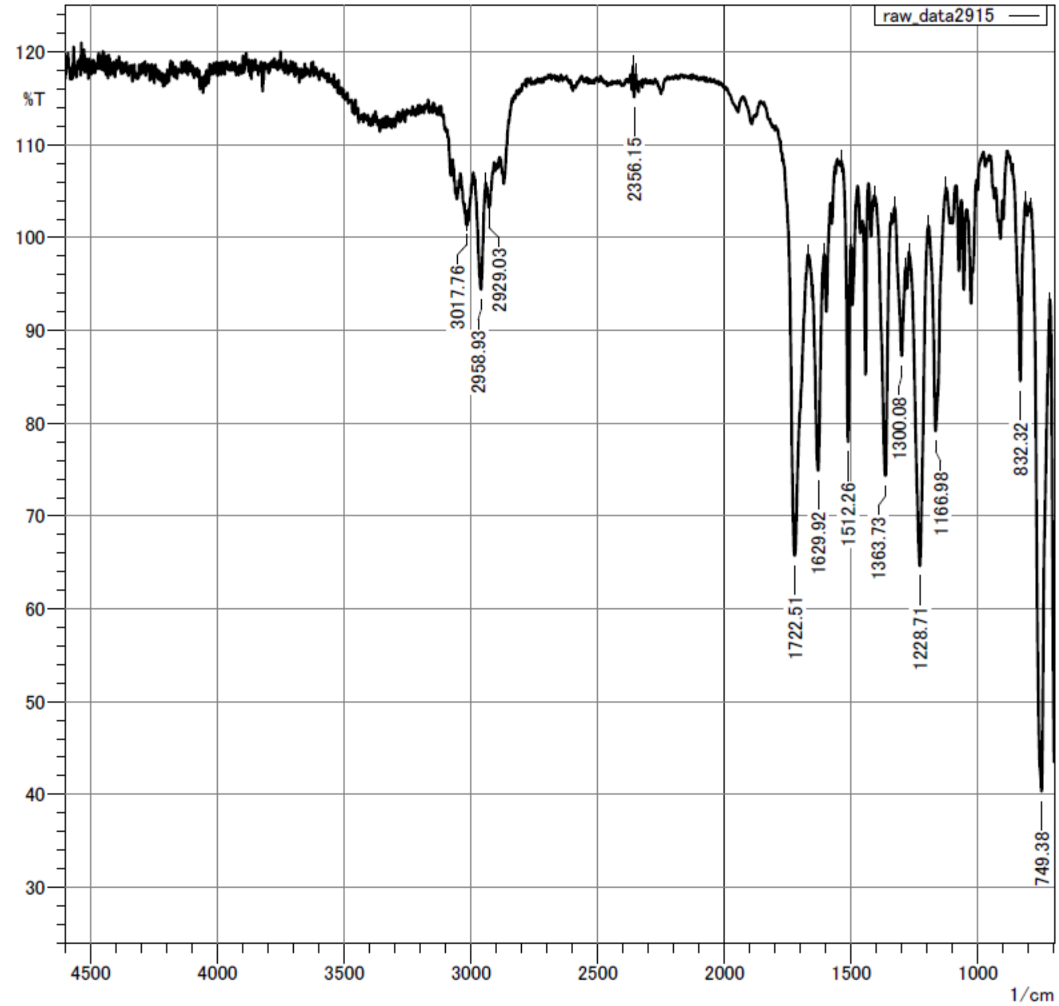

HRMS spectrum of **3k**

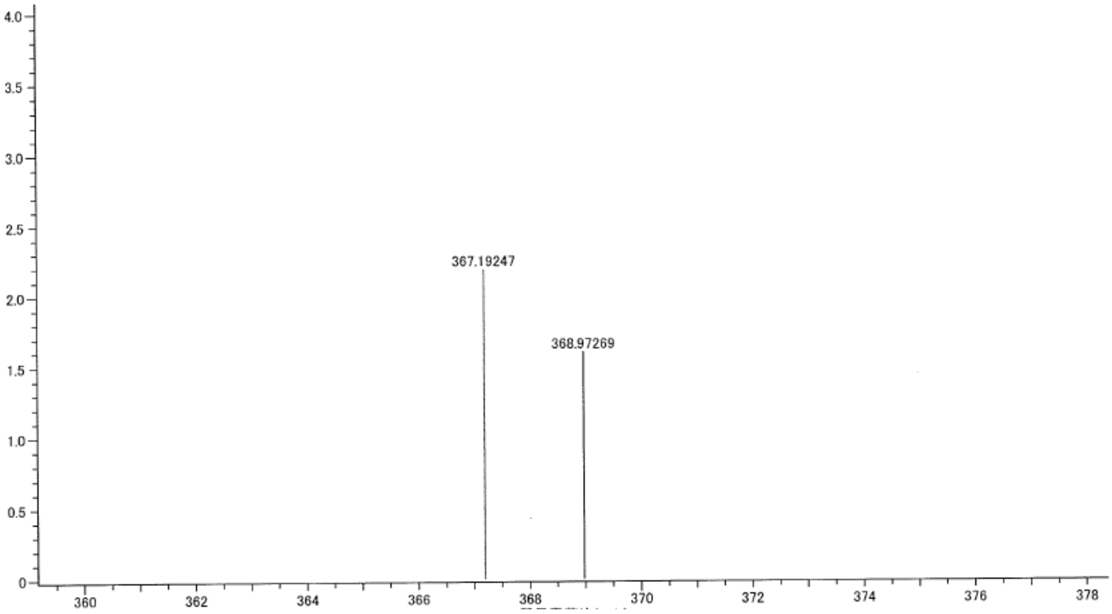

^{1}H-NMR spectrum of **3l** (**3l** : **2l** = 93 : 7, $CDCl_3$, 400 MHz)

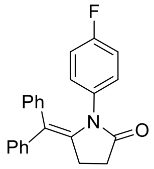

3l

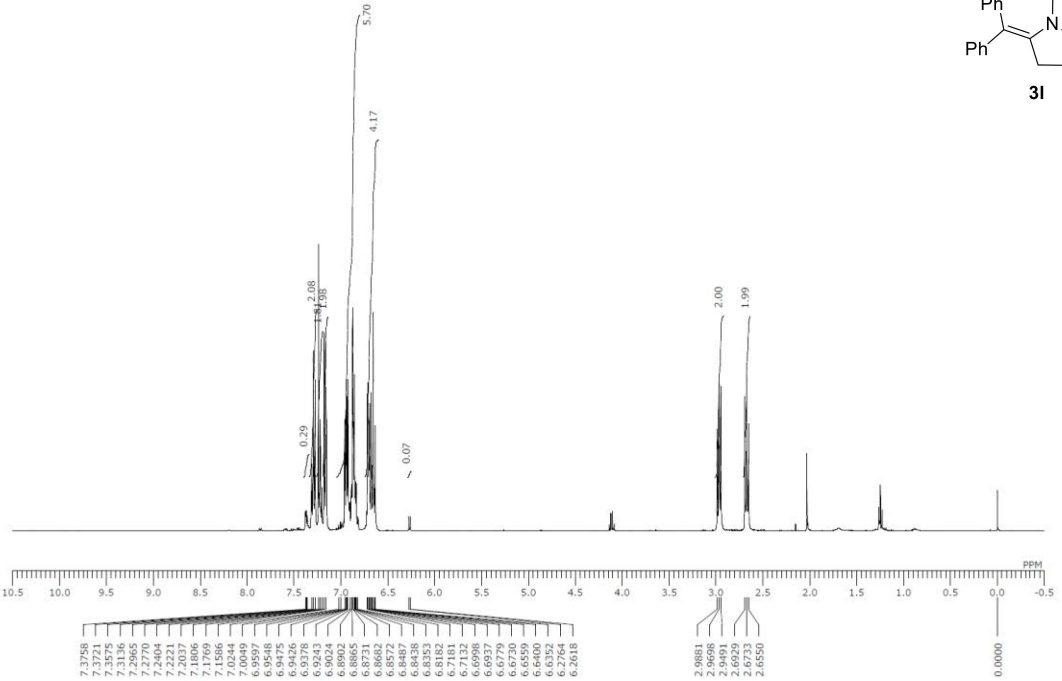

^{1}H-NMR spectrum of **3l** ($CDCl_3$, 600 MHz)

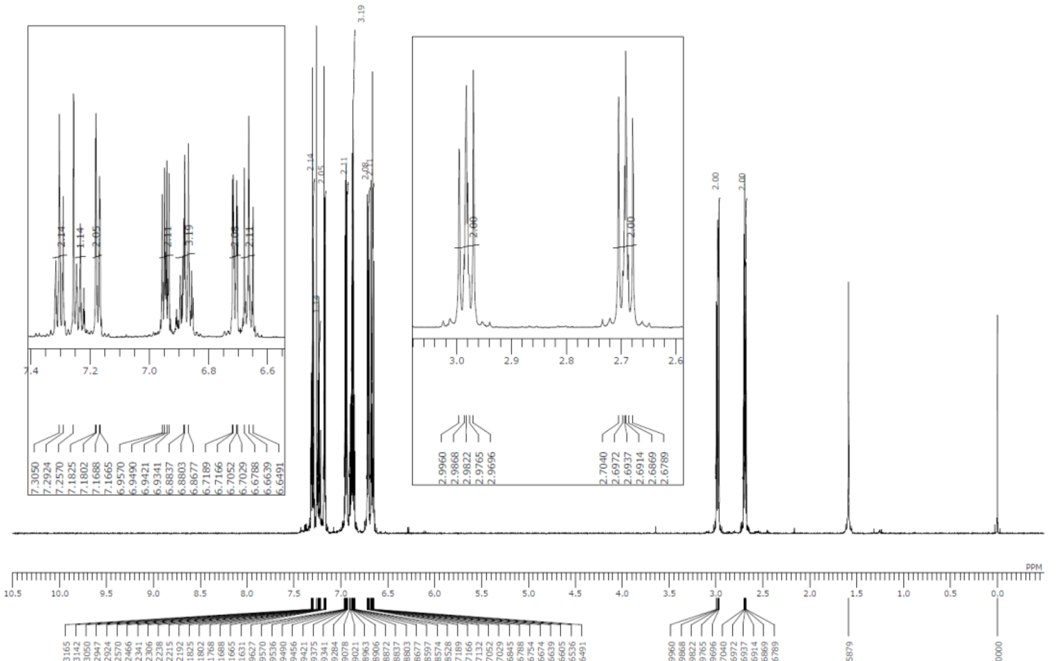

^{13}C-NMR spectrum of **3l** ($CDCl_3$, 150 MHz)

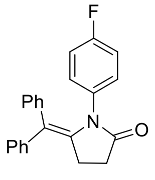

3l

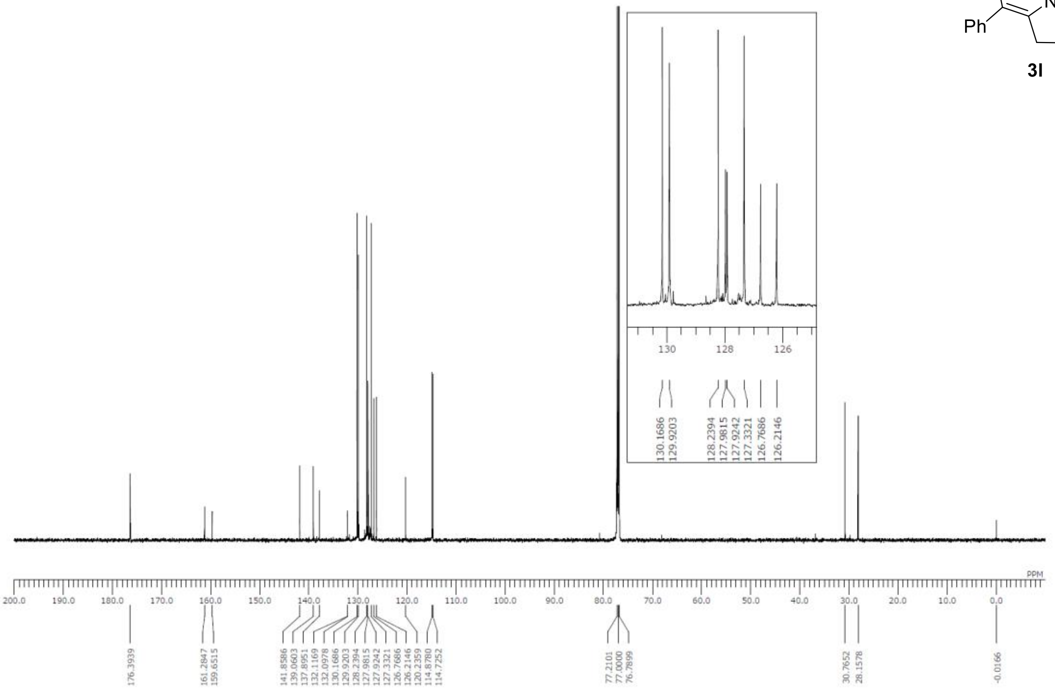

^{19}F-NMR spectrum of **3l** ($CDCl_3$, 565 MHz)

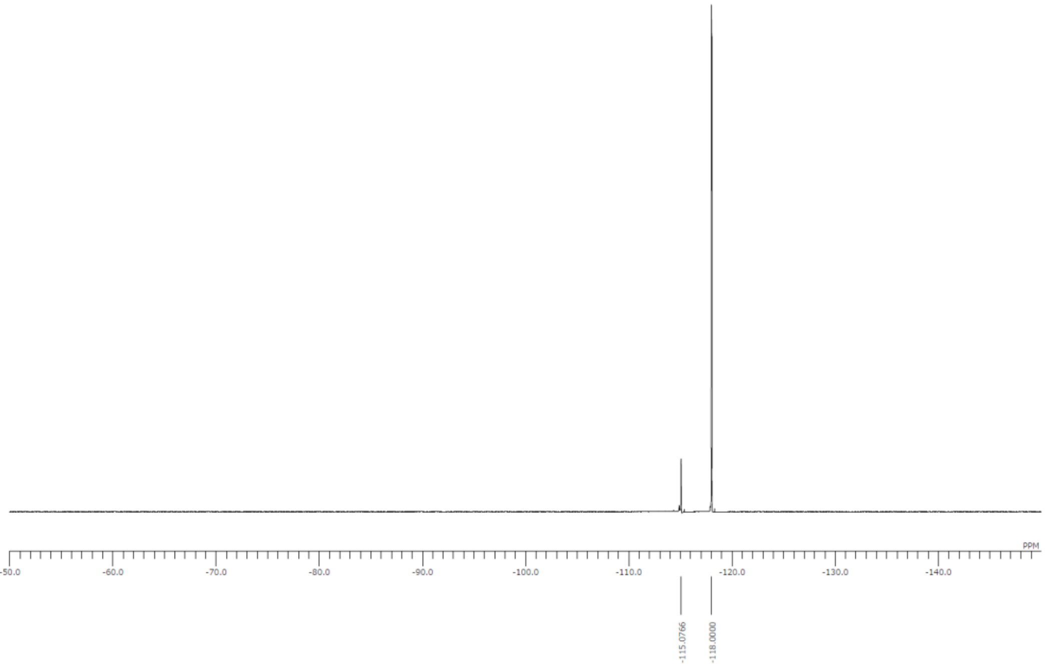

IR spectrum of **3l**

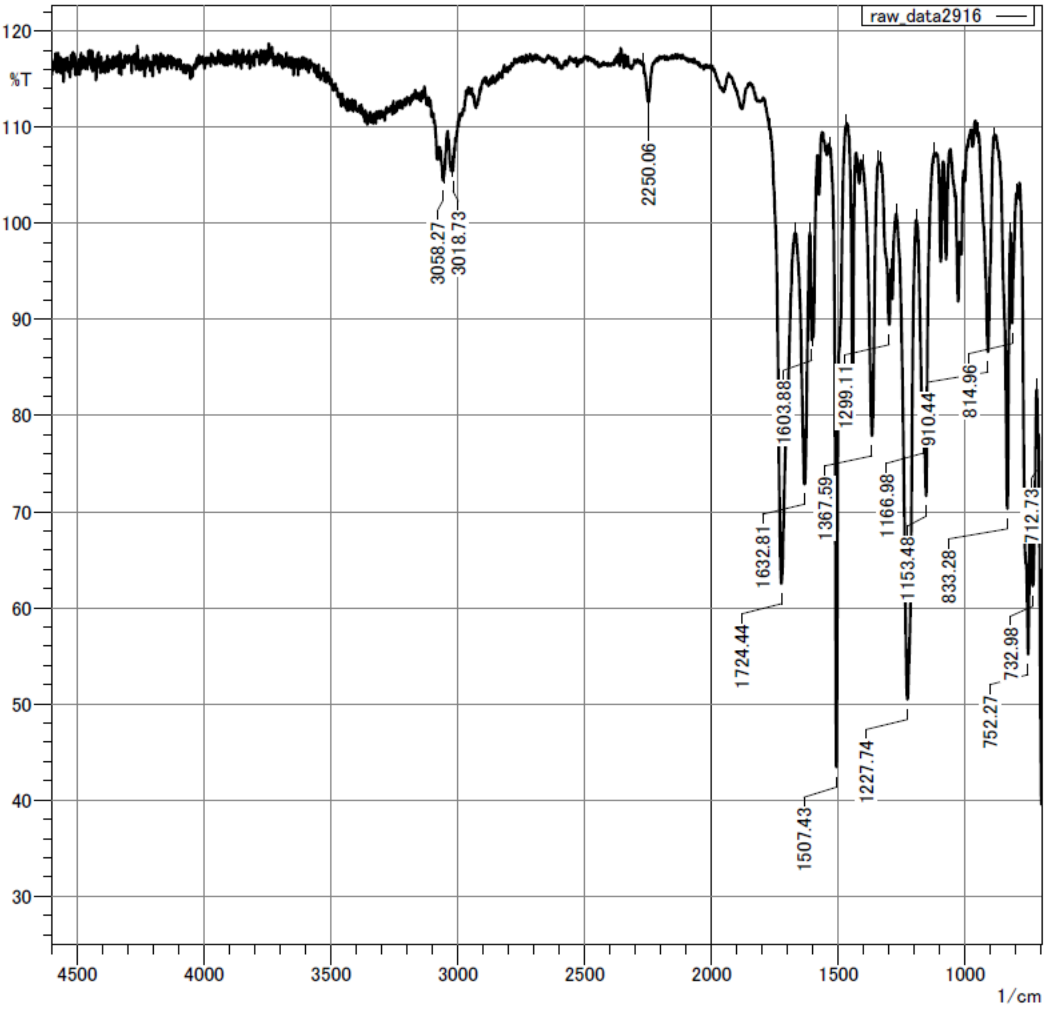

HRMS spectrum of **3l**

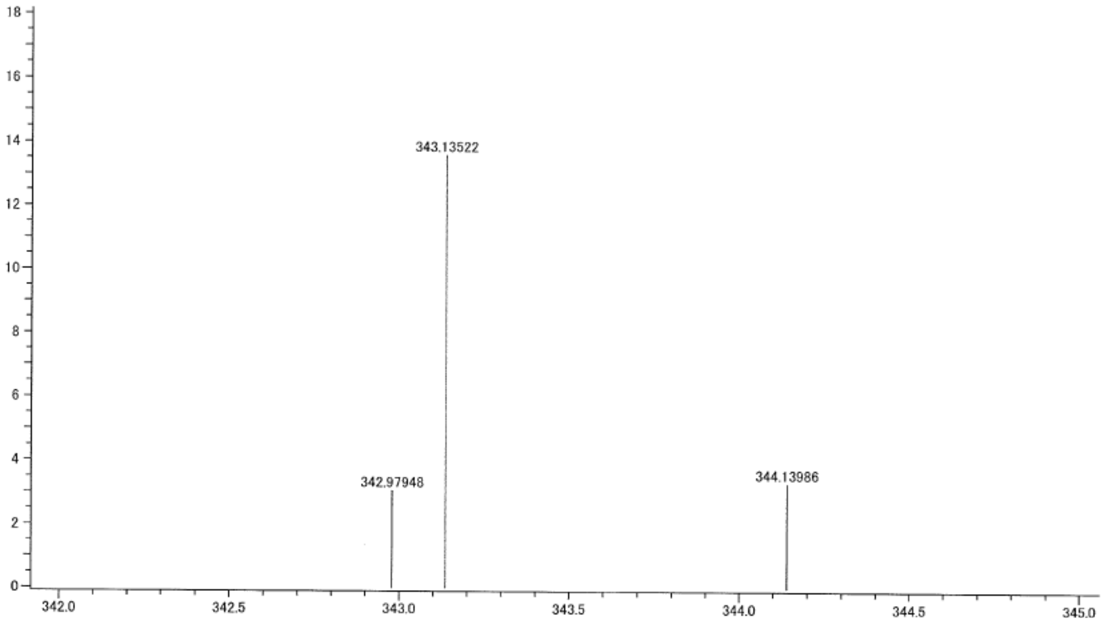

^{1}H-NMR spectrum of **3m** ($CDCl_3$, 600 MHz)

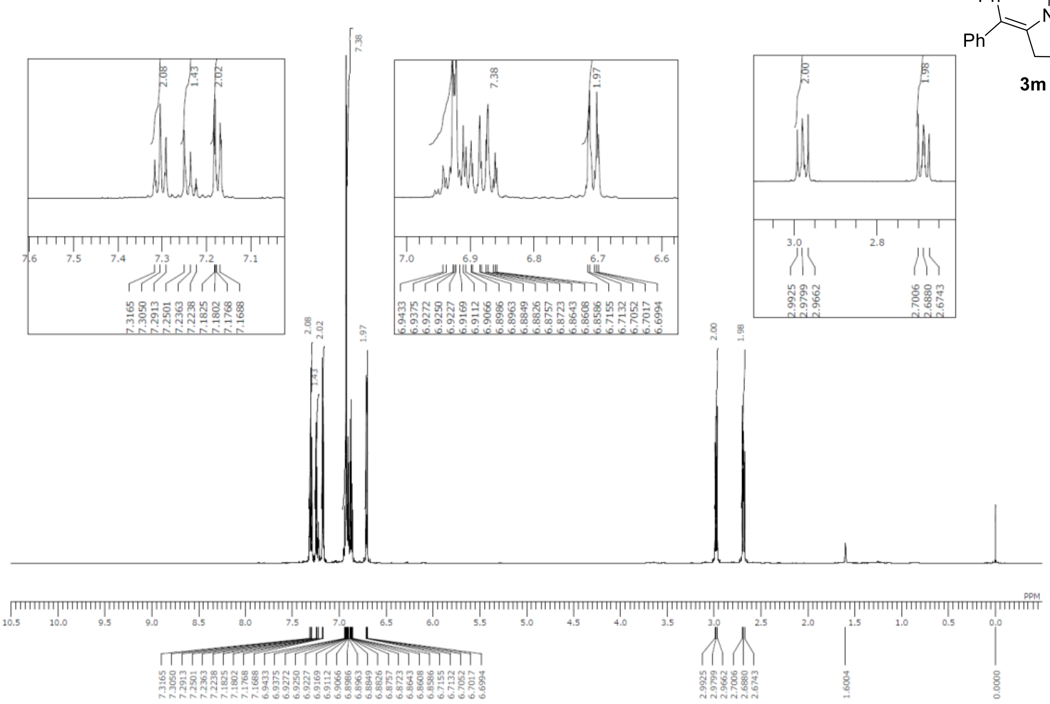

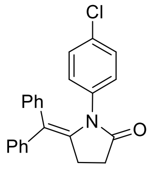

3m

^{13}C-NMR spectrum of **3m** ($CDCl_3$, 150 MHz)

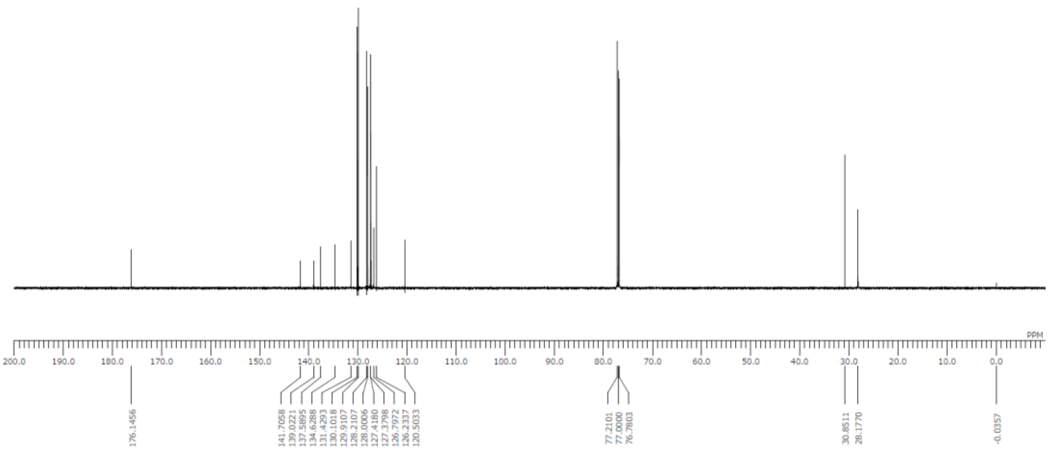

IR spectrum of **3m**

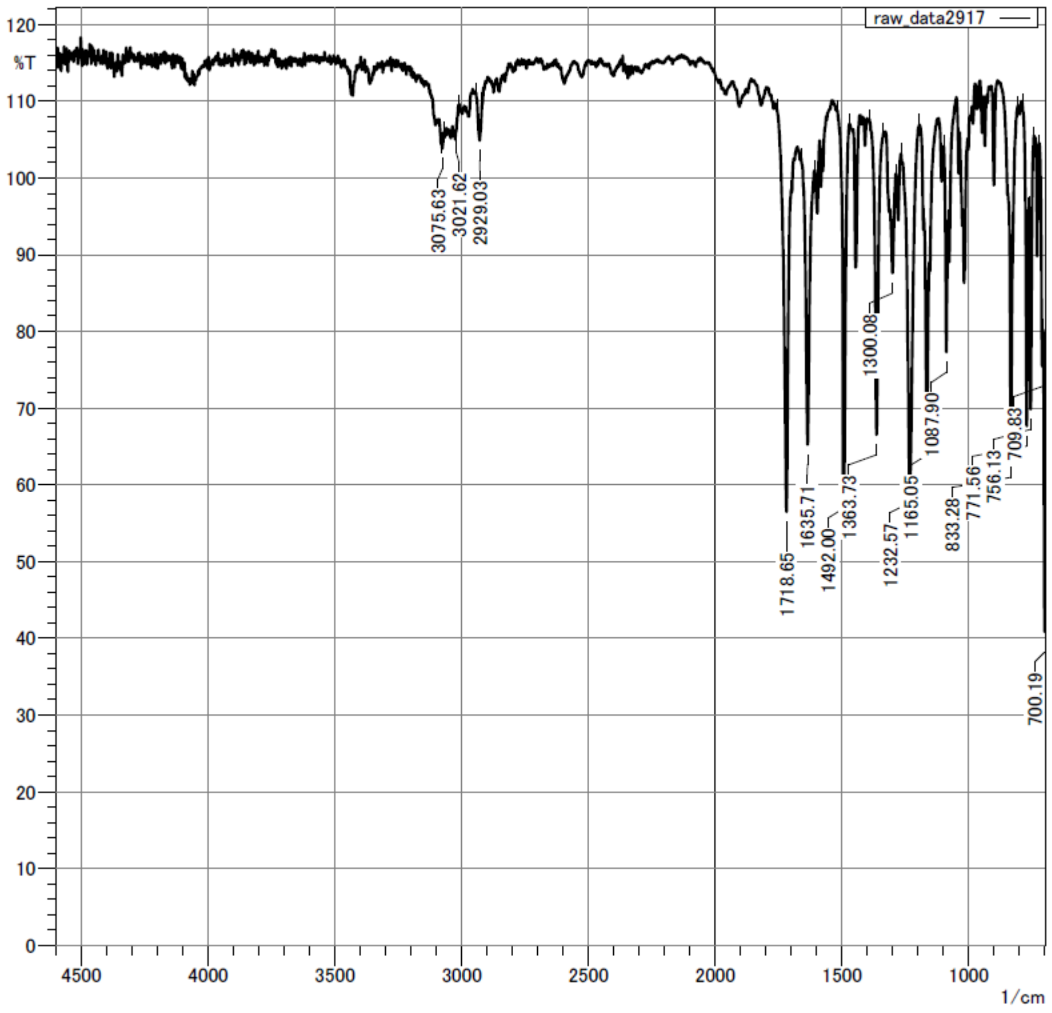

HRMS spectrum of **3m**

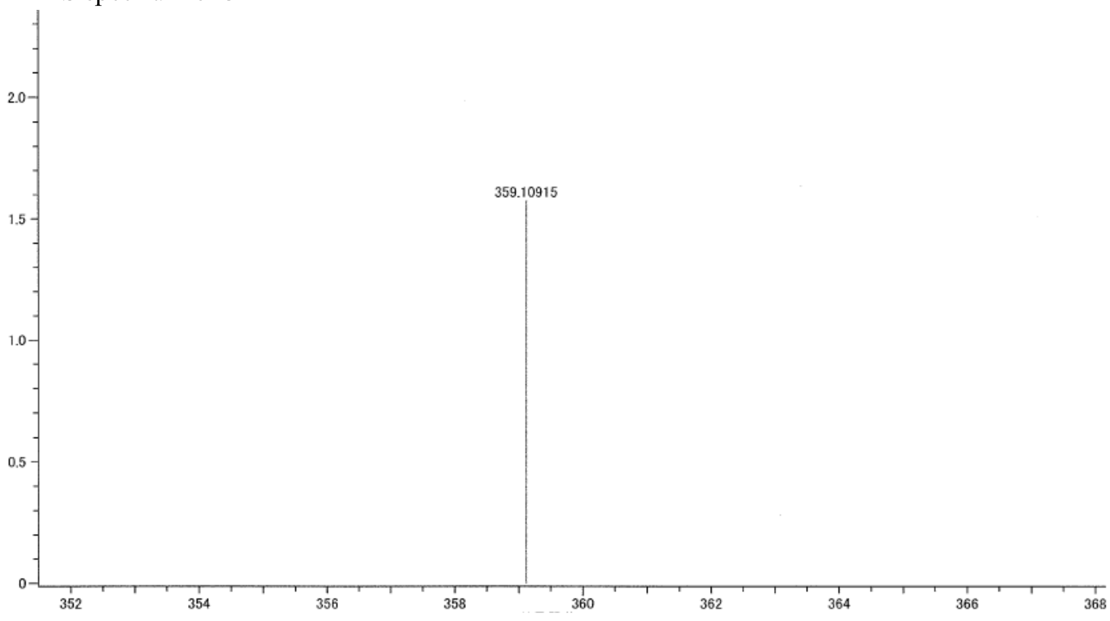

^{1}H-NMR spectrum of **3n** (**3n** : **2n** = 96 : 4, $CDCl_3$, 400 MHz)

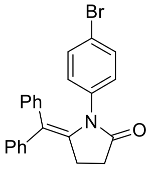

3n

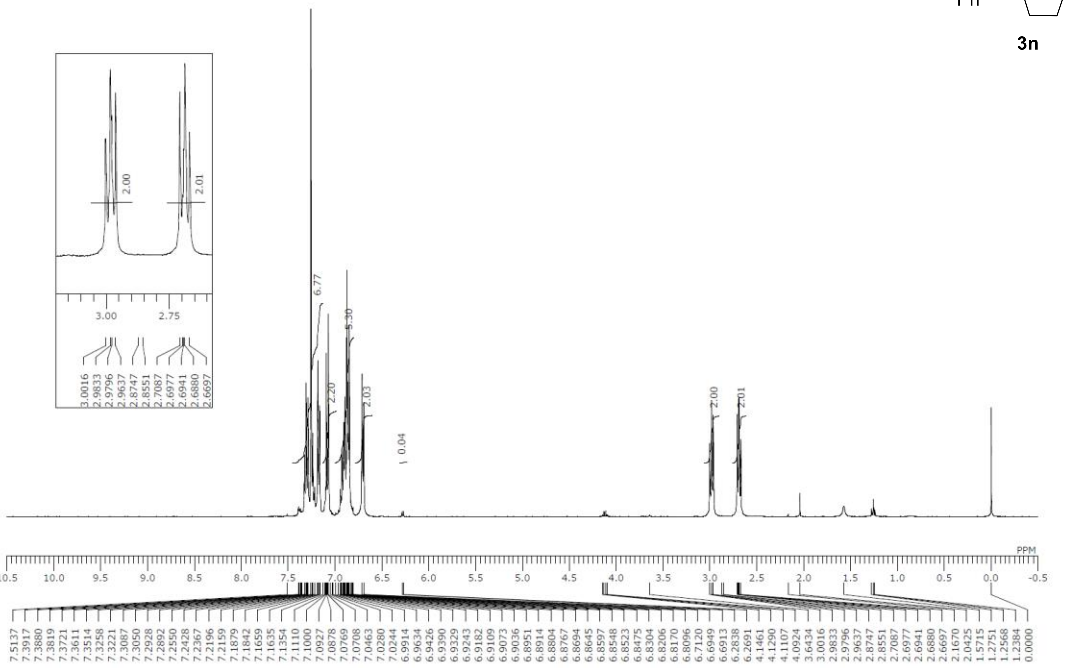

^{1}H-NMR spectrum of **3n** ($CDCl_3$, 600 MHz)

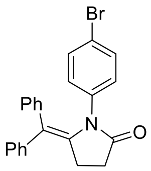

3n

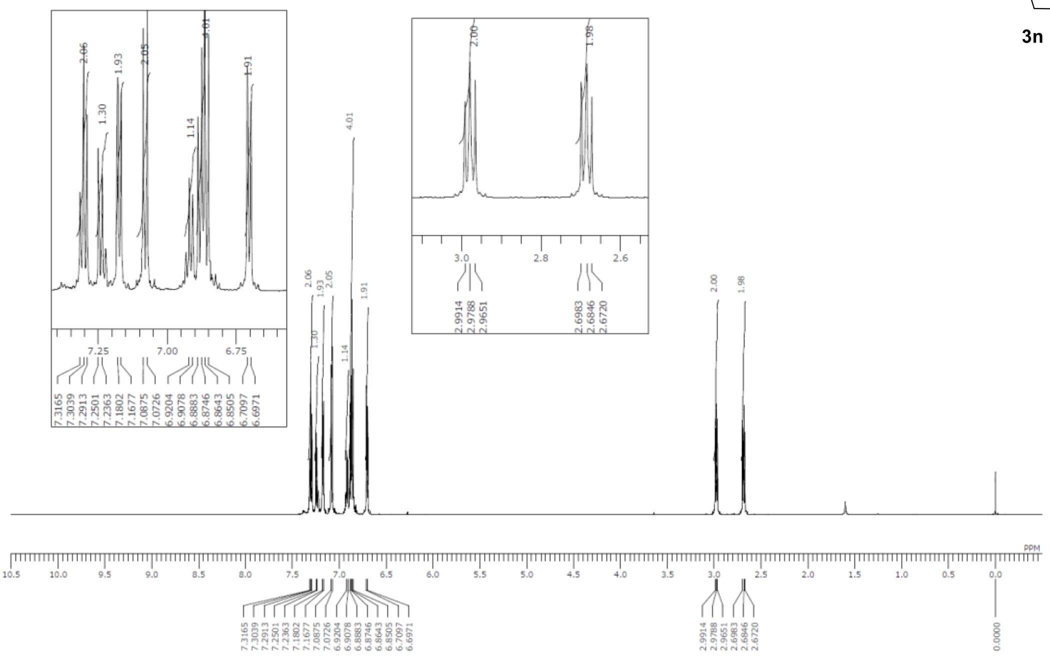

^{13}C-NMR spectrum of **3n** ($CDCl_3$, 150 MHz)

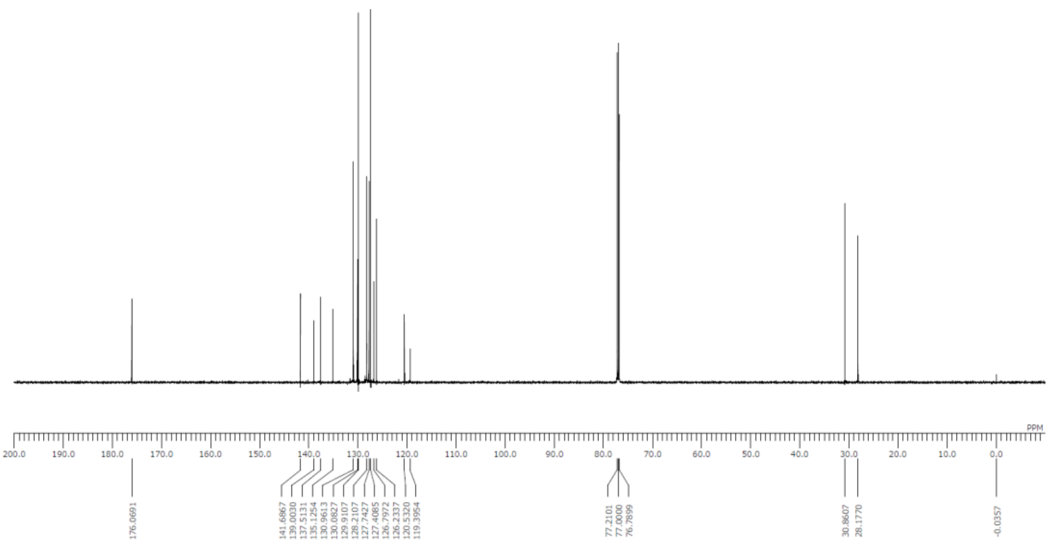

IR spectrum of **3n**

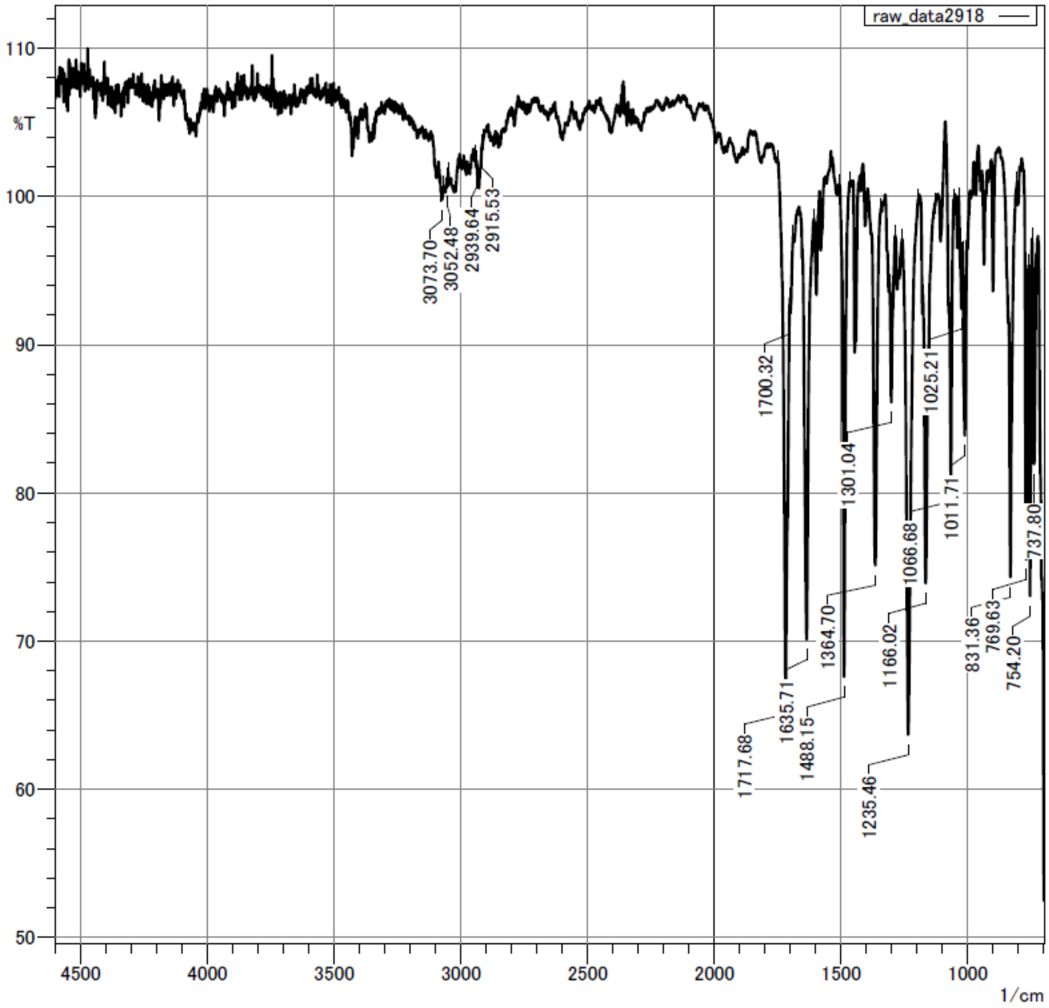

HRMS spectrum of **3n**

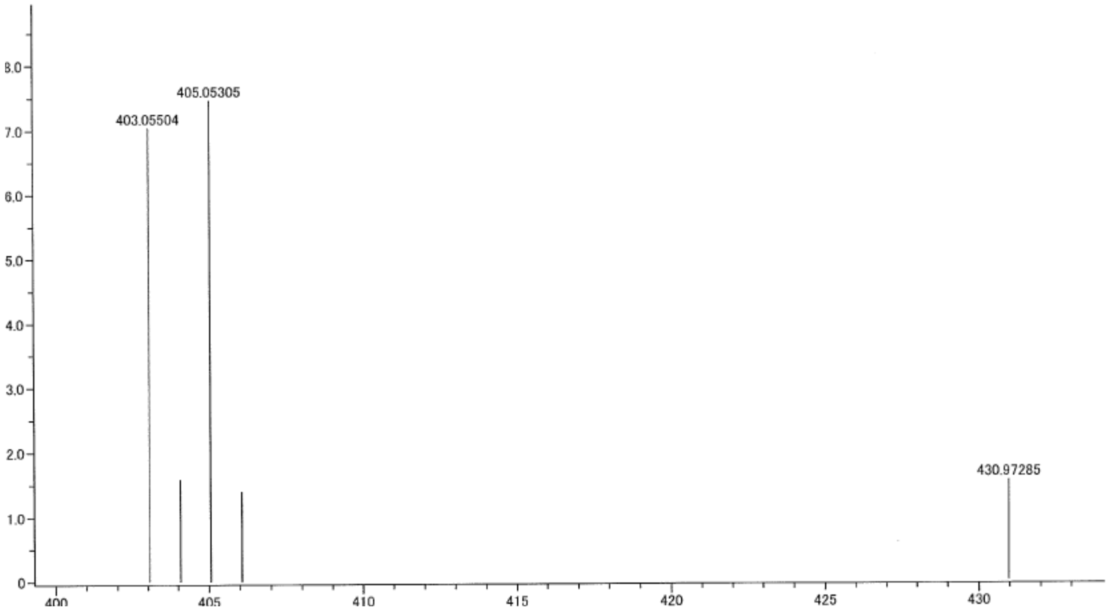

^{1}H-NMR spectrum of **3o** (**3o** : **2o** = 95 : 5, $CDCl_3$, 400 MHz)

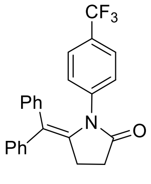

3o

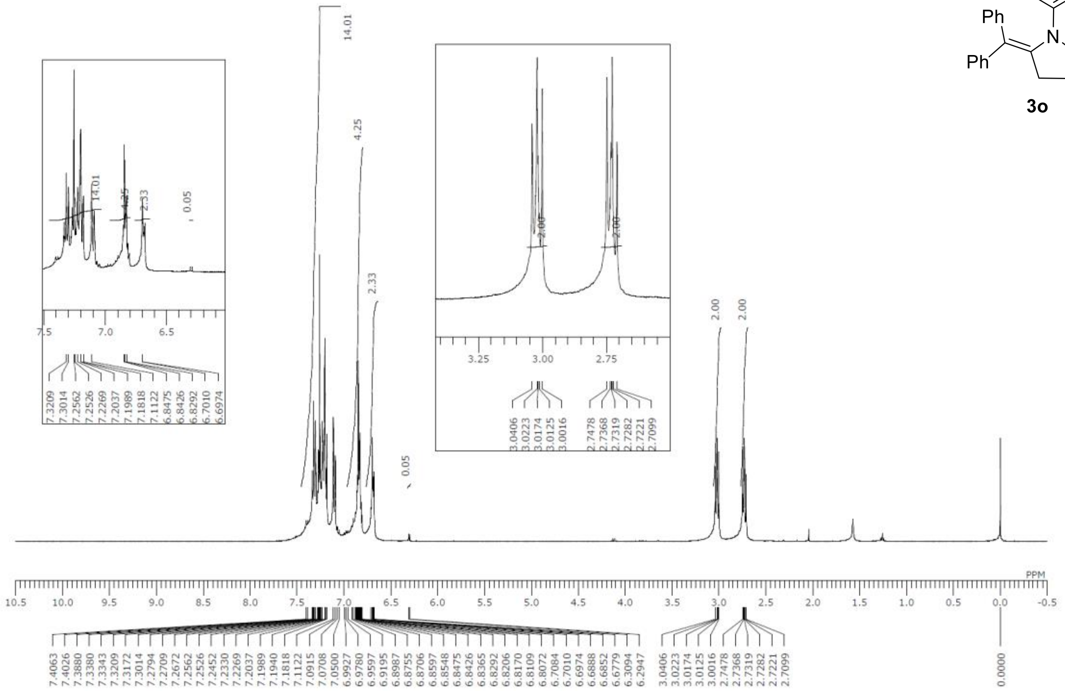

^{1}H-NMR spectrum of **3o** ($CDCl_3$, 600 MHz)

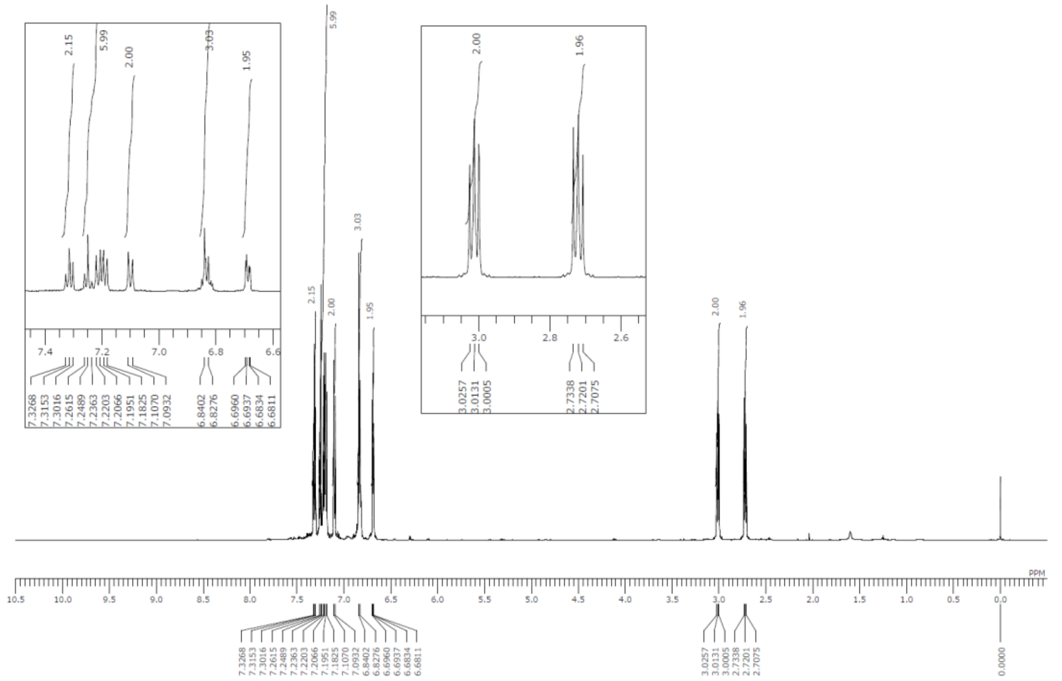

^{13}C-NMR spectrum of **3o** ($CDCl_3$, 150 MHz)

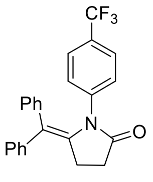

3o

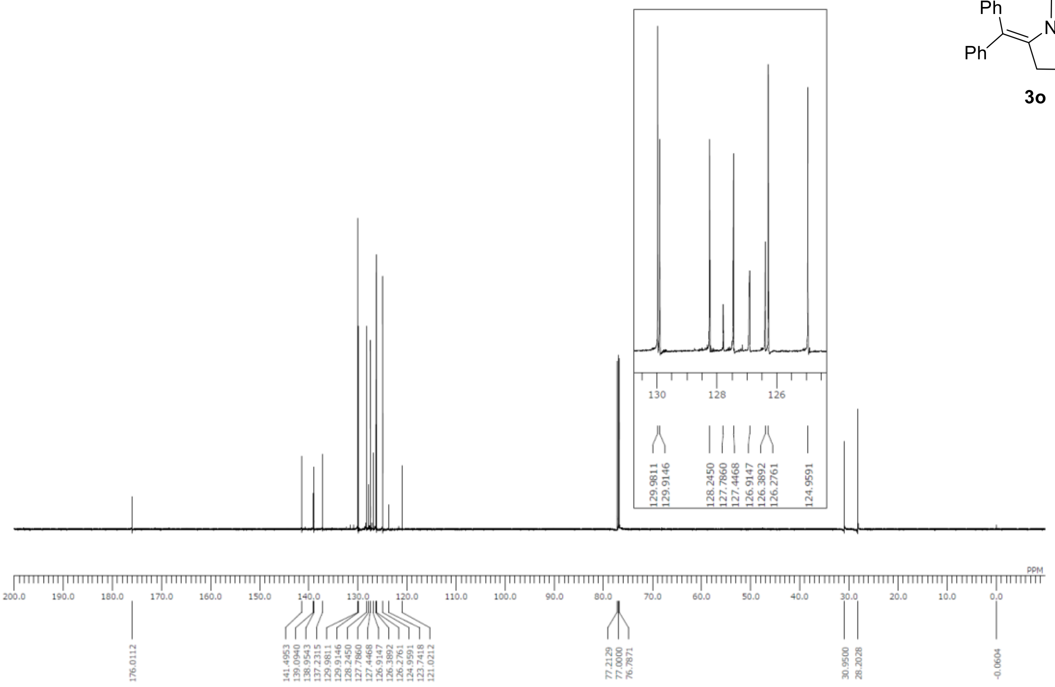

^{19}F-NMR spectrum of **3o** ($CDCl_3$, 565 MHz)

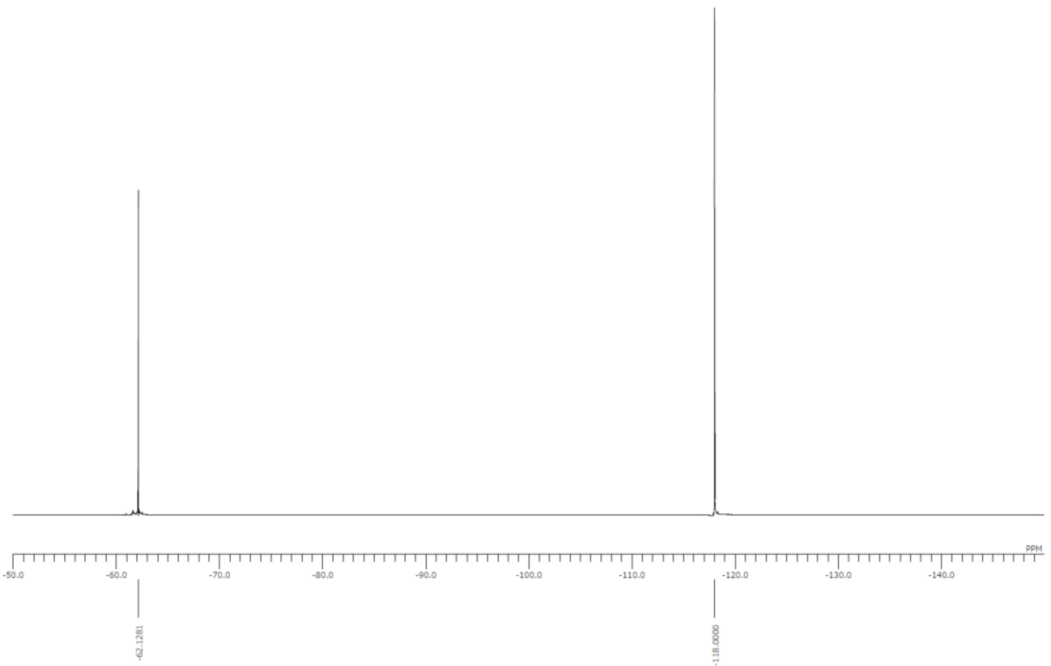

IR spectrum of **3o**

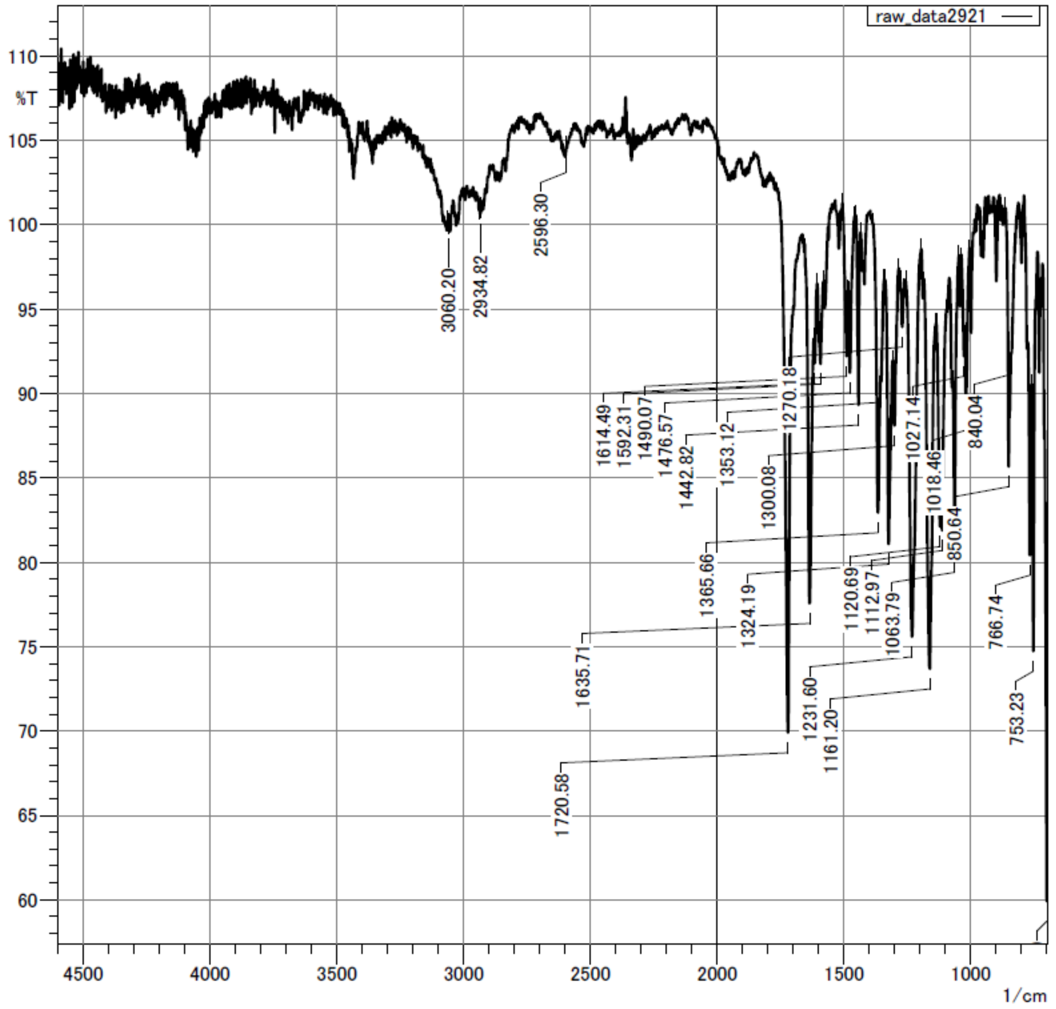

HRMS spectrum of **3o**

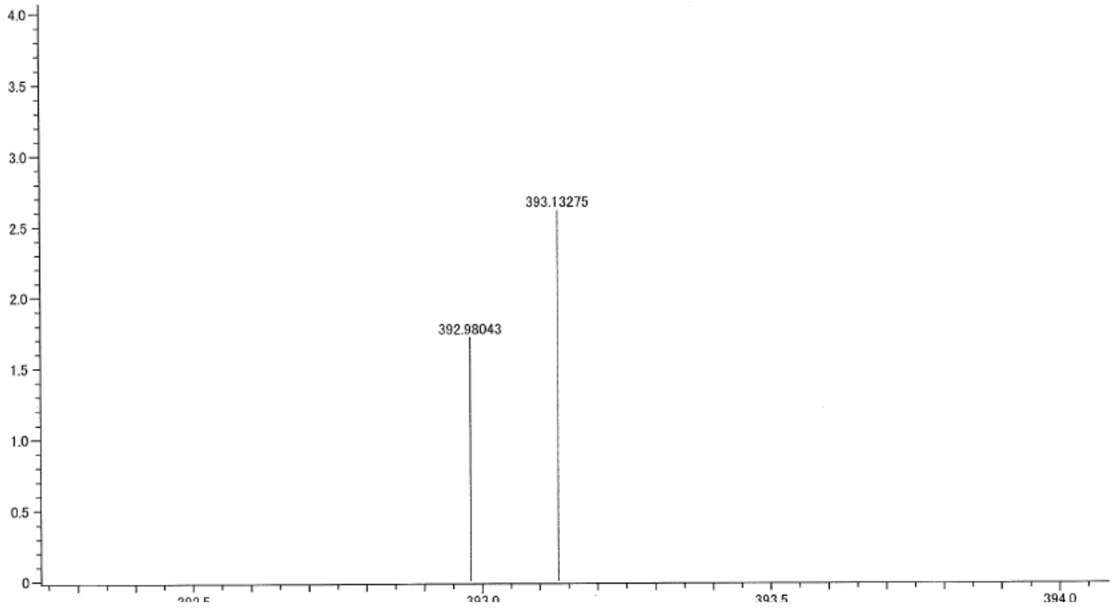

^{1}H-NMR spectrum of **3p** (**3p** : **2p** = 98 : 2, $CDCl_3$, 400 MHz)

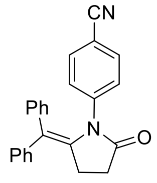

3p

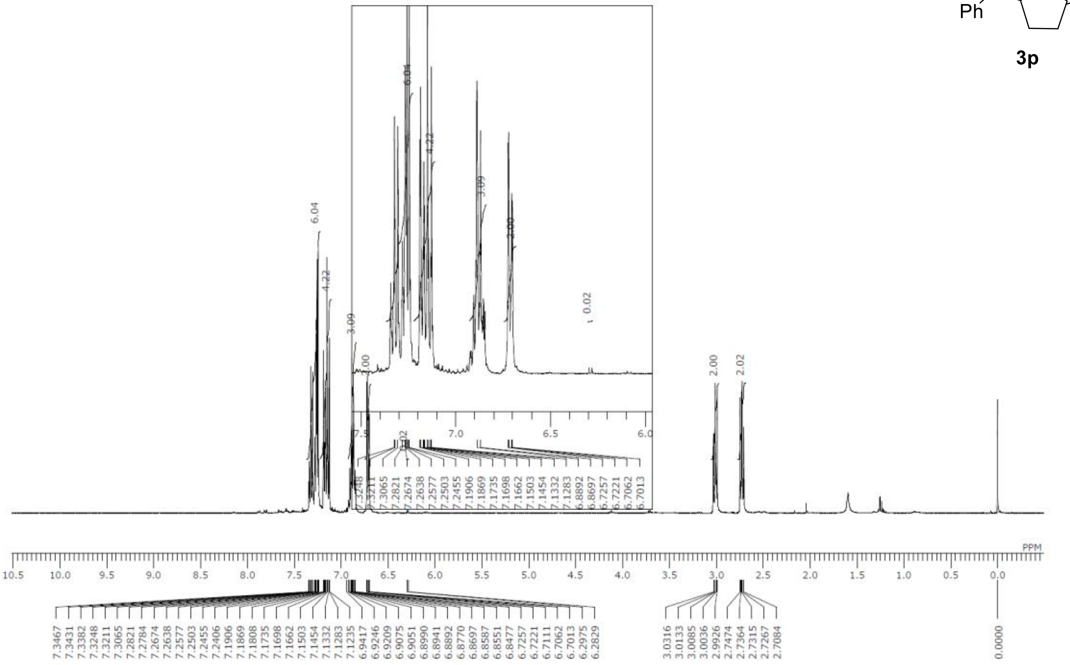

^{1}H-NMR spectrum of **3p** ($CDCl_3$, 600 MHz)

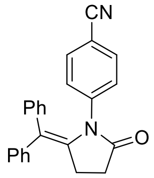

3p

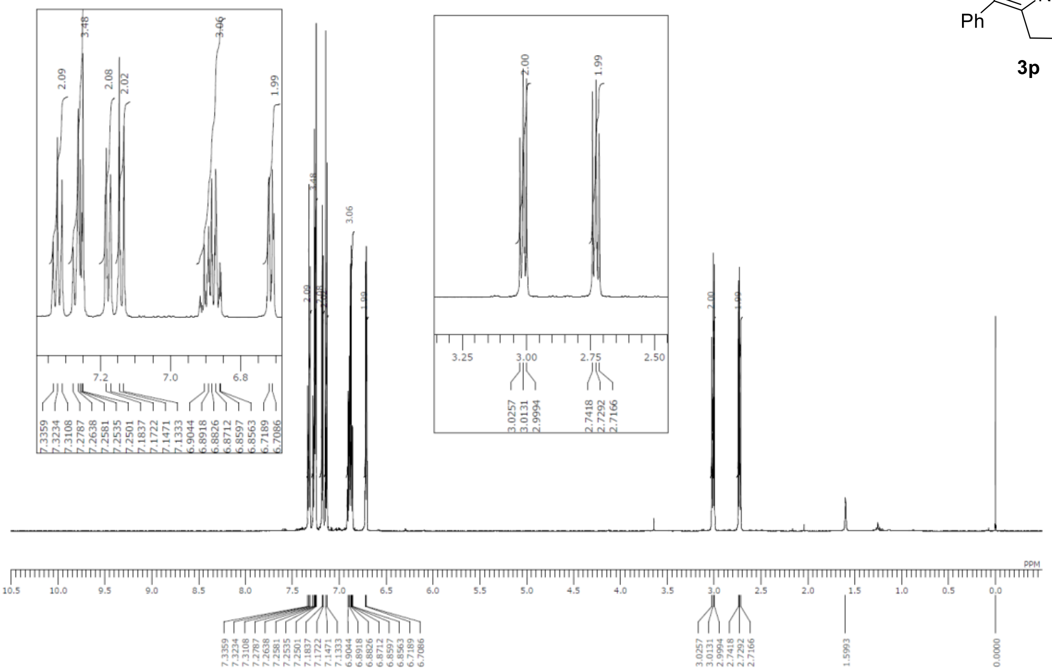

^{13}C-NMR spectrum of **3p** ($CDCl_3$, 150 MHz)

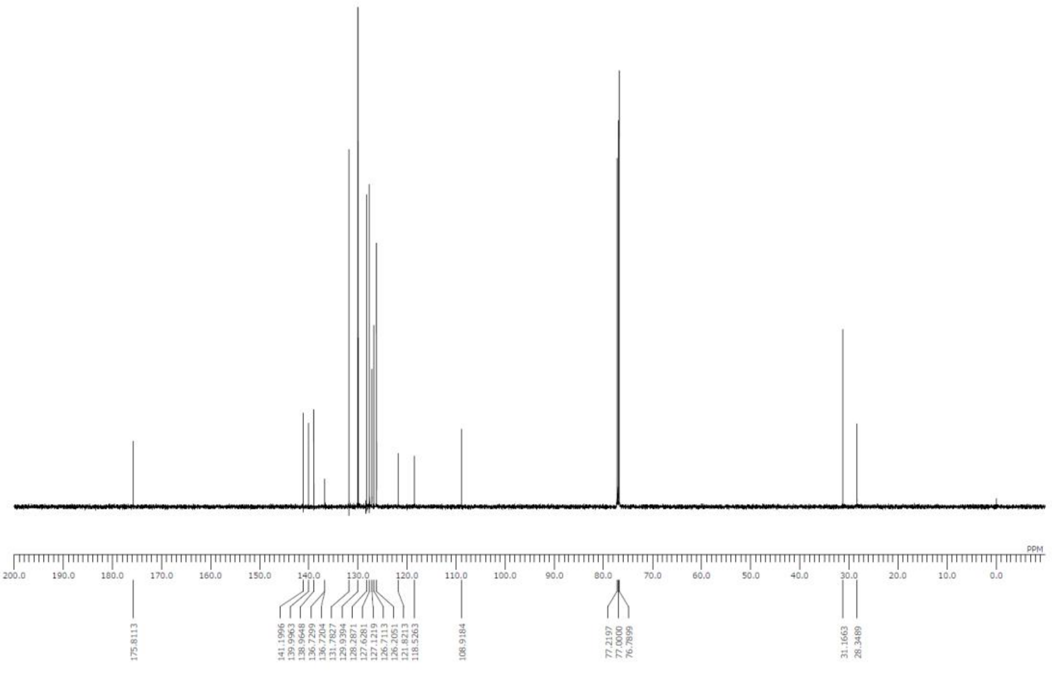

IR spectrum of **3p**

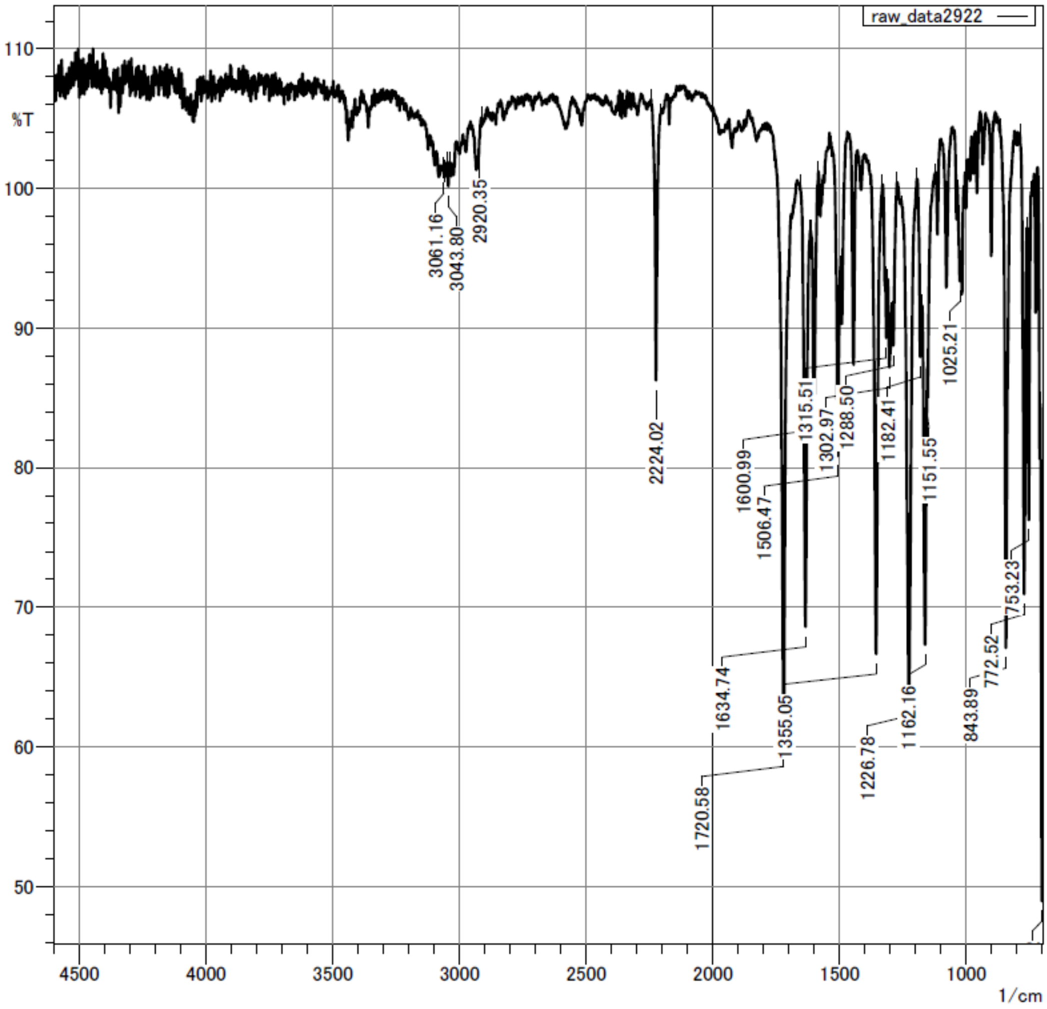

HRMS spectrum of **3p**

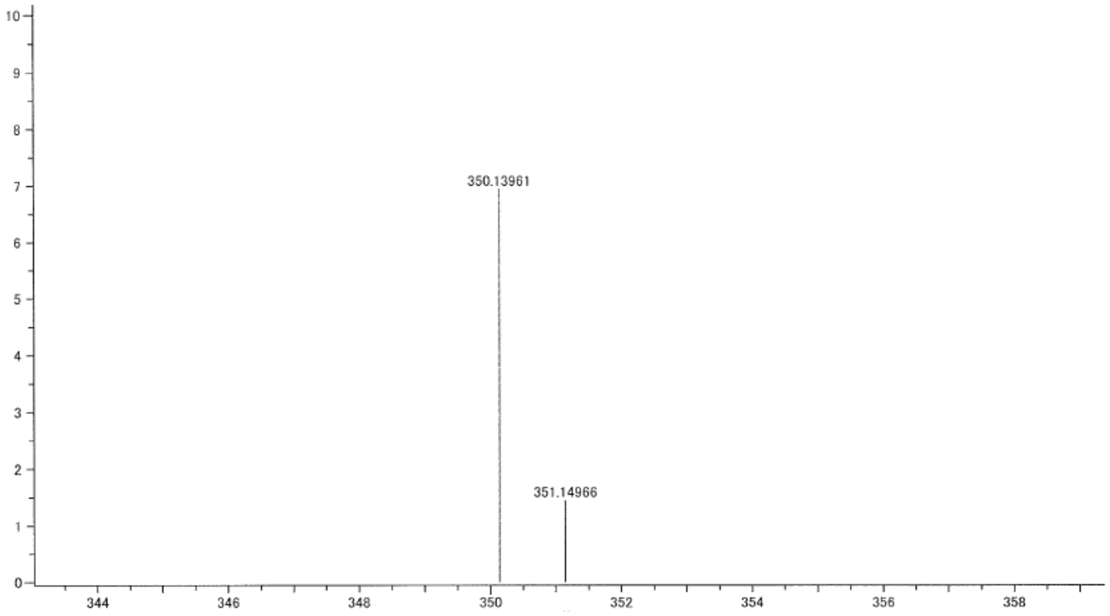

^{1}H-NMR spectrum of **3q** (**3q** : **2q** = 98 : 2, $CDCl_3$, 400 MHz)

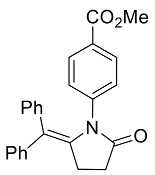

3q

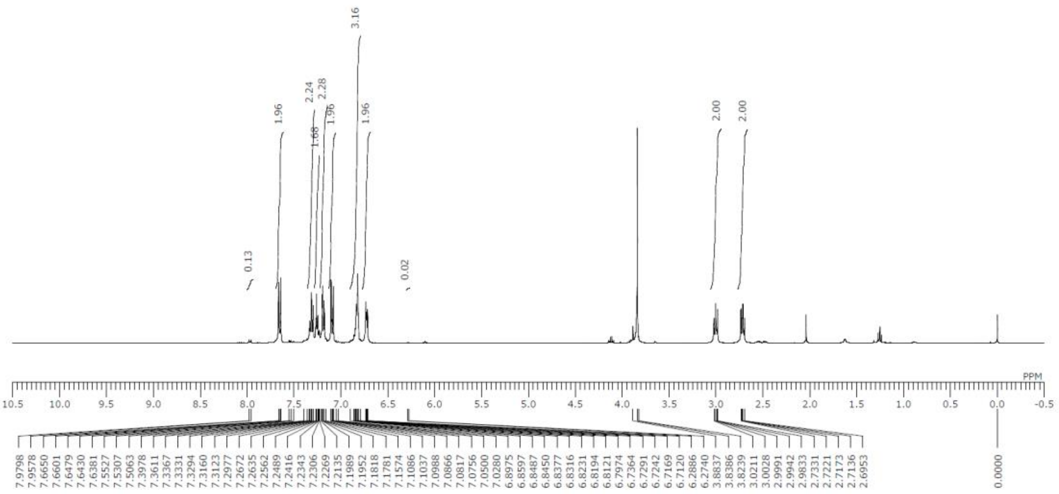

^{1}H-NMR spectrum of **3q** ($CDCl_3$, 600 MHz)

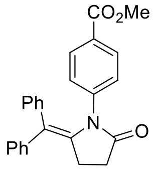

3q

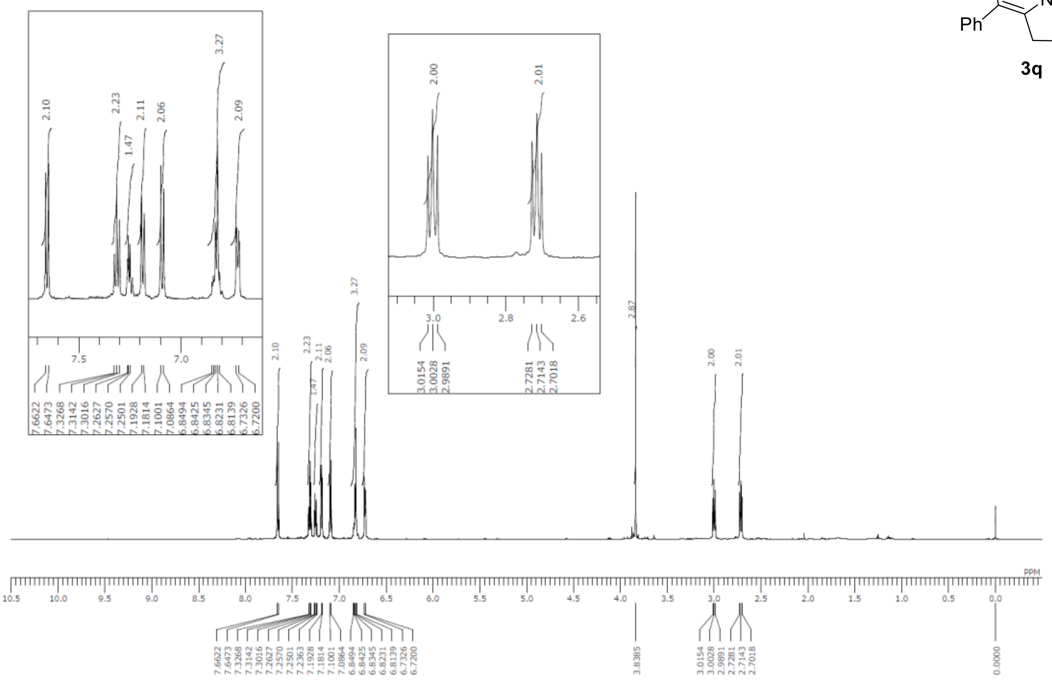

^{13}C-NMR spectrum of **3q** ($CDCl_3$, 150 MHz)

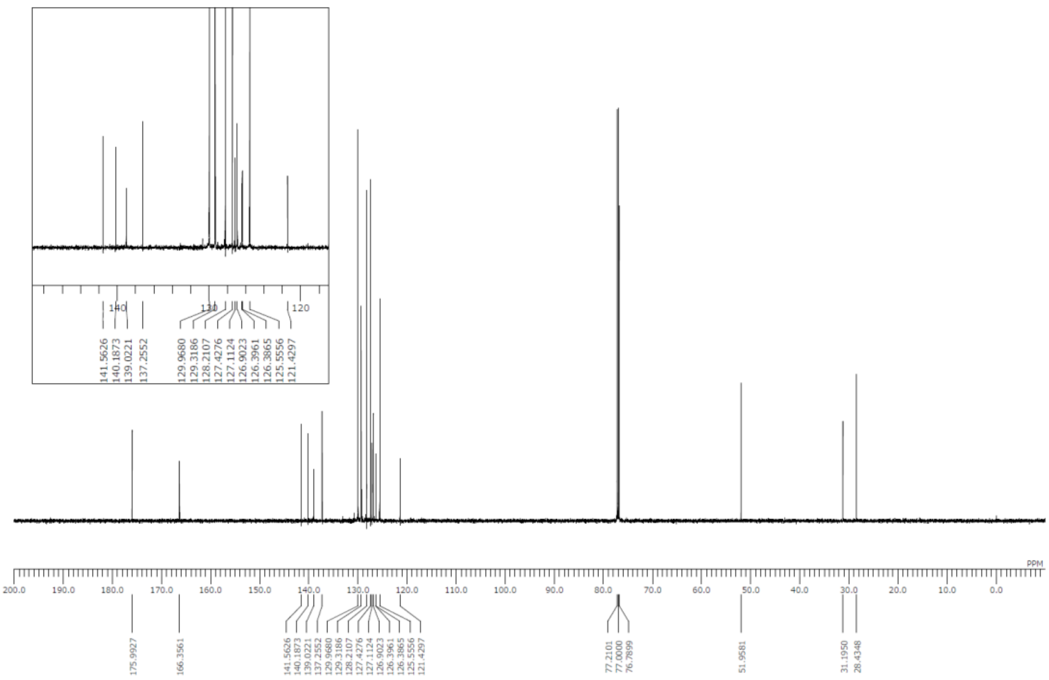

IR spectrum of **3q**

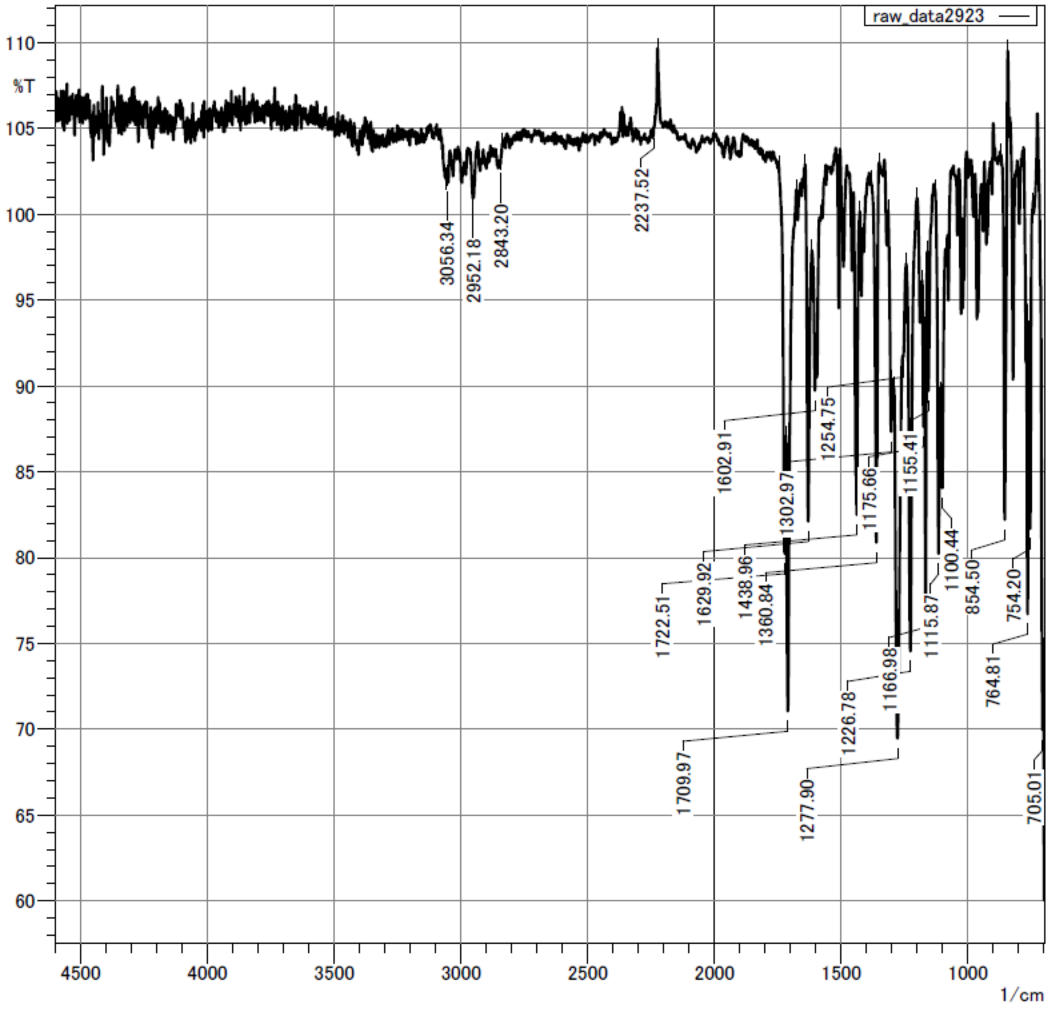

HRMS spectrum of **3q**

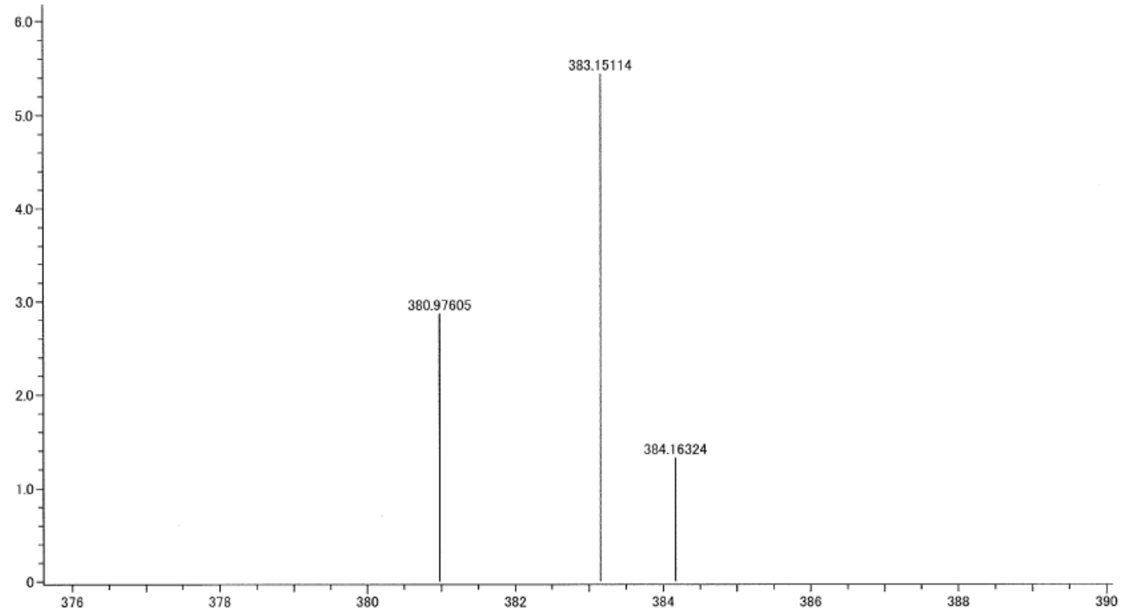

^{1}H-NMR spectrum of **3r** (**3r** : **2r** = 95 : 5, $CDCl_3$, 400 MHz)

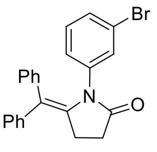

3r

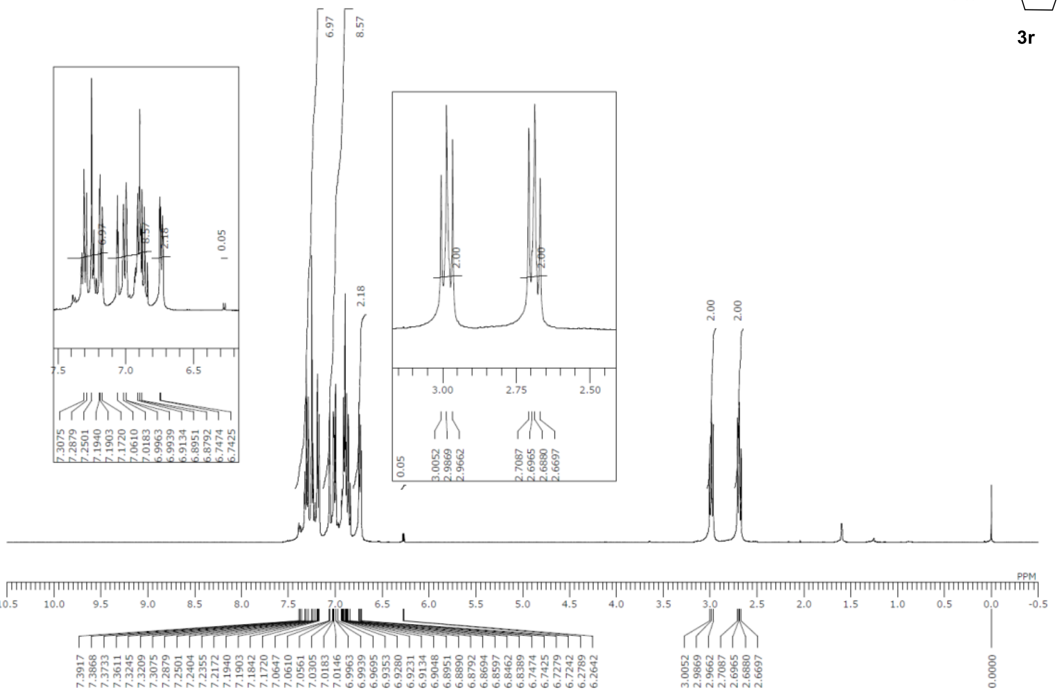

^{1}H-NMR spectrum of **3r** ($CDCl_3$, 600 MHz)

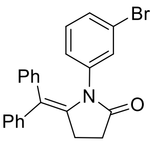

3r

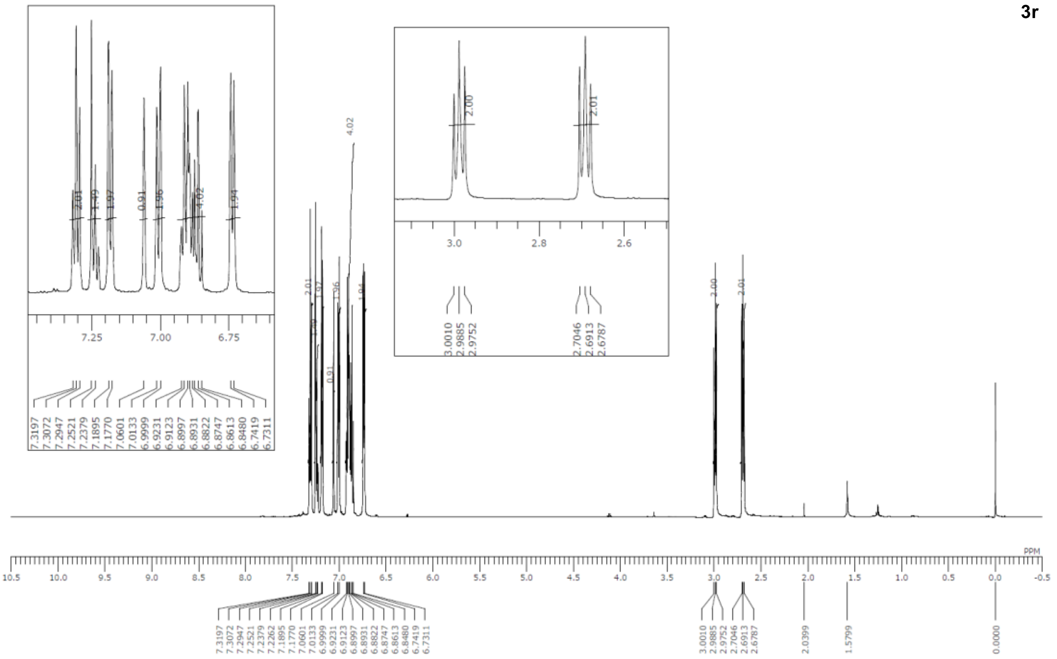

^{13}C-NMR spectrum of **3r** ($CDCl_3$, 150 MHz)

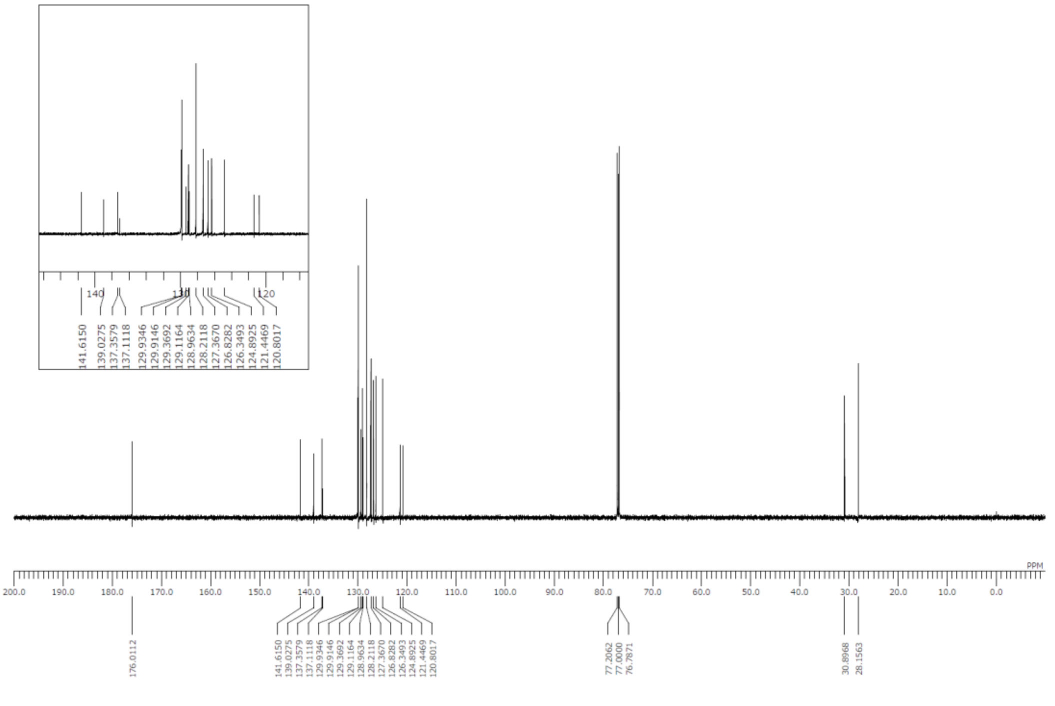

IR spectrum of **3r**

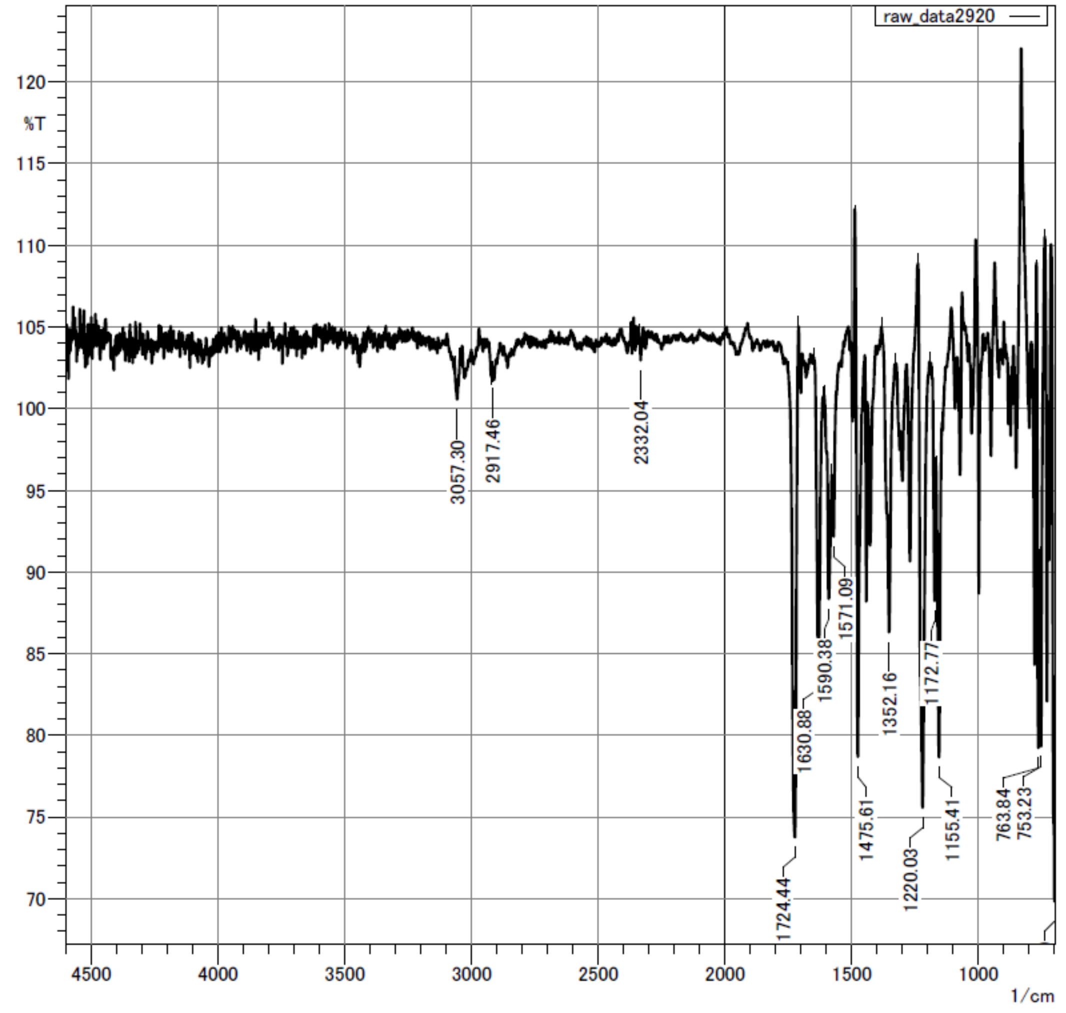

HRMS spectrum of **3r**

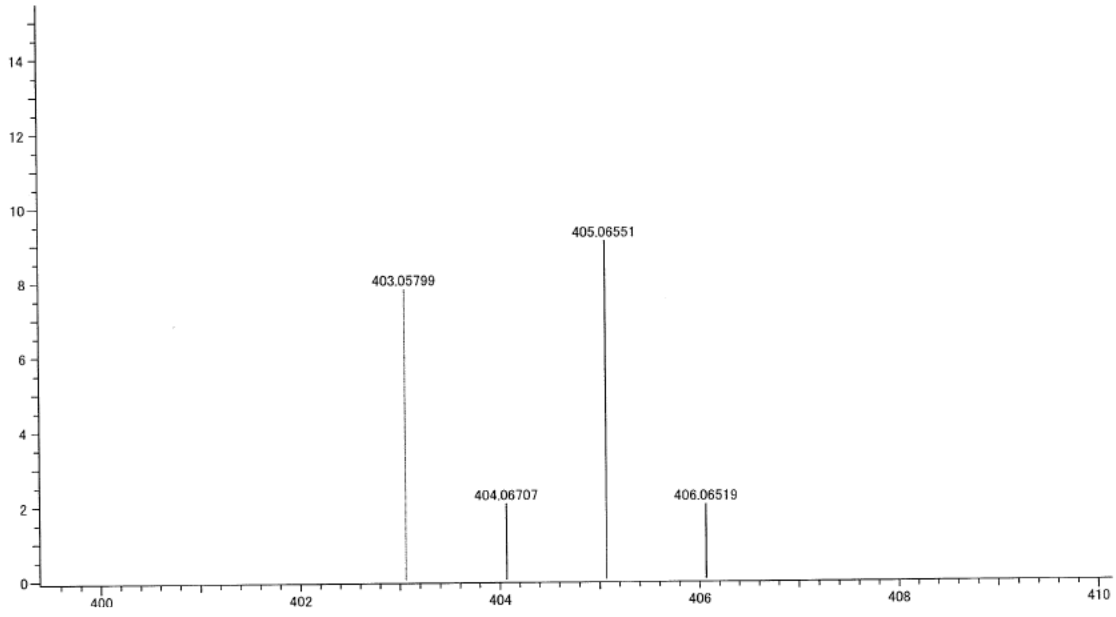

^{1}H-NMR spectrum of **3s** ($CDCl_3$, 600 MHz)

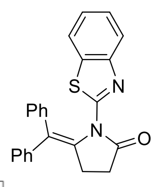

3s

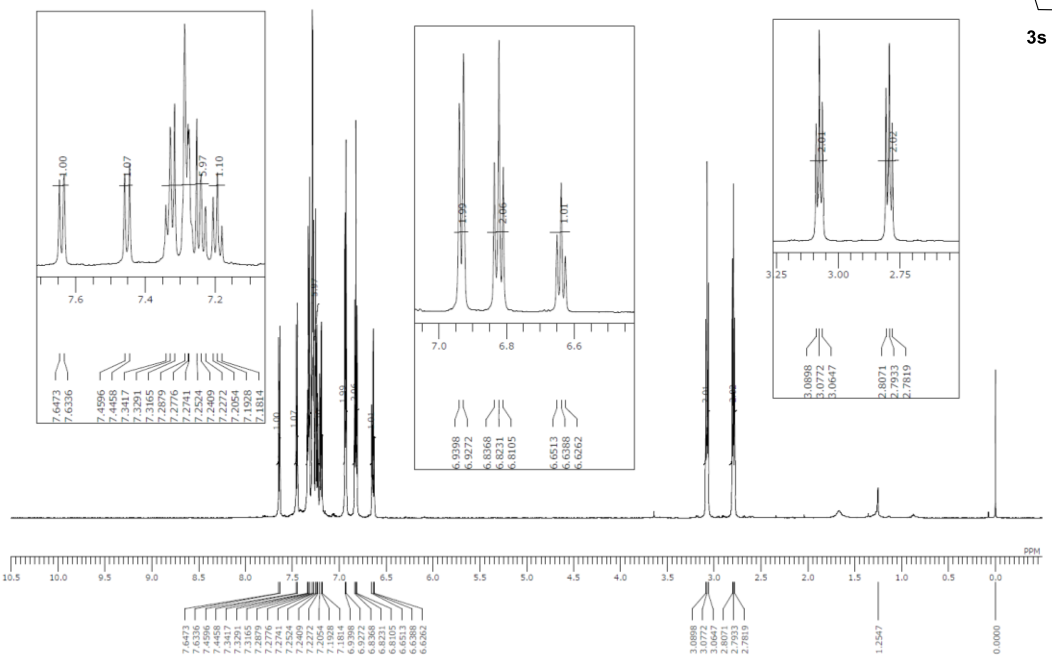

^{13}C-NMR spectrum of **3s** ($CDCl_3$, 150 MHz)

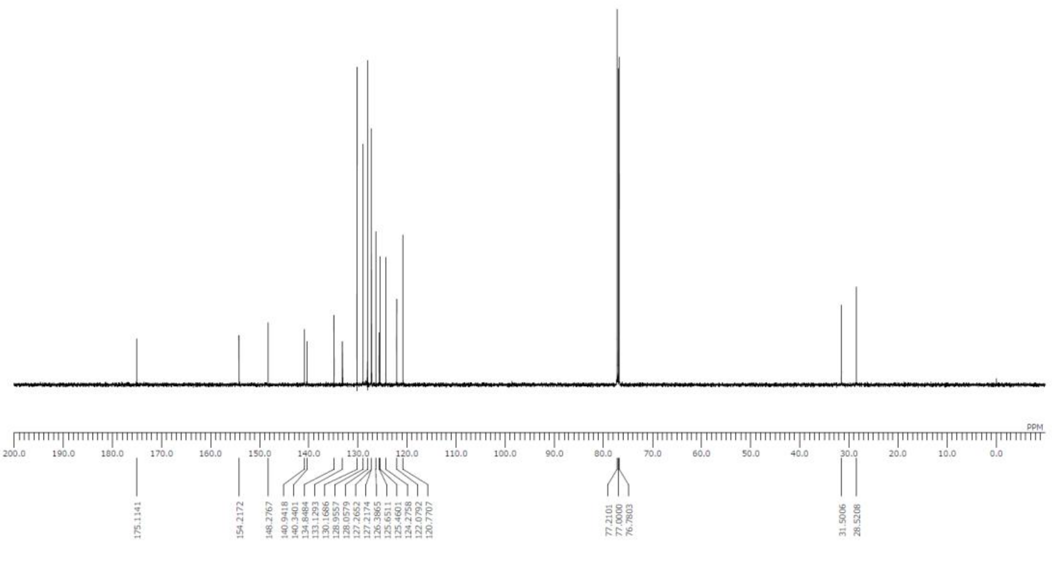

IR spectrum of **3s**

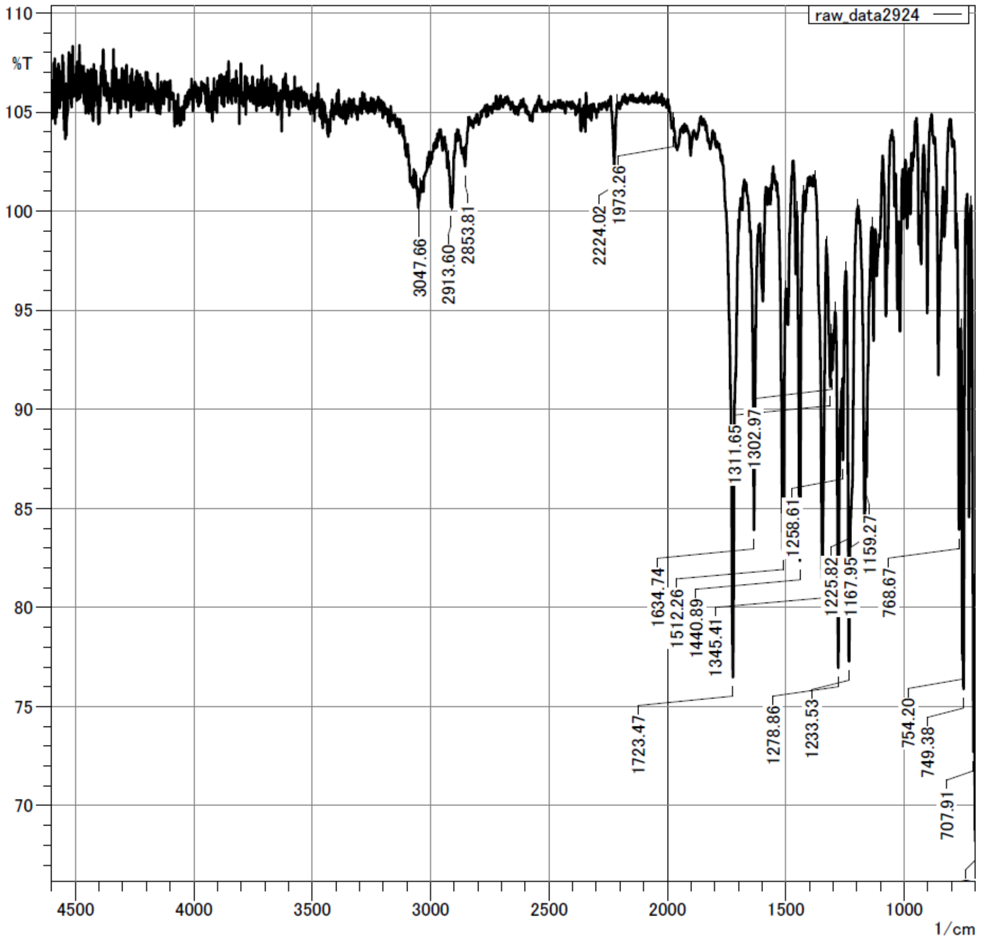

HRMS spectrum of **3s**

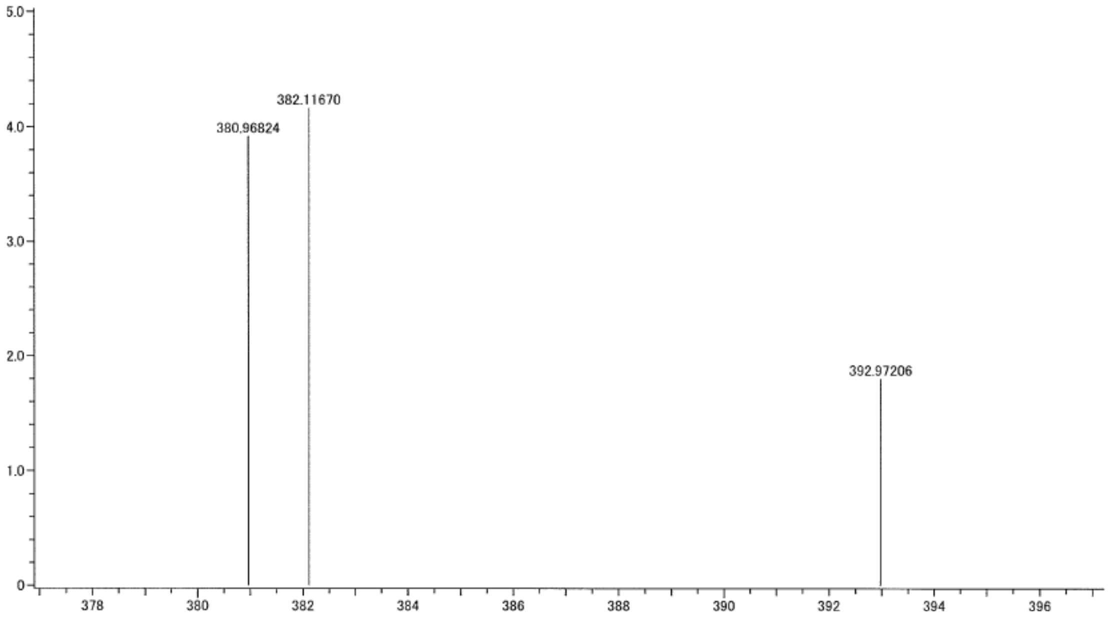

^{1}H-NMR spectrum of **4a** ($CDCl_3$, 600 MHz)

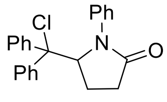

4a

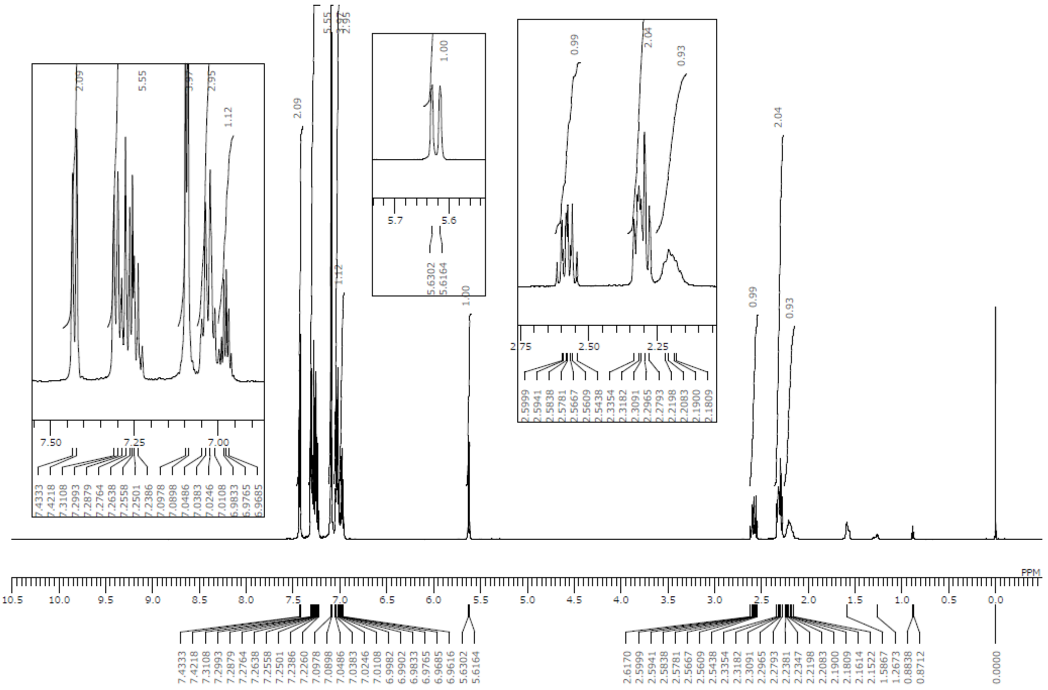

^{13}C-NMR spectrum of **4a** ($CDCl_3$, 150 MHz)

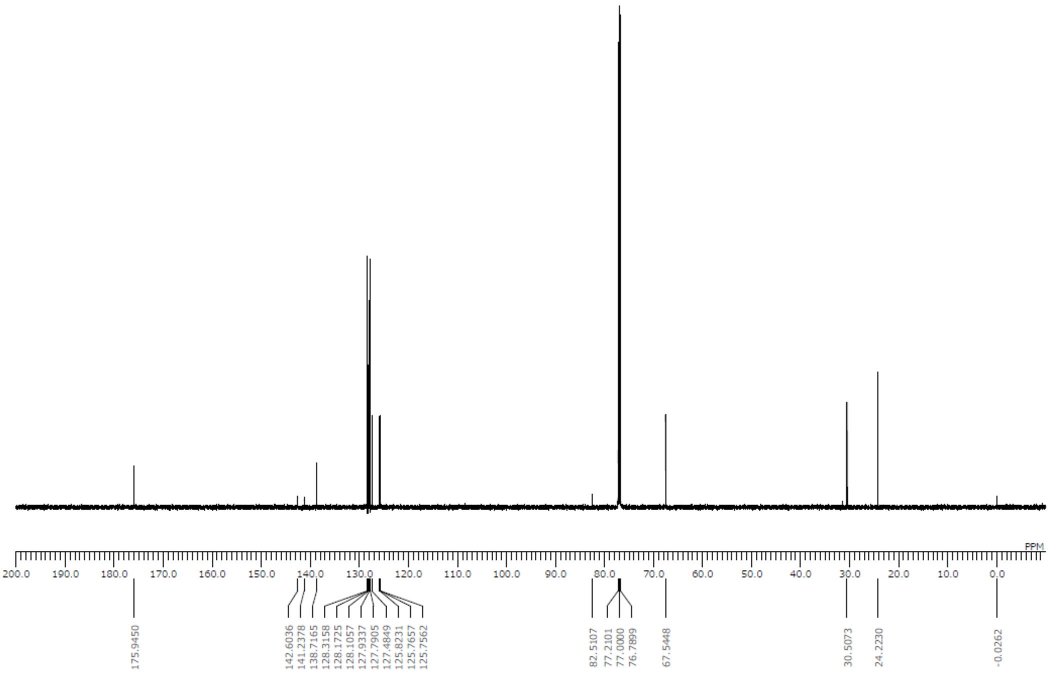

IR spectrum of **4a**

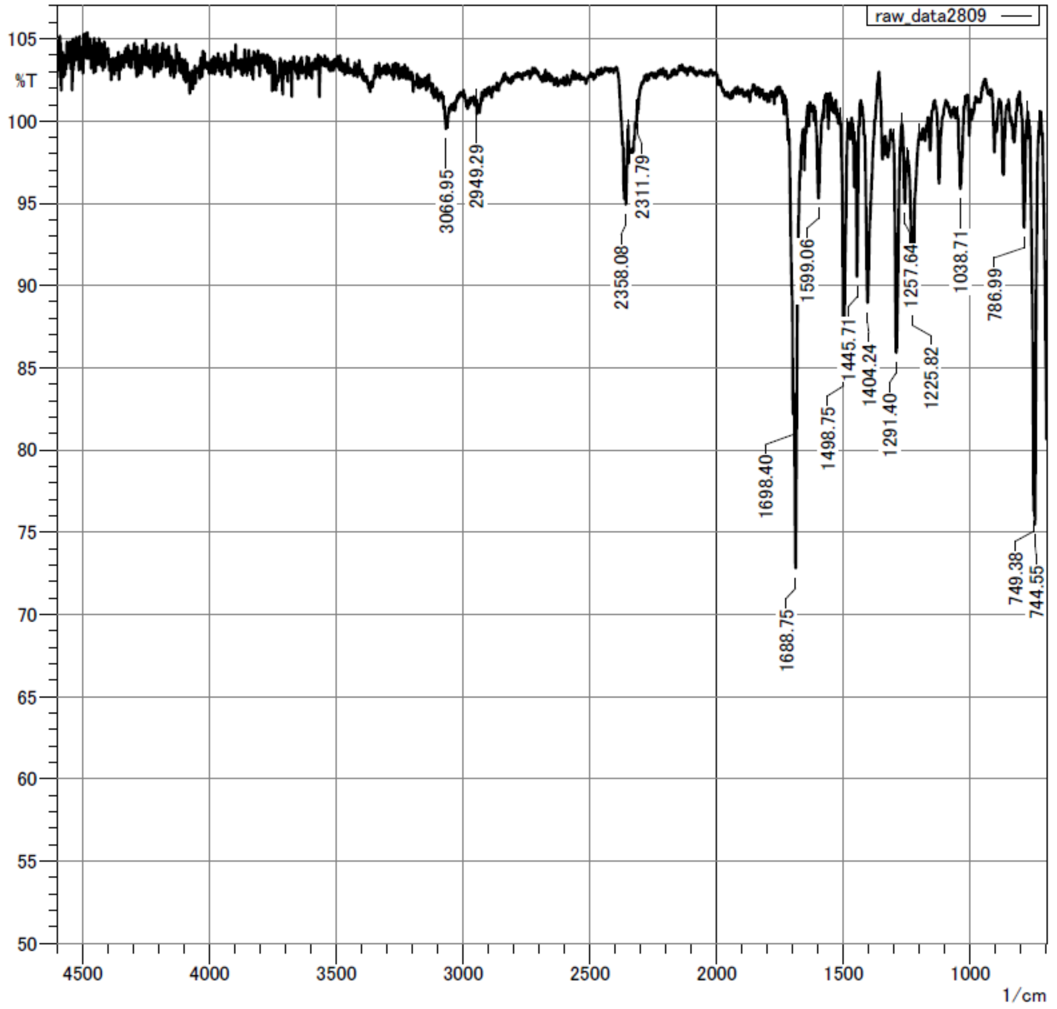

HRMS spectrum of **4a**

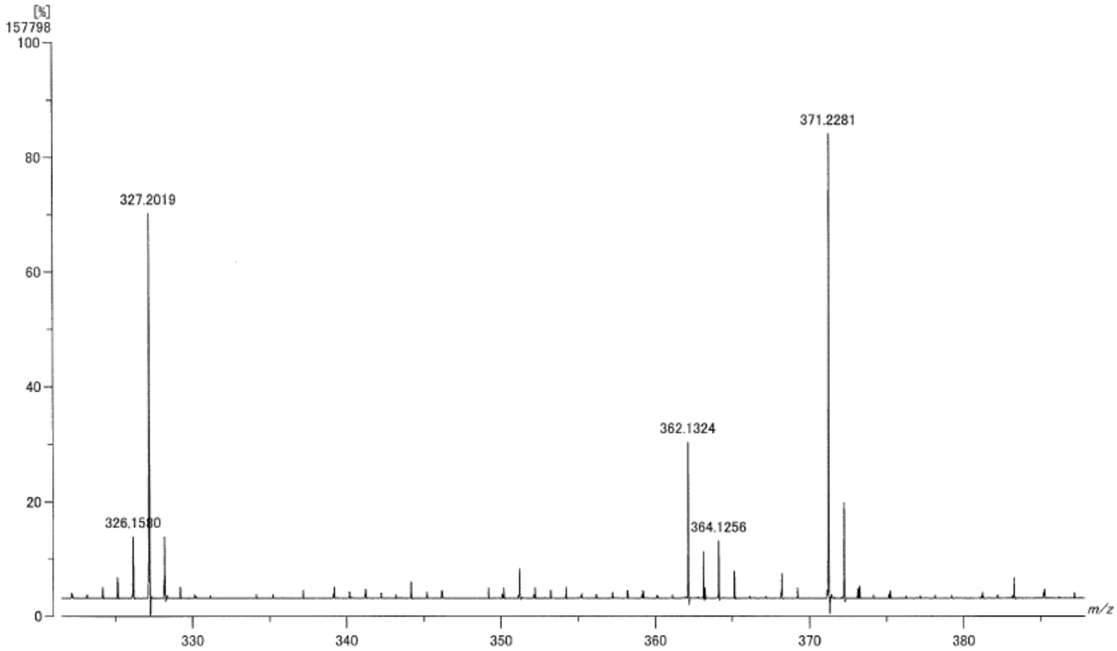

Supplement: Supplementary file 1 [file molecules-28-06682-s001.zip › molecules-2581133-supplementary.pdf]
